# Systematic review of dynamically tailored eHealth interventions targeting physical activity and healthy diet in chronic disease

**DOI:** 10.1038/s41746-025-02054-7

**Published:** 2025-11-19

**Authors:** E. A. G. Hietbrink, C. Lansink, G. D. Laverman, M. M. R. Vollenbroek-Hutten, A. Middelweerd, M. Tabak

**Affiliations:** 1 University of Twente, Biomedical Signals and Systems group, P.O. Box 217, 7500 AE Enschede, The Netherlands; 2 Ziekenhuisgroep Twente (ZGT), Department of Internal Medicine/Nephrology, P.O. Box 7600, 7600 SZ Almelo, The Netherlands; 3 Medisch Spectrum Twente (MST), Board of Directors, P.O. Box 50 000, 7500 KA Enschede, The Netherlands

**Keywords:** Nutrition, Patient education, Weight management, Cardiovascular diseases, Endocrine system and metabolic diseases, Metabolic disorders, Communication, Decision making, Education, Interdisciplinary studies, Psychology

## Abstract

This systematic review synthesized 61 dynamically tailored eHealth interventions for chronic disease management from 117 papers. Tailoring strategies varied in scope and complexity, with most targeting physical activity (87%) and nutrition (43%), while nearly three-quarters also integrated contextual, emotional, or physiological variables. Physical activity was often objectively measured (60%), but dietary intake remained self-reported (100%). Disease-specific biofeedback, such as glucose or blood pressure monitoring, was rare. Tailoring was predominantly rule-based (74%), though data-driven methods like machine learning (13%) are emerging. Most interventions used text-based delivery and drew on behavior change theory, particularly goal setting, self-monitoring, and feedback. While many showed positive within-group outcomes, benefits over controls were inconclusive. Progress within the field requires: (1) multidisciplinary development with rationale, (2) transparent reporting using structured frameworks, and (3) innovative evaluation designs to disentangle multi-component interventions. Strengthening methodological foundations is essential to unlock potential for delivering tailored lifestyle support in chronic disease care.

## Introduction

The significant impact of modern unhealthy lifestyles on the development and progression of chronic diseases is receiving growing attention^1–3^. Regular physical activity and a balanced diet are cornerstones in the management of chronic diseases. These behaviors are consistently emphasized in international guidelines across a wide range of conditions, including type 2 diabetes, cardiovascular disease, and COPD^4–6^. Additionally, several studies have demonstrated that adopting regular physical activity and a healthy diet can result in various health benefits, including greater quality of life, improved weight management, and reduced risk of disease-related complications^7–11^.

Unfortunately, adherence to physical activity and dietary recommendations among people with chronic diseases is often suboptimal^12–16^. Disease-related factors, such as the severity of the disease, the presence of symptoms, physical limitations, and comorbidities, can hinder the maintenance of a healthy lifestyle^17–20^. The complexities of managing chronic diseases often demand considerable time and effort, potentially leading to neglect of healthy lifestyle behaviors. In addition to their direct impact on health behaviors, the aforementioned factors can also negatively affect psychological determinants of an individual’s health behavior, such as outcome expectations, motivation, and self-efficacy^21,22^.

Each individual and every disease has unique characteristics, leading to different challenges in adopting a healthier lifestyle. Consequently, a one-size-fits-all approach to lifestyle interventions is insufficient to meet the needs of every individual. Moreover, such generic interventions often prove insufficient because they fail to adequately engage individuals’ motivation and perceived support for change. Research has shown that tailored interventions, which adapt to an individual’s characteristics, preferences, and needs, are more effective in promoting behavior change compared to generic interventions^23–25^. The fluctuating nature of disease symptoms requires lifestyle support that can be adjusted over time to match changes in health status and capabilities^26^. Adaptive tailored interventions can therefore provide more personalized and flexible support, which is essential in the long-term management of chronic conditions. In this way, tailored interventions might be better equipped to address the complex and evolving needs inherent to chronic disease management. The emergence of eHealth (i.e., using technology to support health, well-being, and healthcare^27^) offers a scalable solution for behavior change support as an addition to or replacement for regular care. Prior research has demonstrated the value of eHealth in improving physical activity and dietary behavior^28–31^, as well as enhancing overall quality of life and health outcomes, such as weight management, blood pressure control, cholesterol levels, and glycemic control^30,32–35^.

eHealth technologies are increasingly utilizing data from smartphone sensors, wearable medical devices, remote monitoring devices, and ecological momentary assessments (EMA; repeated real-time sampling of individuals’ current behaviors in their natural environments)^36,37^ to tailor interventions. The use of (real-time) data not only provides greater insight into the dynamics of health behavior, but also allows for more dynamically tailored eHealth interventions^37,38^. In contrast to static tailored interventions which rely on a single baseline assessment, dynamic tailoring incorporates ongoing information about the individual to iteratively adapt the content, amount, or timing of support to their changing behaviors, circumstances, and context^39^. Literature shows promising results suggesting that dynamically tailored interventions may be more effective in promoting health behaviors than static tailored interventions^39,40^. Krebs et al.^39^ demonstrated that computer-tailored interventions produced clinically meaningful effects across multiple health behaviors, with dynamic tailoring showing greater efficacy over time than static tailoring. More recently, a meta-analysis by Wang et al.^40^ found similar results across a range of behaviors and populations, with evidence that tailoring both to past behavioral patterns and to current needs improved effectiveness.

Different scientific fields use various terms to refer to tailored interventions that use repeated assessments over time^38^. In addition to dynamically tailored interventions, some of these terms include highly personalized interventions, context-aware interventions, real-time interventions, ecological momentary interventions (EMIs), just-in-time interventions (JITs), and just-in-time adaptive interventions (JITAIs). Furthermore, there are major differences in how the support is tailored to an individual, including the data sources utilized (such as wearables^41^, GPS^42^, and EMAs^43^), the type of support offered (such as feedback, prompts, and reminders^44^), and the timing of support^44^. For example, the intensity of dynamic tailored support can vary from providing extensive tailored advice once a week to providing the right support at the right time, as with JITAIs.

Several reviews examined dynamically tailored interventions aimed at promoting various health behaviors^40,45–50^, with most reviews focusing on JITAIs. The effectiveness of dynamically tailored interventions on behavior change has shown mixed results, partly due to limited statistical power in most studies^40,45–47,50^. Some tailoring aspects have been associated with greater efficacy, particularly (1) tailoring interventions based on both prior behavior and current needs and (2) combining algorithm-driven feedback with human guidance^40^. Additionally, dynamically tailored interventions frequently incorporate key behavior change techniques (BCTs)^51^, such as goal setting, self-monitoring, feedback on behavior, action planning, social support, and prompts/cues^45,46,48,50^. However, despite the promising potential of these interventions, many studies provide insufficient detail on intervention infrastructure and methodologies, making replication and further development challenging^46,49^.

The field of dynamically tailored interventions is rapidly evolving, encompassing a broad range of intervention approaches. However, a comprehensive understanding of dynamically tailored eHealth interventions for individuals with chronic diseases remains lacking. A holistic understanding of these interventions is essential, as multiple intervention aspects can influence their impact on health outcomes. Specifically, further insights are needed into how tailoring is operationalized in these interventions, the defined objectives, the underlying theoretical foundations, and the way interventions are delivered. To address these gaps, this systematic review aims to provide a broad overview of dynamically tailored eHealth interventions that support physical activity and healthy nutrition in individuals with chronic diseases.

The aim of this study was to systematically review dynamically tailored eHealth interventions that support physical activity or healthy nutritional behaviors in people with chronic diseases. We aimed to address the following research questions:

1. How are the key components of dynamically tailored eHealth interventions for promoting physical activity and a healthy diet operationalized?
2. In which way are eHealth interventions to promote physical activity or healthy nutritional behaviors delivered?
3. What behavior change theory and techniques are applied in dynamically tailored eHealth interventions to promote physical activity and healthy nutritional behaviors?
4. What are the findings from the studies on dynamically tailored eHealth interventions regarding user experiences, engagement and effects on behavioral and health-related outcomes?

## Results

### Study selection

**Fig. 1:**
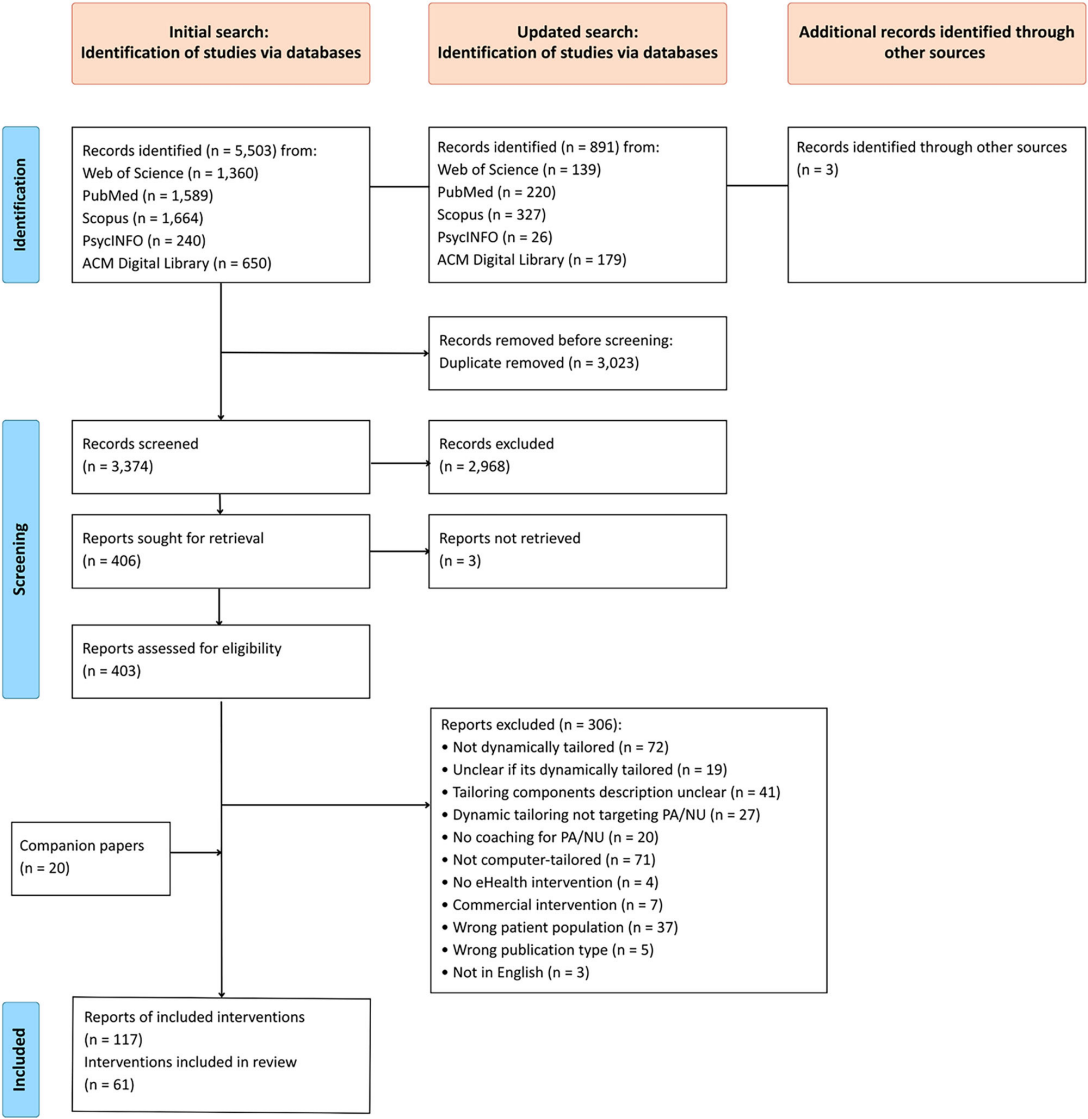
PRISMA flowchart of study selection through the systematic review process. This figure presents the Preferred Reporting Items for Systematic Reviews and Meta-Analyses (PRISMA) flowchart, which illustrates the process of identifying, screening, and including studies. It shows the number of records identified through database searches, the number after removal of duplicates, and the number screened by title, abstract, and full text. Reasons for exclusion at the full-text stage are specified. PA physical activity, NU nutrition.

The search identified 6397 records. After removing duplicates, 3374 reports were screened based on title and abstract. During this phase, the inter-rater agreement (IRA) varied per reviewer pair, ranging from 87.6% to 92.7%, with an overall IRA of 90.7%. A total of 403 records were assessed for eligibility through full-text screening. The IRA for full-text screening ranged from 83.2% to 90.3%, with an overall IRA of 87.8% and Cohen’s kappa values between 0.65 and 0.80, indicating substantial agreement. Ultimately, 117 reports, covering 61 unique interventions, were included in the final analysis. Figure 1 provides an overview of the selection process.

### Study characteristics

An overview of all study characteristics per intervention (*n* = 61) is available in Supplementary Data 1. The included reports (*n* = 117) comprised a variety of study designs. The majority were study protocols (*n* = 28, 23.9%) and full randomized controlled trials (RCTs) (*n* = 26, 22.2%). A considerable number of studies focused on intervention development or design (*n* = 25, 21.4%) or reported on pilot or feasibility RCTs (*n* = 20, 17.1%). A smaller portion of the studies employed secondary analyses of RCT data (*n* = 7, 6.0%), qualitative or mixed-methods designs (*n* = 5, 4.3%), non-randomized quantitative designs (*n* = 4, 3.4%), or usability studies (*n* = 2, 1.7%). The included interventions were conducted across a wide range of countries, most commonly in the United States (*n* = 32, 52.5%), the Netherlands (*n* = 7, 11.5%), Belgium (*n* = 3, 4.9%), and the United Kingdom (*n* = 3, 4.9%). Some studies were multinational, resulting in a total number of countries greater than the number of included interventions. The interventions targeted most frequently type 2 diabetes (*n* = 22, 36.1%), overweight or obesity (*n* = 20, 32.8%), cardiovascular disease (*n* = 10, 16.4%), and hypertension (*n* = 7, 11.5%). Regarding the primary lifestyle behaviors included in this review, interventions focused on physical activity (*n* = 50, 82.0%), nutrition (*n* = 30, 49.2%), and sedentary behavior (*n* = 10, 16.4%). Additionally, medication adherence (*n* = 3, 4.9%), sleep (*n* = 2, 3.8%), stress management (*n* = 1, 1.6%), and mental health (*n* = 1, 1.6%) were also addressed as lifestyle behaviors in a subset of the interventions.

### Key components of tailoring

An overview of the tailoring strategies used per intervention is provided in Supplementary Data 2.

**Fig. 2:**
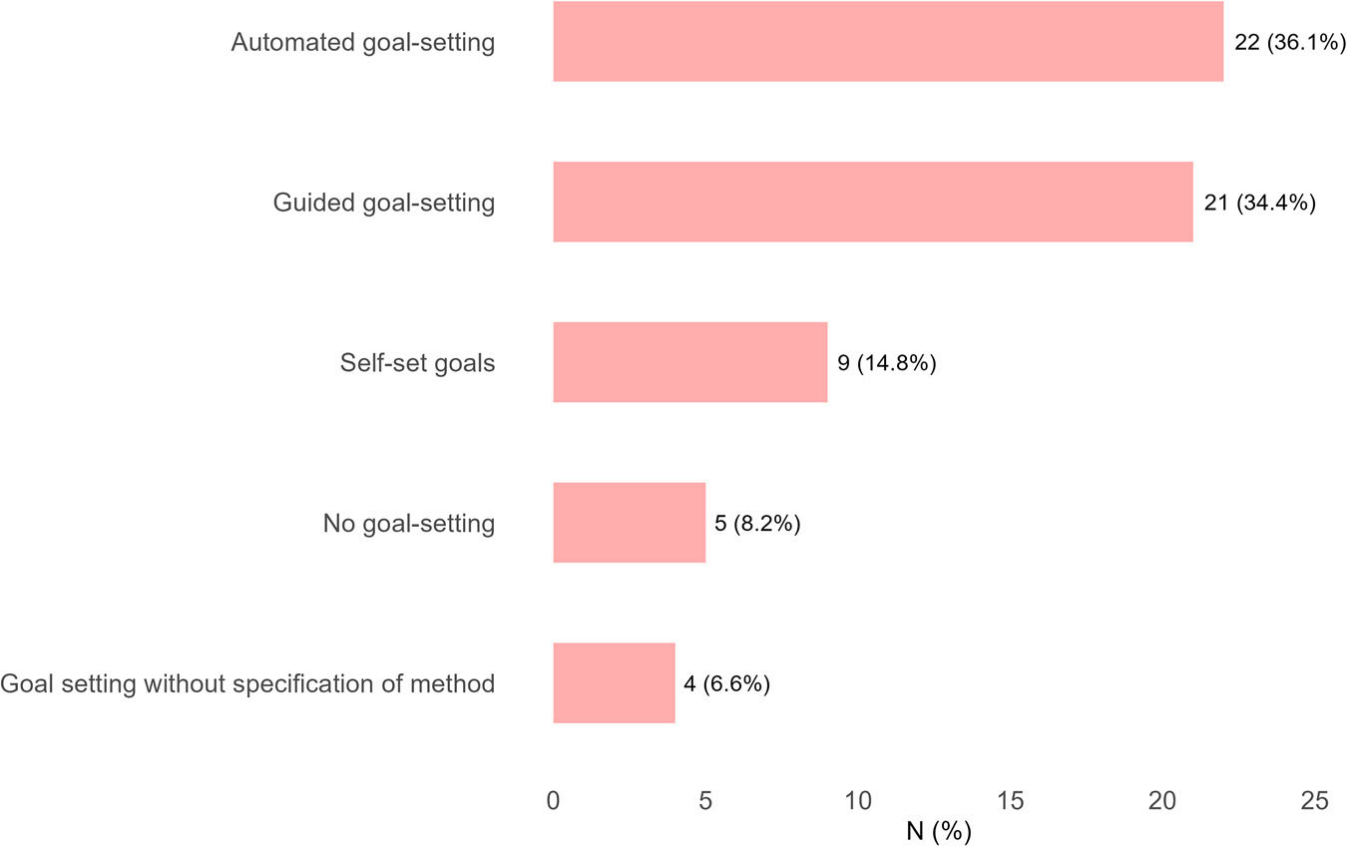
Goal setting strategies in dynamically tailored eHealth interventions. This figure presents the frequency of different types of goal setting strategies reported across interventions. Bars show the absolute number and percentages of studies using each option. The figure refers to whether intervention goals were set automatically by the intervention, guided with a professional or coach, independently by the user, or that no goal-setting was applied within the intervention.

Figure 2 displays the frequency and percentage of each goal-setting method. Most interventions facilitated either automated or guided goal setting. Specifically, 22 interventions (36.1%) used automated goal setting, in which goals were set for the user based on predefined criteria or algorithmic rules. A similar proportion (*n* = 21, 34.4%) employed guided goal setting, where goals were collaboratively developed with the support of the intervention system or a health professional. In 9 interventions (14.8%), participants were allowed to set their goals by themselves. Furthermore, 5 interventions (8.2%) did not include any goal-setting component, while in 4 interventions (6.6%) goal setting was present but the method was not specified.

In 43 of the 61 interventions (70.5%), goals could be adapted over time based on user progress or changing circumstances. Conversely, in 10 interventions (16.4%) goals were static and remained unchanged throughout the intervention. For the remaining interventions using goal-setting, it was not reported whether goals could be adapted.

**Fig. 3:**
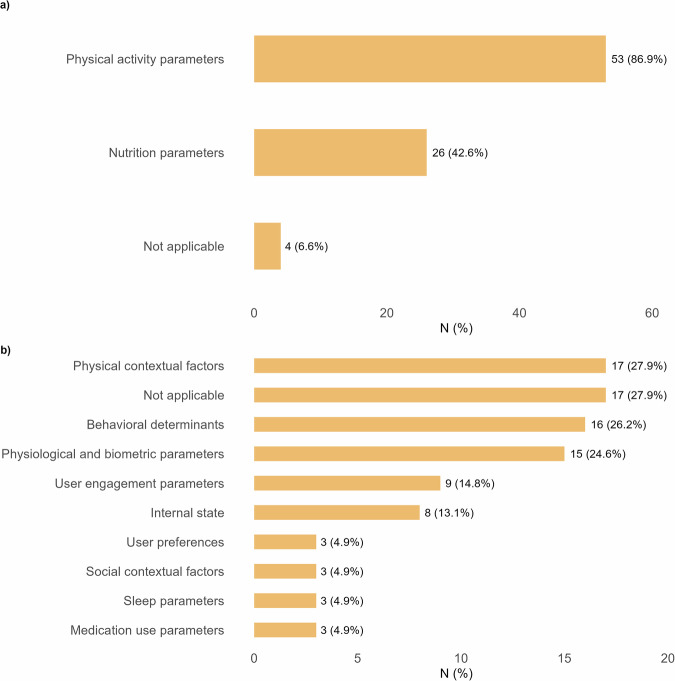
Types of tailoring variables used in dynamically tailored eHealth interventions. This multi-panel figure shows the types of tailoring variables applied across the included interventions. Panel **a** presents the primary tailoring variables, including lifestyle-related parameters, or whether the paper did not specify any primary tailoring variable (not applicable). Panel **b** presents the secondary tailoring variables, which captured additional dimensions beyond lifestyle behavior. These included physical contextual factors (e.g., location and time of day), behavioral determinants (e.g., motivation and self-efficacy), physiological and biometric parameters (e.g., weight and glucose), and user engagement parameters (e.g., app use and response rate), internal states (such as mood), user preferences, social contextual factors, sleep, or medication use.

On average, interventions used 2.6 different categories of tailoring variables (SD = 1.6), ranging from 1 to 7 categories per intervention. Figure 3 shows the frequency and percentage of each tailoring variable category. Tailoring was realized using a wide range of dynamic variables, with support adapted based on multiple measurements over time. The majority of tailoring variables (Fig. 3a) were directly related to the intervention targets, including physical activity parameters (*n* = 53, 86.9%, e.g., steps, sedentary time) and nutrition parameters (*n* = 26, 42.6%, e.g., caloric intake, fruit and vegetable consumption). For a small proportion of interventions, no physical activity or dietary tailoring variables were applicable (*n* = 4, 6.6%). Within physical activity tailoring (*n* = 53), most interventions relied on step counts (*n* = 21, 39.6%) or unspecified physical activity measures (*n* = 18, 34.0%). Sedentary time (*n* = 9, 17.0%) and active minutes (*n* = 6, 11.3%) were also applied, while other indicators such as walking speed, calorie expenditure, or floors climbed were rarely used. For nutrition (*n* = 26), tailoring most often targeted general or unspecified eating behavior (*n* = 10, 38.5%) or overall calorie intake (*n* = 3, 11.5%). More specific dietary indicators, such as macronutrient composition (*n* = 4, 15.4%), alcohol or salt intake (each *n* = 2, 7.7%), or fruit and vegetable consumption (each *n* = 2, 7.7%), were less frequently employed.

Other dynamic tailoring variables (Fig. 3b) reflected contextual, behavioral, or individual factors. Frequently reported were physical contextual factors (*n* = 17, 27.9%, e.g., location and weather), behavioral determinants (*n* = 16, 26.2%, e.g., motivation, self-efficacy), and physiological or biometric parameters (*n* = 15, 24.6%, e.g., weight and blood pressure). Less commonly reported were user engagement parameters (*n* = 9, 14.8%, e.g., app use frequency), internal states (*n* = 8, 13.1%, e.g., stress and mood), medication use parameters (*n* = 3, 4.9%), sleep parameters (*n* = 3, 4.9%), social contextual factors (*n* = 3, 4.9%), and user preferences (*n* = 3, 4.9%).

In addition, 52.4% (*n* = 32) of interventions reported also using static tailoring variables, with support based on a single assessment. These variables mostly included demographic factors (*n* = 14, 23.0%, e.g., age and gender), behavioral determinants (*n* = 12, 19.7%, e.g., self-efficacy and attitude), and health status and medical history (*n* = 8, 13.1%, e.g., comorbidities). Some interventions also incorporated physical or social contextual information, personal goals and preferences, or behavioral patterns.

**Fig. 4:**
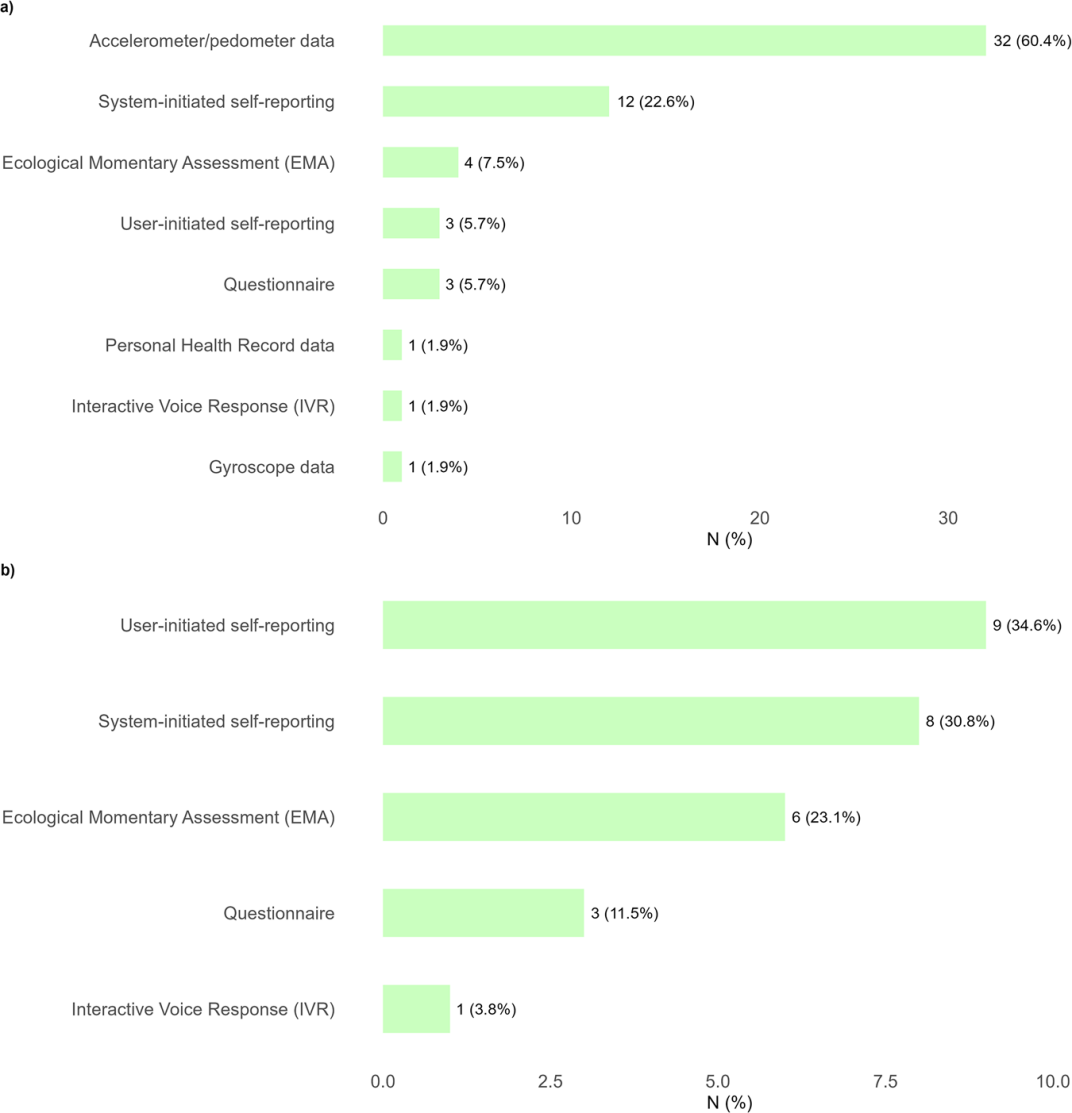
Data sources used to tailor to physical activity and dietary intake. This figure presents the measurement methods applied to assess tailoring variables for physical activity and nutrition. Panel **a** shows the data sources used for tailoring to physical activity, such as accelerometer or pedometer data, system-initiated self-reporting, or ecological momentary assessment (EMA). Panel **b** shows the data sources used for tailoring to dietary intake, such as user-initiated self-reporting, system-initiated self-reporting, or EMA.

Figure 4 provides an overview of the sources of data used to trigger dynamic tailoring and the monitoring devices applied within these interventions. The most frequently used data source for physical activity or sedentary behavior were accelerometers or pedometers, reported in 32 interventions (60.4%) (Fig. 4a). In addition, system-initiated self-reporting (*n* = 12, 22.6%) and EMAs (*n* = 4, 7.5%) were commonly applied. For dietary intake, the most common sources were user-initiated self-reporting (e.g., manual input in food diaries; *n* = 9, 34.6%) and system-initiated self-reporting (e.g., SMS prompts; *n* = 8, 30.8%), while EMA was used in 6 interventions (23.1%) (Fig. 4b). Behavioral determinants were mainly captured through system-initiated self-reporting (*n* = 5, 31.2%), EMA (*n* = 4, 25.0%), and questionnaires (*n* = 3, 18.8%). Internal states such as mood or stress were primarily assessed with EMA (*n* = 5, 62.5%). Physiological and biometric parameters were most often collected using device-measured vital signs or body parameters (*n* = 8, 53.3%), complemented by system-initiated self-reporting (*n* = 4, 26.7%). Physical contextual factors were less consistently reported, with GPS (*n* = 5, 29.4%) and weather or time-based APIs (*n* = 4, 23.5%) being the most frequently applied, while 9 interventions (52.9%) did not specify the method. Less frequently used approaches across all variables included interactive voice response, personal health record data, gyroscope data, and telephone-based counseling, which were applied only in a few interventions.

Regarding monitoring devices, more than half of the interventions (*n* = 32, 52.5%) included an activity tracker as part of the intervention. In 10 interventions (16.4%), a smart scale was used to monitor body weight, while 6 interventions (9.8%) used smart blood pressure monitors. Smartwatches were applied in 5 interventions (8.2%), and blood glucose monitors in 4 interventions (6.6%). Less common monitoring systems included APIs for activity data (*n* = 3, 4.9%) and electronic pill boxes (*n* = 2, 3.3%). In 18 interventions (29.5%), no monitoring device was used, while in 1 case (1.6%) the type of monitoring device was not reported. In total, 32 of the 61 interventions (52.5%) used monitoring devices to inform tailoring, whereas 9 (14.8%) did not, and for 20 interventions (32.8%) this was not applicable.

**Fig. 5:**
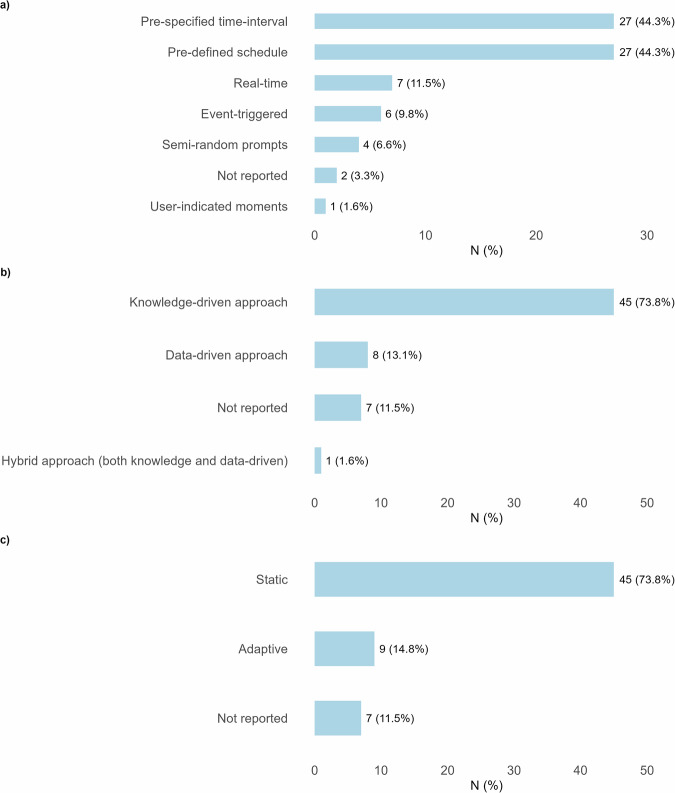
Types of decision points and decision rules used in dynamically tailored eHealth interventions. This figure illustrates the approaches applied for defining decision points and decision rules in dynamically tailored eHealth interventions. Panel **a** shows the types of decision points reported. These include pre-specified time intervals, pre-defined schedules, real-time triggers, event-triggered prompts, semi-random prompts, and user-indicated moments. Panel **b** presents the types of decision rules used. Approaches included knowledge-driven rules, data-driven rules, and hybrid approaches combining both knowledge-based and data-based inputs. Panel **c** shows the adaptivity of decision rules. Decision rules were either static, remaining the same throughout the intervention, or adaptive, allowing modification over time based on accumulated data or user progress.

The duration of the intervention varied widely, ranging from 4 weeks to 48 months. Only 3 of the 61 interventions (4.9%) described a rationale for the choice of the intervention duration. A variety of decision point strategies were identified across interventions (Fig. 5a). The most common approaches were pre-defined schedules (e.g., fixed times of day, weekly or monthly prompts) and pre-specified time intervals (e.g., prompts every 10–15 min, or daily/weekly assessments). Each of these strategies was applied in 27 interventions (44.3%), although the frequency and structure of decision points varied widely within these categories. For example, schedules ranged from a few prompts per month to multiple messages per day, while intervals ranged from very short (every minute) to longer cycles (weekly or monthly adjustments). For most interventions, no justification was provided for the choice of decision points. Other approaches were less frequently applied. Real-time decision points, often linked to behavior or physiological data, were reported in 7 interventions (11.5%). Event-triggered decision points, activated by specific user actions or biometric thresholds, were described in 6 interventions (9.8%). A smaller number of interventions used semi-random prompts (*n* = 4, 6.6%) or user-indicated moments (*n* = 1, 1.6%). In 2 cases (3.3%), the decision point strategy was not reported. Notably, many interventions combined multiple strategies (e.g., daily real-time feedback supplemented by weekly scheduled summaries), suggesting that tailoring often relied on a hybrid structure rather than a single decision point type.

Most interventions (*n* = 45, 73.8%) used a knowledge-driven approach to define decision rules, relying on predefined logic, expert knowledge, or literature synthesis (Fig. 5b). Only 8 interventions (13.1%) employed a data-driven approach, such as machine learning algorithms, to guide tailoring decisions, and 1 intervention used a hybrid approach (*n* = 1, 1.6%). The adaptability of these decision rules was also limited in most cases: static rules (time-invariant and independent of prior behavior) were used in 45 interventions (73.8%) (Fig. 5c). Only 9 interventions (14.8%) implemented adaptive rules that could evolve over time based on previous user data or intervention outcomes. In both categories, 7 interventions (11.5%) did not report sufficient information.

All 61 interventions delivered their tailored support primarily via visual modalities, such as on-screen text or images. A smaller number of interventions included also auditory elements (*n* = 10, 16.4%, e.g., spoken feedback or sound signals), and only 3 interventions included haptic elements (4.9%, e.g., vibrations or tactile signals).

In terms of purpose, almost all intervention options were designed to provide feedback (*n* = 59, 96.7%) and suggestions (*n* = 55, 90.2%). Other common purposes included reinforcement (*n* = 47, 77.0%) and argumentation (*n* = 40, 65.6%). Reminders to perform healthy behavior were used less frequently (*n* = 15, 24.6%).

**Fig. 6:**
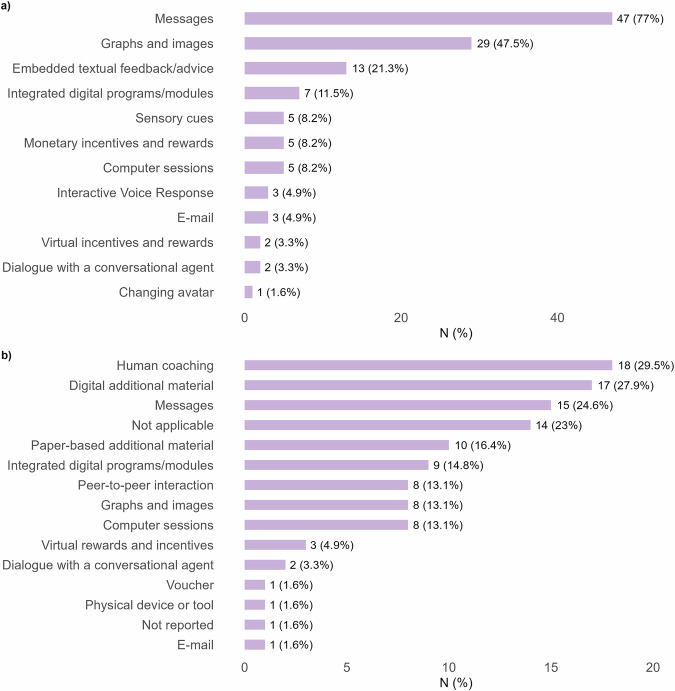
Types of dynamically and non-dynamically tailored intervention options in eHealth interventions. This multi-panel figure presents the range of intervention options used. Panel **a** shows dynamically tailored options, such as tailored messages, graphs or images, or embedded textual feedback within the intervention environment, etc. Panel **b** shows additional non-dynamically or not-computer tailored components. For example, these included human coaching, digital material, paper-based resources, or peer-to-peer interaction.

Several types of dynamically tailored intervention options were identified (Fig. 6a). The most common were messages (*n* = 47, 77.0%) and graphs or images (*n* = 29, 47.5%). Additionally, embedded textual feedback or advice was used in 13 interventions (21.3%), which refers to feedback or advice available within the intervention environment. In addition, integrated digital programs or modules appeared in 7 interventions (11.5%). These are short, structured components within the intervention that users actively work through, for instance a self-reflection exercise. Other types of dynamically tailored options were applied much less frequently (each *n* ≤ 5, ≤8.2%), such as sensory cues, computer sessions, monetary or virtual rewards, or dialogues with conversational agents. Several interventions also included non-dynamically or not-computer tailored components (Fig. 6b). These mostly included human coaching (*n* = 18, 29.5%), digital additional material (*n* = 17, 27.9%), paper-based resources (*n* = 10, 16.4%), and peer-to-peer interaction (*n* = 8, 13.1%).

### Way of delivery

Detailed information regarding the way of delivery, including specific modes, provider types, and forms of blended-care, can be found in Supplementary Data 3.

The computer-tailored modes of delivery of the included interventions varied widely. Most interventions were delivered via an app (*n* = 37, 60.7%), followed by text messaging (*n* = 22, 36.1%) and websites (*n* = 10, 16.4%). Other less frequently used modes included interactive voice response systems (*n* = 4, 6.6%), e-mail (*n* = 3, 4.9%), activity trackers (*n* = 2, 3.3%), automated phone calls (*n* = 1, 1.6%), software installed on a computer (*n* = 1, 1.6%), mobile web interfaces (*n* = 1, 1.6%), and web applications (*n* = 1, 1.6%). For one intervention, the mode of delivery was not reported.

Regarding the use of blended-care approaches, 22 interventions (36.1%) incorporated blended-care, whereas 39 interventions (63.9%) did not. Providers included primary care providers (*n* = 8, 13.1%), allied health professionals (*n* = 7, 11.5%, e.g., physiotherapists and dieticians), secondary care providers (*n* = 4, 6.6%), health coaches and counsellors (*n* = 4, 6.6%), and study-related facilitators (*n* = 3, 4.9%). Concerning the type of blended-care guidance, 12 interventions (19.7%) offered face-to-face support, while others (also) provided remote support (*n* = 11, 18.0%).

### Theoretical foundation and BCTs

A detailed summary of the development frameworks, behavior change theories, and BCT groups per intervention can be found in Supplementary Data 4.

In total, 15 different development frameworks or design principles were reported across the included interventions. The most frequently used approaches were *human-centered or user-centered design* (*n* = 6, 9.8%), *the planning model for tailored print materials* (*n* = 3, 4.9%), *intervention mapping* (*n* = 2, 3.3%), *conceptual model of JITAI components* (*n* = 2, 3.3%), and *the mHealth development and evaluation framework* (*n* = 2, 3.3%). However, most studies (*n* = 42, 68.9%) did not report any.

Regarding theoretical underpinnings, 43 out of 61 interventions (70.5%) explicitly reported the use of one or more behavior change theories or models, while 18 interventions (29.5%) did not mention any theory. In total, 42 different theories, theoretical principles, and models were identified. The most frequently applied theories included *Social Cognitive Theory* (*n* = 12, 19.7%), *Self-Regulation Theory* (*n* = 8, 13.1%), *Self-Determination Theory* (*n* = 6, 9.8%), the *Health Action Process Approach* (HAPA, *n* = 4, 6.6%), and the *Transtheoretical Model* (TTM, *n* = 4, 6.6%). Other reported models included the *Health Belief Model* (HBM, *n* = 3, 4.9%), *Cognitive Behavior Therapy* (CBT, *n* = 2, 3.3%), and *Motivational Interviewing* (*n* = 2, 3.3%). The majority of these theories are rooted in cognitive-behavioral or social-cognitive determinants and focus on constructs such as self-efficacy, behavioral intentions, and the role of contextual and environmental influences on health behaviors.

Across the 61 included interventions, a mean of 6.7 (SD 2.5) of the 16 BCT groups were reported per intervention. The most frequently applied BCT groups were *Feedback and monitoring* (*n* = 60, 98.4%), *Goals and planning* (*n* = 59, 96.7%), *Shaping knowledge* (*n* = 50, 82.0%), *Natural consequences* (*n* = 45, 73.8%), and *Reward and threat* (*n* = 44, 72.1%). In contrast, *Scheduled consequences* (*n* = 2, 3.3%), *Covert learning* (*n* = 5, 8.2%), *Identity* (*n* = 6, 9.8%), and *Comparison of behavior* (*n* = 7, 11.5%) were among the least reported BCT groups.

**Fig. 7:**
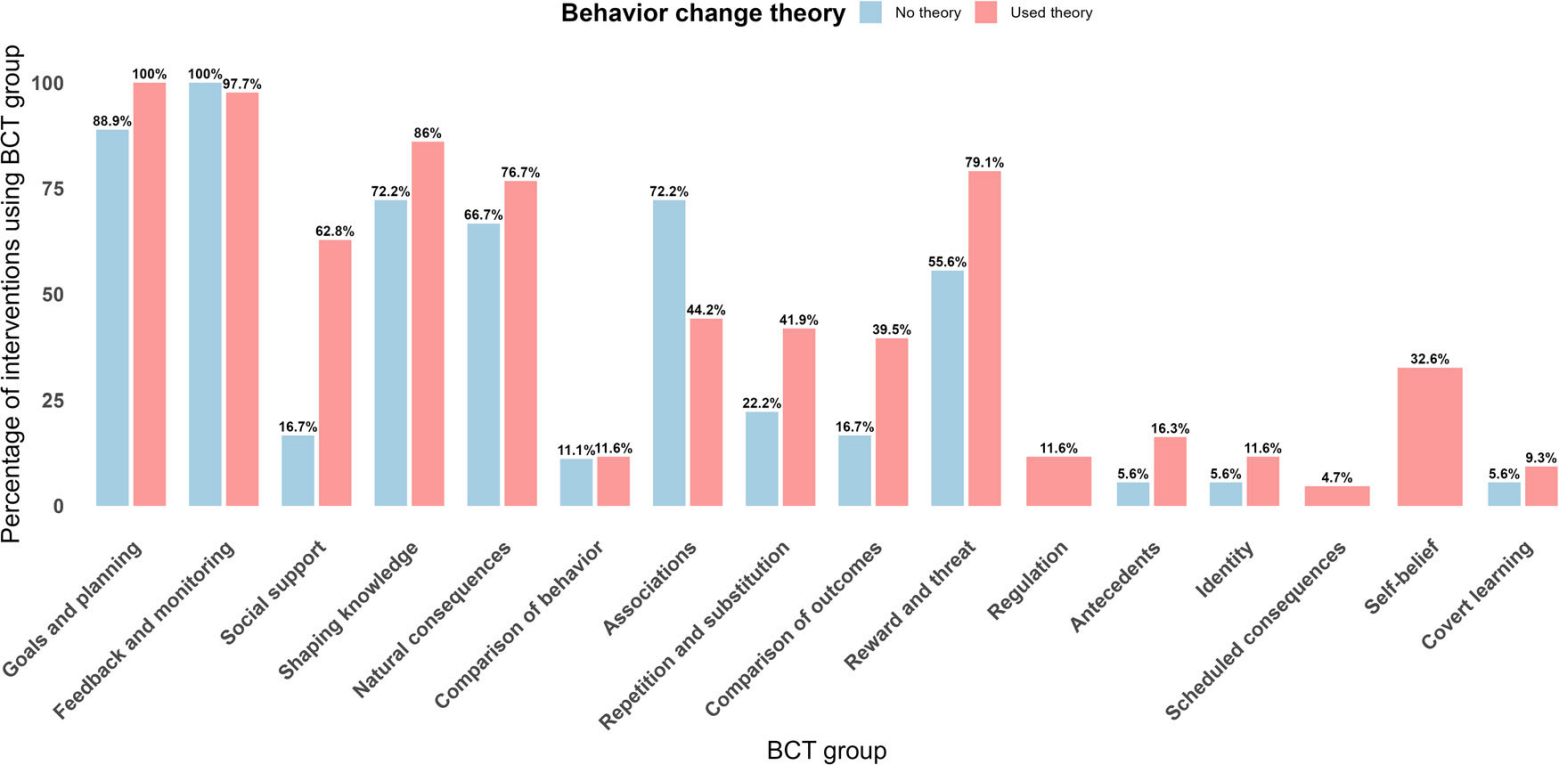
Behavior change technique (BCT) groups applied in dynamically tailored eHealth interventions, split by use of theory. This figure shows the distribution of BCT groups incorporated into dynamically tailored eHealth interventions. The results are presented separately for interventions that explicitly reported the use of a theoretical model or principles and those that did not. The BCTs are displayed at the group level, following the taxonomy by Michie et al.^51^.

As illustrated in Fig. 7, the application of BCT groups differed between interventions that used a behavior change theory (*n* = 43, 70.5%) and those that did not (*n* = 18, 29.5%). Interventions that used behavior change theory reported a mean of 7.3 (SD 2.4) BCT groups, while interventions that did not use theory applied a mean of 5.4 (SD 2.1) BCT groups. Several BCT groups, such as *Goals and planning* and *Feedback and monitoring*, were highly prevalent in both groups, suggesting these techniques are commonly applied regardless of theoretical underpinning. However, interventions that were theory-based more frequently included BCT groups such as *Reward and threat* (79.1% vs. 55.6%) and *Social support* (62.8% vs. 16.7%). Moreover, theory-based interventions made broader use of less common BCT groups such as *Self-belief* (32.6% vs. 0%) and *Regulation* (11.6% vs. 0%), indicating a more diverse and potentially more targeted application of techniques.

### Evaluation outcomes

Outcomes were reported for 44 of the 61 included interventions (72.1%) and 26 interventions (42.6%) were evaluated in a study that included a control group. Supplementary Data 5 provides details on study methods, including eligibility criteria, sample sizes, and intervention and control conditions. Supplementary Data 6 summarizes the key findings per study. Most frequently, studies reported on user experiences and usability (*n* = 35, 57.4%), behavioral effects such as physical activity or dietary changes (*n* = 29, 47.5%), and use, adherence, or engagement metrics (*n* = 29, 47.5%). Fewer studies addressed effects on clinical outcomes (*n* = 18, 29.5%), weight (*n* = 16, 26.2%), and quality of life (*n* = 5, 8.2%).

Overall, user experiences with the interventions were positive, with users valuing personalized and supportive text messages^52–55^. These text messages should be short, clear and positively framed as overly complex or repetitive content reduced the participants satisfaction^56–58^. Users also highlighted the need for greater personalization, flexible frequency, and better timing^59–61^. At the same time, technical challenges such as syncing issues, short battery life, poor connectivity, and confusing navigation often hindered satisfaction and effective use^62–64^.

Table 1 presents a summary of the reported within- and between-group differences in outcome measures among the included studies. The most frequently reported outcome domain was physical activity, which was evaluated in 24 studies (54.5%). Within-group differences were most often significant for effects on weight, including waist circumference (*n* = 7, 87.5%), body mass index (*n* = 6, 66.7%), and weight (*n* = 6, 60.0%). Between-group differences were most frequently significant for physical activity (*n* = 9, 37.5%) and glucose regulation (*n* = 3, 42.9%). However, many studies did not report statistical comparisons between groups. Several outcomes, particularly related to clinical indicators such as blood pressure and physical capacity, were more often found to be non-significant or not reported.

**Table 1:** Reported within- and between-group effects on outcome measures

		Within-group difference			Between-group difference		
Outcome	Studies (*N*)	Significant (*N* (%))	Not significant (*N* (%))	Not reported (*N* (%))	Significant (*N* (%))	Not significant (*N* (%))	Not reported (*N* (%))
Effects on quality of life	5	2 (40.0)	1 (20.0)	2 (40.0)	1 (20.0)	3 (60.0)	1 (20.0)
Effects on health behavior							
Physical activity	24	11 (45.8)	5 (20.8)	8 (33.3)	9 (37.5)	6 (25.0)	9 (37.5)
Sedentary behavior	4	3 (75.0)	0 (0.0)	1 (25.0)	1 (25.0)	0 (0.0)	3 (75.0)
Nutrition	11	5 (45.5)	0 (0.0)	6 (54.5)	2 (18.2)	6 (54.5)	3 (27.3)
Effects on weight							
Weight	10	6 (60.0)	1 (10.0)	3 (30.0)	2 (20.0)	6 (60.0)	2 (20.0)
Body Mass Index	9	6 (66.7)	3 (33.3)	0 (0.0)	1 (11.1)	5 (55.6)	3 (33.3)
Waist circumference	8	7 (87.5)	0 (0.0)	1 (12.5)	2 (25.0)	5 (62.5)	1 (12.5)
Effects on clinical health outcomes							
Physical capacity	4	2 (50.0)	1 (25.0)	1 (25.0)	0 (0.0)	3 (75.0)	1 (25.0)
Blood pressure	7	1 (14.3)	3 (42.9)	3 (42.9)	1 (14.3)	5 (71.4)	1 (14.3)
Cholesterol	4	2 (50.0)	1 (25.0)	1 (25.0)	2 (50.0)	1 (25.0)	1 (25.0)
Glucose regulation	7	4 (57.1)	0 (0.0)	3 (42.9)	3 (42.9)	2 (28.6)	2 (28.6)

There was substantial variability in use, adherence, and engagement levels across studies. Several interventions demonstrated high usage and adherence rates, often exceeding 80–90%, while others reported moderate levels (40–60%) or even low engagement (Supplementary Data 6). A high variability in use and adherence was also reflected in large standard deviations and wide ranges of metrics within an intervention. Furthermore, a decline in intervention use over time was observed in multiple studies. For example, *RENATA* by Storm et al.^65^ reported a drop in session participation from 90.8% at baseline to only 19.9% by the final session. Similarly, *MyPlan 2.0* by Poppe et al.^66^ observed a reduction in time spent per session as the intervention progressed.

### Quality of the intervention descriptions

**Fig. 8:**
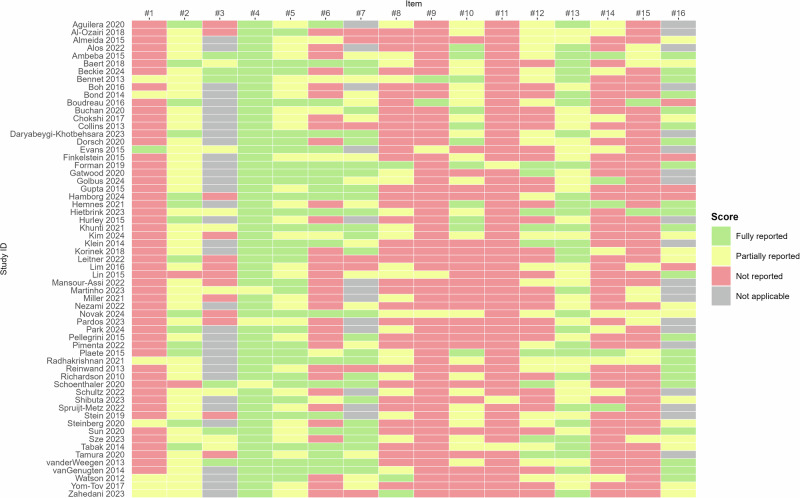
Quality assessment of the included interventions, assessed with the mERA checklist^76^. This figure summarizes the reporting quality ratings based on the mHealth Evidence Reporting and Assessment (mERA). Items (see ref. ^108^ for full descriptions): 1. Infrastructure (population level). 2. Technology platform. 3. Interoperability. 4. Intervention delivery. 5. Intervention content. 6. Usability content/testing. 7. User feedback. 8. Access of individual participants. 9. Cost assessment. 10. Adoption inputs. 11. Limitations for delivery at scale. 12. Contextual adaptability. 13. Replicability. 14. Data security. 15. Compliance with national guidelines or regulatory statutes. 16. Fidelity of the intervention.

Figure 8 provides a detailed overview of the quality assessment for each included intervention. Among the 16 mHealth Evidence Reporting and Assessment (mERA) items, reporting was most complete for *intervention delivery* (item #4), with 58 out of 61 interventions (95.1%) fully reporting on this aspect. This was followed by *intervention content* (item #5), reported in full by 31 interventions (50.8%), and *replicability* (item #13), fully reported by 30 interventions (49.2%). All other interventions provided at least partial reporting on these three items. In contrast, the least reported item was *limitations for delivery at scale* (item #11), for which 58 out of 61 interventions (95.1%) provided no relevant information. This was followed by limited reporting on *cost assessment* (item #9), with 56 interventions (91.8%) lacking information, and *infrastructure* (item #1), not reported in 53 interventions (86.9%).

Among the included interventions, *MyPlan 2.0* by Plaete et al.^59^ and Poppe et al.^66–69^ showed the most comprehensive reporting, with 9 out of 16 mERA items fully addressed and 3 items partially addressed. Similarly, *OnTrack/DietAlert* by Forman et al.^43,70^ and Goldstein et al.^71–74^ also demonstrated high reporting quality, with 9 items fully reported and 2 items partially reported.

## Discussion

This review highlights the current state of dynamically tailored eHealth interventions for lifestyle support among individuals with chronic diseases. In line with prior reviews^45,48,50^, we found a highly heterogeneous field with considerable variation in study design, intervention targets, tailoring strategies, and delivery modes. This heterogeneity reflects the exploratory character of the field but also raises challenges for drawing firm conclusions on effectiveness or best practices.

This review demonstrates that tailoring strategies differed considerably in both the number and type of variables used. On average, interventions incorporated variables from approximately two categories, with a range from one to as many as seven. As most interventions targeted physical activity and nutrition, it is unsurprising that lifestyle-related parameters dominated. In total, 17 interventions relied exclusively on physical activity and/or nutrition variables without considering additional factors. Nevertheless, all other interventions integrated psychosocial, contextual, or physiological parameters, indicating growing recognition that behavior change in chronic disease management is shaped by a broader set of influences than lifestyle behavior alone^75,76^. The considerable variation in tailoring variables highlights the need for systematic consideration of which behavioral, psychosocial, contextual, and physiological factors should be prioritized in dynamically tailored interventions. Future research should aim to identify the most influential variables for behavior change in chronic disease management, assess how multiple variables interact, and develop guidelines to support evidence-based selection of tailoring variables.

A clear divide emerged between physical activity and dietary measurements. For physical activity, about half of the interventions relied on objective devices, while the rest still used self-reports. Dietary intake was exclusively self-reported, reflecting the lack of scalable objective measures in nutritional science^77,78^. Both approaches illustrate an important trade-off: objective monitoring improves accuracy but may increase costs and technical complexity, whereas self-reports are more feasible but risk reduced reliability, especially when response demands are high^79^. Additionally, our review highlights that disease-specific measurements were incorporated only to a limited extent. For weight management in individuals with overweight or obesity, self-monitoring of body weight is relatively common and can be considered a form of biofeedback, directly linking lifestyle choices to physiological outcomes^51^. However, in conditions such as diabetes, hypertension, or COPD, the use of devices like glucose meters or blood pressure monitors was far less frequent. Expanding such monitoring could make the relevance of behavior change more tangible and motivating by providing direct feedback on health outcomes that matter to patients^80,81^. This gap highlights a possible future direction: interventions could also consider outcomes that patients themselves find meaningful in relation to their disease management.

Furthermore, we found that knowledge-driven rules remained dominant in decision-making processes used to translate data into support, confirming earlier reviews^46,48,50^. More advanced, data-driven approaches such as machine learning were rare but are beginning to emerge. Given the rapid advances in artificial intelligence, a shift towards more automated, adaptive tailoring is expected in the coming years^82,83^. Such approaches could enable more nuanced personalization by detecting patterns across large datasets and adjusting support dynamically^84^. However, empirical evidence demonstrating their added value over rule-based methods is still scarce and further research is needed to understand whether these approaches indeed improve tailoring and outcomes in practice.

Intervention delivery modes were equally diverse. However, most dynamic intervention components relied on text-based formats, whereas modalities such as videos, conversational agents, or gamified features were rarely applied. While eHealth is often praised for its scalability and potential to reduce barriers to care^85^, our review shows that current tailoring strategies may inadvertently increase health disparities. While text messages are scalable and low-cost, they may unintentionally exclude individuals with lower literacy or cognitive challenges. This raises concerns about inclusivity and equity: populations who may benefit from eHealth interventions are at risk of being left behind. Richer modalities such as voice-based interaction, adaptive visuals, or gamification could address these gaps and enhance sustained engagement^86,87^. To advance the field, researchers should systematically investigate how these richer modalities can be integrated into dynamically tailored interventions and consider inclusive design principles from the outset to ensure that interventions are accessible and equitable for all users.

Most interventions in this review were theory-driven and incorporated multiple BCT groups^51^, but theoretical underpinnings varied considerably. Over 70% of interventions were explicitly linked to theory, yet 42 different theories and principles were used. This diversity reflects the absence of consensus on which models best explain mechanisms of change in dynamically tailored eHealth. Although heterogeneity is not inherently problematic, since the optimal theoretical foundation depends on the intervention goals, it does create challenges for cumulative knowledge building. Without clearer guidance, developers risk “reinventing the wheel”. Greater clarity is therefore needed regarding which theoretical approaches and BCTs are most valuable for particular intervention objectives. Importantly, in dynamically tailored interventions, theory plays a crucial role in determining when, how, and for whom support should adapt, because it provides a structured understanding of the mechanisms that drive behavior change and helps identify which variables are meaningful for tailoring^88^. Tools which systematically link mechanisms of action to effective BCTs (e.g., the Theory and Technique Tool^89^), offer a concrete way forward by enabling researchers to make evidence-based selections and translate these into intervention components. More systematic use of such resources could accelerate progress and improve the coherence of future interventions.

Evaluation outcomes reflected the heterogeneity in intervention designs. Although many studies reported within-group improvements, between-group effects were less frequently reported and more often statistically non-significant. Notably, around a quarter of the included interventions had not yet published outcome data, including several employing more advanced tailoring approaches^90–92^. Therefore, the added value of dynamically tailored eHealth interventions over usual care or existing programs remains uncertain based on this review. Previous reviews have also reported mixed findings: while some studies demonstrate substantial improvements in health behaviors and clinical outcomes^93–95^, others show modest or inconsistent effects^96–98^. Moreover, many interventions reported a decline in user adherence over time^65,66,69,99^, which is a crucial mediator of intervention success. In addition, user experiences provide important context to interpret these outcomes. Most studies reported positive perceptions of personalized support, while technical issues and overly complex or repetitive content reduced satisfaction. However, these experiences were typically evaluated at the level of the intervention as a whole, rather than disentangling the contribution of dynamically tailored elements. This makes it difficult to determine to what extent user experiences reflects the operationalization of dynamic tailoring. Therefore, we encourage researchers to systematically examine how different tailoring strategies are experienced and which elements support or hinder long-term engagement^100^.

While our review does not yet allow us to identify best practices in dynamic tailoring, it does provide important insights into the methodological and conceptual foundations that need to be strengthened before such best practices can emerge.

This review shows that developing dynamically tailored eHealth interventions is inherently complex. The heterogeneity we observed highlights how interdependent design choices are: the selection of tailoring variables is tied to how these can be measured, which in turn influences the frequency of decision points, and whether rule-based decision rules are sufficient or whether more advanced algorithmic approaches are required. Similarly, intervention outputs must be carefully considered, not only in terms of content but also delivery modality, and should be consistent with the underlying theoretical framework. These interconnections mean that developers face trade-offs between accuracy, feasibility, user burden, and inclusivity at every step^100^. This multifaceted decision-making process underscores the importance of multidisciplinary collaboration, combining expertise from medical, behavioral, data, and design perspectives to create dynamic interventions. To address this complexity in practice, developers should make design trade-offs explicit and document the rationale behind their choices.

However, our findings revealed that many interventions still function as black boxes. In fact, nearly 20% of otherwise eligible studies could not be included due to insufficient reporting of tailoring procedures or theoretical foundations, and even among included studies, algorithms and theoretical justifications were frequently missing. This lack of transparency hampers replication and prevents cumulative knowledge building. This problem is not new: previous reviews have raised similar concerns^45,46,48,49^. Yet, our findings once again highlight the urgent need for greater uniformity in reporting. The Behavior Change Intervention Ontology (BCIO)^101,102^ and the accompanying Paper Authoring Tool^103^ offer a concrete way forward. By providing a structured framework for describing the theoretical justifications, tailoring strategies, and delivery and implementation features of interventions, BCIO can help overcome current heterogeneity and foster comparability across studies. If researchers consistently report and label interventions according to BCIO, this would not only facilitate more robust evidence synthesis but also open the door to approaches that can identify patterns across large datasets and accelerate the discovery of effective intervention components^104^.

Lastly, research is needed that isolates the contribution of specific components and generates cumulative evidence on what works, for whom, and under which circumstances. Our review highlights that progress towards identifying effective components and best practices is hindered by the way interventions are currently evaluated. We found that traditional RCTs continue to dominate but are poorly suited to capture the nuanced effects of multi-component interventions. Only a handful of papers attempt to compare intervention components directly, but those that will, are often still in the protocol stage^64,74,92,105^. Innovative designs such as factorial trials, micro-randomized trials, and SMART designs are better positioned to isolate the effects of specific tailoring strategies and generate cumulative evidence^27^. In parallel, many studies now incorporate process-oriented outcomes such as user engagement and acceptability, which are critical for understanding the working mechanisms of eHealth interventions^106,107^. Taken together, evaluations should extend beyond clinical endpoints to systematically integrate user experience, cost-effectiveness, and scalability, because these are all factors that ultimately determine whether interventions can be implemented successfully in real-world settings^100,108^.

This review offers a comprehensive and multidisciplinary synthesis of dynamically tailored eHealth interventions for promoting healthy lifestyles in people with chronic conditions. By systematically mapping tailoring strategies, theoretical underpinnings, delivery modalities, and evaluation outcomes, it provides new insights into how dynamic tailoring is currently conceptualized and operationalized. In doing so, it contributes to a more nuanced understanding of the state of the science, highlighting not only current practices but also critical points for improvement.

A limitation of this review lies is that many of the included studies were limitedly reported. In particular, dynamically tailored components were often poorly described, making it difficult to determine whether certain interventions met the inclusion criteria or to fully identify their core components. As a result, relevant studies may have been unintentionally excluded, and some BCTs or features may have remained undetected due to insufficient detail. For example, BCTs often had to be inferred from screenshots or message examples rather than from explicit labels. These limitations in reporting constrained the precision of data extraction and synthesis. To mitigate this, a second reviewer verified a subset of the extracted data and uncertainties were discussed in detail, supporting the reliability of the findings. Despite these challenges, inter-rater agreement during screening was high, indicating a consistent and careful selection process.

In conclusion, this systematic review provides a comprehensive overview of the current heterogeneous landscape of dynamically tailored eHealth interventions for lifestyle support in people with chronic diseases. While physical activity and nutrition dominated as tailoring parameters given the behavioral focus of most interventions, nearly three quarters also integrated contextual, emotional, or physiological variables. Measurement approaches varied, with physical activity mostly captured by objective devices, whereas dietary intake remained exclusively self-reported. Biofeedback through disease-specific monitoring was rarely applied. Furthermore, tailoring decisions were largely knowledge-driven, intervention options predominantly text-based, and theoretical foundations diverse. Although many studies reported positive effects on behavior, weight, and related outcomes, the added value of dynamic tailoring over standard care or static approaches remains inconclusive. Collectively, these findings illustrate both the complexity and the promise of dynamically tailored eHealth interventions, and the gradual movement of the field beyond one-size-fits-all approaches. To advance the field, we must move towards identifying effective elements and best practices. This requires some concrete steps. First, developers should make design trade-offs explicit, balancing feasibility, inclusivity, accuracy, and user burden. Second, researchers must commit to transparent reporting of intervention components, ideally using structured frameworks such as the BCIO. Third, evaluation methods need to evolve towards innovative designs that can disentangle effective components. In conclusion, dynamically tailored eHealth interventions are steadily moving towards greater sophistication. With continued efforts, the field is well-positioned to deliver more tailored and impactful lifestyle support for people with chronic diseases.

## Methods

### Study design

This systematic review adhered to the Preferred Reporting Items for Systematic Reviews and Meta-Analyses (PRISMA) checklist^109^ (Supplementary Table 1). The protocol was registered on the international Prospective Register of Systematic Reviews (PROSPERO) under registration number 387396.

### Study designs

We included studies of any design (e.g., qualitative and quantitative studies reporting findings from intervention design and development, feasibility studies, pilot studies or summative evaluation studies), provided the study contains a description of the intervention. Conceptual and purely methodological papers that do not present primary data or findings, such as studies without a description of the developed or evaluated intervention, were excluded because we were mainly interested in summarizing the application of dynamic tailoring in existing eHealth interventions. Systematic reviews and meta-analyses of eHealth interventions were excluded, as the focus was on the application of dynamic tailoring in existing eHealth interventions. The reference lists of four relevant systematic reviews on this topic were checked to ensure that key relevant studies were captured^45,48–50^.

### Participants

We examined studies focusing on adults with key risk factors for lifestyle-related diseases or specific diseases, including chronic obstructive pulmonary disease (COPD), lifestyle-related cardiovascular disease, and conditions related to metabolic syndrome, such as type 2 diabetes mellitus, hypercholesterolemia, hypertension, overweight, and obesity. The reason for examining these risk factors and diseases is that an unhealthy lifestyle plays a pivotal role in their development, and lifestyle modifications can positively impact their progression^3,9,110^. The selection of specific chronic diseases was determined collaboratively by our research team, which included clinicians, behavioral scientists, and an information specialist. Together, we identified relevant diseases and corresponding search terms to ensure comprehensive coverage of the selected lifestyle-related diseases. Studies focusing on cardiovascular diseases that require acute treatment and often have immediate residual damage (e.g., cerebrovascular accident or myocardial infarction) were excluded, as these diseases often have a major impact on a person’s daily functioning and often require a rehabilitation process. Furthermore, studies with children (<18 years) and pregnant women as participants were excluded.

### Interventions

Studies were included if (1) the study describes the development and/or evaluation of a dynamically computer-tailored eHealth intervention and (2) the intervention aims to support healthy physical activity behavior, sedentary behavior, or a healthy diet in daily life.

We applied a broad definition of a computer-tailored dynamically tailored intervention, given the wide range of terminology that is used. We did not rely on the terminology used to describe the intervention during screening. Instead, we decided on inclusion based on the intervention description that was screened against the following criterium: A dynamically tailored intervention was defined as an intervention in which the content, amount, or timing of the behavioral support is tailored based on input obtained through repeated assessments of intervention variables over time^39^. Hardeman et al.^45^ described a total of three intervention features to distinguish different types of tailored interventions: (1) the intervention corresponds directly to a need for real-time support or an opportunity to act positively in line with their health goals (2) the content or timing of behavioral support is adapted or tailored according to input (data) collected by the system since the support was initiated, and (3) behavioral support is triggered by the system and not directly by users themselves. According to our definition of a dynamically tailored intervention, in order to be included, interventions should contain feature 2, and may contain the features 1 and/or 3. The coaching strategy should be computer-tailored and delivered by an eHealth technology (e.g., text-messaging, website, apps, sensors/wearables). Static tailored interventions where all feedback is based on a single baseline assessment were excluded.

The intervention had to aim for health behavior change regarding healthy physical activity behavior, sedentary behavior, or adherence to a healthy diet in daily life^111–115^. Rehabilitation programs were excluded because these interventions aim at restoring body function and condition by means of daily exercises and not specifically at lifestyle behavior change in daily life. Interventions that target multiple health behaviors were included, provided that information about physical activity or diet components could be extracted.

### Comparison

Due to the descriptive focus of the review, interventions with or without a comparator were included.

### Outcomes

Included studies could present one of the following qualitative or quantitative outcomes: usability (e.g., efficiency, learnability), feasibility (e.g., demand, integration)^116^, adherence, user engagement or intervention effectiveness (e.g., increased physical activity, improved health outcomes).

### Setting

Both stand-alone and blended-care interventions in any setting were included.

### Timing

There was no restriction regarding the length of follow-up of outcomes due to the descriptive nature of the review.

### Language

We included papers reported in the English language.

### Type of publication

We included journal and conference papers in the review. Any other type of publication such as reviews, conference abstracts, book chapters, conference proceedings, editorials, PhD theses, and master’s theses were excluded.

### Search strategy

We searched the electronic databases PubMed, Scopus, Web of Science, PsycINFO, and ACM Digital Library for studies published between January 2000 and June 2024. No study design limits were imposed on the primary search. Since the application of computer-tailored interventions is described from the 2000s onwards^117^, the literature search was limited to studies published in or after 2000. Literature search strategies were developed using Medical Subject Headings (MeSH), word stems and text words related to the included target populations, eHealth, dynamic tailoring, physical activity, and nutrition. Search terms were searched for in titles, abstracts and keywords and were adapted for each database. The research team piloted the search terms. An expert in the field of human computer interaction and academic librarians in the field of computer science and behavioral sciences were consulted to refine the search terms. Initial searches took place in March 2023. Due to the time required to screen the high number of full-text papers, an updated search was conducted in June 2024 to include the most recent literature in the review.

The precise search strategy is included in Supplementary Tables 2–6. The electronic database search was supplemented by additional papers of interest identified by the research team.

### Study records

Papers identified via the electronic literature search and by the research team were merged with EndNote (Clarivate Analytics, USA) and duplicate records were removed. To support the time-consuming screening process, an active machine learning software platform, ASReview (Utrecht University, The Netherlands), was used to screen titles and abstracts. ASReview applies a researcher-in-the-loop text mining algorithm. This algorithm continuously provides the next record with the highest probability of being relevant, based on previous decisions by the reviewer. Full-text screening, data extraction and the quality appraisal was performed in Covidence (Veritas Health Innovation, Australia). The electronic data extraction form was prepared and managed in Covidence. Data synthesis was performed in R Studio version 2023.06.0.

### Selection process

For the title and abstract screening, 3 relevant and 3 irrelevant papers were added to ASReview as prior knowledge to train the algorithm, as the heterogeneity in papers was expected to be high. One reviewer (E.A.G.H.) screened titles and abstracts against the pre-specified eligibility criteria supported by ASReview. The stopping criterion was set to cease the screening process if we identified 150 consecutive studies as irrelevant. However, the stopping criterion was not met, indicating that all titles and abstracts underwent screening. Two second reviewers (A.M. or M.T.) screened a random 20% of the first reviewer’s decisions to assess the reliability. If the relevance of a study was unclear from the abstract, then the record was marked as relevant to assess relevancy in the full-text stage. A first reviewer (E.A.G.H.) and three second reviewers (C.L., A.M., M.T.) independently screened all full texts. Reasons for exclusion were recorded at the full text screening stage in line with the PRISMA guidelines^109^. Discrepancies were resolved through discussion and by consulting a third author if necessary.

We determined the IRA for the title-abstract screening and full-text screening, and Cohen’s Kappa for the full-text screening phase. The IRA was calculated based on the agreement between the first reviewer (E.A.G.H.) and the second reviewer (C.L., A.M., or M.T.), by dividing the number of papers on which they agreed (either inclusion or exclusion) by the total number of double-screened papers^45^.

### Data extraction process

Data were extracted into a standardized data extraction form developed in Covidence. The data extraction form can be found in Supplementary Table 7. Studies that described the same eHealth intervention (i.e., same name or description) and had overlapping authors between studies were merged into one record. Data were extracted from each eligible study by one reviewer (E.A.G.H.) and a random 20% was verified by another reviewer (C.L., A.M., M.T.). Discrepancies were resolved through discussion and by consulting another reviewer if required. In the papers that were not double-checked, uncertainties and questions were discussed with the other reviewers.

### Data items

The data extraction form was informed by various existing templates, frameworks and taxonomies to answer the research questions. The data extraction form consisted of several sections, including (1) study eligibility (e.g., study design, PICO items), (2) general study and intervention characteristics, (3) dynamic tailoring features, (4) development framework and theoretical basis, (5) way of delivery, (6) evaluation methods and results (if applicable). The sections regarding general study characteristics, study eligibility, evaluation methods and evaluations results was based on the Cochrane template “Data collection form for intervention reviews: RCTs and non-RCTs”^118^. We used the conceptual model of JITAI components by Nahum-Shani^38^ to design the sections regarding dynamic tailoring features. To extract data regarding development framework and theoretical basis, we based the form on the Behavior Change Technique Taxonomy Version 1 (BCTTv1)^51^.

### Outcomes and prioritization

**Fig. 9:**
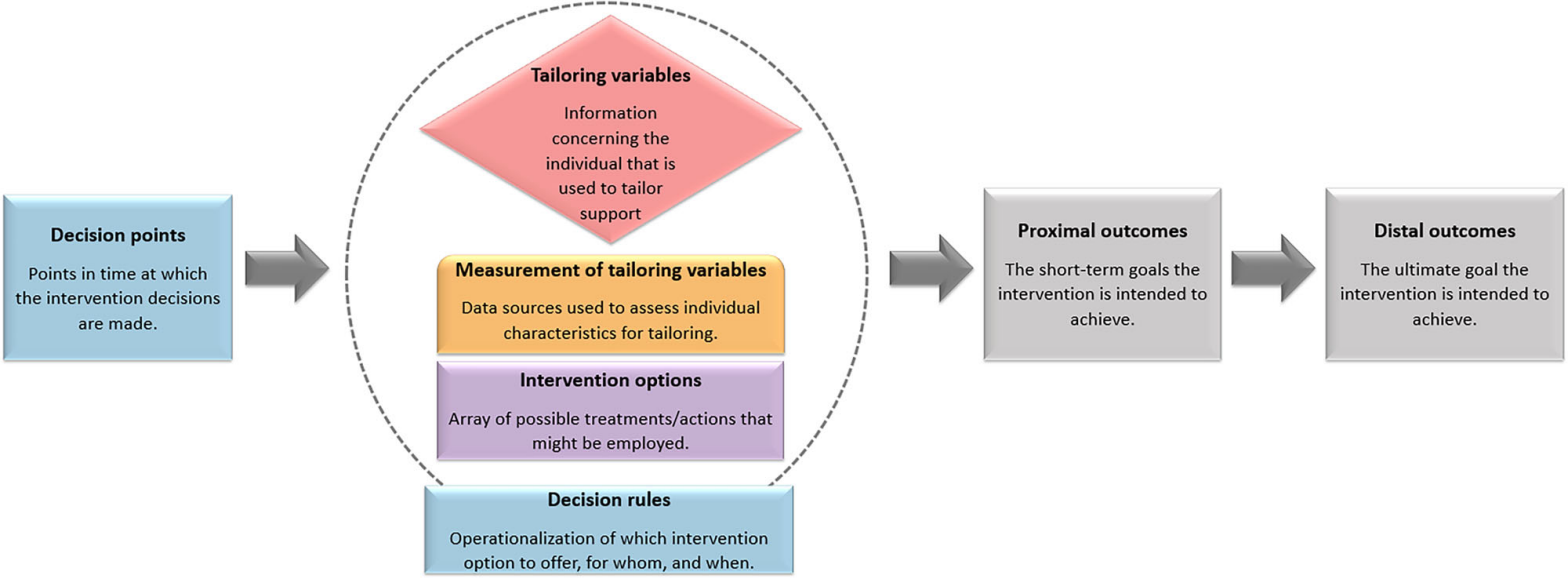
Adapted version of conceptual model of JITAI components by Nahum-Shani^38^ used to describe tailoring strategies. This figure illustrates the adapted conceptual model of JITAI components, based on Nahum-Shani et al.^38^. The model includes several core elements: distal outcomes (long-term goals), proximal outcomes (short-term behavioral or clinical targets), tailoring variables (parameters used for tailoring), measurement methods of tailoring variables, intervention options (array of possible treatments/actions), decision rules (linking tailoring variables to intervention options), and decision points (points in time at which an intervention decision must be made).

How the key components of a dynamically tailored intervention for promoting physical activity and nutrition were operationalized was of primary interest. The dynamic tailored intervention features were described based on the conceptual model of JITAI components by Nahum-Shani (Fig. 9)^38^. The exact elements extracted regarding the primary outcome included (1) type of dynamic tailored intervention (e.g., dynamically tailored or JITAI), (2) goal setting options, (3) tailoring variables, (4) sources of data used to trigger support, (5) types of monitor devices used, (6) type of decision points, (7) type of decision rules, (8) modes of delivery of intervention options, (9) purposes of intervention options, and (10) types of intervention options.

Secondary interests included the general study and intervention characteristics, the development framework and theoretical basis, the way of delivery, and evaluation results. The general study and intervention characteristics included the name of the intervention, the aim of the study, study design, target behaviors, and target populations. The development framework and theoretical basis comprised the framework or model used to develop the intervention, behavior change theory and the BCT groups applied in the intervention. Regarding the way of delivery, the extracted elements included the delivery platform and whether the intervention is delivered in combination with regular care (i.e., blended-care). The evaluation results included any reported outcome regarding the user experiences, usability, use, adherence, engagement, or effectiveness of the intervention. The following studies provide definitions of usability^119^, feasibility^116^, adherence^120^, and engagement^27^.

### Quality appraisal

The mERA checklist^108^ was used to assess the quality of the studies that report evaluation outcomes due to the heterogeneity in study designs and outcomes. This checklist is particularly suitable for this review because it provides a standardized quality assessment for eHealth intervention descriptions across various study designs, making it well-suited to the heterogeneity in study designs. The mERA checklist consists of 16 items focused on transparent reporting of the stages of development of mHealth interventions, technical specification criteria of the intervention, formative research or user testing, feasibility or effectiveness studies, and maturity of the intervention. In line with Perski et al.^46^, each checklist item was scored as ‘fully reported’, ‘partially reported’ or ‘not reported’. The quality appraisal was conducted by one reviewer (E.A.G.H.) and a second reviewer (C.L., A.M., M.T.) independently cross-checked a random subset of 20% of the included studies. We resolved disagreements through discussion or by consulting a third author.

### Data synthesis

A narrative synthesis of extracted data was conducted given the anticipated diversity in study designs of the included studies. Data was presented in text, figures and summarized in tables.

## Supplementary information

Supplementary information

Supplementary data1

Supplementary data2

Supplementary data3

Supplementary data4

Supplementary data5

Supplementary data6

## Data Availability

The datasets used and analyzed during the current study are not publicly available at this time because they are being used for additional analyses. However, they may be made available to researchers upon reasonable request to the corresponding author. The data will be made publicly available once these analyses are completed and published.

## Code availability

The underlying code for this study is not publicly available but may be made available to researchers upon reasonable request to the corresponding author.
