# Supplementary material for: Systematic review of dynamically tailored eHealth interventions targeting physical activity and healthy diet in chronic disease
Source: NPJ Digit Med. 2025 Nov 19;8:696. doi: 10.1038/s41746-025-02054-7 (PMC12630729; doi:10.1038/s41746-025-02054-7)
Supplement: Supplementary file 1 — Supplementary information [file 41746_2025_2054_MOESM1_ESM.pdf]

Supplementary Information

Supplementary Table 1. PRISMA checklist

Section and Topic	Item #	Checklist item	Location where item is reported
**TITLE**			
Title	1	Identify the report as a systematic review.	Page 1
**ABSTRACT**			
Abstract	2	See the PRISMA 2020 for Abstracts checklist.	Page 2
**INTRODUCTION**			
Rationale	3	Describe the rationale for the review in the context of existing knowledge.	Page 3+4
Objectives	4	Provide an explicit statement of the objective(s) or question(s) the review addresses.	Page 4
**METHODS**			
Eligibility criteria	5	Specify the inclusion and exclusion criteria for the review and how studies were grouped for the syntheses.	Page 20+21
Information sources	6	Specify all databases, registers, websites, organisations, reference lists and other sources searched or consulted to identify studies. Specify the date when each source was last searched or consulted.	Page 21
Search strategy	7	Present the full search strategies for all databases, registers and websites, including any filters and limits used.	Supplementary Information
Selection process	8	Specify the methods used to decide whether a study met the inclusion criteria of the review, including how many reviewers screened each record and each report retrieved, whether they worked independently, and if applicable, details of automation tools used in the process.	Page 22
Data collection process	9	Specify the methods used to collect data from reports, including how many reviewers collected data from each report, whether they worked independently, any processes for obtaining or confirming data from study investigators, and if applicable, details of automation tools used in the process.	Page 22
Data items	10a	List and define all outcomes for which data were sought. Specify whether all results that were compatible with each outcome domain in each study were sought (e.g. for all measures, time points, analyses), and if not, the methods used to decide which results to collect.	Page 22+23
	10b	List and define all other variables for which data were sought (e.g. participant and intervention characteristics, funding sources). Describe any assumptions made about any missing or unclear information.	Page 22+23
Study risk of bias assessment	11	Specify the methods used to assess risk of bias in the included studies, including details of the tool(s) used, how many reviewers assessed each study and whether they worked independently, and if applicable, details of automation tools used in the process.	Page 21
Effect measures	12	Specify for each outcome the effect measure(s) (e.g. risk ratio, mean difference) used in the synthesis or presentation of results.	Supplementary Information
Synthesis methods	13a	Describe the processes used to decide which studies were eligible for each synthesis (e.g. tabulating the study intervention characteristics and comparing against the planned groups for each synthesis (item #5)).	Page 19+20 and

Section and Topic	Item #	Checklist item	Location where item is reported
			Supplementary Information
	13b	Describe any methods required to prepare the data for presentation or synthesis, such as handling of missing summary statistics, or data conversions.	Page 22
	13c	Describe any methods used to tabulate or visually display results of individual studies and syntheses.	Page 23
	13d	Describe any methods used to synthesize results and provide a rationale for the choice(s). If meta-analysis was performed, describe the model(s), method(s) to identify the presence and extent of statistical heterogeneity, and software package(s) used.	Page 22+23
	13e	Describe any methods used to explore possible causes of heterogeneity among study results (e.g. subgroup analysis, meta-regression).	NA
	13f	Describe any sensitivity analyses conducted to assess robustness of the synthesized results.	NA
Reporting bias assessment	14	Describe any methods used to assess risk of bias due to missing results in a synthesis (arising from reporting biases).	Page 23-24
Certainty assessment	15	Describe any methods used to assess certainty (or confidence) in the body of evidence for an outcome.	NA
**RESULTS**			
Study selection	16a	Describe the results of the search and selection process, from the number of records identified in the search to the number of studies included in the review, ideally using a flow diagram.	Page 5
	16b	Cite studies that might appear to meet the inclusion criteria, but which were excluded, and explain why they were excluded.	Page 5
Study characteristics	17	Cite each included study and present its characteristics.	Supplementary Files
Risk of bias in studies	18	Present assessments of risk of bias for each included study.	Page 14+15
Results of individual studies	19	For all outcomes, present, for each study: (a) summary statistics for each group (where appropriate) and (b) an effect estimate and its precision (e.g. confidence/credible interval), ideally using structured tables or plots.	Supplementary Data
Results of syntheses	20a	For each synthesis, briefly summarise the characteristics and risk of bias among contributing studies.	Page 5-15
	20b	Present results of all statistical syntheses conducted. If meta-analysis was done, present for each the summary estimate and its precision (e.g. confidence/credible interval) and measures of statistical heterogeneity. If comparing groups, describe the direction of the effect.	Page 5-15
	20c	Present results of all investigations of possible causes of heterogeneity among study results.	Page 5-15
	20d	Present results of all sensitivity analyses conducted to assess the robustness of the synthesized results.	NA
Reporting biases	21	Present assessments of risk of bias due to missing results (arising from reporting biases) for each synthesis assessed.	Page 6-13
Certainty of evidence	22	Present assessments of certainty (or confidence) in the body of evidence for each outcome assessed.	NA

Section and Topic	Item #	Checklist item	Location where item is reported
**DISCUSSION**			
Discussion	23a	Provide a general interpretation of the results in the context of other evidence.	Page 16+17
	23b	Discuss any limitations of the evidence included in the review.	Page 18
	23c	Discuss any limitations of the review processes used.	Page 18
	23d	Discuss implications of the results for practice, policy, and future research.	Page 16-18
**OTHER INFORMATION**			
Registration and protocol	24a	Provide registration information for the review, including register name and registration number, or state that the review was not registered.	Page 20
	24b	Indicate where the review protocol can be accessed, or state that a protocol was not prepared.	Page 20
	24c	Describe and explain any amendments to information provided at registration or in the protocol.	NA
Support	25	Describe sources of financial or non-financial support for the review, and the role of the funders or sponsors in the review.	Page 25
Competing interests	26	Declare any competing interests of review authors.	Page 25
Availability of data, code and other materials	27	Report which of the following are publicly available and where they can be found: template data collection forms; data extracted from included studies; data used for all analyses; analytic code; any other materials used in the review.	Page 25

Supplementary Table 2. Electronic search strategy Web of Science

Advanced search:	Topic (TS) = Title, abstract, author keywords, and keywords plus
Publication date:	Since 1 January 2000
Sort on:	Date: newest first
Export:	Endnote Desktop<br>Author, Title, Source, Abstract
Remarks:	-

Topic	ID	Query
**Result**	**#6**	**#1 AND #2 AND #3 AND (#4 OR #5)**
Chronic conditions	#1	TS= (overweight OR obes* OR “morbid* obes*” OR adipos* OR corpulence OR "type 2 diabetes mellitus" OR T2DM OR “type 2 diabetes” OR T2D OR “diabetes mellitus type 2” OR “diabetes type 2” OR “type-II diabetes mellitus” OR “adult-onset diabetes” OR “adult onset diabetes” OR “maturity-onset diabetes” OR “maturity onset diabetes” OR “non-insulin-

		dependent diabetes" OR "noninsulin-dependent diabetes" OR "chronic obstructive pulmonary disease*" OR COPD OR "chronic obstructive lung disease*" OR COLD OR "chronic obstructive airway disease*" OR COAD OR "chronic airflow obstruction*" OR "cardiovascular disease*" OR CVD OR "coronary heart disease*" OR CHD OR "coronary artery disease*" OR CAD OR "coronary disease*" OR "coronary arterioscleros*" OR "coronary atheroscleros*" OR "ischemic heart disease*" OR "ischaemic heart disease*" OR IHD OR "myocardial ischemia*" OR "angina pectoris" OR "stable angina" OR "cardiac failure" OR "heart failure" OR "congestive heart failure" OR CHF OR "left ventricular failure" OR "right ventricular failure" OR "myocardial failure" OR "claudicatio intermittens" OR "peripheral arterial disease*" OR PAD OR "peripheral vascular occlusive disease*" OR PVOD OR hypertension OR "essential hypertension" OR hyperpiesis OR "high blood pressure" OR prediabetes OR "prediabetic state" OR "insulin resistance" OR "glucose intolerance" OR "glucose metabolism disorder*" OR "impaired glucose tolerance" OR "impaired fasting glucose" OR "impaired glucose regulation" OR "impaired insulin sensitivity" OR "non-diabetic hyperglycaemia" OR "metabolic syndrome" OR "dysmetabolic syndrome" OR "cardiometabolic syndrome" OR "cardiometabolic risk factor*" OR hypercholesterolemia OR "high cholesterol level*" OR "elevated cholesterol" OR hypertriglyceridemia OR "high triglyceride level*" OR "lipid disorder*" OR hyperlipidemia OR dyslipidemia OR "high low-density lipoprotein" OR "high LDL")
eHealth	#2	TS= (ehealth* OR "e-health*" OR "electronic health" OR mhealth* OR "m-health*" OR "mobile health" OR "mobile application*" OR "mobile intervention*" OR "smart phone*" OR smartphone* OR "computer application*" OR "computer-based*" OR "internet-based*" OR "web-based" OR "app-based" OR "technology-based" OR "technology-supported" OR "website intervention" OR telemedicine OR telehealth OR "behavio* change technology" OR "mobile behavio* change intervention*" OR "digital behavio* change intervention*" OR "digital behavio* intervention*" OR "digital health intervention*" OR "digital health coach*" OR "digital lifestyle intervention*" OR "digital lifestyle behavio* change intervention*" OR "health informatic*" OR "health technology" OR "health promotion technolog*" OR "health interactive technolog*" OR "persuasive technolog*" OR "persuasive health* technolog*")
Dynamic tailoring	#3	TS=("dynamic* tailor*" OR "just-in-time" OR JIT OR "just-in-time adaptive" OR JITAI OR "ecological momentary" OR EMI OR "real-time" OR "personali*" OR "tailor*" OR "computer tailor*" OR "context tailor*" OR "context aware" OR "context triggered" OR "sensor triggered" OR "sensing technolog*" OR interactive*)
Physical activity	#4	TS=("physical activit*" OR "physical fitness" OR "active life*" OR "active liv*" OR "activ* behavio*" OR exercise OR workout OR sport* OR "sedentary behavio*")
Healthy nutrition	#5	TS=(nutrition OR "nutrition* intake" OR diet OR "diet* intake" OR "food intake" OR "food consumption" OR "nourishing food" OR "healthy nutrition" OR "healthy diet*" OR "healthy eat*" OR "healthy food*" OR "eating behavio*" OR "diet* behavio*")

Supplementary Table 3. Electronic search strategy PubMed

Advanced search:	MeSH = Medical Subject Headings<br>tiab = Title and abstract
Publication date:	Since 1 January 2000
Sort on:	Publication date
Export:	Citation manager
Remarks:	-

Topic	ID	Query
**Result**	**#6**	**#1 AND #2 AND #3 AND (#4 OR #5)**
Chronic conditions	#1	(Overweight[Mesh] OR Obesity[Mesh] OR overweight [tiab] OR obes* [tiab] OR “morbid* obes*” [tiab] OR adipos* [tiab] OR corpulence [tiab] OR “Diabetes Mellitus, Type 2”[Mesh] OR "type 2 diabetes mellitus" [tiab] OR T2DM [tiab] OR “type 2 diabetes” [tiab] OR T2D [tiab] OR “diabetes mellitus type 2” [tiab] OR “diabetes type 2” [tiab] OR “type-II diabetes mellitus” [tiab] OR “adult-onset diabetes” [tiab] OR “adult onset diabetes” [tiab] OR “maturity-onset diabetes” [tiab] OR “maturity onset diabetes” [tiab] OR “non-insulin-dependent diabetes” [tiab] OR “noninsulin-dependent diabetes” [tiab] OR “Pulmonary Disease, Chronic Obstructive”[Mesh] OR “chronic obstructive pulmonary disease*” [tiab] OR COPD [tiab] OR “chronic obstructive lung disease*” [tiab] OR COLD [tiab] OR “chronic obstructive airway disease*” [tiab] OR COAD [tiab] OR “chronic airflow obstruction*” [tiab] OR “Cardiovascular Diseases”[Mesh] OR “Heart Failure”[Mesh] OR “cardiovascular disease*” [tiab] OR CVD [tiab] OR “coronary heart disease*” [tiab] OR CHD [tiab] OR “coronary artery disease*” [tiab] OR CAD [tiab] OR “coronary disease*” [tiab] OR “coronary arterioscleros*” [tiab] OR “coronary atheroscleros*” [tiab] OR “ischemic heart disease*” [tiab] OR “ischaemic heart disease*” [tiab] OR IHD [tiab] OR “myocardial ischemia*” [tiab] OR “angina pectoris” [tiab] OR “stable angina” [tiab] OR “cardiac failure” [tiab] OR “heart failure” [tiab] OR “congestive heart failure” [tiab] OR CHF [tiab] OR “left ventricular failure” [tiab] OR “right ventricular failure” [tiab] OR “myocardial failure” [tiab] OR “claudicatio intermittens” [tiab] OR “peripheral arterial disease*” [tiab] OR PAD [tiab] OR “peripheral vascular occlusive disease*” [tiab] OR PVOD [tiab] OR hypertension [tiab] OR “essential hypertension” [tiab] OR hyperpiesis [tiab] OR “high blood pressure” [tiab] OR “Insulin Resistance” [Mesh] OR “Glucose Metabolism Disorders” [Mesh] OR “Prediabetic State” [Mesh] OR “Metabolic Syndrome” [Mesh] OR “Cardiometabolic Risk Factors” [Mesh] OR Hypercholesterolemia [Mesh] OR Hypertriglyceridemia [Mesh] OR prediabetes [tiab] OR “prediabetic state” [tiab] OR “insulin resistance” [tiab] OR “glucose intolerance” [tiab] OR “glucose metabolism disorder*” [tiab] OR “impaired glucose tolerance” [tiab] OR “impaired fasting glucose” [tiab] OR “impaired glucose regulation” [tiab] OR “impaired insulin sensitivity” [tiab] OR “non-diabetic hyperglycaemia” [tiab] OR “metabolic syndrome” [tiab] OR “dysmetabolic syndrome” [tiab] OR “cardiometabolic syndrome” [tiab] OR “cardiometabolic risk

		factor*" [tiab] OR hypercholesterolemia [tiab] OR "high cholesterol level*" [tiab] OR "elevated cholesterol" [tiab] OR hypertriglyceridemia [tiab] OR "high triglyceride level*" [tiab] OR "lipid disorder*" [tiab] OR hyperlipidemia [tiab] OR dyslipidemia [tiab] OR "high low-density lipoprotein" [tiab] OR "high LDL" [tiab])
eHealth	#2	("Mobile Applications"[Mesh] OR Telemedicine[Mesh] OR ehealth*[tiab] OR "e-health*" [tiab] OR "electronic health" [tiab] OR mhealth* [tiab] OR "m-health*" [tiab] OR "mobile health" [tiab] OR "mobile application*" [tiab] OR "mobile intervention*" [tiab] OR "smart phone*" [tiab] OR smartphone* [tiab] OR "computer application*" [tiab] OR "computer-based*" [tiab] OR "internet-based*" [tiab] OR "web-based" [tiab] OR "app-based" [tiab] OR "technology-based" [tiab] OR "technology-supported" [tiab] OR "website intervention" [tiab] OR telemedicine [tiab] OR telehealth OR "behavior change technology" [tiab] OR "mobile behavior change intervention*" [tiab] OR "mobile behaviour change intervention*" [tiab] OR "digital behavior change intervention*" [tiab] OR "digital behaviour change intervention*" [tiab] OR "digital behavior intervention*" [tiab] OR "digital behaviour intervention*" [tiab] OR "digital health intervention*" [tiab] OR "digital health coach*" [tiab] OR "digital lifestyle intervention*" [tiab] OR "digital lifestyle behavior change intervention*" [tiab] OR "health informatic*" [tiab] OR "health technology" [tiab] OR "health promotion technolog*" [tiab] OR "health interactive technolog*" [tiab] OR "persuasive technolog*" [tiab] OR "persuasive health technolog*"[tiab])
Dynamic tailoring	#3	("dynamic* tailor*" [tiab] OR "just-in-time" [tiab] OR JIT [tiab] OR "just-in-time adaptive" [tiab] OR JITAI [tiab] OR "ecological momentary" [tiab] OR EMI [tiab] OR "real-time" [tiab] OR "personali*" [tiab] OR "tailor*" [tiab] OR "computer tailor*" [tiab] OR "context tailor*" [tiab] OR "context aware" [tiab] OR "context triggered" [tiab] OR "sensor triggered" [tiab] OR "sensing technolog*" [tiab] OR interactive* [tiab])
Physical activity	#4	("Exercise"[Mesh] OR "Sedentary Behavior"[Mesh] OR "physical activit*" [tiab] OR "physical fitness" [tiab] OR "active life*" [tiab] OR "active liv*" OR "activ* behavior" OR "activ* behaviour" OR exercise [tiab] OR workout [tiab] OR sport* [tiab] OR "sedentary behavior" [tiab] OR "sedentary behaviour" [tiab])
Healthy nutrition	#5	("Diet, Healthy"[Mesh] OR nutrition [tiab] OR "nutrition* intake" [tiab] OR diet [tiab] OR "diet* intake" [tiab] OR food [tiab] OR "food intake" [tiab] OR "food consumption" [tiab] OR "nourishing food" [tiab] OR "healthy nutrition" [tiab] OR "healthy diet*" [tiab] OR "healthy eat*" [tiab] OR "healthy food*" [tiab] OR "eating behavior" [tiab] OR "eating behaviour" [tiab] OR "diet* behavior" [tiab] OR "diet* behaviour" [tiab])

Supplementary Table 4. Electronic search strategy Scopus

Advanced search:	TITLE-ABS-KEY = Doc Title, Abstract, Keyword
Publication date:	Since 1 January 2000
Sort on:	Date (newest)
Export:	RIS format (EndNote)
	Citation information, Bibliographical information, Abstract & keywords

Remarks: -

Topic	ID	Query
**Result**	**#6**	**#1 AND #2 AND #3 AND (#4 OR #5)**
Chronic conditions	#1	TITLE-ABS-KEY (overweight OR obes* OR “morbid* obes*” OR adipos* OR corpulence OR "type 2 diabetes mellitus" OR T2DM OR “type 2 diabetes” OR T2D OR “diabetes mellitus type 2” OR “diabetes type 2” OR “type-II diabetes mellitus” OR “adult-onset diabetes” OR “adult onset diabetes” OR “maturity-onset diabetes” OR “maturity onset diabetes” OR “non-insulin-dependent diabetes” OR “noninsulin-dependent diabetes” OR “chronic obstructive pulmonary disease*” OR COPD OR “chronic obstructive lung disease*” OR COLD OR “chronic obstructive airway disease*” OR COAD OR “chronic airflow obstruction*” OR “cardiovascular disease*” OR CVD OR “coronary heart disease*” OR CHD OR “coronary artery disease*” OR CAD OR “coronary disease*” OR “coronary arterioscleros*” OR “coronary atheroscleros*” OR “ischemic heart disease*” OR “ischaemic heart disease*” OR IHD OR “myocardial ischemia*” OR “angina pectoris” OR “stable angina” OR “cardiac failure” OR “heart failure” OR “congestive heart failure” OR CHF OR “left ventricular failure” OR “right ventricular failure” OR “myocardial failure” OR “claudicatio intermittens” OR “peripheral arterial disease*” OR PAD OR “peripheral vascular occlusive disease*” OR PVOD OR hypertension OR “essential hypertension” OR hyperpiesis OR “high blood pressure” OR prediabetes OR “prediabetic state” OR “insulin resistance” OR “glucose intolerance” OR “glucose metabolism disorder*” OR “impaired glucose tolerance” OR “impaired fasting glucose” OR “impaired glucose regulation” OR “impaired insulin sensitivity” OR “non-diabetic hyperglycaemia” OR “metabolic syndrome” OR “dysmetabolic syndrome” OR “cardiometabolic syndrome” OR “cardiometabolic risk factor*” OR hypercholesterolemia OR “high cholesterol level*” OR “elevated cholesterol” OR hypertriglyceridemia OR “high triglyceride level*” OR “lipid disorder*” OR hyperlipidemia OR dyslipidemia OR “high low-density lipoprotein” OR “high LDL”)
eHealth	#2	TITLE-ABS-KEY (ehealth* OR “e-health*” OR “electronic health” OR mhealth* OR “m-health*” OR “mobile health” OR “mobile application*” OR “mobile intervention*” OR “smart phone*” OR smartphone* OR “computer application*” OR “computer-based*” OR “internet-based*” OR “web-based” OR “app-based” OR “technology-based” OR “technology-supported” OR “website intervention” OR telemedicine OR telehealth OR “behavio* change technology” OR “mobile behavio* change intervention*” OR “digital behavio* change intervention*” OR “digital behavio* intervention*” OR “digital health intervention*” OR “digital health coach*” OR “digital lifestyle intervention*” OR “digital lifestyle behavio* change intervention*” OR “health informatic*” OR “health technology” OR “health promotion technolog*” OR “health interactive technolog*” OR “persuasive technolog*” OR “persuasive health* technolog*”)
Dynamic tailoring	#3	TITLE-ABS-KEY (“dynamic* tailor*” OR “just-in-time” OR JIT OR “just-in-time adaptive” OR JITAI OR “ecological momentary” OR EMI OR “real-time” OR “personali*” OR “tailor*” OR “computer tailor*” OR “context tailor*” OR “context aware” OR “context triggered” OR “sensor triggered” OR “sensing technolog*” OR interactive*)

Physical activity	#4	TITLE-ABS-KEY (“physical activit*” OR “physical fitness” OR “active life*” OR “active liv*” OR “activ* behavio*” OR exercise OR workout OR sport* OR “sedentary behavio*”)
Healthy nutrition	#5	TITLE-ABS-KEY (nutrition OR “nutrition* intake” OR diet OR “diet* intake” OR “food intake” OR “food consumption” OR “nourishing food” OR “healthy nutrition” OR “healthy diet*” OR “healthy eat*” OR “healthy food*” OR “eating behavio*” OR “diet* behavio*”)

Supplementary Table 5. Electronic search strategy PsycINFO

Advanced search:	DE = Subjects [exact]<br>TI = Title<br>AB = Abstract
Publication date:	Since 1 January 2000
Sort on:	Date newest
Export:	Add all records to folder and export RIS-file of records to EndNote
Remarks:	-

Topic	ID	Query
**Result**	**#6**	**#1 AND #2 AND #3 AND (#4 OR #5)**
Chronic conditions	#1	DE ("Overweight" OR "Obesity" OR "Type 2 Diabetes" OR "Chronic Obstructive Pulmonary Disease" OR "Atherosclerosis" OR "Angina Pectoris" OR "Coronary Heart Disease" OR "Cardiovascular Disorders" OR "Arteriosclerosis" OR "Heart Disorders" OR "Hypertension" OR "Ischemia" OR “Metabolic Syndrome” OR “Metabolism Disorders” OR “Lipid Metabolism Disorders”) OR TI (overweight OR obes* OR “morbid* obes*” OR adipos* OR corpulence OR "type 2 diabetes mellitus" OR T2DM OR “type 2 diabetes” OR T2D OR “diabetes mellitus type 2” OR “diabetes type 2” OR “type-II diabetes mellitus” OR “adult-onset diabetes” OR “adult onset diabetes” OR “maturity-onset diabetes” OR “maturity onset diabetes” OR “non-insulin-dependent diabetes” OR “noninsulin-dependent diabetes” OR “chronic obstructive pulmonary disease*” OR COPD OR “chronic obstructive lung disease*” OR COLD OR “chronic obstructive airway disease*” OR COAD OR “chronic airflow obstruction*” OR “cardiovascular disease*” OR CVD OR “coronary heart disease*” OR CHD OR “coronary artery disease*” OR CAD OR “coronary disease*” OR “coronary arterioscleros*” OR “coronary atheroscleros*” OR “ischemic heart disease*” OR “ischaemic heart disease*” OR IHD OR “myocardial ischemia*” OR “angina pectoris” OR “stable angina” OR “cardiac failure” OR “heart failure” OR “congestive heart

		failure" OR CHF OR "left ventricular failure" OR "right ventricular failure" OR "myocardial failure" OR "claudicatio intermittens" OR "peripheral arterial disease*" OR PAD OR "peripheral vascular occlusive disease*" OR PVOD OR hypertension OR "essential hypertension" OR hyperpiesis OR "high blood pressure" OR prediabetes OR "prediabetic state" OR "insulin resistance" OR "glucose intolerance" OR "glucose metabolism disorder*" OR "impaired glucose tolerance" OR "impaired fasting glucose" OR "impaired glucose regulation" OR "impaired insulin sensitivity" OR "non-diabetic hyperglycaemia" OR "metabolic syndrome" OR "dysmetabolic syndrome" OR "cardiometabolic syndrome" OR "cardiometabolic risk factor*" OR hypercholesterolemia OR "high cholesterol level*" OR "elevated cholesterol" OR hypertriglyceridemia OR "high triglyceride level*" OR "lipid disorder*" OR hyperlipidemia OR dyslipidemia OR "high low-density lipoprotein" OR "high LDL") OR AB (overweight OR obes* OR "morbid* obes*" OR adipos* OR corpulence OR "type 2 diabetes mellitus" OR T2DM OR "type 2 diabetes" OR T2D OR "diabetes mellitus type 2" OR "diabetes type 2" OR "type-II diabetes mellitus" OR "adult-onset diabetes" OR "adult onset diabetes" OR "maturity-onset diabetes" OR "maturity onset diabetes" OR "non-insulin-dependent diabetes" OR "noninsulin-dependent diabetes" OR "chronic obstructive pulmonary disease*" OR COPD OR "chronic obstructive lung disease*" OR COLD OR "chronic obstructive airway disease*" OR COAD OR "chronic airflow obstruction*" OR "cardiovascular disease*" OR CVD OR "coronary heart disease*" OR CHD OR "coronary artery disease*" OR CAD OR "coronary disease*" OR "coronary arterioscleros*" OR "coronary atheroscleros*" OR "ischemic heart disease*" OR "ischaemic heart disease*" OR IHD OR "myocardial ischemia*" OR "angina pectoris" OR "stable angina" OR "cardiac failure" OR "heart failure" OR "congestive heart failure" OR CHF OR "left ventricular failure" OR "right ventricular failure" OR "myocardial failure" OR "claudicatio intermittens" OR "peripheral arterial disease*" OR PAD OR "peripheral vascular occlusive disease*" OR PVOD OR hypertension OR "essential hypertension" OR hyperpiesis OR "high blood pressure" OR prediabetes OR "prediabetic state" OR "insulin resistance" OR "glucose intolerance" OR "glucose metabolism disorder*" OR "impaired glucose tolerance" OR "impaired fasting glucose" OR "impaired glucose regulation" OR "impaired insulin sensitivity" OR "non-diabetic hyperglycaemia" OR "metabolic syndrome" OR "dysmetabolic syndrome" OR "cardiometabolic syndrome" OR "cardiometabolic risk factor*" OR hypercholesterolemia OR "high cholesterol level*" OR "elevated cholesterol" OR hypertriglyceridemia OR "high triglyceride level*" OR "lipid disorder*" OR hyperlipidemia OR dyslipidemia OR "high low-density lipoprotein" OR "high LDL")
eHealth	#2	DE ("Information and Communication Technology" OR "Electronic Health Services" OR "Digital Interventions" OR "Mobile Health" OR "Telemedicine" OR "Wearable Devices") OR TI (ehealth* OR "e-health*" OR "electronic health" OR mhealth* OR "m-health*" OR "mobile health" OR "mobile application*" OR "mobile intervention*" OR "smart phone*" OR smartphone* OR "computer application*" OR "computer-based*" OR "internet-based*" OR "web-based" OR "app-based" OR "technology-based" OR "technology-supported" OR "website intervention" OR telemedicine OR telehealth OR "technology-supported health" OR "behavio* change technology" OR "digital* behavio* change intervention*" OR "digital* behavio* intervention*" OR "digital* health intervention*" OR "digital* health coach*" OR "digital* lifestyle

		intervention*" OR "digital* lifestyle behavio*change intervention*" OR "health informatic*" OR "health technology" OR "health promotion technolog*" OR "health interactive technolog*" OR "persuas* technolog*" OR "persuas* health* technolog*") OR AB (ehealth* OR "e-health*" OR "electronic health" OR mhealth* OR "m-health*" OR "mobile health" OR "mobile application*" OR "mobile intervention*" OR "smart phone*" OR smartphone* OR "computer application*" OR "computer-based*" OR "internet-based*" OR "web-based" OR "app-based" OR "technology-based" OR "technology-supported" OR "website intervention" OR telemedicine OR telehealth OR "technology-supported health" OR "behavio* change technology" OR "digital* behavio* change intervention*" OR "digital* behavio* intervention*" OR "digital* health intervention*" OR "digital* health coach*" OR "digital* lifestyle intervention*" OR "digital* lifestyle behavio*change intervention*" OR "health informatic*" OR "health technology" OR "health promotion technolog*" OR "health interactive technolog*" OR "persuas* technolog*" OR "persuas* health* technolog*")
Dynamic tailoring	#3	DE "Personalization" OR TI ("dynamic* tailor*" OR "just-in-time" OR JIT OR "just-in-time adaptive" OR JITAI OR "ecological momentary" OR EMI OR "real-time" OR personali* OR tailor* OR "computer tailored" OR "context tailor*" OR "context aware" OR "context triggered" OR "sensor" OR "sensing" OR interactive*) OR AB ("dynamic* tailor*" OR "just-in-time" OR JIT OR "just-in-time adaptive" OR JITAI OR "ecological momentary" OR EMI OR "real-time" OR personalization OR personalised OR personalized OR tailor* OR "computer tailored" OR "context tailor*" OR "context aware" OR "context triggered" OR "sensor" OR "sensing" OR interactive*)
Physical activity	#4	DE ("Active Living" OR "Exercise" OR "Physical Activity" OR "Sedentary Behavior") OR TI ("physical activit*" OR "physical fitness" OR "active life*" OR "active liv*" OR "activ* behavio*" OR exercise OR workout OR sport* OR "sedentary behavio*") OR AB ("physical activit*" OR "physical fitness" OR "active life*" OR "active liv*" OR "activ* behavio*" OR exercise OR workout OR sport* OR "sedentary behavio*")
Healthy nutrition	#5	DE ("Healthy Eating" OR "Eating Behavior" OR "Diets" OR "Nutrition") OR TI (nutrition OR "nutrition* intake" OR diet OR "diet* intake" OR food OR "food intake" OR "food consumption" OR "nourishing food" OR "healthy nutrition" OR "healthy diet*" OR "healthy eat*" OR "healthy food*" OR "eating behavio*" OR "diet* behavio*") OR AB (nutrition OR "nutrition* intake" OR diet OR "diet* intake" OR "food intake" OR "food consumption" OR "nourishing food" OR "healthy nutrition" OR "healthy diet*" OR "healthy eat*" OR "healthy food*" OR "eating behavio*" OR "diet* behavio*")

Supplementary Table 6. Electronic search strategy ACM Digital Library

Advanced search:	Each ID is used for both a "Title" and "Abstract" string. The query was manually modified to put "OR" instead of an "AND" between the title string and abstract string per set of terms and to put parentheses around the title string and abstract string of each set of terms to separate the different sets.
Publication date:	Since 1 January 2000
Sort on:	Latest

Export: EndNote

Remarks: Term set 2 (eHealth) was not included in the final search string due to the technological specificity of the database. Searched in The ACM Guide to Computing Literature

Topic	ID	Query
**Result**	**#4**	**#1 AND #2 AND (#3 OR #4)**
Chronic conditions	#1	overweight OR obesity OR “morbid obesity” OR adipose OR corpulence OR "type 2 diabetes mellitus" OR T2DM OR “type 2 diabetes” OR T2D OR “diabetes mellitus type 2” OR “diabetes type 2” OR “type-II diabetes mellitus” OR “adult-onset diabetes” OR “adult onset diabetes” OR “maturity-onset diabetes” OR “maturity onset diabetes” OR “non-insulin-dependent diabetes” OR “noninsulin-dependent diabetes” OR “chronic obstructive pulmonary disease” OR COPD OR “chronic obstructive lung disease” OR COLD OR “chronic obstructive airway disease” OR COAD OR “chronic airflow obstruction” OR “cardiovascular disease” OR CVD OR “coronary heart disease” OR CHD OR “coronary artery disease” OR CAD OR “coronary disease” OR “coronary arteriosclerosis” OR “coronary atherosclerosis” OR “ischemic heart disease*” OR “ischaemic heart disease” OR “ischemic heart disease” OR IHD OR “myocardial ischemia” OR “angina pectoris” OR “stable angina” OR “cardiac failure” OR “heart failure” OR “congestive heart failure” OR CHF OR “left ventricular failure” OR “right ventricular failure” OR “myocardial failure” OR “claudicatio intermittens” OR “peripheral arterial disease” OR PAD OR “peripheral vascular occlusive disease*” OR PVOD OR hypertension OR “essential hypertension” OR hyperpiesis OR “high blood pressure” OR prediabetes OR “prediabetic state” OR “insulin resistance” OR “glucose intolerance” OR “glucose metabolism disorder*” OR “impaired glucose tolerance” OR “impaired fasting glucose” OR “impaired glucose regulation” OR “impaired insulin sensitivity” OR “non-diabetic hyperglycaemia” OR “metabolic syndrome” OR “dysmetabolic syndrome” OR “cardiometabolic syndrome” OR “cardiometabolic risk factor*” OR hypercholesterolemia OR “high cholesterol level*” OR “elevated cholesterol” OR hypertriglyceridemia OR “high triglyceride level*” OR “lipid disorder*” OR hyperlipidemia OR dyslipidemia OR “high low-density lipoprotein” OR “high LDL”
Dynamic tailoring	#2	“dynamic tailored” OR “dynamic tailoring” OR “dynamically tailored” OR “just-in-time*” OR JIT OR “just-in-time adaptive” OR JITAI OR “computer tailored” OR “real time” OR “context aware” OR momentary OR personalized OR personalised OR adaptive OR “machine learning” OR “deep learning” OR interactive
Physical activity	#3	“physical activity” OR “physical activities” OR “physical fitness” OR “active lifestyle” OR “active living” OR “active behavior” OR “active behaviour” OR “activity behavior” OR “activity behaviour” OR exercise OR workout OR sport OR sports OR “sedentary behavior” OR “sedentary behaviour”
Healthy nutrition	#4	nutrition OR “nutritional intake” OR diet OR “dietary intake” OR “food intake” OR “food consumption” OR “nourishing food” OR “healthy nutrition” OR “healthy diet” OR “healthy dietary” OR “healthy eating” OR “healthy food” OR “healthy

		foods" OR "eating behavior" OR "eating behaviour" OR "dietary behavior" OR "dietary behaviour"

Supplementary Table 7. Empty Data Extraction Form

**Section 1: General study and intervention characteristics**		
**Field name**	**Value(s)**	**Operationalization**
Aim of the study		The aim of the study.
Study design	☐ Study protocol	The study is a study protocol.
	☐ Design study	The study describes the development/design of an intervention.
	☐ Usability study	The study is described as an usability study.
	☐ Feasibility study	The study is described as a feasibility study .
	☐ Randomized Controlled Trial	The study is a Randomized Controlled Trial (RCT).
	☐ Quasi-Randomized Trial	The study is a quasi-randomized trial.
	☐ Non-Randomized Experimental Study	The study is a non-randomized experimental study.
	☐ Cross sectional study	The study is a cross-sectional study.
	☐ Economic evaluation	The study is a economic evaluation.
	☐ Other, namely:	The study has another design.
Intervention name		Name of the intervention.
Authors		Names of the article authors.
Country		The country in/for which the intervention was developed.
Target behavior(s) intervention (multiple options possible)	☐ Physical activity	The purpose of the intervention is aimed at improving physical activity.
	☐ Sedentary behavior	The purpose of the intervention is aimed at decreasing sedentary behavior.
	☐ Healthy diet	The purpose of the intervention is aimed at adopting healthy eating habits.
	☐ Other, namely:	The intervention has another purpose.
Target population intervention (multiple options possible)	☐ People with diabetes type 2	The intervention targets people with type 2 diabetes.
	☐ People with COPD	The intervention targets people with Chronic Obstructive Pulmonary Disease (COPD).
	☐ People with cardiovascular disease	The intervention targets people with lifestyle-related cardiovascular disease.
	☐ People with metabolic syndrome	The intervention targets people with metabolic syndrome (i.e., combination of overweight, hypertension, hypercholesterolemia, and insulin resistance).
	☐ People with hypercholesterolemia	The intervention targets people with hypercholesterolemia.

	☐ People with hypertension	The intervention targets people with hypertension.
	☐ People with prediabetes	The intervention targets people with prediabetes.
	☐ People with overweight/obesity	The intervention targets people with overweight or obesity.
	☐ Other	The intervention targets another population.
**Section 2: Intervention features**		
**Field name**	**Value(s)**	**Operationalization**
Type of dynamically tailored intervention (multiple options possible)	☐ Support is based on a real-time need	The intervention corresponds directly to a need for real-time support or an opportunity to act positively in line with one's goals [1].
	☐ Support is tailored according to input (data) collected by the system	The content or timing of behavioral support is adapted or tailored according to input (data) collected by the system [1].
	☐ Support is triggered by the system	The support is automatically triggered by the system (e.g. app, website, health-care professional, peer) and not directly by the users themselves [1].
Intervention duration	☐ Unrestricted	The intervention is not tied to a specific duration.
	☐ A specific time frame[a]	The duration of the intervention is restricted to a specific number of weeks.
	☐ Not reported	The duration of the intervention is not reported.
Intervention goals (long and short term)		The goals the intervention is intended to achieve [2].
Goal setting method	☐ Self-set goals	The intervention allows the user to set their own goals without providing guidance.
	☐ Guided goal setting	The intervention guides the user in setting goals, but ultimately the user chooses the goal.
	☐ Automated goals	The intervention automatically sets goals for the user. The user cannot choose the goal himself.
	☐ Goal setting without specification of method	The intervention has a goal-setting option, but the method is not further specified.
	☐ No goal setting	The intervention does not offer goal setting options.
	☐ Not reported	It is not reported whether the intervention offers goal setting options.
(IF "goal setting method" ≠ No goal setting) Goal setting personalization	☐ Generic goals	The intervention goals are generic (i.e., the same goal for all users).
	☐ Personalized goals	The intervention goals are personalized to the user.
	☐ Not reported	It is not reported whether the intervention goals are personalized.
(IF "goal setting method" ≠ No goal setting)	☐ Static goals	The intervention goals are fixed during the intervention period.
	☐ Adaptive goals	The intervention goals can or will be adapted during the intervention period.

Goal setting adaptivity	☐ Not reported	It is not reported whether the intervention goals are static or adaptive.
Dynamic tailoring variables (i.e., Information concerning the individual that is used to dynamically tailor support over time [2]) (multiple options possible)	☐ Lifestyle goals (not specified)	The intervention tailors support to the progress of lifestyle goals at a decision point. Select only if the parameters of behavior are not further specified.
	☐ Physical activity behavior (not specified)	The intervention tailors support to the assessed physical activity behavior at a decision point. Select only if the parameters of behavior are not further specified.
	☐ Eating behavior (not specified)	The intervention tailors support to the assessed eating behavior at a decision point. Select only if the parameters of behavior are not further specified.
	☐ Sedentary behavior time	The intervention tailors support to the time spent in sedentary behavior at a decision point.
	☐ Steps	The intervention tailors support to the step count at a decision point [2].
	☐ Active minutes	The intervention tailors support to the number of minutes spent on light/moderate/vigorous physical activity at a decision point.
	☐ Nutrients	The intervention tailors support to the reported intake of nutrients. [2].
	☐ Determinants of behavior	The intervention tailors support to theoretically determinants of behavior (e.g., self-efficacy, motivation, attitude).
	☐ Current activity	The intervention tailors support to the individual's current activity (e.g., working, reading etc.) [2].
	☐ Location	The intervention tailors support to the individual's current location [2].
	☐ Weather	The intervention tailored support to weather conditions.
	☐ Time of day	The intervention tailors support to a specific time of day [2].
	☐ Other, namely:	The intervention tailors support to other variables.
	☐ Not reported	It is not reported which tailoring variables are applied in the intervention.
Static tailoring variables (i.e., Static information concerning the individual that is used to tailor support [2]) (multiple options possible)	☐ Age	The intervention tailors support to the age (group).
	☐ Gender	The intervention tailors support to gender.
	☐ Social environment	The intervention tailors support to the social environment (e.g., family members, friends).
	☐ Physical environment	The intervention tailors support to the physical environment (e.g., residence)
	☐ Chronic disease	The intervention tailors support to the chronic disease.
	☐ Ethnicity	The intervention tailors support to ethnicity.
	☐ Occupation	The intervention tailors support to an occupation (e.g., sedentary jobs)

	☐ Determinants of behavior	The intervention tailors support to determinants of behavior (e.g., motivation, self-efficacy, phase of behavior change).
	☐ Other, namely:	The intervention tailors support to other variables.
	☐ Not applicable	There are no static tailoring variables used to tailor the intervention.
	☐ Not reported	It is not reported which tailoring variables are applied in the intervention.
Type(s) of data used to trigger tailored support (multiple options possible; only applicable for measurement of the dynamic tailored variables)	☐ Accelerometer/pedometer data	The intervention can access accelerometer or pedometer data [3,4].
	☐ Device-measured vital signs/body parameters	The intervention can access sensor data about vital signs or body parameters (e.g., blood pressure, heart rate, or glucose values) [3].
	☐ Location data (with GPS or antenna)	The intervention can access the device's location [3,4].
	☐ Weather data (internet source/API)	The intervention can access data or information regarding the weather [5].
	☐ Calendar data (internet source/API)	The intervention can access data from the individual's calendar [5].
	☐ Questionnaire(s)	The intervention collects data through questionnaires [4].
	☐ Ecological Momentary Assessment (EMA)	The intervention collects data through Ecological Momentary Assessments (EMAs) [6].
	☐ System-initiated self-reporting	The intervention collects data through self-reporting triggered by the system (e.g., prompt or reminder to self-report in intervention; EMA and questionnaire excluded) [4].
	☐ User-initiated self-reporting	The intervention collects data through self-reporting initiated by the user (i.e., no prompt or reminder to self-report data) [4].
	☐ Interactive Voice Response (IVR)	The intervention collects data through Interactive Voice Response (IVR).
	☐ Other, namely:	The intervention collects or measures data in a different way.
	☐ Not reported	It is not reported which types of data are used to trigger support.
Type(s) of monitor devices that can be used for self-monitoring (multiple options possible)	☐ Activity tracker or smartwatch	It is possible to use an activity tracker (e.g., Fitbit, Xiaomi, Denver) [7] or a smart watch (e.g., Apple/ Samsung/Garmin Watch) [8].
	☐ Smart scale	It is possible to use a smart scale [9].
	☐ Smart blood pressure monitor	It is possible to use a smart blood pressure monitor [9,10].
	☐ Smart ECG monitor	It is possible to use a ECG monitor [9,11].
	☐ Wearable vital sign monitors	It is possible to use a wearable vital sign monitor (e.g., patches, chest straps, clothing monitors, upper band monitors) [12].
	☐ Continuous/Flash glucose monitoring sensor	It is possible to use a Glucose Sensor to the intervention (e.g., Freestyle Libre or Dexcom) [13,14].
	☐ Other, namely:	Users can use other monitoring devices.

	☐ Not applicable	There is no possibility to use/connect monitor devices.
	☐ Not reported	It is not reported which monitoring devices can be used.
Decision points (i.e., points in time at which the intervention decisions are made [2].)	☐ Real-time	Support is triggered based on real-time data (i.e., tailoring variables are measured continuously).
	☐ Pre-specified time-interval	Support is triggered based on a pre-specified time interval (e.g., a tailoring variable is monitored every 5 minutes) [2].
	☐ Pre-defined schedule	Support is triggered based on a pre-defined schedule (e.g., specific times of day/week/month (e.g., every Monday, 2 pm)) [2].
	☐ Random prompts	Support is triggered based on random prompts [2].
	☐ Not reported	It is not reported which decision points are used.
Decision rules (i.e., specifying which intervention option to offer, for whom, and when [2].)	☐ Data-driven approach	Decision rules are operationalized by means of a data-driven approach (e.g., machine learning).
	☐ Knowledge-driven approach	Decision rules are operationalized by means of a non-data driven approach (e.g., knowledge synthesis, expert opinions).
	☐ Other, namely:	Decision rules are operationalized in another way.
	☐ Not reported	It is not reported which decision rules are developed.
Static or adaptive decision rules	☐ Static	Decision rules are static/time-invariant, which means that decision rules are fixed (i.e., independent of the previous history) [15].
	☐ Adaptive	Decision rules in the intervention are adaptive/time-variant, which means that decision rules can change over time (i.e., dependent of previous history).
	☐ Not reported	It is not reported which type of decision rules are developed.
Modes of delivery for intervention options (i.e., array of possible treatments/actions that might be employed at any given decision point [2]) (multiple options possible)	☐ Auditive	Intervention options are delivered via audio (e.g., spoken text) [16].
	☐ Visual	Intervention options are delivered via visuals (e.g., graphs, videos, natural language) [16].
	☐ Haptic	Intervention options are delivered via haptic signals (e.g., vibrations) [16].
	☐ Not reported	It is not reported which modes of delivery are used for the intervention options.
(IF “modes of delivery” = Visual) Visual modes of delivery for intervention options (multiple options possible)	☐ Natural language	Visual intervention options are delivered in text.
	☐ Graphic	Visual intervention options are delivered by a graphical representation of information (e.g., graphs or pictures)
	☐ Video	Intervention options are delivered by videos.
	☐ Other, namely:	Intervention options are delivered in another way.
	☐ Not reported	It is not reported which visual modes of delivery are applied.

User interaction	☐ One-way communication/ static/ non-interactive	Intervention options are delivered as one-way communication (i.e., the user cannot interact with the information).
	☐ Interactive	Intervention options are delivered in an interactive manner (i.e., user can respond to intervention options or has interaction with the system).
	☐ Not reported	It is not reported whether user interaction is applied.
(IF "user interaction" = Interactive) Interactive components as part of intervention options (multiple options possible)	☐ (Embodied) Conversational agent	Intervention options contain interaction with a (embodied) conversational agent or coach.
	☐ Gamification	Intervention options are delivered through games (i.e., gamification elements).
	☐ Other, namely:	Another type of interactive components are applied in the intervention.
	☐ Not reported	It is not reported which interactive components are used.
Intention intervention options (multiple options possible)	☐ Suggestions	The intervention options contain suggestions to give the user a suggestion on how to achieve his desired behavior [5].
	☐ Argument	The intervention option contain arguments to educate the user about the benefits of the healthy behavior (gain-framed) or the negative consequences of the unhealthy behavior (loss framed) and can attempt to convince the user of changing his view on his specific behavior [5].
	☐ Feedback	The intervention options contain feedback on the user's current progress to give the user an insight in his current performance [5].
	☐ Reinforcement	The intervention options contain reinforcement to reinforce the user's current behavior (only applicable when the user is performing well) [5].
	☐ Reminder	The intervention options contain a reminder to perform (earlier planned) healthy behavior.
	☐ Other, namely:	The intervention options contain other intentions.
	☐ Not reported	It is not reported what the intentions of the intervention options are.
**Section 3: Development framework and theoretical basis**		
**Field name**	**Value(s)**	**Operationalization**
Framework used to inform development of the intervention (multiple options possible)	☐ Conceptual model of JITAI components	The intervention is developed based on the conceptual model of JITAI components proposed by Nahum-Shani et al. [2].
	☐ Practical framework for designing just-in-time feedback	The intervention is developed based on the practical framework for designing just-in-time feedback proposed by Schembre et al. [17].
	☐ Planning model for tailored print materials	The intervention is developed based on the planning model for tailored print materials proposed by Kreuter et al. [18].

	☐ Intervention mapping	The intervention is developed based on The Intervention Mapping approach proposed by Bartholomew et al. [19].
	☐ Persuasive System Design (PSD) model	The intervention is developed based on the Persuasive System Design (PSD) model proposed by Oinas-Kukkonen et al. [20].
	☐ CeHRes roadmap	The intervention is developed based on the CeHRes roadmap proposed by van Gemert-Pijnen et al. [21].
	☐ Behavior Change Wheel	The intervention is developed based on the Behavior Change Wheel (BCW) proposed by Michie et al. [22].
	☐ None	The intervention is not developed based on a framework.
	☐ Other, namely:	The intervention is developed based on a different development framework.
	☐ Not reported	It is not reported based on which framework the intervention was developed.
Theory used to inform content of the intervention<br>(multiple options possible)	☐ Social Cognitive Theory (SCT)	Intervention content is based on the Social Cognitive Theory (SCT) [23].
	☐ Theory of Planned Behavior (TPB)	Intervention content is based on the Theory of Planned Behavior (TPB) [24].
	☐ Theory of Reasoned Action (TRA)	Intervention content is based on the Theory of Reasoned Action (TRA) [25].
	☐ Self-Determination Theory (SDT)	Intervention content is based on the Self-Determination Theory (SDT) [26].
	☐ Health Belief Model (HBM)	Intervention content is based on the Health Belief Model (HBM) [27].
	☐ Health Action Process Approach (HAPA)	Intervention content is based on the Health Action Process Approach (HAPA) [28].
	☐ Trans Theoretical Model (TTM)	Intervention content is based on the Trans Theoretical Model (TTM) [29].
	☐ Goal Setting Theory (GST)	Intervention content is based on the Goal Setting Theory (GST) [30].
	☐ Information, Motivation and Behavior skills model (IMB)	Intervention content is based on the Information, Motivation and Behavior skills model (IMB) [31].
	☐ COM-B model	Intervention content is based on the Capability, Opportunity, and Motivation for Behavior change (COM-B) model [22].
	☐ Relapse prevention theory	Intervention content is based on the relapse prevention theory [32].
	☐ Rothman's theory of maintenance	Intervention content is based on the Rothman's theory of maintenance [33].
	☐ None	Intervention content is not based on a behavior change theory.
	☐ Other, namely:	Intervention content is based on another theory.
	☐ Not reported	It is not reported on which theory intervention content is based.
Behavior Change Techniques (BCTs) [34]<br>(multiple options possible)	☐ Goals and planning	The intervention contains BCTs from the category "Goals and planning".
	☐ Feedback and monitoring	The intervention contains BCTs from the category "Feedback and monitoring".
	☐ Social support	The intervention contains BCTs from the category "Social support".

	☐ Shaping knowledge	The intervention contains BCTs from the category “Shaping knowledge”.
	☐ Natural consequences	The intervention contains BCTs from the category “Natural consequences”.
	☐ Comparison of behavior	The intervention contains BCTs from the category “Comparison of behavior”.
	☐ Associations	The intervention contains BCTs from the category “Associations”.
	☐ Repetition and substitution	The intervention contains BCTs from the category “Repetition and substitution”.
	☐ Comparison of outcomes	The intervention contains BCTs from the category “Comparison of outcomes”.
	☐ Reward and threat	The intervention contains BCTs from the category “Reward and threat”.
	☐ Regulation	The intervention contains BCTs from the category “Regulation”.
	☐ Antecedents	The intervention contains BCTs from the category “Antecedents”.
	☐ Identity	The intervention contains BCTs from the category “Identity”.
	☐ Scheduled consequences	The intervention contains BCTs from the category “Scheduled consequences”.
	☐ Self-belief	The intervention contains BCTs from the category “Self-belief”.
	☐ Covert learning	The intervention contains BCTs from the category “Covert learning”.
	☐ Not reported	It is not reported which BCTs were applied.
**Section 4: Way of delivery**		
**Field name**	**Value(s)**	**Operationalization**
Mode of delivery<br>(multiple options possible)	☐ App	The intervention is a smartphone app.
	☐ Website	The intervention is a website for computer or smartphone.
	☐ Smartwatch	The intervention is a smartwatch.
	☐ Text messaging	The intervention consists of text messaging.
	☐ Robot	The intervention is a robot (e.g., Tinybot Tessa).
	☐ Other, namely:	The intervention is delivered in another way.
	☐ Not reported	It is not reported in what way the intervention can be delivered.
Delivery platform<br>(multiple options possible)	☐ iOS/iPadOS	The intervention is available for technology with operating system iOS and/or iPadOS.
	☐ Android	The intervention is available for technology with operating system Android.
	☐ Windows	The intervention is available for technology with operating system Windows.
	☐ Other, namely:	The intervention is available for technology with another operating system.
	☐ Not yet available	The intervention it not yet available on a delivery platform.
	☐ Not applicable	This field is not applicable for the intervention.
	☐ Not reported	It is not reported for which operating system the intervention is available.
Blended-care	☐ Yes	The intervention is combined with face-to-face support.

	☐ No	The intervention is used as stand-alone intervention.
	☐ Not reported	It is not reported whether the intervention was offered as blended-care.
(IF "blended care" = Yes) Blended-care setting (multiple options possible)	☐ General practice	The intervention is offered as part of care from the general practitioner.
	☐ Hospital care	The intervention is offered as part of hospital care.
	☐ Lifestyle coach	The intervention is offered with active involvement of a lifestyle coach (e.g., as part of a combined lifestyle intervention).
	☐ Dietician	The intervention is offered with active involvement of a dietician.
	☐ Physiotherapist	The intervention is offered with active involvement of a physiotherapist.
	☐ Other, namely:	The intervention is offered by another healthcare professional.
	☐ Not reported	It is not reported which healthcare professional is involved.
(IF "blended care" = Yes) Type of blended-support (multiple options possible)	☐ Remote guidance from a healthcare professional	Blended-support is provided remotely (e.g. by telephone or video calling) by a healthcare professional.
	☐ Face-to-face guidance from a healthcare professional	Blended-support is provided face-to-face (i.e., individual or group sessions) by a healthcare professional.
	☐ Other, namely:	Another type of blended-support is provided.
	☐ Not reported	It is not reported which type of blended-support is offered.
**Section 5: Evaluation methods *(if applicable)***		
**Field name**	**Value(s)**	**Operationalization**
Population description (multiple options possible)	☐ People with diabetes type 2	The intervention targets people with type 2 diabetes.
	☐ People with COPD	The intervention targets people with Chronic Obstructive Pulmonary Disease (COPD).
	☐ People with cardiovascular disease	The intervention targets people with lifestyle-related cardiovascular disease.
	☐ People with metabolic syndrome	The intervention targets people with metabolic syndrome (i.e., combination of overweight, hypertension, hypercholesterolemia, and insulin resistance).
	☐ People with hypercholesterolemia	The intervention targets people with hypercholesterolemia.
	☐ People with hypertension	The intervention targets people with hypertension.
	☐ People with prediabetes	The intervention targets people with prediabetes.
	☐ People with overweight/obesity	The intervention targets people with overweight or obesity.
	☐ Other, namely:	The intervention was evaluated among another target group.
Inclusion criteria		The inclusion criteria applied in the study.
Exclusion criteria		The exclusion criteria applied in the study.
Recruitment		The recruitment strategy.

Total number of participants		The number of participants included in the study.
**Section 6: Evaluation results *(if applicable; fill in for every outcome)***		
**Field name**	**Value(s)**	**Operationalization**
Outcome name		The name of the outcome (e.g., HbA1c, BMI, steps per day, perceived effectiveness) provided by the authors.
Outcome definition		The definition of the outcome provided by the authors.
Time points measured		The time points at which the outcome was measured from start or end of intervention.
Unit of measurement		The unit in which the outcome is measured (e.g., mmol/L, kg/m^3)
Results		Summary of the results provided by the authors (e.g. mean, SD, mean difference, CI, P-value, summary of qualitative results)

**Section 7: Other information**	
**Field name**	**Value(s)**
Key conclusions of study authors	
References to other relevant studies	
Correspondence required for further study information *(from whom, what and when)*	
Notes:	

Overview of Supplementary Data Files

Supplementary Data 1

Title: Study Characteristics

Legend: Overview of key characteristics of all included studies, such as research designs, country, target behavior, and target groups.

Supplementary Data 2

Title: Tailoring Strategy

Legend: Detailed description of tailoring strategies, including variables, measurements, tailoring logic, and intervention options.

Supplementary Data 3

Title: Way of Delivery

Legend: Formats, channels, and modalities used for intervention delivery.

Supplementary Data 4

Title: Theory and Behavior Change Techniques (BCTs)

Legend: Theoretical models referenced in each intervention and the behavior change techniques applied, categorized by BCT taxonomy.

Supplementary Data 5

Title: Study Evaluation and Participant Characteristics

Legend: Information on inclusion and exclusion criteria, sample size, participant demographics, and evaluation methods.

Supplementary Data 6

Title: Study Findings

Legend: Reported behavioral and clinical outcomes, and acceptability and adherence data, grouped by intervention.

Supplementary References

1 Hardeman, W., Houghton, J., Lane, K., Jones, A. & Naughton, F. A systematic review of just-in-time adaptive interventions (JITAIs) to promote physical activity. *International Journal of Behavioral Nutrition and Physical Activity.* **16**, 31, 10.1186/s12966-019-0792-7 (2019).

2 Nahum-Shani, I. *et al.* Just-in-Time Adaptive Interventions (JITAIs) in Mobile Health: Key Components and Design Principles for Ongoing Health Behavior Support. *Ann Behav Med.* **52**, 446-462, 10.1007/s12160-016-9830-8 (2018).

3 Cornet, V. P. & Holden, R. J. Systematic review of smartphone-based passive sensing for health and wellbeing. *Journal of Biomedical Informatics.* **77**, 120-132, https://doi.org/10.1016/j.jbi.2017.12.008 (2018).

4 Bush, N. E., Armstrong, C. M. & Hoyt, T. V. Smartphone apps for psychological health: A brief state of the science review. *Psychological Services.* **16**, 188-195, 10.1037/ser0000286 (2019).

5 op den Akker, H., Cabrita, M., op den Akker, R., Jones, V. M. & Hermens, H. J. Tailored motivational message generation: A model and practical framework for real-time physical activity coaching. *Journal of Biomedical Informatics.* **55**, 104-115, https://doi.org/10.1016/j.jbi.2015.03.005 (2015).

6 de Vries, L. P., Baselmans, B. M. L. & Bartels, M. Smartphone-Based Ecological Momentary Assessment of Well-Being: A Systematic Review and Recommendations for Future Studies. *J Happiness Stud.* **22**, 2361-2408, 10.1007/s10902-020-00324-7 (2021).

7 Fitbit Inc. *Fitbit Fitness Trackers*, https://www.fitbit.com/global/us/products/trackers (2022).

8 Xiaomi. *Xiaomi Wearable*, https://www.mi.com/nl/life-style#wearable (2022).

9 Nederend, M. *et al.* Potential of eHealth smart technology in optimization and monitoring of heart failure treatment in adults with systemic right ventricular failure. *European Heart Journal - Digital Health.* **2**, 215-223, 10.1093/ehjdh/ztab028 (2021).

10 Rodriguez, S., Hwang, K. & Wang, J. Connecting Home-Based Self-Monitoring of Blood Pressure Data Into Electronic Health Records for Hypertension Care: A Qualitative Inquiry With Primary Care Providers. *JMIR Form Res.* **3**, e10388, 10.2196/10388 (2019).

11 Guo, S. L. *et al.* The future of remote ECG monitoring systems. *J Geriatr Cardiol.* **13**, 528-530, 10.11909/j.issn.1671-5411.2016.06.015 (2016).

12 Soon, S., Svavarsdottir, H., Downey, C. & Jayne, D. G. Wearable devices for remote vital signs monitoring in the outpatient setting: an overview of the field. *BMJ Innovations.* **6**, 55, 10.1136/bmjinnov-2019-000354 (2020).

13 Abbott. *Freestyle Libre*, https://www.freestyle.abbott/nl-nl/home.html (2022).

14 Dexcom. *Glucosewaarschuwingen in realtime op uw smartphone*, https://www.dexcom.com/nl-NL (2022).

15 Perski, O. *et al.* Technology-mediated just-in-time adaptive interventions (JITAIs) to reduce harmful substance use: a systematic review. *Addiction.* **117**, 1220-1241, https://doi.org/10.1111/add.15687 (2022).

16 Op den Akker, H., Cabrita, M., Op den Akker, R., Jones, V. M. & Hermens, H. J. Tailored motivational message generation: A model and practical framework for real-time physical activity coaching. *J Biomed Inform.* **55**, 104-115, 10.1016/j.jbi.2015.03.005 (2015).

17 Schembre, S. M. *et al.* Just-in-Time Feedback in Diet and Physical Activity Interventions: Systematic Review and Practical Design Framework. *J Med Internet Res.* **20**, e106, 10.2196/jmir.8701 (2018).

18 Kreuter, M., Strecher, V. & Glassman, B. One size does not fit all: The case for tailoring print materials. *Ann Behav Med.* **21**, 276-283, 10.1007/BF02895958 (1999).

19 Bartholomew, L. K., Parcel, G. S. & Kok, G. Intervention mapping: a process for developing theory- and evidence-based health education programs. *Health Educ Behav.* **25**, 545-563, 10.1177/109019819802500502 (1998).
20 Oinas-Kukkonen, H. & Harjumaa, M. Persuasive Systems Design: Key Issues, Process Model, and System Features. *Communications of the Association for Information Systems.* **24**, 10.17705/1CAIS.02428 (2009).
21 van Gemert-Pijnen, J. E. *et al.* A holistic framework to improve the uptake and impact of eHealth technologies. *J Med Internet Res.* **13**, e111, 10.2196/jmir.1672 (2011).
22 Michie, S., van Stralen, M. M. & West, R. The behaviour change wheel: a new method for characterising and designing behaviour change interventions. *Implement Sci.* **6**, 42, 10.1186/1748-5908-6-42 (2011).
23 Bandura, A. *Social foundations of thought and action: A social cognitive theory*. (Prentice-Hall, Inc, 1986).
24 Ajzen, I. The theory of planned behavior. *Organizational Behavior and Human Decision Processes.* **50**, 179-211, https://doi.org/10.1016/0749-5978(91)90020-T (1991).
25 Fishbein, M. & Ajzen, I. *Belief, attitude, intention and behaviour: An introduction to theory and research*. Vol. 27 (1975).
26 Deci, E. L. a. & Ryan, R. M. a. *Intrinsic motivation and self-determination in human behavior*. (New York (N.Y.) : Plenum, 1985).
27 Sheeran, P. & Abraham, C. in *Predicting health behaviour: Research and practice with social cognition models.* 23-61 (Open University Press, 1996).
28 Schwarzer, R. Modeling Health Behavior Change: How to Predict and Modify the Adoption and Maintenance of Health Behaviors. *Applied Psychology.* **57**, 1-29, 10.1111/j.1464-0597.2007.00325.x (2008).
29 Prochaska, J. O. & DiClemente, C. C. Stages and processes of self-change of smoking: Toward an integrative model of change. *Journal of Consulting and Clinical Psychology.* **51**, 390-395, 10.1037/0022-006X.51.3.390 (1983).
30 Locke, E. & Latham, G. A Theory of Goal Setting & Task Performance. *The Academy of Management Review.* **16**, 10.2307/258875 (1991).
31 Fisher, W. A., Fisher, J. D. & Harman, J. in *Social psychological foundations of health and illness. Blackwell series in health psychology and behavioral medicine.* 82-106 (Blackwell Publishing, 2003).
32 Larimer, M. E., Palmer, R. S. & Marlatt, G. A. Relapse prevention. An overview of Marlatt's cognitive-behavioral model. *Alcohol Res Health.* **23**, 151-160 (1999).
33 Rothman, A. Toward a theory-based analysis of behavioral maintenance. *Health psychology : official journal of the Division of Health Psychology, American Psychological Association.* **19**, 64-69, 10.1037//0278-6133.19.Suppl1.64 (2000).
34 Michie, S. *et al.* The behavior change technique taxonomy (v1) of 93 hierarchically clustered techniques: building an international consensus for the reporting of behavior change interventions. *Ann Behav Med.* **46**, 81-95, 10.1007/s12160-013-9486-6 (2013).
