# Supplementary material for: Systematic review of dynamically tailored eHealth interventions targeting physical activity and healthy diet in chronic disease
Source: NPJ Digit Med. 2025 Nov 19;8:696. doi: 10.1038/s41746-025-02054-7 (PMC12630729; doi:10.1038/s41746-025-02054-7)
Supplement: Supplementary file 2 — Supplementary data1 [file 41746_2025_2054_MOESM2_ESM.pdf]

Supplementary Data 1. Study Characteristics

Author (year)	Intervention name	Study design	Country	Health condition	Target behavior(s)	Intervention duration	Study duration
**Aguilera (2020[1])**	DIAMANTE	• Study protocol	United States	• Diabetes type 2<br>• Depressive symptoms	• Physical activity	6-9 months	6 months
**Almeida (2015[2])**<br>**Estabrooks (2011[3])**	CardiACTION	• Design study<br>• Randomized controlled trial	United States	• Cardiovascular disease	• Physical activity	6 months	6 months
**Alos (2022[4])**	Walk@Work	• Study protocol	Spain	• Diabetes type 2	• Physical activity<br>• Sedentary behavior	13 weeks	12 months
**Al-Ozari (2018[5])**	DATES	• Study protocol	Kuwait	• Diabetes type 2	• Physical activity<br>• Healthy diet	12 months	12 months
**Ambeba (2015[6])**<br>**Bizhanova (2023[7])**<br>**Burke (2017[8], 2020[9], 2022a[10], 2022b[11])**<br>**Cheng (2023[12])**<br>**Kariuki (2023[13])**<br>**Wang (2012[14])**	SMARTER	• Study protocol<br>• Feasibility study<br>• 3 randomized controlled trials<br>• 3 secondary analyses of a randomized controlled trial<br>• Mediation analysis based on randomized controlled trial data	United States	• Overweight/obesity	• Physical activity<br>• Healthy diet	12 months	12 months
**Baert (2018[15])**<br>**Bohanec (2021[16])**<br>**Clays (2021[17])**<br>**Voorend (2019[18])**	HeartMan DSS	• Study protocol<br>• Design study<br>• Qualitative evaluation study<br>• Proof-of-concept trial	Belgium/ Italy	• Cardiovascular disease	• Physical activity<br>• Healthy diet<br>• Medication intake	3-6 months	3-6 months

**Beckie (2024[19])**<br>**Sengupta (2020a[20], 2020b[21])**	HerBeat	• Design study<br>• Usability study<br>• Pilot randomized controlled trial	United States	• Cardiovascular disease	• Physical activity<br>• Healthy diet<br>• Stress management	3 months	3 months
**Bennett (2013[22], 2018[23])**<br>**Foley (2012[24], 2016[25])**<br>**Steinberg (2013[26])**	Shape/ Track	• 2 study protocols<br>• Feasibility study<br>• 2 randomized controlled trials	United States	• Overweight/obesity	• Physical activity;<br>• Healthy diet	Steinberg: 6 months<br>Bennett: 12 months	Steinberg: 6 months<br>Bennett: 12 months
**Boh (2016[27])**	Think Slim	• Study protocol	Netherlands/ Belgium	• Overweight/obesity	• Healthy diet	6 weeks	12 months
**Bond (2014[28])**<br>**Thomas (2015[29])**	B-MOBILE	• A Randomized Within-Subjects Experimental Trial<br>• Secondary analysis of a Randomized Within-Subjects Experimental Trial	United States	• Overweight/obesity	• Sedentary behavior	3 weeks (1 week per condition)	3 weeks
**Boudreau (2016[30])**<br>**Moreau (2015[31])**	Diabète en Forme (DEF)	• Study protocol<br>• Design study	Canada	• Diabetes type 2	• Physical activity	8 weeks	9 months
**Buchan (2020[32])**	Onitor	• Mixed method feasibility and usability study	United Kingdom	• Overweight/obesity	• Physical activity<br>• Healthy diet	4 weeks	4 weeks
**Chokshi (2017[33])**	ACTIVE REWARD	• Randomized controlled trial	United States	• Cardiovascular disease	• Physical activity	16 weeks	24 weeks
**Collins (2010[34], 2012[35], 2013[36])**	The Biggest Loser Club	• Study protocol<br>• 2 randomized controlled trials	Australia	• Overweight/obesity	• Physical activity<br>• Healthy diet	Up to 18 months (phase 1: 12 weeks with optional 12 additional weeks; phase 2: 12 or 15 months)	24 weeks

**Daryabeygi-Khotbehsara (2022[37], 2023[38])**	iMove	• Study protocol<br>• Design study	Australia	• Diabetes type 2	• Physical activity<br>• Sedentary behavior	6 weeks	6 weeks
**Dorsch (2018[39], 2020[40])**	LowSalt4Life	• Study protocol<br>• Pilot randomized controlled trial	United States	• Hypertension	• Healthy diet	8 weeks	8 weeks
**Evans (2015[41])**	NULevel	• Study protocol	United Kingdom	• Overweight/obesity	• Physical activity<br>• Healthy diet	6 months	12 months
**Finkelstein (2015[42])**	Not reported	• Randomised feasibility study (cross-over design)	United States	• Overweight/obesity	• Sedentary behavior	4 weeks	8 weeks
**Forman (2019a[43], 2019b[44])<br>Goldstein (2017[45], 2020[46], 2021a[47], 2021b[48])**	OnTrack/ DietAlert	• Study protocol<br>• Design study<br>• Feasibility study<br>• 2 randomized controlled trials<br>• Secondary analysis of a randomized controlled trial	United States	• Overweight/obesity	• Healthy diet	Forman2019a: 8 weeks<br>Forman2019b: 10 weeks	Forman2019a: 8 weeks<br>Forman2019b: 10 weeks
**Gatwood (2020[49])**	Model	• Design study	United States	• Diabetes type 2	• Physical activity<br>• Healthy diet<br>• Medication adherence	1 year	1 year
**Golbus (2024[50])<br>Hellem (2023[51])**	myBPmyLife	• 2 design studies	United States	• Hypertension	• Physical activity<br>• Healthy diet	6 months	6 months
**Gupta (2015[52])**	Let's Exercise	• Design study	India	• Overweight/obesity	• Physical activity	4 weeks	4 weeks
**Hamborg (2024[53])<br>Martens Anderson (2022[54])**	FAIR	• Study protocol<br>• Feasibility study	Denmark	• Cardiovascular disease	• Physical activity	12 weeks	12 weeks

**Hemnes (2021[55])**<br>**Martin (2015[56])**	mActive	• 2 randomized controlled trials	United States	• Pulmonary arterial hypertension	• Physical activity	Martin: 5 weeks<br>Hemnes: 12 weeks	Martin: 5 weeks<br>Hemnes: 12 weeks
**Hietbrink (2023a[57], 2023b[58])**	E-Supporter	• Design study<br>• Feasibility study	Netherlands	• Diabetes type 2	• Physical activit<br>• Healthy diet	Hietbrink2023a: 10 weeks<br>Hietbrink2023b: 9 weeks	Hietbrink2023a: 5 weeks<br>Hietbrink2023b: 9 weeks
**Hurley (2015[59])**	WalkIT	• Factorial Randomized Controlled Trial	United States	• Overweight/obesity	• Physical activity	4 months	4 months
**Khunti (2021[60])**<br>**Morton (2015[61])**<br>**Yates (2015[62])**	PROPELS/ Walking Away	• Study protocol<br>• Design study<br>• Randomized controlled trial	United Kingdom	• Prediabetes	• Physical activity	48 months	48 months
**Kim (2024[63])**<br>**Park (2024[64])**	APSC program	• Feasibility study<br>• Mixed-method user experience study	Korea	• Diabetes type 2	• Physical activity<br>• Healthy diet	3 months	3 months
**Klein (2014[65])**	eMate	• Design study	Netherlands	• Diabetes type 2<br>• Cardiovascular disease<br>• HIV	• Physical activity<br>• Healthy diet	3 months	3 months
**Korinek (2018[66])**	JustWalk	• Design and feasibility study	United States	• Overweight/obesity	• Physical activity	12 weeks	14 weeks
**Leitner (2022[67])**	P3.AI	• Design and thee-armed clinical trial	United States	• Hypertension	• Physical activity<br>• Healthy diet	6 months	6 months
**Lim (2016[68])**	CDSS u-healthcare service	• Randomized controlled trial	Korea	• Diabetes type 2	• Physical activity<br>• Healthy diet	6 months	6 months

**Lin (2015[69])**	TRIMM	• Randomized controlled trial	United States	• Overweight/obesity	• Physical activity<br>• Healthy diet	6 months	12 months
**Mansour-Assi (2022[70])**	Smart2.0	• Study protocol	United States	• Overweight/obesity	• Physical activity<br>• Healthy diet	24 months	24 months
**Martinho (2023[71])**<br>**Pinto (2022[72])**	FoodFriend	• 2 design studies	Portugal	• Diabetes type 2	• Healthy diet	Not reported	Not reported
**Miller (2021[73])**	Nourish app	• Study protocol	United States	• Hypertension	• Healthy diet	6 months	12 months
**Nezami (2022[74])**	PATH trial	• Pilot randomized controlled trial	United States	• Overweight/obesity	• Physical activity<br>• Healthy diet	6 months	6 months
**Novak (2024[75])**<br>**Vetrovsky (2023[76])**	ENERGISED	• Study protocol<br>• Design study	Czech Republic	• Diabetes type 2	• Physical activity<br>• Sedentary behavior	Vetrovsky: 12 months<br>Novak: 2 weeks	Vetrovsky: 12 months<br>Novak: 2 weeks
**Pardos (2023[77])**	Not reported	• Design study	Greece	• COPD<br>• Other diseases that are not further specified	• Physical activity<br>• Sleep<br>• Mental health	Not reported	Not reported
**Park (2024[78])**	Sit Less Program	• Study protocol	United States	• Cardiovascular disease	• Sedentary behavior	12 weeks	12 weeks
**Pellegrini (2015[79])**	NEAT	• Feasibility study	United States	• Diabetes type 2	• Sedentary behavior	1 month	1 month
**Pimenta (2022[80])**	Vitoria	• Design study	Portugal	• Diabetes type 2	• Physical activity	8 days to several weeks (the follow-up phase depends on patient adherence to the target behavior)	NA

**Plaete (2015[81])**<br>**Poppe (2017[82], 2018[83], 2019a[84], 2019b[85])**	MyPlan2.0	• Study protocol<br>• Design study<br>• Randomized controlled trial<br>• 2 qualitative acceptability studies	Belgium	• Diabetes type 2	• Physical activity<br>• Sedentary behavior<br>• Healthy diet	5 weeks	5 weeks
**Radhakrishnan (2020[86], 2021[87])**	Heart Mountain	• Usability study<br>• A feasibility randomized controlled trial	United States	• Cardiovascular disease	• Physical activity	12 weeks	24 weeks
**Reinwand (2013[88])**<br>**Storm (2016[89])**	RENATA	• Study protocol<br>• Randomized controlled trial	Netherlands/ Germany	• Completed cardiac rehabilitation	• Physical activity<br>• Healthy diet	8 weeks	12 months
**Richardson (2007[90], 2010[91])**	Stepping Up to Health	• Randomized controlled trial<br>• Pilot randomized controlled trial	United States	• Diabetes type 2<br>• Cardiovascular disease<br>• Overweight/obesity	• Physical activity	16 weeks	16 weeks
**Schoenthaler (2020[92])**	i-Matter	• Development and usability study	United States	• Diabetes type 2	• Physical activity<br>• Healthy diet<br>• Sleep<br>• Medication	Not reported	2 weeks
**Schultz (2022[93])**	GPI Tailored Park Prescriptions	• Study protocol	United States	• Diabetes type 2<br>• Prediabetes<br>• Hypertension	• Physical activity	3 months	3 months
**Shibuta (2023[94])**	DialBetes Step	• Feasibility study	Japan	• Hypertension	• Physical activity	24 weeks	24 weeks
**Spruijt-Metz (2022[95])**	HeartStepsII	• Study protocol	United States	• Overweight/obesity	• Physical activity<br>• Sedentary behavior	12 months	12 months
**Stein (2019[96])**<br>**Tabak (2018[97])**	Working for You	• Study protocol<br>• Design study	United States	• Overweight/obesity	• Physical activity<br>• Healthy diet	24 months	24 months

**Steinberg (2020[98])**	DASH Cloud	• Randomized Controlled Feasibility Trial	United States	• Hypertension<br>• People with prehypertension	• Healthy diet	3 months	3 months
**Sun (2020[99])**	BeActive	• Feasibility study	South Korea	• Cardiovascular disease	• Sedentary behavior	1 week	1 week
**Sze (2023[100])<br>Waki (2024[101])**	StepAdd	• Study protocol<br>• Pre-post evaluation design	Japan	• Diabetes type 2	• Physical activity	12 weeks	12 weeks
**Tabak (2013[102], 2014a[103], 2014b[104], 2014c[105])<br>Wieringa (2011[106])**	Activity Coach	• Design study<br>• Feasibility study<br>• Pilot randomized controlled trial<br>• A single-case experimental design (SCED)<br>• Analysis of an intervention arm of a randomized controlled trial	Netherlands	• COPD	• Physical activity	Tabak2013: NA (think-aloud); Tabak2014a: 4 weeks; Tabak2014b: 9 months	Tabak2013: NA (think-aloud); Tabak2014a: 4 weeks; Tabak2014b: 9 months
**Tamura (2020[107])**	Step It Up (Tailored-to-Place Messages)	• Study protocol	United States	• Overweight/obesity and insulin resistance	• Physical activity	6 months	6 months
**vanderWeegen (2013[108], 2015[109])<br>Verwey (2014a[110], 2014b[111])**	It's LiFe!	• Study protocol<br>• Design study<br>• Feasibility study<br>• Randomized controlled trial	Netherlands	• Diabetes type 2<br>• COPD	• Physical activity	4-6 months	9 months

**vanGenugten (2010[112], 2012[113], 2014[114])**	Gripp	• Design study<br>• 2 randomized controlled trials	Netherlands	• Overweight/obesity	• Physical activity<br>• Healthy diet	4 weeks	6 months
**Watson (2012[115])**	Virtual Coach Program	• Randomized controlled trial	United States	• Overweight/obesity	• Physical activity	12 weeks	12 weeks
**Yom-Tov (2017[116])**	Not reported	• Micro randomized trial	Israel	• Diabetes type 2	• Physical activity	26 weeks	26 weeks
**Zahedani (2023[117])**	Season of Me	• Non-randomized experimental study	United States	• Diabetes type 2	• Physical activity<br>• Healthy diet	12 weeks (4-weeks with CGM + 8-weeks without CGM)	12 weeks

References

1 Aguilera, A. *et al.* mHealth app using machine learning to increase physical activity in diabetes and depression: clinical trial protocol for the DIAMANTE Study. *Bmj Open*. **10**, e034723, doi: 10.1136/bmjopen-2019-034723 (2020).

2 Almeida, F. A. *et al.* An Interactive Computer Session to Initiate Physical Activity in Sedentary Cardiac Patients: Randomized Controlled Trial. *J Med Internet Res*. **17**, e206, doi:10.2196/jmir.3759 (2015).

3 Estabrooks, P. A. *et al.* Building a multiple modality, theory-based physical activity intervention: The development of CardiACTION! *Psychol Sport Exerc*. **12**, 46-53, doi: 10.1016/j.psychsport.2010.04.012 (2011).

4 Alòs, F. *et al.* Effectiveness of a healthcare-based mobile intervention on sedentary patterns, physical activity, mental well-being and clinical and productivity outcomes in office employees with type 2 diabetes: study protocol for a randomized controlled trial. *BMC Public Health*. **22**, 1269, doi:10.1186/s12889-022-13676-x (2022).

5 Al-Ozairi, E. *et al.* Diabetes and TelecommunicationS (DATES) study to support self-management for people with type 2 diabetes: a randomized controlled trial. *BMC Public Health*. **18**, doi:10.1186/s12889-018-6136-8 (2018).

6 Ambeba, E. J. *et al.* The Use of mHealth to Deliver Tailored Messages Reduces Reported Energy and Fat Intake. *Journal of Cardiovascular Nursing*. **30**, 35-43, doi:10.1097/jcn.0000000000000120 (2015).

7 Bizhanova, Z. *et al.* Identifying Predictors of Adherence to the Physical Activity Goal: A Secondary Analysis of the SMARTER Weight Loss Trial. *Med Sci Sports Exerc.* **55**, 856-864, doi: 10.1249/mss.0000000000003114 (2023).
8 Burke, L. E. *et al.* The SMARTER pilot study: Testing feasibility of real-time feedback for dietary self-monitoring. *Prev Med Rep.* **6**, 278-285, doi:10.1016/j.pmedr.2017.03.017 (2017).
9 Burke, L. E. *et al.* The SMARTER Trial: Design of a trial testing tailored mHealth feedback to impact self-monitoring of diet, physical activity, and weight. *Contemp Clin Trials.* **91**, 105958, doi:10.1016/j.cct.2020.105958 (2020).
10 Burke, L. E. *et al.* Effect of tailored, daily feedback with lifestyle self-monitoring on weight loss: The SMARTER randomized clinical trial. *Obesity.* **30**, 75-84, doi:10.1002/oby.23321 (2022).
11 Burke, L. E. *et al.* The Effect of Tailored, Daily, Smartphone Feedback to Lifestyle Self-Monitoring on Weight Loss at 12 Months: the SMARTER Randomized Clinical Trial. *J Med Internet Res.* **24**, e38243, doi:10.2196/38243 (2022).
12 Cheng, J. *et al.* Effect of an mHealth weight loss intervention on Healthy Eating Index diet quality: the SMARTER randomised controlled trial. *British Journal of Nutrition.* 1-9, doi: 10.1017/S0007114523001137 (2023).
13 Kariuki, J. K. *et al.* The Association between Neighborhood Walkability and Physical Activity in a Behavioral Weight Loss Trial Testing the Addition of Remotely Delivered Feedback Messages to Self-Monitoring. *Behavioral Medicine.* doi: 10.1080/08964289.2023.2238102 (2023).
14 Wang, J. *et al.* Effect of adherence to self-monitoring of diet and physical activity on weight loss in a technology-supported behavioral intervention. *Patient Preference and Adherence.* **6**, 221-226, doi:10.2147/ppa.S28889 (2012).
15 Baert, A. *et al.* A Personal Decision Support System for Heart Failure Management (HeartMan): study protocol of the HeartMan randomized controlled trial. *BMC Cardiovascular Disorders.* **18**, 186, doi: 10.1186/s12872-018-0921-2 (2018).
16 Bohanec, M. *et al.* HeartMan DSS: A decision support system for self-management of congestive heart failure. *Expert Systems with Applications.* **186**, doi:10.1016/j.eswa.2021.115688 (2021).
17 Clays, E. *et al.* Proof-of-concept trial results of the HeartMan mobile personal health system for self-management in congestive heart failure. *Scientific Reports.* **11**, 5663, doi: 10.1038/s41598-021-84920-4 (2021).
18 Voorend, R., Derboven, J., Slegers, K., Baert, A. & Clays, E. *Human Agency in Self-Management Tools*. (Association for Computing Machinery, 2019).
19 Beckie, T. M. *et al.* A Mobile Health Behavior Change Intervention for Women with Coronary Heart Disease: A Randomized Controlled Pilot Study. *Journal of Cardiopulmonary Rehabilitation and Prevention.* **44**, 40-48, doi: 10.1097/HCR.0000000000000804 (2024).
20 Sengupta, A., Beckie, T., Dutta, K., Dey, A. & Chellappan, S. A Mobile Health Intervention System for Women With Coronary Heart Disease: Usability Study. *JMIR Form Res.* **4**, e16420, doi: 10.2196/16420 (2020).

21 Sengupta, A., Dutta, K., Beckie, T. & Chellappan, S. Designing a Health Coach-Augmented mHealth System for the Secondary Prevention of Coronary Heart Disease Among Women. *IEEE Transactions on Engineering Management*. **69**, 3085-3100, doi: 10.1109/TEM.2020.2997662 (2020).
22 Bennett, G. G. *et al*. Behavioral treatment for weight gain prevention among black women in primary care practice: a randomized clinical trial. *JAMA Intern Med*. **173**, 1770-1777, doi: 10.1001/jamainternmed.2013.9263 (2013).
23 Bennett, G. G. *et al*. Effectiveness of an App and Provider Counseling for Obesity Treatment in Primary Care. *Am J Prev Med*. **55**, 777-786, doi: 10.1016/j.amepre.2018.07.005 (2018).
24 Foley, P. *et al*. Weight gain prevention among black women in the rural community health center setting: The Shape Program. *BMC Public Health*. **12**, doi:10.1186/1471-2458-12-305 (2012).
25 Foley, P. *et al*. Track: A randomized controlled trial of a digital health obesity treatment intervention for medically vulnerable primary care patients. *Contemporary Clinical Trials*. **48**, 12-20, doi:10.1016/j.cct.2016.03.006 (2016).
26 Steinberg, D. M., Levine, E. L., Askew, S., Foley, P. & Bennett, G. G. Daily Text Messaging for Weight Control Among Racial and Ethnic Minority Women: Randomized Controlled Pilot Study. *Journal of Medical Internet Research*. **15**, doi:10.2196/jmir.2844 (2013).
27 Boh, B. *et al*. An Ecological Momentary Intervention for weight loss and healthy eating via smartphone and Internet: study protocol for a randomised controlled trial. *Trials*. **17**, 154, doi:10.1186/s13063-016-1280-x (2016).
28 Bond, D. S. *et al*. B-MOBILE--a smartphone-based intervention to reduce sedentary time in overweight/obese individuals: a within-subjects experimental trial. *PLoS One*. **9**, e100821, doi: 10.1371/journal.pone.0100821 (2014).
29 Thomas, J. G. & Bond, D. S. Behavioral Response to a Just-in-Time Adaptive Intervention (JITAI) to Reduce Sedentary Behavior in Obese Adults: Implications for JITAI Optimization. *Health Psychology*. **34**, 1261-1267, doi:10.1037/hea0000304 (2015).
30 Boudreau, F., Moreau, M. & Côté, J. Effectiveness of Computer Tailoring Versus Peer Support Web-Based Interventions in Promoting Physical Activity Among Insufficiently Active Canadian Adults With Type 2 Diabetes: Protocol for a Randomized Controlled Trial. *JMIR Res Protoc*. **5**, e20, doi:10.2196/resprot.5019 (2016).
31 Moreau, M., Gagnon, M. P. & Boudreau, F. Development of a fully automated, web-based, tailored intervention promoting regular physical activity among insufficiently active adults with type 2 diabetes: integrating the I-change model, self-determination theory, and motivational interviewing components. *JMIR Res Protoc*. **4**, e25, doi:10.2196/resprot.4099 (2015).
32 Buchan, K. & Morgan, H. M. Using the Onitor (R) Track for weight loss: A mixed methods study among overweight and obese women. *Health Informatics Journal*. **26**, 1841-1865, doi:10.1177/1460458219890790 (2020).

33 Chokshi, N. P. *et al*. Loss-framed Financial Incentives and Personalized Goal Setting Increase Physical Activity in Ischemic Heart Disease Patients Using Wearable Devices: The ACTIVE REWARD Randomized Clinical Trial. *Circulation*. **136**, doi: 10.1161/JAHA.118.009173 (2017).
34 Collins, C. E. *et al*. Evaluation of a commercial web-based weight loss and weight loss maintenance program in overweight and obese adults: a randomized controlled trial. *BMC Public Health*. **10**, doi:10.1186/1471-2458-10-669 (2010).
35 Collins, C. E. *et al*. A 12-Week Commercial Web-Based Weight-Loss Program for Overweight and Obese Adults: Randomized Controlled Trial Comparing Basic Versus Enhanced Features. *Journal of Medical Internet Research*. **14**, doi:10.2196/jmir.1980 (2012).
36 Collins, C. E., Morgan, P. J., Hutchesson, M. J. & Callister, R. Efficacy of Standard Versus Enhanced Features in a Web-Based Commercial Weight-Loss Program for Obese Adults, Part 2: Randomized Controlled Trial. *Journal of Medical Internet Research*. **15**, 84-105, doi:10.2196/jmir.2626 (2013).
37 Daryabeygi-Khotbehsara, R. *et al*. Development of an Android Mobile Application for Reducing Sitting Time and Increasing Walking Time in People with Type 2 Diabetes. *Electronics*. **11**, doi:10.3390/electronics11193011 (2022).
38 Daryabeygi-Khotbehsara, R. *et al*. Just-In-Time Adaptive Intervention to Sit Less and Move More in People With Type 2 Diabetes: Protocol for a Microrandomized Trial. *JMIR Res Protoc*. **12**, e41502, doi: 10.2196/41502 (2023).
39 Dorsch, M. P., An, L. C. & Hummel, S. L. A Novel Just-in-Time Contextual Mobile App Intervention to Reduce Sodium Intake in Hypertension: Protocol and Rationale for a Randomized Controlled Trial (LowSalt4Life Trial). *JMIR Res Protoc*. **7**, e11282, doi: 10.2196/11282 (2018).
40 Dorsch, M. P. *et al*. Effects of a Novel Contextual Just-In-Time Mobile App Intervention (LowSalt4Life) on Sodium Intake in Adults With Hypertension: Pilot Randomized Controlled Trial. *JMIR Mhealth Uhealth*. **8**, e16696, doi:10.2196/16696 (2020).
41 Evans, E. H. *et al*. The NULevel trial of a scalable, technology-assisted weight loss maintenance intervention for obese adults after clinically significant weight loss: study protocol for a randomised controlled trial. *Trials*. **16**, 421, doi:10.1186/s13063-015-0931-7 (2015).
42 Finkelstein, J. *et al*. *Mobile App to Reduce Inactivity in Sedentary Overweight Women*. Vol. 216 (2015).
43 Forman, E. M. *et al*. OnTrack: development and feasibility of a smartphone app designed to predict and prevent dietary lapses. *Transl Behav Med*. **9**, 236-245, doi:10.1093/tbm/iby016 (2019).
44 Forman, E. M. *et al*. Randomized controlled trial of OnTrack, a just-in-time adaptive intervention designed to enhance weight loss. *Transl Behav Med*. **9**, 989-1001, doi:10.1093/tbm/ibz137 (2019).

45 Goldstein, S. P. *et al.* Return of the JITAI: Applying a Just-in-Time Adaptive Intervention Framework to the Development of m-Health Solutions for Addictive Behaviors. *International Journal of Behavioral Medicine.* **24**, 673-682, doi:10.1007/s12529-016-9627-y (2017).

46 Goldstein, S. P. *et al.* Refining an algorithm-powered just-in-time adaptive weight control intervention: A randomized controlled trial evaluating model performance and behavioral outcomes. *Health Informatics Journal.* **26**, 2315-2331, doi:10.1177/1460458220902330 (2020).

47 Goldstein, S. P., Brick, L. A., Thomas, J. G. & Forman, E. M. Examination of the relationship between lapses and weight loss in a smartphone-based just-in time adaptive intervention. *Translational Behavioral Medicine.* **11**, 993-1005, doi:10.1093/tbm/ibaa097 (2021).

48 Goldstein, S. P. *et al.* Optimizing a Just-in-Time Adaptive Intervention to Improve Dietary Adherence in Behavioral Obesity Treatment: Protocol for a Microrandomized Trial. *JMIR Res Protoc.* **10**, e33568, doi:10.2196/33568 (2021).

49 Gatwood, J. *et al.* The Management of Diabetes in Everyday Life (MODEL) program: development of a tailored text message intervention to improve diabetes self-care activities among underserved African-American adults. *Transl Behav Med.* **10**, 204-212, doi: 10.1093/tbm/ibz024 (2020).

50 Golbus, J. R. *et al.* A Physical Activity and Diet Just-in-Time Adaptive Intervention to Reduce Blood Pressure: The myBPmyLife Study Rationale and Design. *J Am Heart Assoc.* **13**, e031234, doi: 10.1161/jaha.123.031234 (2024).

51 Hellem, A. K. *et al.* A Community Participatory Approach to Creating Contextually Tailored mHealth Notifications: myBPmyLife Project. *Health Promot Pract.* 15248399221141687, doi:10.1177/15248399221141687 (2023).

52 Gupta, S., Sood, S., Management, I. D. S. & Bharati Vidyapeeths Inst Comp, A. *Context Aware Mobile Agent for Reducing Stress and Obesity by Motivating Physical Activity: A Design Approach*. (2015).

53 Hamborg, T. G., Tang, L. H., Andersen, R. M., Skou, S. T. & Simonÿ, C. It is like someone holding your hand when you need it - lived experiences of patients with cardiovascular disease participating in a digital health intervention focusing on the maintenance of physical activity after cardiac rehabilitation. *Disabil Rehabil Assist Technol.* **19**, 1718-1728, doi: 10.1080/17483107.2023.2228839 (2024).

54 Andersen, R. M. *et al.* Maintenance of physical activity after cardiac rehabilitation (FAIR): study protocol for a feasibility trial. *BMJ Open.* **12**, e060157, doi: 10.1136/bmjopen-2021-060157 (2022).

55 Hemnes, A. R. *et al.* A Mobile Health Intervention to Increase Physical Activity in Pulmonary Arterial Hypertension. *Chest.* **160**, 1042-1052, doi:10.1016/j.chest.2021.04.012 (2021).

56 Martin, S. S. *et al.* mActive: A Randomized Clinical Trial of an Automated mHealth Intervention for Physical Activity Promotion. *J Am Heart Assoc.* **4**, doi: 10.1161/jaha.115.002239 (2015).

57 Hietbrink, E. A. G. *et al.* A Digital Lifestyle Coach (E-Supporter 1.0) to Support People With Type 2 Diabetes: Participatory Development Study. *JMIR Hum Factors*. **10**, e40017, doi:10.2196/40017 (2023).

58 Hietbrink, E. A. G. *et al.* A Digital Coach (E-Supporter 1.0) to Support Physical Activity and a Healthy Diet in People With Type 2 Diabetes: Acceptability and Limited Efficacy Testing. *Jmir Formative Research*. **7**, doi: 10.2196/45294 (2023).

59 Hurley, J. C. *et al.* The Walking Interventions Through Texting (WalkIT) Trial: Rationale, Design, and Protocol for a Factorial Randomized Controlled Trial of Adaptive Interventions for Overweight and Obese, Inactive Adults. *JMIR Res Protoc*. **4**, e108, doi:10.2196/resprot.4856 (2015).

60 Khunti, K. *et al.* Promoting physical activity in a multi-ethnic population at high risk of diabetes: the 48-month PROPELS randomised controlled trial. *Bmc Medicine*. **19**, doi:10.1186/s12916-021-01997-4 (2021).

61 Morton, K. *et al.* A Text-Messaging and Pedometer Program to Promote Physical Activity in People at High Risk of Type 2 Diabetes: The Development of the PROPELS Follow-On Support Program. *JMIR Mhealth Uhealth*. **3**, e105, doi:10.2196/mhealth.5026 (2015).

62 Yates, T. *et al.* PRomotion Of Physical activity through structured Education with differing Levels of ongoing Support for people at high risk of type 2 diabetes (PROPELS): study protocol for a randomized controlled trial. *Trials*. **16**, doi:10.1186/s13063-015-0813-z (2015).

63 Kim, M., Lee, H., Park, G. & Khang, A. R. Participation experience in self-care program for type 2 diabetes: A mixed-methods study. *Journal of Korean Gerontological Nursing*. **26**, 31-42, doi: 10.17079/jkgn.2023.00220 (2024).

64 Park, G. *et al.* Automated Personalized Self-care Program for Patients With Type 2 Diabetes Mellitus: A Pilot Trial. *Asian Nursing Research*. **18**, 114-124, doi: 10.1016/j.anr.2024.04.003 (2024).

65 Klein, M., Mogles, N. & van Wissen, A. Intelligent mobile support for therapy adherence and behavior change. *Journal of Biomedical Informatics*. **51**, 137-151, doi:10.1016/j.jbi.2014.05.005 (2014).

66 Korinek, E. V. *et al.* Adaptive step goals and rewards: a longitudinal growth model of daily steps for a smartphone-based walking intervention. *Journal of Behavioral Medicine*. **41**, 74-86, doi:10.1007/s10865-017-9878-3 (2018).

67 Leitner, J., Chiang, P. H., Khan, B., Dey, S. & Ieee, I. C. S. *An mHealth Lifestyle Intervention Service for Improving Blood Pressure using Machine Learning and IoMTs*. (2022).

68 Lim, S. *et al.* Multifactorial intervention in diabetes care using real-time monitoring and tailored feedback in type 2 diabetes. *Acta Diabetologica*. **53**, 189-198, doi:10.1007/s00592-015-0754-8 (2016).

69 Lin, M. *et al.* Tailored, Interactive Text Messages for Enhancing Weight Loss Among African American Adults: The TRIMM Randomized Controlled Trial. *American Journal of Medicine*. **128**, 896-904, doi:10.1016/j.amjmed.2015.03.013 (2015).

70 Mansour-Assi, S. J. *et al.* Social Mobile Approaches to Reducing Weight (SMART) 2.0: protocol of a randomized controlled trial among young adults in university settings. *Trials*. **23**, doi:10.1186/s13063-021-05938-7 (2022).

71 Martinho, D. *et al.* An Architecture for a Coaching System to Support Type 2 Diabetic Patients. *Lecture Notes in Networks and Systems*. **603 LNNS**, 167-178, doi: 10.1007/978-3-031-22356-3_16 (2023).

72 Pinto, A. *et al.* *Improving the lifestyle behavior of type 2 diabetes mellitus patients using a mobile application*. Vol. 2022 (Institute of Electrical and Electronics Engineers Inc., 2022).

73 Miller, H. N. *et al.* The Nourish Protocol: A digital health randomized controlled trial to promote the DASH eating pattern among adults with hypertension. *Contemporary Clinical Trials*. **109**, doi:10.1016/j.cct.2021.106539 (2021).

74 Nezami, B. T., Hurley, L., Power, J., Valle, C. G. & Tate, D. F. A pilot randomized trial of simplified versus standard calorie dietary self-monitoring in a mobile weight loss intervention. *Obesity*. **30**, 628-638, doi:10.1002/oby.23377 (2022).

75 Novak, J. *et al.* Participatory development of an mHealth intervention delivered in general practice to increase physical activity and reduce sedentary behaviour of patients with prediabetes and type 2 diabetes (ENERGISED). *Bmc Public Health*. **24**, doi: 10.1186/s12889-024-18384-2 (2024).

76 Vetrovsky, T. *et al.* mHealth intervention delivered in general practice to increase physical activity and reduce sedentary behaviour of patients with prediabetes and type 2 diabetes (ENERGISED): rationale and study protocol for a pragmatic randomised controlled trial. *BMC Public Health*. **23**, 613, doi: 10.1186/s12889-023-15513-1 (2023).

77 Pardos, A., Gallos, P., Menychtas, A., Panagopoulos, C. & Maglogiannis, I. Enriching Remote Monitoring and Care Platforms with Personalized Recommendations to Enhance Gamification and Coaching. *Stud Health Technol Inform*. **302**, 332-336, doi: 10.3233/shti230129 (2023).

78 Park, C. *et al.* A multiple technology-based and individually-tailored Sit Less program for people with cardiovascular disease: A randomized controlled trial study protocol. *Plos One*. **19**, e0302582, doi: 10.1371/journal.pone.0302582 (2024).

79 Pellegrini, C. A. *et al.* Acceptability of smartphone technology to interrupt sedentary time in adults with diabetes. *Transl Behav Med*. **5**, 307-314, doi: 10.1007/s13142-015-0314-3 (2015).

80 Pimenta, N., Felix, I. B., Monteiro, D., Marques, M. M. & Guerreiro, M. P. Promoting Physical Activity in Older Adults With Type 2 Diabetes via an Anthropomorphic Conversational Agent: Development of an Evidence and Theory-Based Multi-Behavior Intervention. *Frontiers in Psychology*. **13**, doi:10.3389/fpsyg.2022.883354 (2022).

81 Plaete, J., De Bourdeaudhuij, I., Verloigne, M., Oenema, A. & Crombez, G. A Self-Regulation eHealth Intervention to Increase Healthy Behavior Through General Practice: Protocol and Systematic Development. *JMIR Res Protoc*. **4**, e141, doi: 10.2196/resprot.4835 (2015).

82 Poppe, L. *et al.* Users' thoughts and opinions about a self-regulation-based eHealth intervention targeting physical activity and the intake of fruit and vegetables: A qualitative study. *PloS One*. **12**, doi:10.1371/journal.pone.0190020 (2017).

83 Poppe, L. *et al.* Experiences and Opinions of Adults with Type 2 Diabetes Regarding a Self-Regulation-Based eHealth Intervention Targeting Physical Activity and Sedentary Behaviour. *International Journal of Environmental Research and Public Health.* **15**, doi:10.3390/ijerph15050954 (2018).
84 Poppe, L. *et al.* A Self-Regulation-Based eHealth and mHealth Intervention for an Active Lifestyle in Adults With Type 2 Diabetes: Protocol for a Randomized Controlled Trial. *JMIR Res Protoc.* **8**, e12413, doi: 10.2196/12413 (2019).
85 Poppe, L. *et al.* Efficacy of a Self-Regulation-Based Electronic and Mobile Health Intervention Targeting an Active Lifestyle in Adults Having Type 2 Diabetes and in Adults Aged 50 Years or Older: Two Randomized Controlled Trials. *J Med Internet Res.* **21**, e13363, doi: 10.2196/13363 (2019).
86 Radhakrishnan, K. *et al.* Usability Testing of a Sensor-Controlled Digital Game to Engage Older Adults with Heart Failure in Physical Activity and Weight Monitoring. *Appl Clin Inform.* **11**, 873-881, doi: 10.1055/s-0040-1721399 (2020).
87 Radhakrishnan, K. *et al.* Feasibility of a Sensor-Controlled Digital Game for Heart Failure Self-management: Randomized Controlled Trial. *JMIR Serious Games.* **9**, e29044, doi:10.2196/29044 (2021).
88 Reinwand, D., Kuhlmann, T., Wienert, J., de Vries, H. & Lippke, S. Designing a theory- and evidence-based tailored eHealth rehabilitation aftercare program in Germany and the Netherlands: study protocol. *BMC Public Health.* **13**, doi:10.1186/1471-2458-13-1081 (2013).
89 Storm, V. *et al.* Effectiveness of a Web-Based Computer-Tailored Multiple-Lifestyle Intervention for People Interested in Reducing their Cardiovascular Risk: A Randomized Controlled Trial. *J Med Internet Res.* **18**, e78, doi:10.2196/jmir.5147 (2016).
90 Richardson, C. R. *et al.* A randomized trial comparing structured and lifestyle goals in an internet-mediated walking program for people with type 2 diabetes. *Int J Behav Nutr Phys Act.* **4**, 59, doi: 10.1186/1479-5868-4-59 (2007).
91 Richardson, C. R. *et al.* An Online Community Improves Adherence in an Internet-Mediated Walking Program. Part 1: Results of a Randomized Controlled Trial. *Journal of Medical Internet Research.* **12**, 138-153, doi:10.2196/jmir.1338 (2010).
92 Schoenthaler, A. *et al.* Investigation of a Mobile Health Texting Tool for Embedding Patient-Reported Data Into Diabetes Management (i-Matter): Development and Usability Study. *Jmir Formative Research.* **4**, doi:10.2196/18554 (2020).
93 Schultz, C. L., Bocarro, J. N., Hipp, J. A., Bennett, G. J. & Floyd, M. F. Prescribing Time in Nature for Human Health and Well-Being: Study Protocol for Tailored Park Prescriptions. *Front Digit Health.* **4**, 932533, doi:10.3389/fdgth.2022.932533 (2022).
94 Shibuta, T. *et al.* Preliminary Efficacy, Feasibility, and Perceived Usefulness of a Smartphone-Based Self-Management System With Personalized Goal Setting and Feedback to Increase Step Count Among Workers With High Blood Pressure: Before-and-After Study. *JMIR Cardio.* **7**, e43940, doi: 10.2196/43940 (2023).
95 Spruijt-Metz, D. *et al.* Advancing Behavioral Intervention and Theory Development for Mobile Health: The HeartSteps II Protocol. *Int J Environ Res Public Health.* **19**, doi:10.3390/ijerph19042267 (2022).

96 Stein, R. I. *et al.* Design of a randomized trial testing a multi-level weight-control intervention to reduce obesity and related health conditions in low-income workers. *Contemporary Clinical Trials*. **79**, 89-97, doi:10.1016/j.cct.2019.01.011 (2019).
97 Tabak, R. G. *et al.* Development of a scalable weight loss intervention for low-income workers through adaptation of interactive obesity treatment approach (iOTA). *BMC Public Health*. **18**, 1265, doi: 10.1186/s12889-018-6176-0 (2018).
98 Steinberg, D. M. *et al.* Feasibility of a Digital Health Intervention to Improve Diet Quality Among Women With High Blood Pressure: Randomized Controlled Feasibility Trial. *JMIR mHealth and uHealth*. **8**, doi:10.2196/17536 (2020).
99 Sun, J. *et al. BeActive: Encouraging Physical Activities with Just-in-time Health Intervention and Micro Financial Incentives*. (Association for Computing Machinery, 2020).
100 Sze, W. T. *et al.* StepAdd: A personalized mHealth intervention based on social cognitive theory to increase physical activity among type 2 diabetes patients. *Journal of Biomedical Informatics*. **145**, doi: 10.1016/j.jbi.2023.104481 (2023).
101 Waki, K. *et al.* Efficacy of StepAdd, a Personalized mHealth Intervention Based on Social Cognitive Theory to Increase Physical Activity Among Patients With Type 2 Diabetes Mellitus: Protocol for a Randomized Controlled Trial. *Jmir Research Protocols*. **13**, doi: 10.2196/53514 (2024).
102 Tabak, M. *et al. ACCEPTANCE AND USABILITY OF AN AMBULANT ACTIVITY COACH FOR PATIENTS WITH COPD*. (2013).
103 Tabak, M., Op den Akker, H. & Hermens, H. Motivational cues as real-time feedback for changing daily activity behavior of patients with COPD. *Patient Education and Counseling*. **94**, 372-378, doi:10.1016/j.pec.2013.10.014 (2014).
104 Tabak, M., Brusse-Keizer, M., van der Valk, P., Hermens, H. & Vollenbroek-Hutten, M. A telehealth program for self-management of COPD exacerbations and promotion of an active lifestyle: a pilot randomized controlled trial. *International Journal of Chronic Obstructive Pulmonary Disease*. **9**, 935-944, doi:10.2147/copd.S60179 (2014).
105 Tabak, M., Op den Akker, H., Vollenbroek-Hutten, M. M. R. & Hermens, H. J. Improving long-term activity behaviour of individual patients with COPD using an ambulant activity coach. *Centre for Telematics and Information Technology*. 97-118 (2014).
106 Wieringa, W., Akker, H. O. D., Jones, V. M., Akker, R. O. D. & Hermens, H. J. *Ontology-based generation of dynamic feedback on physical activity*. (Springer-Verlag, 2011).
107 Tamura, K. *et al.* Multilevel mobile health approach to improve cardiovascular health in resource-limited communities with Step It Up: a randomised controlled trial protocol targeting physical activity. *BMJ Open*. **10**, doi:10.1136/bmjopen-2020-040702 (2020).
108 van der Weegen, S. *et al.* The Development of a Mobile Monitoring and Feedback Tool to Stimulate Physical Activity of People With a Chronic Disease in Primary Care: A User-Centered Design. *JMIR mHealth and uHealth*. **1**, doi:10.2196/mhealth.2526 (2013).
109 van der Weegen, S. *et al.* It's LiFe! Mobile and Web-Based Monitoring and Feedback Tool Embedded in Primary Care Increases Physical Activity: A Cluster Randomized Controlled Trial. *Journal of Medical Internet Research*. **17**, doi:10.2196/jmir.4579 (2015).

110 Verwey, R. *et al.* A monitoring and feedback tool embedded in a counselling protocol to increase physical activity of patients with COPD or type 2 diabetes in primary care: study protocol of a three-arm cluster randomised controlled trial. *Bmc Family Practice.* **15**, doi:10.1186/1471-2296-15-93 (2014).
111 Verwey, R. *et al.* A pilot study of a tool to stimulate physical activity in patients with COPD or type 2 diabetes in primary care. *J Telemed Telecare.* **20**, 29-34, doi: 10.1177/1357633x13519057 (2014).
112 van Genugten, L., van Empelen, P., Flink, I. & Oenema, A. Systematic development of a self-regulation weight-management intervention for overweight adults. *BMC Public Health.* **10**, 649, doi: 10.1186/1471-2458-10-649 (2010).
113 van Genugten, L. *et al.* Results from an online computer-tailored weight management intervention for overweight adults: randomized controlled trial. *J Med Internet Res.* **14**, e44, doi: 10.2196/jmir.1901 (2012).
114 van Genugten, L., van Empelen, P. & Oenema, A. Intervention use and action planning in a web-based computer-tailored weight management program for overweight adults: randomized controlled trial. *JMIR Res Protoc.* **3**, e31, doi:10.2196/resprot.2599 (2014).
115 Watson, A., Bickmore, T., Cange, A., Kulshreshtha, A. & Kvedar, J. An Internet-Based Virtual Coach to Promote Physical Activity Adherence in Overweight Adults: Randomized Controlled Trial. *Journal of Medical Internet Research.* **14**, doi:10.2196/jmir.1629 (2012).
116 Yom-Tov, E. *et al.* Encouraging Physical Activity in Patients With Diabetes: Intervention Using a Reinforcement Learning System. *Journal of Medical Internet Research.* **19**, doi:10.2196/jmir.7994 (2017).
117 Zahedani, A. D. *et al.* Digital health application integrating wearable data and behavioral patterns improves metabolic health. *NPJ Digit Med.* **6**, 216, doi: 10.1038/s41746-023-00956-y (2023).
