# Supplementary material for: Systematic review of dynamically tailored eHealth interventions targeting physical activity and healthy diet in chronic disease
Source: NPJ Digit Med. 2025 Nov 19;8:696. doi: 10.1038/s41746-025-02054-7 (PMC12630729; doi:10.1038/s41746-025-02054-7)
Supplement: Supplementary file 3 — Supplementary data2 [file 41746_2025_2054_MOESM3_ESM.pdf]

Supplementary Data 2. Tailoring Strategy

Author (year)	Behavioral goal-setting	Dynamic tailoring variables	Static tailoring variables	Types of data used	Types of self-monitoring devices
**Aguilera (2020)**	• Guided goal-setting<br>• Personalized<br>• Not reported	• Steps/step goal achievement day before<br>• Timing and frequency of previous messages	• Clinical data<br>• Demographic data (such as age, gender language and PHQ-8 scores)	**For dynamic variables:**<br>• Accelerometer/pedometer data<br>**For static variables:**<br>• Questionnaire	**Connected for tailoring:**<br>• Pooling from Google Fit<br>• Apple HealthKit<br>• Built-in phone pedometer
**Almeida (2015) Estabrooks (2011)**	• Guided goal-setting<br>• Personalized<br>• Adaptive	• Physical activity goals<br>• Mediators of behavior change ("Response & self-efficacy" and "severity and vulnerability" scores)	• Physical environment (home, work, and PA facilities)<br>• Reported barriers for PA	**For dynamic variables:**<br>• Interactive Voice Response (IVR)<br>**For static variables:**<br>• Geographic information system (GIS)<br>• Interactive computer sessions	• Not applicable
**Alos (2022)**	• Automated goal-setting<br>• Personalized<br>• Adaptive	• Sedentary behavior time (sitting time is prolonged for more than 1 hour and achievement of individualized goals)<br>• Steps (achievement of individualized goals)	• Not reported	**For dynamic variables:**<br>• Accelerometer/pedometer data	**Connected for tailoring:**<br>• Activity tracker (MetaWearC external sensor)
**Al-Ozari (2018)**	• No goal-setting	• Steps (optimal (e.g. 10k steps a day), sub-optimal (e.g. 3k steps/day) and low levels (< 3k steps/day))<br>• Cravings<br>• Need for help<br>• Lapse	• Personal goals (motivators) for better diabetes control	**For dynamic variables:**<br>• Accelerometer/pedometer data<br>• User-initiated self-reporting<br>**For static variables:**<br>• Not reported	**Connected for tailoring:**<br>• Activity tracker (wristband pedometer)

**Ambeba (2015)**<br>**Bizhanova (2023)**<br>**Burke (2017, 2020, 2022a, 2022b)**<br>**Cheng (2023)**<br>**Kariuki (2023)**<br>**Wang (2012)**	• Automated goal-setting<br>• Personalized and generic<br>• Static and adaptive	• Dietary intake (calories (% of goal), fat (% of goal), sugar (g))<br>• Physical activity (number of steps in the previous week, number of active minutes in the last week)<br>• Weight: whether self-weighing occurred and based on the amount and rate of weight loss, maintenance, or gain	• Not reported	**For dynamic variables:**<br>• Accelerometer/pedometer data<br>• Device-measured vital signs/ body parameters<br>• User-initiated self-reporting	**Connected for tailoring:**<br>• Activity tracker (Fitbit Charge 2)<br>• Scale (not further specified)
**Baert (2018)**<br>**Bohanec (2021)**<br>**Clays (2021)**<br>**Voorend (2019)**	• Guided goal-setting<br>• Personalized<br>• Adaptive	<u>Individualized exercise program:</u><br>• Week in the program<br>• Current frequency<br>• The possible physician's and patient's suggestions for the change<br>• Adherence to the program (normative vs. current)<br>• Meeting intensity criteria<br><u>Daily exercise management:</u><br>• Blood pressure<br>• Heartrate<br>• Overall feeling<br><u>Nutrition:</u><br>• Scores on the nutrition questionnaire	• Patient's physical capacity<br>• BMI<br>• Prescribed amount of liquid intake<br>• Diabetes diagnosis	**For dynamic variables:**<br>• Accelerometer/pedometer data<br>• Device-measured vital signs<br>• EMA<br>• Questionnaire<br>**For static variables:**<br>• Cardiopulmonary exercise (cycle ergometry) test	**Connected for tailoring:**<br>• Activity tracker (custom wristband sensor)<br>**Not connected for tailoring:**<br>• A digital bathroom scale (ADE, Model Silje BE1303)<br>• Upper arm blood pressure monitor (A&D Medical, Model Number UA-611)<br>• A pill box organizer (PuTwo, 7-Day AM/PM Night Reminder Medi-Planner)
**Beckie (2024)**<br>**Sengupta (2020a, 2020b)**	• Self-set goals<br>• Personalized<br>• Adaptive	• Number of minutes walked/walking goal<br>• Current activity<br>• Mood<br>• Location<br>• Eating episodes<br>• Social context	• Gender	**For dynamic variables:**<br>• Accelerometer/pedometer data<br>• Daily EMAs	**Connected for tailoring:**<br>• Smart watch (Moto 360 2nd Gen, Android Wear OS 2.0)

**Bennett (2013, 2018)**<br>**Foley (2012, 2016)**<br>**Steinberg (2013)**	• Automated goal-setting<br>• Personalized<br>• Adaptive	• 4 obesogenic behavior change goals (a.o. physical activity and diet goals, goals changing over time)<br>• Weight (weight goal progress)	• Not reported	**For dynamic variables:**<br>• System-initiated self-reporting (SMS prompts)<br>• Interactive Voice Response (IVR).	**Not connected for tailoring:**<br>• Activity tracker (Yamax SW-650/651 Digi-Walker)<br>• Weighing scale (BodyTrace)
**Boh (2016)**	• Self-set goal<br>• Personalize<br>• Not reported	<u>Feedback module:</u><br>• Food desire strength<br>• Specific food desires<br>• Emotions<br>• Location<br>• Social company<br>• Activities<br><u>Cognitive module:</u><br>• Neutral cognitions (including description of an eating event, hunger, desire and taste, energy needed)<br>• Functional cognitions (including healthy intention, successful control)<br>• Dysfunctional cognitions (including negative emotions, positive, emotions, social activities and pressure, reward, control failure, other dysfunctional cognitions)	• Not reported	**For dynamic variables:**<br>• Ecological Momentary Assessment<br>• User-initiated self-reporting	• Not applicable

**Bond (2014)**<br>**Thomas (2015)**	• Automated goal-setting<br>• Generic<br>• Static	• Sedentary behavior time (sedentary behavior: ≤1.5 METs; dependent on intervention condition: (1) 3-min walking break after 30 continuous sedentary minutes, (2) 6-min walking break after 60 continuous sedentary minutes, and (3) 12-min walking break after 120 continuous sedentary minutes)	• Not reported	**For dynamic variables:**<br>• Accelerometer/pedometer data (from smartphone)	• Not applicable
**Boudreau (2016)**<br>**Moreau (2015)**	• Self-set goal<br>• Personalize<br>• Adaptive	• Physical activity behavior<br>• Intention<br>• Importance ruler<br>• Confidence ruler	• Gender<br>• Name<br>• Attitude<br>• Self-efficacy<br>• Type of motivation	**For dynamic and static variables:**<br>• Questionnaire (assessment of PA behavior and behavioral constructs)	• Not applicable
**Buchan (2020)**	• Goal setting without specification of method<br>• Personalized<br>• Adaptive	<u>Progress towards daily goal:</u><br>• Steps (progress to goal)<br>• Calories burned (progress to goal)<br><u>Personalized exercise program:</u><br>• Activity/heartrate<br>• Self-reported weight input data	• Weight loss goal<br>• Target date of achievement<br>• Fitness level	**For dynamic variables:**<br>• Accelerometer/pedometer data<br>• Device-measured vital signs<br>• User-initiated self-reporting<br>**For static variables:**<br>• Not reported	**Connected for tailoring:**<br>• Activity tracker + heartrate clip (Onitor Track)
**Chokshi (2017)**	• Automated goal-setting<br>• Personalized<br>Adaptive	• Steps (achievement of step goal)	• Not reported	**For dynamic variables:**<br>• Accelerometer/pedometer data	**Connected for tailoring:**<br>• Activity tracker. Misfit Shine
**Collins (2010, 2012, 2013)**	• Goal setting without specification of method<br>• Personalized<br>• Adaptive	• Physical activity behavior (not specified)<br>• Eating behavior (not specified)<br>• Self-monitored weight	• Not reported	**For dynamic variables:**<br>• System-initiated self-reporting (e-mail or text message reminders to use the online diary and enter weight)	• Not applicable

**Daryabeygi-Khotbehsara (2022, 2023)**	• Self-set goals<br>• Personalized<br>• Adaptive	• Sedentary behavior time (seated for 1 hour and goal)<br>• Physical activity (goal)<br>• Location (home or workplace)<br>• Weather (good or suitable for PA, rainy and cold or not suitable for PA)<br>• Day of the week (weekend vs week days)<br>• Time of day	• Not reported	**For dynamic variables:**<br>• Accelerometer/pedometer data<br>• Smartphone's GPS<br>• OpenWeather representational state transfer (REST) API<br>• Android LocalTime API	**Connected for tailoring:**<br>• Activity tracker (SORD wearable sensor)
**Dorsch (2018, 2020)**	• Goal setting without specification of method<br>• Not reported<br>• Not reported	• Location (entrance of grocery store or restaurant)	• User's confidence in following a low-sodium diet<br>• Top 5 high sodium-containing foods	**For dynamic variables:**<br>• Geofencing with mobile phones sensors (including Wi-Fi, Bluetooth, accelerometer, gyroscope, magnetometer, global positioning system)<br>**For static variables:**<br>• Survey<br>• Block Sodium Screener	• Not applicable
**Evans (2015)**	• Guided goal-setting<br>• Personalized<br>• Adaptive	• Eating behavior (achievement of goals)<br>• Steps (achievement of goals)<br>• Weight	• Not reported	**For dynamic variables:**<br>• System-initiated self reporting (SMS prompts)	**Not connected for tailoring:**<br>• Activity tracker (Omron III Walking Style pedometer)<br>• Smart scale (SIM-enabled scale)
**Finkelstein (2015)**	• Automated goal-setting<br>• Generic<br>• Static	• Steps (when the person walked less than 15 steps in the past hour)<br>• Time of day	• Not reported	**For dynamic variables:**<br>• Accelerometer/pedometer data	**Connected for tailoring:**<br>• Activity tracker (Fitbit One)

**Forman (2019, 2019)**<br>**Goldstein (2017, 2020, 2021a, 2021b)**	• Automated goal-setting<br>• Generic<br>• Static	• Affect<br>• Boredom<br>• Hunger<br>• Cravings<br>• Tiredness<br>• Unhealthy food availability<br>• Temptations<br>• Missed meals/snacks<br>• Self-efficacy<br>• Motivation<br>• Socializing<br>• TV watching<br>• Negative interpersonal interactions<br>• Healthy food presence<br>• Cognitive load<br>• Food cues<br>• Hours of sleep<br>• Exercise<br>• Alcohol consumption<br>• Planning food intake<br>• Time of day	• Not reported	**For dynamic variables:**<br>• Ecological Momentary Assessment (EMA)	• Not applicable
**Gatwood (2020)**	• Self-set goals<br>• Personalized<br>• Adaptive	• Self-reported behavior (corresponding to diet, exercise, and medication use)<br>• Patient preferences for (1) focus (healthy eating, physical activity, or medication adherence), (2) frame (purely educational, motivational, or focused on a particular goal), (3) frequency (twice daily, once a day, or once every other day); time of day	• Age<br>• Gender<br>• Ethnicity (African-American adults)<br>• Competence<br>• Quality of life<br>• Adherence<br>• Self-regulation	**For dynamic variables:**<br>• System-initiated self-reporting (self-reported responses via text-based bi-directional messaging)<br>**For static variables:**<br>• Questionnaires (a.o. Summary of Diabetes Self-Care Activities measure, Diabetes-39 quality of life instrument, the Adherence to Refills and Medications Scale)	• Not applicable

**Golbus (2024)**<br>**Hellem (2023)**	• Self-set goals<br>• Personalized<br>• Adaptive	• Weather<br>• Time of day<br>• Day of the week (weekend or week day or grocery day)	• Community<br>• Level of mobility<br>• Confidence in selecting lower-sodium food choices	**For dynamic variables:**<br>• Not reported<br>**For static variables:**<br>• Single-item question ('Are you able to walk for 10 minutes without restriction?')	**Not connected for tailoring:**<br>• Smart watch (Fitbit versa 2)<br>• Wireless BP monitor (Omron Evolv BP7000)
**Gupta (2015)**	• No goal-setting	• Location (home, office, outside)<br>• Weather (ideal or adverse)<br>• Time of day (acceptable and unacceptable)	• Not reported	**For dynamic variables:**<br>• Google Fused Location API which uses either GPS or WiFi network<br>• Open Weather API<br>• Time on the user's mobile device	**Not connected for tailoring:**<br>• Activity tracking with Google Fused Location API
**Hamborg (2024)**<br>**Martens**<br>**Anderson (2022)**	• Guided goal-setting<br>• Personalize<br>• Adaptive	• Physical activity behavior (achievement of physical activity action plans)	• Not reported	**For dynamic variables:**<br>• System-initiated self reporting (SMS prompts)	• Not applicable
**Hemnes (2021)**<br>**Martin (2015)**	• Automated goal-setting<br>• Personalized<br>• Adaptive	• Steps (progress toward daily step count target)	• Preferred name<br>• Age<br>• Gender<br>• Physician name<br>• Preferred activity<br>• Favorite athlete<br>• Cardiac risk factors<br>• Occupation<br>• Spouse's name<br>• Children's name<br>• Dog's name<br>• Name local park<br>• Name gym<br>• Work schedule<br>• Television watching habits	**For dynamic variables:**<br>• Accelerometer/pedometer data<br>**For static variables:**<br>• Questionnaire	**Connected for tailoring:**<br>• Activity tracker (Fitbit Charge HR)

**Hietbrink (2023a, 2023b)**	• Guided goal-setting<br>• Personalized<br>• Adaptive	• Duration of intervention use (after X days the user progresses to another phase of behavioral change (intention, action, maintenance))<br>• Time of day (morning, afternoon or evening message)<br>• Type of behavioral goal (physical activity or nutrition)<br>• Goal achievement (yes or no)<br>• Identified barriers for goal achievement (motivation, self-efficacy, mood, stress, planning)	• Type of chronic disease (type 2 diabetes)	**For dynamic variables:**<br>• Calendar after first login<br>• Smartphone clock<br>• Accelerometer/pedometer data<br>• EMA<br>**For static variables:**<br>• Not reported	**Connected for tailoring:**<br>• Activity tracker (Fitbit activity tracker)<br>**Not connected for tailoring:**<br>• Continuous/Flash glucose monitoring (Freestyle Libre 2)
**Hurley (2015)**	• Automated goal-setting<br>• Personalized<br>• Adaptive	• Steps (achievement of step goal)	• Not reported	**For dynamic variables:**<br>• User-initiated self-reporting (via SMS)	**Not connected for tailoring:**<br>• Activity tracker (Fitbit Zip)
**Khunti (2021)<br>Morton (2015)<br>Yates (2015)**	• Guided goal-setting<br>• Personalized<br>• Adaptive	• Step goal achievement (on short and long term)<br>• Barriers for goal achievement (ill/health/injury, energy/motivation, time, other)	• Confidence in increasing PA<br>• Previous experience with PA<br>• Potential mobility issues.	**For dynamic variables:**<br>• System-initiated self-reporting (text prompts)<br>**For static variables:**<br>• Telephone-administered assessment	**Not connected for tailoring:**<br>• Activity tracker (Yamax SW200 pedometer)
**Kim (2024)<br>Park (2024)**	• Automated goal-setting<br>• Generic<br>• Adaptive	• Self-care behaviors (highest and lowest goal achievement rates of self-care behaviors on the previous day)	• Not reported	**For dynamic variables:**<br>• System-initiated self reporting (in-app activity monitoring features and food photo diary; monitoring reminder daily at 8 PM)	• Not applicable

**Klein (2014)**	• Goal setting without specification of method<br>• Personalized<br>• Not reported	• Food intake behavior (level of adherence to drinking fluids, eating meat or fish, eating vegetables, eating starchy products, eating fruit and eating snacks)<br>• Physical activity behavior (level of adherence to PA minutes goals based on walking, cycling, sports)<br>• Medication intake behavior (level of adherence)<br>• Stage of behavior change	• Barriers to adherence to physical activity, diet or medication intake	**For dynamic variables:**<br>• Graphical question on the mobile phone<br>• Web-based calendar and EMA/mobile phone questions<br>• Opening of an electronic pill box<br>• Questionnaires and self-monitoring answers for PA and NU<br>**For static variables:**<br>• Intake survey	**Connected for tailoring:**<br>• Electronic pill box
**Korinek (2018)**	• Automated goal-setting<br>• Personalized<br>• Adaptive	• Steps (achievement of step goal)	• Not reported	**For dynamic variables:**<br>• Accelerometer/pedometer data	**Connected for tailoring:**<br>• Activity tracker (Fitbit Zip)
**Leitner (2022)**	• No goal-setting	• Physiological parameters (blood pressure, heartrate)<br>• Physical activity behavior (steps, floors, walking/running speed, sedentary time, lightly active time, very active time)<br>• Sleep (sleep duration, bed time, wake up time, light sleep, deep sleep, REM sleep, sleep awareness)<br>• Dietary choices (alcohol intake, red meat intake, servings of fruit, serving of vegetables, salt intake)<br>• Stress<br>• Mood<br>• Time of day	• Age<br>• Gender	**For dynamic variables:**<br>• Heartrate monitor, accelerometer, ambient light sensor and barometer (in smartwatch)<br>• Device-measured vital signs<br>• Questionnaire(s)<br>**For static variables:**<br>• Not reported	**Connected for tailoring:**<br>• Smart watch (Samsung Galaxy Watch)<br>• Wireless BP monito (Omron Evolv wireless BP monitor)

**Lim (2016)**	• No goal-setting	• Blood glucose levels (fasting, postprandial, bedtime)<br>• Physical activity levels (time, calorie expenditure)<br>• Dietary intake (total caloric intake, macronutrient composition)	• Not reported	**For dynamic variables:**<br>• Accelerometer/pedometer data<br>• Device-measured vital signs/parameters<br>• User-initiated self-reporting (self-reported via a u-healthcare website, processed by CAN Pro 3.0 software)	**Connected for tailoring:**<br>• Activity tracker (H3 System Co. Ltd.)<br>• PSTN-connected glucometer (GlucActiviyoDr Plus AGM-3000B-Bluetooth HDP version meter)
**Lin (2015)**	• Guided goal-setting<br>• Generic<br>• Adaptive	• Health behaviors related to the current topic<br>• Current weight<br>• Motivation level<br>• Time of day (participant's wake, lunch, and sleep times)	• 3 self-selected TRIMM goals related to physical activity and diet (increase moving in daily life, increase exercise, better portion control, more balanced diet, reduce fat intake, reduce sugar intake, eat only when hungry)	**For dynamic variables:**<br>• System-initiated self-reporting (text prompts)<br>**For static variables:**<br>• Selection from a list of 8 behavioral goals	**Not connected for tailoring:**<br>• Activity tracker (pedometer)
**Mansour-Assi (2022)**	• Automated goal-setting<br>• Generic<br>• Adaptive	• Physical activity (achievement of MVPA goals)<br>• Diet (achievement of energy intake goals)<br>• Sleep (achievement of sleep duration goals)<br>• Weight loss progress	• Not reported	**For dynamic variables:**<br>• Accelerometer/pedometer data<br>• Device-measured vital signs<br>• User-initiated self-reporting (logging via the Fitbit app)<br>• Weighing scale data	**Connected for tailoring:**<br>• Activity tracker (Fitbit Charge 3 or 4)<br>• Smart weighing scale (Aria 2 scale/Aria Air scale)
**Martinho (2023) Pinto (2022)**	• Automated goal-setting<br>• Generic<br>• Static	• Eating behavior (carbohydrates, proteins, fats, fiber, salt and some micronutrients)<br>• Phase of behavior change (precontemplation/ contemplation, preparation, action and maintenance)	• Not reported	**For dynamic variables:**<br>• User-initiated self-reporting (in-app food dairy with a bar-code scanner and meal photography)	• Not applicable

**Miller (2021)**	• Automated goal-setting<br>• Generic<br>• Adaptive	• Adherence to the DASH eating pattern (scores based on target nutrient ranges: potassium, fiber, calcium, sodium, magnesium, saturated fat, protein)<br>• Dietary tracking	• Self-identified participant characteristics (e.g. having young children at home, working full-time, eating out often, dietary restrictions)	**For dynamic variables:**<br>• User-initiated self-reporting (self-monitoring of diet in the Nourish app)<br>**For static variables:**<br>• Not reported	• Not applicable
**Nezami (2022)**	• Automated goal-setting<br>• Personalized<br>• Adaptive	• Weight change last week<br>• Weigh days last week<br>• Dietary tracking last week<br>• Average red foods/calories per day last week in relationship to goal<br>• Average daily active minutes in relationship to goal<br>• Tracker wear days last week	• Not reported	**For dynamic variables:**<br>• Accelerometer/pedometer data<br>• Weighing scale data<br>• User-initiated self-reporting (tracking red foods in a food log in the PATH app)	**Connected for tailoring:**<br>• Activity tracker (Fitbit Inspire)<br>• Smart scale (Withings Body scale)
**Novak (2024) Vetrovsky (2023)**	• Guided goal-setting<br>• Personalized<br>• Adaptive	• Steps (review and feedback on weekly step goal)<br>• Walking pace (5 min of 60–100 steps/min)<br>• Sedentary behavior time (30 min of 0 steps/min and recorded heartrate)<br>• Individual action plans	• Not reported	**For dynamic variables:**<br>• Accelerometer/pedometer data<br>• Device-measured vital signs<br>• Phone counseling sessions	**Connected for tailoring:**<br>• Activity tracker (Fitbit Inspire 2)
**Pardos (2023)**	• Automated goal-setting<br>• Personalized<br>• Adaptive	• Sleep<br>• Physical activity (not specified)<br>• BMI<br>• Glucose values<br>• Blood pressure<br>• Mental health<br>• Weather<br>• Preferred types of recommendations	• Not reported	**For dynamic variables:**<br>• Data retrieved from the Personal Health Record (PHR)<br>• System-initiated self-reporting<br>• Weather data	**Connected for tailoring:**<br>• Connected PHR (e.g. smartwatch, manual user input)

**Park (2024)**	• Guided goal-setting<br>• Personalized<br>• Adaptive	• Sedentary behavior time (prompt after sitting for 1 hour, mean total daily sitting hours, longest sedentary period)	• Not reported	**For dynamic variables:**<br>• Accelerometer/pedometer data	**Connected for tailoring:**<br>• Activity tracker (Fitbit Inspire 2)
**Pellegrini (2015)**	• Automated goal-setting<br>• Generic<br>• Static	• Sedentary behavior time (20 minutes of consecutive sedentary time)	• Not reported	**For dynamic variables:**<br>• Accelerometer/pedometer data	**Connected for tailoring:**<br>• Accelerometer (Shimmer)
**Pimenta (2022)**	• Guided goal-setting<br>• Personalized<br>• Adaptive	• Daily step counts (goal achievement)<br>• Selected barriers to walking/achieving goals	• Psychological needs for exercise (competence score, relatedness score, autonomy score)	**For dynamic variables:**<br>• User-initiated self-reporting (user input in the application interface)<br>**For static variables:**<br>• Questionnaire (Basic Psychological in Exercise Scale)	**Not connected for tailoring:**<br>• Activity tracker (New-Lifestyles NL-2000i Activity Monitor)
**Plaete (2015) Poppe (2017, 2018, 2019a, 2019b)**	• Self-set goals<br>• Personalized<br>• Adaptive	• Physical activity<br>• Sedentary behavior<br>• Barriers to performing the behavior	• Age<br>• Gender	**For dynamic variables:**<br>• Questionnaires (not further specified)<br>**For static variables:**<br>• Pre-intervention questionnaire	• Not applicable
**Radhakrishnan (2020, 2021)**	• Guided goal-setting<br>• Personalized<br>• Static	• Steps (progress towards step goal)<br>• Weight-monitoring behaviors	• Gender (avatar gender)<br>• Skin tone (avatar skin tone)	**For dynamic variables:**<br>• Accelerometer/pedometer data<br>• Weight-monitoring data<br>**For static variables:**<br>• Not reported	**Connected for tailoring:**<br>• Activity tracker (Go activity tracker)<br>• Smart scale (Body + smart weight scale)

**Reinwand (2013)**<br>**Storm (2016)**	• Self-set goals<br>• Personalized<br>• Adaptive	• Physical activity<br>• Fruit and vegetable intake	• Risk perception<br>• Outcome expectancies	**For dynamic variables:**<br>• Questionnaires (International Physical Activity Questionnaire Short Form, Four questions regarding fruit, fruit juice/vegetable juice, cooked or steamed vegetable, and salad and raw vegetable)<br>**For static variables:**<br>• Questionnaires (Adaption of Perloff's and Fetzer's perceived vulnerability scale, Four items with two about positive and two about negative expectancies)	• Not applicable
**Richardson (2007, 2010)**	• Automated goal-setting<br>• Personalized<br>• Adaptive	• Steps (mean steps and goal achievement)	• Age<br>• Gender<br>• Ethnicity<br>• BMI<br>• Marital status<br>• Children<br>• Home status<br>• Employment<br>• Family history<br>• Health conditions<br>• Personality<br>• Preferred activities<br>• Ability to perform physical activity<br>• Dog ownership<br>• Smoking<br>• Social support<br>• Motivation<br>• Self-efficacy<br>• Try to lose weight<br>• Barriers for physical activity	**For dynamic variables:**<br>• Accelerometer/pedometer data<br>**For static variables:**<br>• Questionnaire	**Connected for tailoring:**<br>• Activity tracker (Omron HJ-720IT pedometer)

**Schoenthaler (2020)**	• Self-set goals<br>• Generic<br>• Adaptive	• Patient Reported Outcomes (PROs) for (1) Lifestyle: Healthy living goal (lose weight, eat more fruits and vegetables, eat less sweets and carbohydrates, have better portion control); overall diet; sleep quality; physical activity, (2) Medication: Medication adherence, (3) Quality of life: Feeling of control over diabetes, (4) Emotional health: Feeling irritable or moody owing to diabetes<br>• Response rate to the messages<br>• Duration of intervention use	• Language	**For dynamic variables:**<br>• Ecological Momentary Assessment (EMAs)<br>**For static variables:**<br>• Not reported	• Not applicable
**Schultz (2022)**	• Guided goal-setting<br>• Personalized<br>• Static	• Physical activity (achievement of a nature-based physical activity SMART goal)	• Friend name as support person if the SMART goal was not reached	**For dynamic variables:**<br>• System-initiated self-reporting (SMS prompts)<br>**For static variables:**<br>• Patient Intake Form	• Not applicable
**Shibuta (2023)**	• Guided goal-setting<br>• Personalized<br>• Adaptive	• Steps (achievement of step goals)<br>• Barriers for walking<br>• Diet<br>• Blood pressure<br>• Weight<br>• Glucose values	• Physical activities from the "Evaluation scale for self-management behavior related to physical activity of type 2 diabetic patients (ES-SMBPA-2D)"	**For dynamic variables:**<br>• Accelerometer/pedometer data<br>• Device-measured vital signs/ body parameters<br>• User-initiated self-reporting (diet)<br>• System-initiated self-reporting (barriers)<br>**For static variables:**<br>• Questionnaires (ES-SMBPA-2D)	**Connected for tailoring:**<br>• Activity tracker (Terumo MT-KT02DZ)<br>• Smart weighing scale (Omron HBF-255T)<br>• Blood pressure monitor (Omron HEM-7271T)<br>• Glucometer (Terumo MS-FR201B)

**Spruijt-Metz (2022)**	• Guided goal-setting<br>• Personalized<br>• Adaptive	• Steps (anti-sedentary messages: at least 40 min of time with fewer than 150 steps)<br>• Time of day<br>• Weekday/weekend<br>• Weather<br>• Location (work, home, other)	• Not reported	**For dynamic variables:**<br>• Accelerometer/pedometer data<br>• GPS	**Connected for tailoring:**<br>• Smart watch (Fitbit Versa Lite activity tracker)
**Stein (2019)<br>Tabak (2018)**	• Guided goal-setting<br>• Personalized<br>• Adaptive	• Lifestyle goals (achievement of three goals chosen from a list of 19 goals regarding physical activity or diet)	• Not reported	**For dynamic variables:**<br>• System-initiated self-reporting (SMS prompts)	**Connected for tailoring:**<br>• Activity tracker (Fitbit)
**Steinberg (2020)**	• No goal-setting	• DASH diet adherence score	• Gender (women)	**For dynamic variables:**<br>• User-initiated self-reporting (daily self-monitoring of all foods and beverages in the Nutritionix app)	• Not applicable
**Sun (2020)**	• Automated goal-setting<br>• Generic<br>• Static	• Sedentary behavior time (prompt after no physical activity for 1 hour)	• Not reported	**For dynamic variables:**<br>• Accelerometer/pedometer data<br>• Gyroscope data.	**Connected for tailoring:**<br>• Google's Activity Recognition Transition API
**Sze (2023)<br>Waki (2024)**	• Guided goal-setting<br>• Personalized<br>• Adaptive	• Steps (progression towards goal)<br>• Barriers for step goal achievement	• Culture of the older Japanese population	**For dynamic variables:**<br>• Accelerometer/pedometer data<br>• System-initiated self-reporting	**Connected for tailoring:**<br>• Activity tracker (OMRON HJA-405 T-W or Yamasa AW-001)<br>**Not connected for tailoring:**<br>• Smart scale (OMRON body composition meter HBF-255T)<br>• Smart blood pressure monitor (OMRON brachial blood pressure monitor HEM-7271T)

**Tabak (2013, 2014a, 2014b, 2014c) Wieringa (2011)**	• Automated goal-setting<br>• Personalized<br>• Adaptive	• Physical activity behavior (difference between physical activity behavior and reference activity level)<br>• Previous motivational cues<br>• Context factors (a.o. weather conditions, time of day)	• Not reported	**For dynamic variables:**<br>• Accelerometer/pedometer data	**Connected for tailoring:**<br>• Activity tracker (3D-activity sensor MTx-W sensor or Inertia Technology B.V.)
**Tamura (2020)**	• Automated goal-setting<br>• Personalized<br>• Adaptive	• Location (a geofence or geographic boundary in a 0.75 mile (approximately 1200 meter) radius around each PA venue)	• Gender (women)<br>• Ethnicity (Afro-American)	**For dynamic variables:**<br>• GPS (geofencing)	**Not connected for tailoring:**<br>• Activity tracker (Fitbit Charge 2, all participants)<br>• Wireless blood pressure monitor (up to 25 participants)<br>• Weighing scale (up to 25 participants)<br>• Blood glucose monitor (up to 25 participants)
**vanderWeegen (2013, 2015) Verwey (2014a, 2014b)**	• Guided goal-setting<br>• Personalized<br>• Adaptive	• Minutes of moderate to vigorous activity (related to personal goal)	• Enjoyment and exertion of activities<br>• Barriers and facilitators to physical activity	**For dynamic variables:**<br>• Accelerometer/pedometer data<br>**For static variables:**<br>• System-initiated self-reporting (dialogue sessions in the app/web-app)	**Connected for tailoring:**<br>• Activity tracker (3D-activity monitor)
**vanGenugten (2010, 2012, 2014)**	• Guided goal-setting<br>• Personalized<br>• Adaptive	• Goal/behavioral performance<br>• Barriers to goal performance (motivation, negative thoughts about one self or change)<br>• Identified high-risk situations in past or future	• Reasons for weight loss<br>• Current diet and PA behavior<br>• Confidence to change<br>• Willingness to change<br>• Future weight goal	**For dynamic variables:**<br>• System-initiated self reporting (question in the module to indicate goal performance, barriers, and high-risk situations)<br>**For static variables:**<br>• System-initiated self reporting (questions in the module)	• Not applicable

**Watson (2012)**	• Guided goal-setting<br>• Personalized<br>• Adaptive	• Steps (e.g. whether they had met their step count goals)<br>• Progress in the system (e.g. whether they had progressed past baseline)<br>• Discourse context (e.g. whether they had just asked the virtual coach a question or asked for help)	• Not reported	**For dynamic variables:**<br>• Accelerometer/pedometer data	**Connected for tailoring:**<br>• Activity tracker (ActiPed)
**Yom-Tov (2017)**	• Guided goal-setting<br>• Personalized<br>• Static	• Activity attributes (number of minutes of activity in the last day, cumulative number of minutes of activity this week, fraction of activity goal, fraction versus expected at this point in the week)<br>• Feedback attributes (number of days since each feedback message was sent)	• Demographics (age, gender)	**For dynamic variables:**<br>• Accelerometer/pedometer data (smartphone app)<br>**For static variables:**<br>• Not reported	• Not applicable
**Zahedani (2023)**	• Automated goal-setting<br>• Personalized<br>• Adaptive	• Physical activity<br>• Diet (e.g. macronutrients such as carbohydrates, fat, and protein)<br>• Continuous glucose monitoring (CGM)<br>• Heartrate<br>• Time of day	• Age<br>• Gender<br>• Weight and height (for calorie restriction recommendations)	**For dynamic variables:**<br>• Accelerometer/pedometer data<br>• Device-measured vital signs/ body parameters<br>• User-initiated self-reporting (food and activity logging)<br>**For static variables:**<br>• Not reported	**Connected for tailoring:**<br>• Activity tracker or smart watch (Apple Watch/Fitbit)<br>• Continuous glucose monitoring (CGM, Freestyle Libre)

Author (year)	Type of decision points	Type of decision rules	Dynamically computer-tailored intervention options	Remaining intervention options
**Aguilera (2020)**	• Pre-specified time-interval<br>• The algorithm selects message timing daily, based on 2.5-hour intervals between 09:00 and 19:00	• Data-driven<br>• Adaptive<br>• Reinforcement Learning algorithm using contextual multi-armed bandit (MAB) problems. Bayesian approach: Thompson Sampling for continuous learning	• Visual<br>• Feedback and motivational textual messages	• Not applicable
**Almeida (2015) Estabrooks (2011)**	• Pre-defined schedule<br>• 3 IVR calls and 3 tailored newsletters at 1, 3, and 5 months post randomization	• Not reported	• Auditive, visual<br>• Interactive Voice Response with tailored messages and personal action planning<br>• Automated tailored newsletters with (1) targeted messages, (2) storylines based on goal achievement, (3) availability of PA resources as illustrated by a detailed street map, and (4) practical PA information	• Interactive computer session with an action planning session and identifying fitness facilities<br>• A free 12-month voucher to a fitness facility
**Alos (2022)**	• Real-time and pre-defined schedule<br>• Sitting bouts in real-time<br>• End of working day summary message<br>• End of working week motivational message	• Knowledge-driven<br>• Static<br>• If-Then rules	• Visual, haptic<br>• Phone vibrations as prompt to move<br>• Feedback and motivational messages<br>• Colored chair images<br>• Feedback graphs	• Infographic of advice on sitting less and moving more at work
**Al-Ozari (2018)**	• Pre-defined schedule and event-triggered<br>• 4 messages per day: 2 standard text messages, 1 personalized message based on personal motivator, and 1 messages in response to the biodata<br>• Months 1–3: diet, weight and healthy eating	• Knowledge-driven<br>• Static<br>• If-Then rules	• Visual<br>• Responsive and biofeedback messages	• Personalized messages<br>• Standardized messages

	• Months 4–6: physical activity and exercise<br>• Months 7–12: diet, weight, healthy eating, physical activity and exercise<br>• Event-triggered for need for help, cravings and lapse messages			
**Ambeba (2015)**<br>**Bizhanova (2023)**<br>**Burke (2017, 2020, 2022a, 2022b)**<br>**Cheng (2023)**<br>**Kariuki (2023)**<br>**Wang (2012)**	• Semi-random time-interval<br>• Dietary and PA feedback: 3 times daily<br>• Weight feedback: every 6–8 days	• Knowledge-driven<br>• Static<br>• Predefined thresholds and conditions based on participant goal	• Visual<br>• Feedback messages tailored to dietary intake, PA, and weight data	• Diabetes Prevention Program (DPP) online<br>• One-time 90-minute counseling session by a dietitian
**Baert (2018)**<br>**Bohanec (2021)**<br>**Clays (2021)**<br>**Voorend (2019)**	• Real-time and pre-defined schedule<br>• <u>Individualized exercise program:</u> weekly schedule<br>• <u>Daily exercise management:</u> real-time<br>• <u>Nutrition:</u> education every day of the first week, behavior second week and after that voluntarily every three months (advice 1-3 times per week)	• Knowledge-driven<br>• Static<br>• <u>Individual exercise plan:</u> qualitative multi-criteria decision analysis method DEX (rule-based structure)<br>• <u>Daily exercise management:</u> rule-based on safety margins<br>• <u>Nutrition:</u> probabilistic modeling	• Visual<br>• Individual endurance and resistance exercise program<br>• Exercise reminders<br>• Daily exercise management (e.g. advice to stop/pause/change intensity during exercise)<br>• Personalized feedback and education on nutrition	• In-app educational material
**Beckie (2024)**<br>**Sengupta (2020a, 2020b)**	• Real-time and pre-defined schedule.<br>• Goal achievement: real-time feedback<br>• Other messages: specific days or time of day	• Knowledge-driven<br>• Adaptive<br>• Decision rules with built-in flexibility for adaptation by the health coach	• Visual<br>• Motivational text messages with gender-specific graphic user interface	• Health behavior videos<br>• Peer chat<br>• Health coach contact by phone or via messaging

**Bennett (2013, 2018)**<br>**Foley (2012, 2016)**<br>**Steinberg (2013)**	• Pre-specified time-interval<br>• Weekly	• Knowledge-driven<br>• Static<br>• Rule-based decisions	• Auditive, visual<br>• Automated tailored feedback via IVR or SMS on progress towards behavior change goals<br>• SMS messages with weight loss progress	• Printed and video-based skills training materials for each Track goal<br>• 18 telephone counseling calls provided by a registered dietitian and psychology graduate students<br>• Primary care provider weight counseling during medical visits
**Boh (2016)**	• <u>Feedback module:</u> a combination of semi-random prompts and event-triggered<br>• <u>Cognitive module:</u> when a participant selects a cognition in the Cognition Evaluation module during a meal or diet temptation	<u>Feedback module:</u><br>• Data-driven<br>• Static<br>• Computational decision-tree algorithm determines rule-based classifications (65 pre-existing rules derived from a prior EMA dataset. Participants are assigned to rule-triggering groups based on EMA data. Reallocation of participants occurs at the end of week 3 based on new EMA data)<br><u>Cognitive module:</u><br>• Knowledge-driven<br>• Static<br>• Rule-based decision-making	• Visual<br><u>Feedback module:</u><br>• Feedback messages when at risk for overeating (warning message and behavioral advice)<br>• Summarized graphical feedback with insights into how variables are related to eating behavior<br><u>Cognitive module:</u><br>• Cognitive evaluation module with guided questioning and response formulation to critically assess cognition and formulate a helpful thought<br>• Generic or cCBT-based feedback message	• General information on dieting and exercising (two online sessions in week 2)<br>• 10 individual online sessions cognitive behavioral therapy on a computer (cCBT sessions)

**Bond (2014)**<br>**Thomas (2015)**	• Pre-specified time-interval<br>• Each minute	• Knowledge-driven<br>• Static<br>• Not further specified	• Auditive, visual<br>• Audible prompt with on-screen text to take a physically active break from sedentary behavior and possibility to react to prompt (performing a physical activity break, silencing the prompt, or delaying the prompt to reoccur after a set period of 30 minutes)<br>• Praising message and a green "go" light on the dashboard after successful SB break to the prompt (up to 10 go lights per day)<br>• Reminders to meet the walking prompt (triggered after 5 and 10 minutes of continued SB)	• One-time in-person education from a trained research staff member to provide a rationale for reducing sedentary behavior
**Boudreau (2016)**<br>**Moreau (2015)**	• Pre-defined schedule<br>• <u>Registration:</u> all constructs<br>• <u>Session 4:</u> PA behavior, intention and importance ruler<br>• <u>Session 8:</u> PA behavior, intention and confidence ruler<br>• <u>Self-monitoring tool:</u> event-triggered (when a PA session was entered)	• Knowledge-driven<br>• Static<br>• Decision trees	• Visual<br>• Tailored feedback PA behavior/ mediators of behavior during motivational sessions<br>• Self-monitoring tool with an overview of MVPA per week and evaluative feedback	• 8 motivational sessions (incl. videos)<br>• Action planning tool<br>• Safety tips<br>• FAQs tab
**Buchan (2020)**	• <u>Progress towards daily goal:</u> real-time<br>• <u>Personalized exercise program:</u> continuously adapted (not specified)	• <u>Progress towards daily goal:</u> not reported<br>• <u>Personalized exercise program:</u> complex algorithm (not specified)	• Auditive, visual<br>• LEDs on the wearable where more lights illuminate as progress towards the goal increases<br>• Real-time in app feedback of user data<br>• Personalized exercise program (incl. videos)	• Dietary sheet<br>• Facebook group

**Chokshi (2017)**	• Pre-specified time interval<br>• Daily assessment	• Knowledge-driven<br>• Static<br>• If-Then rules	• Auditive, visual<br>• Feedback on step goal with text message, automated interactive voice call, or email<br>• Loss-framed financial incentive (each week, participants had $14 allocated to a virtual account. If they met their daily step goal, the balance remained the same. However, for each day they failed to meet the goal, $2 was deducted from the account. Every Monday, the balance was reset to $14 for the new week)	• Not applicable

**Collins (2010, 2012, 2013)**	• Pre-specified time interval<br>• Phase 1: Weekly<br>• Phase 2: Weekly and monthly assessment	• Not reported	• Visual<br>Phase 1:<br>• Weekly (enhanced and maintenance phase) and monthly (maintenance phase) automated personalized feedback for nutrition and physical activity levels, the level of success of their weight loss journey, and general use of website features<br>• Calculations of energy balance and nutrition summaries referenced to recommended nutrient targets<br>• Graphical display of changes in body measurement data and body (BMI) silhouette<br>Additional in Phase 2:<br>• Maintenance-specific feedback on weigh-ins including warnings if the user's weight is creeping up<br>• Specific "relapse" weight loss program if weight rebounds by more than 3% of their baseline weight<br>• Congratulation emails for successful maintenance of lost weight	Phase 1:<br>• Weekly calorie-controlled, low fat menu plans and grocery list<br>• Weekly physical activity plan according to exercise preferences<br>• Weekly educational tips and challenges<br>• Social support via online forums<br>• Weekly email newsletters notifying the user of new content relevant to their point in the program<br>• Automated weekly reminders for entering weight<br>• A personalized enrolment report which suggests weight loss goals and prioritizes key behaviors<br>Additional in Phase 2:<br>• Weekly menu plans and exercise programs<br>• A certificate to celebrate the achievement of goal weight or reaching the maintenance phase<br>• Access to an online information page detailing evidence-based behavioral strategies to enhance the likelihood of weight loss maintenance<br>• Initial "welcome to weight maintenance phase" phone call from a trained consultant<br>• Regular questionnaires to help the user identify and prioritize behaviors that can help achieve weight maintenance and prevent rebounds

**Daryabeygi-Khotbehsara (2022, 2023)**	• Pre-defined schedule<br>• There are 5 decision points from 8 AM to 8:30 PM: morning, lunchtime, afternoon 1, afternoon 2, evening<br>• Notifications only if available (not driving, not sleeping, not already active, and online)	• Knowledge-driven<br>• Static<br>• If-Then algorithms	• Visual<br>• Prompts/messages to sit less or to move more<br>• In-app feedback on standing, sitting and walking	• Not applicable
**Dorsch (2018, 2020)**	• Not reported	• Data-driven<br>• Adaptive<br>• Artificial intelligence algorithms that takes each user's past, recognizes his or her present context, and predicts future activity	• Visual<br>• Just-in-time, contextually tailored messages when entering a grocery store or restaurant, based on the user's top 5 high-sodium foods and confidence in a low-sodium diet. In grocery stores, users can scan/search foods for sodium content using a traffic light system and receive lower-sodium alternatives. In restaurants, users receive three curated low-sodium meal options and can search menu items sorted by sodium content.	• Graphical feedback on blood pressure
**Evans (2015)**	• Pre-specified time-interval<br>• Physical activity and nutrition: weekly assessment<br>• Weight: based on number of days in the green/yellow/red zones	• Not reported	• Visual<br>• Immediate feedback via on-screen messages regarding their dietary and physical activity goal progress<br>• Feedback and behavioral strategy messages regarding weight in the green/yellow/red zone	• Individual face-to-face WLM consultation with a psychologist<br>• Theory-based messages (eg, motivation, resources)<br>• Graphical feedback on weight

**Finkelstein (2015)**	• Pre-specified time interval<br>• <u>Prompts for sitting break:</u> every 10-15 minutes<br>• <u>Report with step summary:</u> daily	• Knowledge-driven<br>• Static<br>• Decision tree. Possible blackout conditions in which no messages were sent: (1) self-reported preferences collected from a participant at enrolment, (2) participant texted S(X) (no messages for the next X hours), (3) participant texted 'okay' (no messages for the next hour).	• Visual<br>• Prompt to take a break from sitting: a text alert that sedentary time has exceeded healthy limits, providing suggestions from a message library for short activity breaks at work or home, tailored to the time of day<br>• Daily report with a summary of the user's steps from the previous day	• Links to exercise education materials (only during weekends)
**Forman (2019, 2019) Goldstein (2017, 2020, 2021a, 2021b)**	• Pre-specified time-interval<br>• EMA prompt ~6 semi-random intervals/day, spaced 2-3 hours apart. Assessment frequency varies per variable (e.g., mood assessed 3-4 times/day; alcohol once/day in the evening)	• Data-driven<br>• Adaptive<br>• Machine learning. Combines classification model predictions with variable selection models. Models that combined a weighted vote of predictions from logit boost, bagging, random subspace, random forest, and Bayes net	• Visual, auditive<br>• Risk alerts that indicate the risk for a lapse. Alerts can be opened to receive more comprehensive interventions that request user responses. 7–10 textual prompts per risk factor (total of 157 intervention options) based on the top 3 risk factors identified at a decision point.<br>• Goldstein2021b: 4 types of intervention options (Enhanced Education, Self-efficacy, Autonomous Motivation, Self-regulation) consisting of a randomly selected module (primarily composed of 3- to 5-minute interactive videos).	• Not applicable
**Gatwood (2020)**	• Pre-specified time-interval<br>• <u>Self-reported behaviors:</u> monthly adjustments<br>• <u>Preferences:</u> quarterly adjustments<br>• <u>Message frequency:</u> monthly increase if no improvement	• Knowledge-driven<br>• Static<br>• Not further specified	• Visual<br>• Tailored messages into 3 categories: (1) information related to diabetes self-care (educational), (2) motivation to adhere to the three focus areas (motivational), and (3) guidance for participants on achieving self-care goals (goal setting).	• Standardized messages for additional self-care topics (e.g., immunizations, stress management, blood sugar monitoring)

**Golbus (2024)**<br>**Hellem (2023)**	• Pre-defined schedule<br>• 4 daily time points (morning, lunch, afternoon, evening) with participants having a 25% probability of receiving a notification at each decision point	• Likely knowledge-driven<br>• Static, but unclear if additional rules apply<br>• Not further specified	• Visual<br>• Community- and expert-generated push notifications to promote increased physical activity and lower-sodium food choices	• Feedback graphs with steps, salt consumption and BP<br>• Possibility to search for lower-sodium food options
**Gupta (2015)**	• Pre-specified time interval<br>• The user configures alerts by specifying the number of daily alerts and the acceptable time frame<br>• The system calculates the total available time within that window, divides it by the number of alerts, and sets alerts at evenly spaced intervals throughout the day	• Knowledge-driven<br>• Static<br>• Rule aggregator	• Visual<br>• Contextual alerts: Positive and motivational message + Context based physical activity recommendation	• Location-Based Services: The app identifies nearby gyms and parks, allowing users to select a location. Once chosen, it tracks activities like walking, jogging, running, or cycling, recording distance, calories burned, time taken, and the user's path<br>• Recommended Exercises: The app offers various exercises for users to follow, providing guidance and suggestions for physical activities.
**Hamborg (2024)**<br>**Martens**<br>**Anderson (2022)**	• Pre-defined schedule<br>• <u>Physical activity prompt:</u> Wednesdays at 3 p.m. (15:00)<br>• <u>Follow-up on action plans:</u> Sundays at 11 a.m. (11:00)	• Knowledge-driven<br>• Static<br>• Decision tree	• Visual<br>• Autogenerated text messages to prompt, self-monitor, and support the execution of physical activity plans.	• Not applicable
**Hemnes (2021)**<br>**Martin (2015)**	• Pre-defined schedule<br>• 3 texts per day, aligned with their preferred morning, lunch, and evening leisure schedule defined at enrollment	• Knowledge-driven<br>• Static<br>• Theory-based algorithm	• Visual<br>• Tailored texting prompts with real-time progress toward their daily step count target	• Not applicable
**Hietbrink (2023a, 2023b)**	• Pre-defined schedule<br>• 2 semi-random times per day and 7 days after setting a goal	• Knowledge-driven<br>• Static<br>• IF-THEN rules. There were three types of decision rules: (1) rules that triggered the type of motivational message, (2) rules that triggered feedback on goal achievement, and (3) rules that triggered a type of psychological exercise	• Visual<br>• Motivational messages<br>• Feedback on goal achievement<br>• Psychological exercises toward the identified barrier	• Not applicable

**Hurley (2015)**	• Event-triggered<br>• Automated feedback was provided as per the participant's intervention assignment when a step report was obtained (intention to receive a daily step report).	• Knowledge-driven<br>• Static<br>• Not further specified	• Visual<br>• Praise messages<br>• Daily reward points and incentives (daily point when step goal was met, $5 for every 5 points earned that could be exchanged for retail gift cards or used to support a charity)	• 2 health information brochures<br>• Daily text message prompts (except when EMA was administered) with motivational quotes, health risks of inactivity, benefits of PA, and encouragement to be active
**Khunti (2021), Morton (2015), Yates (2015)**	• Pre-specified time-interval<br>• Week 2-8: weekly assessment (the remaining time non-dynamic intervention options)<br>• Month 2-6: weekly tailored text message<br>• Month 7-12: approx. 2x/month tailored text message	• Knowledge-driven<br>• Static<br>• Rule-based decision flow	• Visual<br>• Feedback messages on step goal achievement<br>• Motivational or informational text messages with problem-solving strategies	• Advice leaflet<br>• Walking Away structured group education program and annual maintenance session<br>• Personalized and tailored text messages<br>• Telephone call with educator
**Kim (2024), Park (2024)**	• Pre-defined schedule<br>• Every morning at 8 AM	• Algorithms (not specified)	• Visual<br>• Feedback messages regarding the highest and lowest goal achievement rates of self-care behaviors on the previous day<br>• Mileage incentives for achieving goals, viewing educational materials and feedback messages, or recording self-care behaviors	• Educational materials using cartoons and videos in the mobile app (one per week)<br>• Facilitator offered weekly positive feedback via phone or text message<br>• Diabetic complication risk shared via phone or text message

**Klein (2014)**	• Pre-specified time-interval<br>• Weekly assessment	• Knowledge-driven<br>• Static<br>• A rule-based model consisting of 92 rules that specify the reasoning process	• Visual<br>• Motivational and informative messages related to the problematic constructs, consisting of 3 components: (1) a status update for the user, (2) a motivational message targeting a specific bottleneck (the person's bottleneck according to the reasoning process), and (3) a link to the relevant part of the website for more information<br>• Overview of the extent to which the user has reached his/her goals in the past week, which is represented as a percentage and an iconic thumb on the eMate website.	• Information about what is considered 'healthy behavior' with respect to food intake, exercise and medicine intake on the website
**Korinek (2018)**	• Achievement of the step goal and awarding of points are evaluated daily, but it is not specified whether this is done in real time, at an interval, or at a fixed time	• Knowledge-driven<br>• Static<br>• Not further specified	• Visual<br>• Point-based rewards (participants in the study could earn points by meeting daily step goals, with rewards ranging from 100 to 500 points per day, determined by a pseudo-random multisine cycle. Every time participants reached 2500 points, they received a $5 Amazon gift card)<br>• Graphical feedback on steps and goal achievement within the app	• Not applicable
**Leitner (2022)**	• Pre-specified time-interval<br>• Weekly assessment	• Data-driven<br>• Adaptive<br>• Random Forest model with Shapley Value-based interpretability	• Visual<br>• Feedback on blood pressure + Personalized lifestyle recommendation	• Not applicable

**Lim (2016)**	• Combination of event-triggered and pre-defined schedule<br>• Glucose: event-triggered (within 1 minute upon glucose meter use), weekly (average glucose levels sent every Monday at 10 AM), monthly (average glucose levels sent on the last day of the month at 11 AM)<br>• Physical activity: daily (physical activity report at 10 PM)<br>• Diet: event-triggered (after self-reported data-entry)	• Knowledge-driven<br>• Static<br>• Glucose: rules based on clinical practice guidelines (ADA and KDA)<br>• Physical activity and diet: not further specified	• Visual<br>• Automated tailored messages on lifestyle and blood glucose levels (e.g. actionable messages in response to blood glucose measurements)<br>• Daily reports of patients' physical activity levels including time and calorie expenditure<br>• Detailed information about dietary pattern on the website	• Standard diabetes education and lifestyle counseling sessions at baseline, 3 months, and 6 months.
**Lin (2015)**	• Pre-defined schedule<br>• Daily 2 hours before reported sleep times<br>• Weight on Sundays<br>• Motivation level on Wednesdays<br>• Health behavior on the other days	• Knowledge-driven<br>• Static<br>• Rule-based decision flow	• Visual<br>• Tailored feedback messages based on participant responses	• Clinical assessment consisting of a 20-minute one-on-one session with a dietitian<br>• A brief feedback session with a dietitian<br>• A visit with a study physician to review their health status<br>• Educational materials on diet and physical activity<br>• Weight control plan<br>• One-way "push" messages (3-4 times per day)
**Mansour-Assi (2022)**	• Pre-defined schedule<br>• 1 to 2 text messages are delivered at a consistent time each day<br>• 4-week format with each day a specific focus (e.g. Monday = weekly PA goal/feedback)	• Not reported	• Visual<br>• Tailored feedback messages	• Social media and online groups (Facebook messenger/ Instagram/ Twitter)<br>• Remote health coaching
**Martinho (2023)<br>Pinto (2022)**	• Event-triggered<br>• Automated feedback and recommendations were provided directly after a food/meal entry in the app	• Knowledge-driven<br>• Static<br>• Rule engine	• Visual<br>• Tailored feedback messages and recommendations	• Not yet applicable (under development)

**Miller (2021)**	• Pre-defined schedule<br>• Daily assessment for feedback regarding the weekly focus of the module-based feedback schedule (e.g. module 2-3: fiber in weeks 4 and 11, protein weeks 9 and 16)	• Knowledge-driven<br>• Static<br>• Not specified	• Visual<br>• Tailored feedback messages	• Educational materials and skills training on the DASH eating pattern within the Nourish app (several skills training videos and informational documents)<br>• An initial videoconferencing visit with a Nourish registered dietitian<br>• Responsive coaching from a Nourish registered dietitian<br>• Motivational messages
**Nezami (2022)**	• Pre-specified time-interval and semi-random prompts<br>• <u>In-app feedback:</u> weekly assessment<br>• <u>Tailored messages on self-monitoring data:</u> semi-random prompts	• Knowledge-driven<br>• Static<br>• Computer-tailored algorithms	• Visual<br>• In-app feedback on weight change and progress towards behavioral goals<br>• 3-4 weekly text-messages based on self-monitoring data	• Insight into progress toward their goals in the PATH app<br>• In-app weekly or biweekly lessons on behavioral strategies<br>• General messages regarding new lessons, parenting skills or general motivation
**Novak (2024) Vetrovsky (2023)**	• Pre-specified time-interval and pre-defined schedule<br>• <u>Walk Faster and Stand Up messages:</u> every 15 minutes with a maximum of one per day for Stand Up and two per day for Walk Faster<br>• <u>Goal Review:</u> Friday evenings between 8 and 10 pm<br>• <u>Feedback and Encouragement:</u> Sunday evenings between 6 and 8 pm<br>• <u>Action Plan Reminder messages:</u> according to individual participants' routines once or more times per week	• Knowledge-driven<br>• Static<br>• Triggering rules	• Visual<br>• Walk Faster and Stand Up messages<br>• Goal Review messages<br>• Feedback and Encouragement messages<br>• Action Plan Reminder messages	• Brief physical activity advice from the general practitioner at baseline<br>• Educational leaflet<br>• Receipt with physical activity prescription and a specific goal<br>• Phone counseling sessions at 2 weeks, then monthly till the six months<br>• Health Education messages

**Pardos (2023)**	• Achievement of the step goal and awarding of points are evaluated daily, but it is not specified whether this is done in real time, at an interval, or at a fixed time.	• Knowledge-driven<br>• Static<br>• Rule-based knowledge model	• Visual<br>• Tailored health recommendation cards based on the user's score in each category<br>• Credits to play a variety of games through the app<br>• Success indicator rings<br>• Status history graphs	• Not applicable
**Park (2024)**	• Real-time and pre-defined schedule.<br>• Prompts: Real-time. Participants can select the days and the time ranges to receive the move alerts<br>• Tailored text messages:<br>• Monday morning: review of sedentary behavior<br>Thursday: midweek sedentary behavior feedback<br>Sunday: encouragement to achieve the goal and sync the Fitbit to the app	• Knowledge-driven<br>• Static<br>• Rule-based algorithm	• Visual, haptic<br>• Haptic prompts to take a break from sitting<br>• Tailored text messages:<br>Monday's message includes feedback on the daily sitting goal, a weekly summary of sedentary time and longest sedentary period, suggestion for extra sitting breaks, and one tip from the Ten Top Tips (TTT) booklet.<br>Thursday's message provides midweek sedentary behavior feedback.<br>Sunday's message encourages participants to achieve their goal and sync the Fitbit to the app	• Ten Top Tips (TTT) booklet with tips to add sedentary breaks into their daily routine<br>• Smart water bottle as a natural motivator to have sedentary breaks
**Pellegrini (2015)**	• Pre-specified time-interval<br>• Not further specified.	• Knowledge-driven<br>• Static<br>• Not further specified	• Auditive, visual, haptic<br>• Noise of vibration prompt to stand up for at least 2 minutes and question to indicate adherence to prompt (stand, extend, can't stand, ignore)<br>• Every 2-minute reminders until the user stands up or chooses a different response to the prompt than standing up	• Not applicable

**Pimenta (2022)**	• Pre-defined schedule<br>• Day 1: Assign task<br>• Day 2 and subsequent even days: counseling<br>• Day 3 and subsequent odd days: assess/counseling<br>• Assessment and counseling every 8 days based on weekly step goals (Lite Physical Activity Component)	• Knowledge-driven<br>• Static<br>• Six if-then rules were derived and depicted in the dialogue steps through process flow diagrams	• Auditive, visual<br>• Anthropomorphic Conversational Agent (ACA) that provides verbal and visual feedback on step counts<br>• ACA counseling to overcome selected barriers to walking	• General information about T2D and complications provided by the ACA<br>• ACA counseling to improve competence, autonomy and relatedness
**Plaete (2015) Poppe (2017, 2018, 2019a, 2019b)**	• Pre-specified time-interval<br>• Weekly assessment in 5 sessions	• Knowledge-driven<br>• Static<br>• Not further specified	• Visual<br>• Iterative tailored feedback on their current behavior and their behavior change process each week<br>• Making and revising action and coping plans based on identified barriers	• Information/knowledge about the behaviors (tips and quizzes)<br>• Optional pages with tips and tricks to become more physically active or less sedentary<br>• Success stories tailored to age and gender<br>• Collecting points by using the intervention to earn victory cups
**Radhakrishnan (2020, 2021)**	• Daily assessment (e.g. daily progress reports and daily steps climbing up the mountain if the goal was attained) but data must be manually synced in the app with the Health Mate app	• Knowledge-driven<br>• Static<br>• Not further specified	• Visual<br>• Older adult avatar that is used to depict changes in health status, show progress (avatar climbs up the mountain), spend rewards (game coins to purchase healthy recipes or accessories for the game avatar), and provide feedback messages<br>• Leaderboard	• Each step on the mountain triggers heart-failure self-management educational content, problem-solving challenge (e.g. quizzes), mini-game (word puzzle or slot game), or bonus wheel spin for extra points

**Reinwand (2013) Storm (2016)**	• Pre-defined schedule<br>• Physical activity: first 4 weeks with one session per week (session 1-4)<br>• Fruit and vegetable intake: following 4 weeks with one session per week (session 5-8)	• Knowledge-driven<br>• Static<br>• Not further specified	• Visual<br>• Tailored ipsative and normative feedback on physical activity or fruit and vegetable intake consisting of textual and graphical feedback with prior behavior, last weeks behavior, and the recommended behavior	• One-time tailored feedback about their risk perception, outcome expectancies (session 1) and fruit and vegetable consumption (session 5)<br>• Defining own health outcomes<br>• Support in making goals, action plans, and coping plans and review of plans<br>• Motivating feedback to increase self-efficacy<br>• Identifying social support
**Richardson (2007, 2010)**	• Pre-specified time-interval<br>• Weekly assessment	• Knowledge-driven<br>• Static<br>• Logic statements/motivational message algorithm	• Visual<br>• Tailored feedback messages on steps and goal achievement	• Daily tips<br>• Motivational messages<br>• 6 tailored sessions/newsletters with behavior change strategies (e.g. discussing motivation, problem-solving, stimulating social support)<br>• Online community
**Schoenthaler (2020)**	• Pre-defined schedule (not clearly reported for both intervention options)<br>• Personalized reports (feedback messages): monthly overview with feedback over the last week or month<br>• Motivational messages: weekly (table 1) or every 10 days at 2 PM (figure 1)	• Knowledge-driven<br>• Static<br>• Decision rules	• Visual<br>• Personalized reports with 2 types of insight messages displaying 2 types of insights: (1) correlational, which compares associations between 2 different PROs, and (2) individual, which identify trends in patients' responses to the PROs over the past month<br>• 3 types of motivational text messages: (1) response-based (weekly supportive messages based on PRO responses), (2) activity-based (weekly messages based on response rates to the messages), and (3) completion-based (messages based on patient duration in the study)	• Reflective questions and tips regarding lifestyle behaviors

**Schultz (2022)**	• Pre-defined schedule<br>• Every Friday at 10 AM	• Knowledge-driven<br>• Static<br>• If-Then rules	• Visual<br>• Tailored motivational messaging regarding achieving nature-based physical activity SMART goal (but participants could opt-out for the messages)	• Not applicable
**Shibuta (2023)**	• Pre-specified time-interval<br>• Feedback on steps and evaluation messages: daily assessment<br>• Feedback on a combination of parameters and barrier identification and solutions: weekly assessment	• Knowledge-driven<br>• Static<br>• Algorithms with conditional equations (partly adapted from another program)	• Visual<br>• Daily feedback on the user's step goal achievement<br>• Weekly feedback on step count, blood pressure, body weight, and blood glucose levels<br>• Identification of barriers to walking and possible solutions<br>• Evaluation messages on BP, body weight, and blood glucose levels<br>• Evaluation and advice about diet	• Group-based educational session<br>• Lecture on hypertension and its management<br>• General information (action list) to increase step count<br>• Information for safe physical activity<br>• Action planning to achieve step goals<br>• Self-rating of action plan achievement<br>• Feedback on the user's favorable physical activity behaviors (when the user meets their step goal)<br>• Individualized advice to promote physical activity (when the user does not meet their step goal)
**Spruijt-Metz (2022)**	• Walking suggestions: semi-random decision points. Five times a day at user-specified times in the morning, mid-day, mid-afternoon, late-afternoon, and evening<br>• Anti-sedentary suggestions: pre-specified time-interval. Every 5 minutes	• Data-driven<br>• Adaptive<br>• Walking suggestions: Contextual bandit reinforcement learning algorithm<br>• Anti-sedentary suggestions: Sequential Risk Time Sampling (SeqRTS) algorithm<br>• Both algorithms take into account the availability of users when deciding whether to provide a suggestion message	• Visual<br>• Walking suggestion messages<br>• Anti-sedentary suggestion messages	• Feedback graph on MVPA goal progression and information on daily step count<br>• Physical activity week planning<br>• Activity log with physical activity statistics<br>• Morning motivational messages<br>• Reflection on experiences, barriers, goals and plans

**Stein (2019)**<br>**Tabak (2018)**	• Pre-specified time-interval<br>• Each Tuesday, participants will be prompted to report their current weight and indicate whether or not they met their behavior goals for the week	• Not reported	• Visual<br>• Tailored feedback messages on chosen behavior goal (reinforce successes and/or offer motivational strategies and the automatic opportunity, if appropriate, to increase their goal levels (e.g. increase the number of days per week they will try to meet the dietary or physical activity target))	• Participatory workplace intervention (i.e. the participatory workplace intervention involves worker-led teams designing and implementing solutions to promote healthier eating and physical activity at work. Guided by management and a research facilitator, these teams create tailored environmental and cultural changes)<br>• Skills training, informational and motivational text messages (an average of 5 days per week)<br>• Health risk assessment at baseline<br>• Weekly and monthly tips customized to the goals the participant selected<br>• Quarterly guidance from a health coach
**Steinberg (2020)**	• Pre-defined schedule<br>• Daily feedback at 12:00 PM EST for the first 2 weeks and weekly feedback for the remainder of the study	• Knowledge-driven<br>• Static<br>• An automated algorithm (not further specified)	• Visual<br>• Tailored and motivational feedback and advice on adherence to DASH	• Messages with links to animated videos designed to increase skills around different topics related to DASH<br>• The DASH booklet available from the National Heart, Lung, and Blood Institute
**Sun (2020)**	• Pre-specified time-interval (most likely based on the description)<br>• Not further specified	• Knowledge-driven<br>• Static<br>• Decision tree/if-then rules	• Visual, auditive<br>• Push notification with a simple contextualized suggestion for walking<br>• Micro-financial incentives (300 points for standing up after intervention delivery; 200 points for walking extra 500 steps after intervention delivery; 400 points for voluntarily moving around 10 min before the scheduled intervention delivery; and 100 points for every 1,000 steps walked (10,000 points = $1)	• Rich visualization UI to helps users to self-monitor physical activities and track adherence history and reward amount<br>• Remote guidance from a clinician or caregiver

**Sze (2023)**<br>**Waki (2024)**	• <u>Steps:</u> pre-defined schedule. Push notifications four times a day (at 11:00, 13:00, 16:00, and 18:00) and end of week feedback on achievement of goals over the week<br>• <u>Barriers:</u> pre-specified time-interval. Weekly assessment in app (and monthly revised with the pharmacist)	• Knowledge-driven<br>• Static<br>• Rule-based algorithm	• Visual<br><u>Steps:</u><br>• Daily push notifications with feedback on progress towards the step goal<br>• Daily feedback graphs regarding step goal<br>• Weekly written and graphical feedback on achievement of step goals over the week<br><u>Barriers:</u><br>• Personalized coping planning based on barriers for achievement of step goals<br>• Weekly feedback on implementation of coping strategies	• Monthly meetings with a community pharmacist to build motivation, knowledge and skills
**Tabak (2013, 2014a, 2014b, 2014c)**<br>**Wieringa (2011)**	• <u>Visual feedback:</u> real-time<br>• <u>Motivational cues:</u> pre-specified time-interval. A two-hour interval (Tabak 2014a) or adaptively timed based on prior responsiveness (Tabak 2014b) (predicted by analyzing previously given cues and learning when a patient was likely to respond well to the message by relating relevant context factors to patient compliance and content)	• Hybrid: knowledge-driven and data-driven<br>• Adaptive<br>• <u>Knowledge-driven:</u> Standard deviation thresholds from reference line (>10%)<br>• <u>Data-driven:</u> Timing of cues is adjusted based on prior responsiveness (learning from context factors and compliance) (Tabak 2014b).	• Visual<br>• Continuous visual feedback (graph displaying measured vs. reference activity)<br>• Text-based motivational cues (encouraging cues [>10% deviation below reference line], discouraging cues [>10% deviation above reference line] and neutral cues [≤10% deviation with reference line])<br>• Standard web portal for viewing activity data (daily, weekly, monthly)	• Questions on the smartphone about self-perceived activity performance and dyspnea and fatigue levels

**Tamura (2020)**	• Real-time	• Knowledge-driven<br>• Static<br>• If-then rules	• Visual<br>• Real-time messages tailored to PA resources within the participants' activity space or geographic region	• Informational modules on the benefits of PA, potential barriers to PA and methods to overcome these barriers<br>• Daily motivational messages<br>• Awards<br>• Graphs to self-monitor PA and health metrics measured by Bluetooth-enabled PA monitor, scale, blood pressure cuff and glucometer<br>• Social forum
**vanderWeegen (2013, 2015) Verwey (2014a, 2014b)**	• Pre-defined schedule<br>• After 3, 5 and 14 days if the goal is achieved or not achieved	• Knowledge-driven<br>• Static<br>• Rule-based decisions (e.g. percentage of target achieved: 100% or 80%)	• Visual<br>• Automated feedback messages in relation to the physical activity goal (e.g. tips, encouragement, positive trend, reward, barriers, facilitators, suggestions for goal adjustment)<br>• Real-time activity results and history in minutes of moderate to vigorous activity in relation to the personal goal	• Dialogue sessions in the app or web-app consisting of "diary sessions" about enjoyment and exertion of performed activities and "preparation for goal setting" about barriers and facilitators for PA<br>• Setting up activity action plan<br>• Self-Management Support Program (SSP) consisting of four consultations with a practice nurse (week 1, week 2, week 8-12, and week 16-24)<br>• Informational booklet (e.g. SQUASH questionnaire, locally organized PA options)

**vanGenugten (2010, 2012, 2014)**	• Pre-defined schedule<br>• Visit 2 and visit 3 (1 week between visits)	• Knowledge-driven<br>• Static<br>• Not further specified	• Visual<br>• Factual and visual feedback on goal/behavioral performance<br>• If poor goal performance: decisional balance of advantage and disadvantage for old & new behavior (if lack of motivation) or exercise to replace negative feeling with positive feelings (if negative thoughts about one self or change)<br>• Coping planning for high-risk situation	Visit 1:<br>• Generic information about weight gain prevention<br>• Personalized risk information<br>• Decisional balance<br>• One-time tailored feedback on diet and physical activity (sub-)behaviors and recommendations for change<br>• Feedback on reasons for weight loss and alternative views<br>• Peer story about positive experiences with difficulties with weight gain prevention (if user is not confident), anticipated regret about weight in 5 years (if user is not willing)<br>• Goal setting and action planning<br>• Guidance to plan for behavior change (e.g. social support, practical needs)<br>Visit 4:<br>• Generic information on reasons for monitoring and evaluating body weight<br>• Written and visual feedback of weight development in past weeks<br>• Learning about weight gain and when to undertake action<br>• Generic information how to continue with self-regulation<br>• Explanation about how to choose a new behavior change<br>• Create a personal reward plan<br>• Behavioral contracting<br>Generally available:<br>• Forum<br>• Recipe database<br>• Links to useful websites

**Watson (2012)**	• User-indicated moments<br>• Three times a week<br>• The virtual coach sets the commitment for the next day of interaction during a conversation	• Knowledge-driven<br>• Static<br>• Interdisciplinary developed algorithm-driven script	• Auditive, visual<br>• Review of pedometer step count, feedback and goal setting with a virtual coach (a computer-animated exercise advisor)	• Social interaction<br>• Tips on activity or diet<br>• Encouragement<br>• Advice about maintaining a healthy diet and activity level<br>• Graphs with activity levels over time on the website
**Yom-Tov (2017)**	• Pre-specified time-interval<br>• The Reinforcement Learning algorithm was rerun every day and makes a decision every morning about which SMS message to send	• Data-driven<br>• Adaptive<br>• <u>During the first 3 months:</u> Decisions based on a predefined policy (“initial policy”)<br>• <u>After the initial period:</u> Reinforcement learning algorithm (“learned policy”). The algorithm uses Bolzmann sampling to choose the best message with a probability distribution and continues to test less likely messages	• Visual<br>• Daily feedback SMS messages (negative feedback [performance avoidance], positive feedback relative to positive-self [mastery], positive feedback relative to positive-social [performance approach])<br>• Weekly summary SMS messages (activity reminder message, maximal increase message, significant increase message, maximal social message, significant social message)	• Not applicable
**Zahedani (2023)**	• Pre-defined schedule<br>• <u>During the first 14 days:</u> Daily tasks and feedback (specific food experiments)<br>• <u>During days 15–30:</u> Daily tasks with 3-5 personalized task cards per day	• Data-driven<br>• Adaptive<br>• Machine learning-driven model (RNN with LSTM architecture)	• Visual<br>• Individualized recommendations aimed at improving glycemic control, focusing on the enhancement of TIR for diet and PA<br>• Daily task cards (day 1-14: food experimentation tasks, time-restricted feeding tasks, day 15-30: carbohydrate reduction, caloric restriction, exercise regimens, time-restricted eating protocols, and mindfulness practices to enhance Time in Range)<br>• Program-related insight cards<br>• General insight cards<br>• Earning XPs for completing tasks	• General educational content
