# Supplementary material for: Systematic review of dynamically tailored eHealth interventions targeting physical activity and healthy diet in chronic disease
Source: NPJ Digit Med. 2025 Nov 19;8:696. doi: 10.1038/s41746-025-02054-7 (PMC12630729; doi:10.1038/s41746-025-02054-7)
Supplement: Supplementary file 4 — Supplementary data3 [file 41746_2025_2054_MOESM4_ESM.pdf]

Supplementary Data 3. Way of Delivery

Author (year)	Mode of delivery	Delivery platform	Blended-care	Blended-care setting	Type of blended-support
**Aguilera (2020)**	• App	• iOS/iPadOS<br>• Android	Yes	• General practice (primary care)	• Face-to-face guidance
**Almeida (2015)**<br>**Estabrooks (2011)**	• Interactive voice response<br>• Automated telephone calls<br>• Mailings	• Not applicable	No	• Not applicable	• Not applicable
**Alos (2022)**	• App<br>• Website	• iOS/iPadOS<br>• Android	No	• Not applicable	• Not applicable
**Al-Ozari (2018)**	• Text messaging	• Not applicable	Yes	• Usual diabetes clinical service	• Face-to-face guidance
**Ambeba (2015)**<br>**Bizhanova (2023)**<br>**Burke (2017, 2020, 2022a, 2022b)**<br>**Cheng (2023)**<br>**Kariuki (2023)**<br>**Wang (2012)**	• App	• iOS/iPadOS<br>• Android	No	• Not applicable	• Not applicable
**Baert (2018)**<br>**Bohanec (2021)**<br>**Clays (2021)**<br>**Voorend (2019)**	• App	• Not reported	Yes	• Hospital care (secondary care)	• Not reported
**Beckie (2024)**<br>**Sengupta (2020a, 2020b)**	• App	• Android	Yes	• Health coach	• Remote guidance
**Bennett (2013, 2018)**<br>**Foley (2012, 2016)**<br>**Steinberg (2013)**	• Interactive voice response<br>• Text-messaging	• Not applicable	Yes	• General practice (primary care)<br>• Dietician	• Remote guidance<br>• Face-to-face guidance
**Boh (2016)**	• App<br>• Website	• iOS/iPadOS	No	• Not applicable	• Not applicable
**Bond (2014)**<br>**Thomas (2015)**	• App	• Android	No	• Not applicable	• Not applicable
**Boudreau (2016)**<br>**Moreau (2015)**	• Website	• Not applicable	No	• Not applicable	• Not applicable
**Buchan (2020)**	• App	• iOS/iPadOS	No	• Not applicable	• Not applicable

	• Activity tracker				
**Chokshi (2017)**	• App<br>• Text-messaging<br>• E-mail<br>• Interactive voice recording	• Not reported	No	• Not applicable	• Not applicable
**Collins (2010, 2012, 2013)**	• Website<br>• E-mail<br>• Text-messaging	• Not applicable	No	• Not applicable	• Not applicable
**Daryabeygi-Khotbehsara (2022, 2023)**	• App	• Android	No	• Not applicable	• Not applicable
**Dorsch (2018, 2020)**	• App	• iOS/iPadOS	No	• Not applicable	• Not applicable
**Evans (2015)**	• Mobile web interface<br>• Text-messaging	• Not applicable	Yes	• Study team member	• Remote guidance
**Finkelstein (2015)**	• App	• Android	No	• Not applicable	• Not applicable
**Forman (2019, 2019)**<br>**Goldstein (2017, 2020, 2021a, 2021b)**	• App	• iOS/iPadOS	No	• Not applicable	• Not applicable
**Gatwood (2020)**	• Text-messaging	• Not applicable	No	• Not applicable	• Not applicable
**Golbus (2024)**<br>**Hellem (2023)**	• App	• iOS/iPadOS<br>• Android	No	• Not applicable	• Not applicable
**Gupta (2015)**	• App	• Android	No	• Not applicable	• Not applicable
**Hamborg (2024)**<br>**Martens Anderson (2022)**	• Text-messaging	• Not applicable	Yes	• Physiotherapist	• Remote guidance
**Hemnes (2021)**<br>**Martin (2015)**	• Text-messaging	• Not reported	No	• Not applicable	• Not applicable
**Hietbrink (2023a, 2023b)**	• App	• iOS/iPadOS<br>• Android	Yes	• General practice (primary care)<br>• Hospital care (secondary care)	• Face-to-face guidance
**Hurley (2015)**	• Text-messaging	• Not applicable	No	• Not applicable	• Not applicable

**Khunti (2021)**<br>**Morton (2015)**<br>**Yates (2015)**	• Text-messaging	• Not applicable	Yes	• PROPELS educator (registered health care professionals or a suitable non-registered professional)	• Remote guidance
**Kim (2024)**<br>**Park (2024)**	• App	• Android	Yes	• Human facilitator	• Remote guidance
**Klein (2014)**	• App<br>• Website	• iOS/iPadOS<br>• Android	No	• Not applicable	• Not applicable
**Korinek (2018)**	• App	• Android	No	• Not applicable	• Not applicable
**Leitner (2022)**	• Text-messaging	• Not applicable	No	• Not applicable	• Not applicable
**Lim (2016)**	• Text-messaging	• Not reported	Yes	• Dietician<br>• Exercise specialist	• Face-to-face guidance
**Lin (2015)**	• Text-messaging	• Not applicable	Yes	• Dietician	• Face-to-face guidance
**Mansour-Assi (2022)**	• Text-messaging	• Not applicable	Yes	• Health coach	• Remote guidance
**Martinho (2023), Pinto (2022)**	• App	• iOS/iPadOS<br>• Android	No	• Not applicable	• Not applicable
**Miller (2021)**	• App<br>• Text-messaging	• Not applicable	Yes	• Dietician	• Remote guidance
**Nezami (2022)**	• App	• iOS/iPadOS	No	• Not applicable	• Not applicable
**Novak (2024)**<br>**Vetrovsky (2023)**	• Text-messaging	• Not applicable	Yes	• General practice (primary care)<br>• Counsellor	• Remote guidance
**Pardos (2023)**	• App	• Not reported	No	• Not applicable	• Not applicable
**Park (2024)**	• Activity tracker<br>• Text-messaging	• Not applicable	No	• Not applicable	• Not applicable
**Pellegrini (2015)**	• App	• Android	No	• Not applicable	• Not applicable
**Pimenta (2022)**	• App	• Not reported	No	• Not applicable	• Not applicable
**Plaete (2015)**<br>**Poppe (2017, 2018, 2019a, 2019b)**	• App<br>• Website	• Not reported	No	• Not applicable	• Not applicable
**Radhakrishnan (2020, 2021)**	• App	• iOS/iPadOS<br>• Android	No	• Not applicable	• Not applicable

**Reinwand (2013)**<br>**Storm (2016)**	• Website	• Not applicable	No	• Not applicable	• Not applicable
**Richardson (2007, 2010)**	• Website	• Windows	No	• Not applicable	• Not applicable
**Schoenthaler (2020)**	• Text-messaging (prompts)<br>• Not reported (graphs etc.)	• Not reported	Yes	• General practice (primary care)	• Face-to-face guidance
**Schultz (2022)**	• Text-messaging	• Not applicable	Yes	• General practice (primary care)	• Face-to-face guidance
**Shibuta (2023)**	• App	• Android	No	• Not applicable	• Not applicable
**Spruijt-Metz (2022)**	• App	• iOS/iPadOS<br>• Android	No	• Not applicable	• Not applicable
**Stein (2019)**<br>**Tabak (2018)**	• Text-messaging	• Not applicable	Yes	• Health coach	• Face-to-face guidance
**Steinberg (2020)**	• App<br>• Text-messaging	• iOS/iPadOS<br>• Android	No	• Not applicable	• Not applicable
**Sun (2020)**	• App	• Android	Yes	• Clinicians<br>• Caregivers	• Remote guidance
**Sze (2023)**<br>**Waki (2024)**	• App	• iOS/iPadOS	Yes	• Pharmacist	• Face-to-face guidance
**Tabak (2013, 2014a, 2014b, 2014c)**<br>**Wieringa (2011)**	• App<br>• Website	• Android	No	• Not applicable	• Not applicable
**Tamura (2020)**	• App	• Not reported	Yes	• Not reported	• Face-to-face guidance
**vanderWeegen (2013, 2015)**<br>**Verwey (2014a, 2014b)**	• App<br>• Web-application	• Not reported	Yes	• General practice (primary care)	• Remote guidance<br>• Face-to-face guidance
**vanGenugten (2010, 2012, 2014)**	• Website	• Not applicable	No	• Not applicable	• Not applicable
**Watson (2012)**	• Software installed on users' home computers	• Not applicable	No	• Not applicable	• Not applicable
**Yom-Tov (2017)**	• App<br>• Text-messaging	• Android	No	• Not applicable	• Not applicable
**Zahedani (2023)**	• App	• Not reported	No	• Not applicable	• Not applicable
