# Supplementary material for: Systematic review of dynamically tailored eHealth interventions targeting physical activity and healthy diet in chronic disease
Source: NPJ Digit Med. 2025 Nov 19;8:696. doi: 10.1038/s41746-025-02054-7 (PMC12630729; doi:10.1038/s41746-025-02054-7)
Supplement: Supplementary file 5 — Supplementary data4 [file 41746_2025_2054_MOESM5_ESM.pdf]

Supplementary Data 4. Theory and Behavior Change Techniques (BCTs)

Author (year)	Development framework	Behavior change theory	Behavior Change Technique (BCT) groups
**Aguilera (2020)**	• Not reported	• COM-B model	• Goals and planning<br>• Feedback and monitoring<br>• Shaping knowledge*<br>• Natural consequences<br>• Associations*<br>• Reward and threat<br>• Self-belief
**Almeida (2015)**<br>**Estabrooks (2011)**	• Not reported	• The Ecological Model of Physical Activity (EMPA)<br>• Protection Motivation Theory (PMT)	• Goals and planning<br>• Feedback and monitoring<br>• Shaping knowledge<br>• Natural consequences<br>• Repetition and substitution<br>• Comparison of outcomes<br>• Reward and threat<br>• Identity<br>• Self-belief<br>• Covert learning
**Alos (2022)**	• Behavior Change Wheel	• Theories underlying the Behavior Change Wheel	• Goals and planning<br>• Feedback and monitoring<br>• Social support<br>• Shaping knowledge<br>• Natural consequences<br>• Associations<br>• Reward and threat*
**Al-Ozari (2018)**	• Not reported	• Motivational interviewing<br>• Cognitive Behavior Therapy (CBT)	• Goals and planning<br>• Feedback and monitoring<br>• Natural consequences<br>• Self-belief

**Ambeba (2015)**<br>**Bizhanova (2023)**<br>**Burke (2017, 2020, 2022a, 2022b)**<br>**Cheng (2023)**<br>**Kariuki (2023)**<br>**Wang (2012)**	• Not reported	• Self-regulation theory	• Goals and planning<br>• Feedback and monitoring<br>• Shaping knowledge<br>• Comparison of outcomes<br>• Reward and threat<br>• Self-belief*
**Baert (2018)**<br>**Bohanec (2021)**<br>**Clays (2021)**<br>**Voorend (2019)**	• Human Centered Design	• Not reported	• Goals and planning<br>• Feedback and monitoring<br>• Shaping knowledge<br>• Natural consequences<br>• Associations<br>• Reward and threat*
**Beckie (2024)**<br>**Sengupta (2020a, 2020b)**	• Design science research (DSR) methodology	• Social Cognitive Theory (SCT)<br>• Theory of Planned Behavior (TPB)<br>• Self-Determination Theory (SDT)<br>• Trans Theoretical Model (TTM)<br>• Information, Motivation and Behavior skills model (IMB)<br>• Control Theory<br>• Theory of Reasoned Action<br>• Operant conditioning	• Goals and planning<br>• Feedback and monitoring<br>• Social support<br>• Shaping knowledge<br>• Natural consequences<br>• Associations<br>• Repetition and substitution<br>• Comparison of outcomes*<br>• Reward and threat*<br>• Antecedents
**Bennett (2013, 2018)**<br>**Foley (2012, 2016)**<br>**Steinberg (2013)**	• Not reported	• Social Cognitive Theory (SCT)	• Goals and planning<br>• Feedback and monitoring<br>• Social support<br>• Shaping knowledge<br>• Natural consequences*<br>• Repetition and substitution<br>• Comparison of outcomes<br>• Reward and threat*
**Boh (2016)**	• Not reported	• Cognitive Behavior Therapy (CBT)	• Goals and planning<br>• Feedback and monitoring<br>• Shaping knowledge<br>• Natural consequences<br>• Associations

			• Regulation<br>• Antecedents<br>• Identity<br>• Self-belief
**Bond (2014)**<br>**Thomas (2015)**	• Not reported	• Not reported	• Goals and planning<br>• Feedback and monitoring<br>• Natural consequences<br>• Associations<br>• Reward and threat
**Boudreau (2016)**<br>**Moreau (2015)**	• Planning model for tailored print materials	• Self-Determination Theory (SDT)<br>• Motivational interviewing<br>• I-Change Model	• Goals and planning<br>• Feedback and monitoring<br>• Social support<br>• Shaping knowledge<br>• Natural consequences<br>• Comparison of behavior<br>• Associations<br>• Comparison of outcomes<br>• Reward and threat<br>• Regulation<br>• Self-belief<br>• Covert learning
**Buchan (2020)**	• Not reported	• Not reported	• Goals and planning<br>• Feedback and monitoring<br>• Social support<br>• Shaping knowledge<br>• Natural consequences<br>• Comparison of behavior<br>• Associations<br>• Repetition and substitution<br>• Comparison of outcomes<br>• Reward and threat<br>• Antecedents<br>• Identity

**Chokshi (2017)**	• Not reported	• 4 important psychological principles: Individuals tend to be more motivated by losses than gains, favor immediate over delayed gratification, try to avoid the feeling of regret, and tend to be more driven for aspirational behavior around temporal landmarks such as the beginning of the week (the fresh start effect).	• Goals and planning<br>• Feedback and monitoring<br>• Reward and threat<br>• Scheduled consequences
**Collins (2010, 2012, 2013)**	• Not reported	• Social Cognitive Theory (SCT)	• Goals and planning<br>• Feedback and monitoring<br>• Social support<br>• Shaping knowledge<br>• Natural consequences*<br>• Reward and threat
**Daryabeygi-Khotbehsara (2022, 2023)**	• None	• None	• Goals and planning<br>• Feedback and monitoring<br>• Social support<br>• Shaping knowledge<br>• Natural consequences<br>• Associations<br>• Repetition and substitution
**Dorsch (2018, 2020)**	• Not reported	• Theory of Planned Behavior (TPB)<br>• Self-regulation theory<br>• Mindful decision making	• Goals and planning<br>• Feedback and monitoring<br>• Shaping knowledge<br>• Natural consequences*<br>• Associations<br>• Repetition and substitution
**Evans (2015)**	• User-centered design	• Self-regulation theory	• Goals and planning<br>• Feedback and monitoring<br>• Social support<br>• Shaping knowledge<br>• Natural consequences<br>• Repetition and substitution<br>• Reward and threat*<br>• Self-belief

**Finkelstein (2015)**	• Not reported	• Not reported	• Goals and planning<br>• Feedback and monitoring<br>• Shaping knowledge<br>• Associations
**Forman (2019, 2019)<br>Goldstein (2017, 2020, 2021a, 2021b)**	• Conceptual model of JITAI components	• Goldstein2021b: Information-Motivation-Strategy (IMS) model	• Goals and planning<br>• Feedback and monitoring<br>• Social support<br>• Shaping knowledge<br>• Natural consequences<br>• Associations<br>• Repetition and substitution<br>• Reward and threat<br>• Regulation<br>• Self-belief
**Gatwood (2020)**	• Planning model for tailored print materials	• Self-Determination Theory (SDT)<br>• Health Belief Model (HBM)	• Goals and planning<br>• Shaping knowledge*<br>• Natural consequences*<br>• Associations*
**Golbus (2024)<br>Hellem (2023)**	• Not reported	• Social Cognitive Theory (SCT)<br>• Self-Determination Theory (SDT)	• Goals and planning<br>• Feedback and monitoring<br>• Shaping knowledge<br>• Associations
**Gupta (2015)**	• Not reported	• Not reported	• Feedback and monitoring<br>• Associations
**Hamborg (2024)<br>Martens Anderson (2022)**	• Not reported	• Health Action Process Approach (HAPA)	• Goals and planning<br>• Feedback and monitoring<br>• Social support<br>• Associations<br>• Comparison of outcomes*<br>• Reward and threat
**Hemnes (2021)<br>Martin (2015)**	• Not reported	• Not reported	• Goals and planning<br>• Feedback and monitoring<br>• Shaping knowledge<br>• Natural consequences

			• Associations<br>• Reward and threat
**Hietbrink (2023a, 2023b)**	• Conceptual model of JITAI components<br>• Planning model for tailored print materials	• Health Action Process Approach (HAPA)<br>• Relapse prevention theory<br>• Rothman's theory of maintenance	• Goals and planning<br>• Feedback and monitoring<br>• Social support<br>• Shaping knowledge<br>• Natural consequences<br>• Comparison of behavior<br>• Associations<br>• Repetition and substitution<br>• Comparison of outcomes<br>• Reward and threat<br>• Regulation<br>• Identity<br>• Self-belief
**Hurley (2015)**	• Not reported	• Not reported	• Goals and planning<br>• Feedback and monitoring<br>• Shaping knowledge<br>• Natural consequences<br>• Reward and threat
**Khunti (2021)**<br>**Morton (2015)**<br>**Yates (2015)**	• Model by Dijkstra and De Vries for developing computer-generated tailored interventions<br>• The mHealth development and evaluation framework	• Social Cognitive Theory (SCT)<br>• Gollwitzer's implementation intentions<br>• Leventhal's common sense mode<br>• Chaiken's dual process theory	• Goals and planning<br>• Feedback and monitoring<br>• Social support<br>• Shaping knowledge<br>• Natural consequences<br>• Associations<br>• Repetition and substitution<br>• Comparison of outcomes<br>• Reward and threat<br>• Identity<br>• Self-belief
**Kim (2024)**<br>**Park (2024)**	• Not reported	• Self-regulation theory	• Goals and planning<br>• Feedback and monitoring<br>• Natural consequences*

			• Reward and threat
**Klein (2014)**	• Not reported	• The integrated model of behavior change COMBI	• Goals and planning<br>• Feedback and monitoring<br>• Social support<br>• Shaping knowledge<br>• Natural consequences<br>• Associations*<br>• Reward and threat<br>• Antecedents*
**Korinek (2018)**	• Not reported	• Social Cognitive Theory (SCT)	• Goals and planning<br>• Feedback and monitoring<br>• Reward and threat
**Leitner (2022)**	• Not reported	• Not reported	• Goals and planning<br>• Feedback and monitoring<br>• Shaping knowledge
**Lim (2016)**	• Not reported	• Not reported	• Goals and planning<br>• Feedback and monitoring<br>• Shaping knowledge<br>• Natural consequences*<br>• Associations<br>• Comparison of outcomes
**Lin (2015)**	• Not reported	• Health Belief Model (HBM)<br>• Trans Theoretical Model (TTM)<br>• Self-regulation theory	• Goals and planning<br>• Feedback and monitoring;<br>• Shaping knowledge<br>• Natural consequences<br>• Associations<br>• Comparison of outcomes<br>• Reward and threat
**Mansour-Assi (2022)**	• Not reported	• Operant conditioning<br>• Theories of social comparison<br>• Theories of social support<br>• Ecological theory	• Goals and planning<br>• Feedback and monitoring;<br>• Social support<br>• Shaping knowledge<br>• Comparison of behavior<br>• Comparison of outcomes

			• Antecedents
**Martinho (2023), Pinto (2022)**	• Not reported	• Trans Theoretical Model (TTM)	• Goals and planning<br>• Feedback and monitoring<br>• Shaping knowledge<br>• Natural consequences<br>• Repetition and substitution
**Miller (2021)**	• Not reported	• Social Cognitive Theory (SCT);<br>• Social Ecological Model	• Goals and planning<br>• Feedback and monitoring<br>• Social support<br>• Shaping knowledge<br>• Natural consequences<br>• Repetition and substitution<br>• Comparison of outcomes<br>• Reward and threat<br>• Self-belief
**Nezami (2022)**	• Not reported	• Social Cognitive Theory (SCT)	• Goals and planning<br>• Feedback and monitoring<br>• Social support<br>• Shaping knowledge<br>• Repetition and substitution<br>• Reward and threat*<br>• Identity
**Novak (2024)**<br>**Vetrovsky (2023)**	• The mHealth development and evaluation framework	• Self-regulation theory	• Goals and planning<br>• Feedback and monitoring<br>• Social support<br>• Shaping knowledge<br>• Natural consequences<br>• Associations<br>• Repetition and substitution<br>• Comparison of outcomes<br>• Reward and threat
**Pardos (2023)**	• Not reported	• Not reported	• Goals and planning<br>• Feedback and monitoring<br>• Shaping knowledge

			• Natural consequences<br>• Reward and threat
**Park (2024)**	• Not reported	• Habit Formation framework	• Goals and planning<br>• Feedback and monitoring<br>• Shaping knowledge<br>• Natural consequences<br>• Associations<br>• Repetition and substitution<br>• Reward and threat
**Pellegrini (2015)**	• Not reported	• Not reported	• Goals and planning<br>• Feedback and monitoring<br>• Associations
**Pimenta (2022)**	• Not reported	• Self-Determination Theory (SDT)	• Goals and planning<br>• Feedback and monitoring<br>• Social support<br>• Shaping knowledge<br>• Natural consequences<br>• Antecedents<br>• Self-belief
**Plaete (2015)**<br>**Poppe (2017, 2018, 2019a, 2019b)**	• Intervention mapping	• Health Action Process Approach (HAPA)<br>• Self-regulation theory	• Goals and planning<br>• Feedback and monitoring<br>• Social support<br>• Shaping knowledge<br>• Natural consequences<br>• Repetition and substitution<br>• Reward and threat
**Radhakrishnan (2020, 2021)**	• Not reported	• Fogg behavioral model	• Goals and planning<br>• Feedback and monitoring<br>• Shaping knowledge*<br>• Natural consequences<br>• Comparison of behavior<br>• Reward and threat<br>• Scheduled consequences

**Reinwand (2013)**<br>**Storm (2016)**	• Not reported	• Health Action Process Approach (HAPA)	• Goals and planning<br>• Feedback and monitoring<br>• Social support<br>• Shaping knowledge<br>• Natural consequences<br>• Repetition and substitution<br>• Reward and threat*
**Richardson (2007, 2010)**	• Not reported	• Social Cognitive Theory (SCT)<br>• Health Belief Model (HBM)<br>• Social learning theory	• Goals and planning<br>• Feedback and monitoring<br>• Social support<br>• Shaping knowledge<br>• Natural consequences<br>• Comparison of behavior<br>• Repetition and substitution<br>• Reward and threat<br>• Self-belief<br>• Covert learning
**Schoenthaler (2020)**	• User-centered design	• COM-B model<br>• Technology Acceptance Model (TAM)	• Goals and planning<br>• Feedback and monitoring<br>• Shaping knowledge<br>• Comparison of outcomes
**Schultz (2022)**	• RE-AIM framework	• Social Cognitive Theory (SCT)<br>• The Five A's model	• Goals and planning<br>• Feedback and monitoring<br>• Social support<br>• Reward and threat*
**Shibuta (2023)**	• Not reported	• Social Cognitive Theory (SCT)<br>• Trans Theoretical Model (TTM)	• Goals and planning<br>• Feedback and monitoring<br>• Social support<br>• Shaping knowledge<br>• Natural consequences<br>• Repetition and substitution<br>• Reward and threat<br>• Antecedents

**Spruijt-Metz (2022)**	• Not reported	• Not reported	• Goals and planning<br>• Feedback and monitoring<br>• Shaping knowledge<br>• Natural consequences<br>• Associations<br>• Repetition and substitution
**Stein (2019)**<br>**Tabak (2018)**	• Chambers and Norton's guidance and Stirman model	• Combination of various social-cognitive and socio-ecological factors	• Goals and planning<br>• Feedback and monitoring<br>• Social support<br>• Shaping knowledge<br>• Natural consequences<br>• Comparison of outcomes<br>• Reward and threat
**Steinberg (2020)**	• Not reported	• Not reported	• Feedback and monitoring<br>• Shaping knowledge<br>• Natural consequences<br>• Reward and threat
**Sun (2020)**	• User-centered design	• Not reported	• Goals and planning<br>• Feedback and monitoring<br>• Social support<br>• Associations<br>• Comparison of outcomes<br>• Reward and threat
**Sze (2023)**<br>**Waki (2024)**	• Not reported	• Social Cognitive Theory (SCT)	• Goals and planning<br>• Feedback and monitoring<br>• Social support<br>• Shaping knowledge<br>• Associations<br>• Comparison of outcomes<br>• Reward and threat
**Tabak (2013, 2014a, 2014b, 2014c)**<br>**Wieringa (2011)**	• Self-defined design approach consisting of (1) user needs/state of the art, (2) design, (3) evaluation, (4) product implementation	• Not reported	• Goals and planning<br>• Feedback and monitoring;<br>• Shaping knowledge<br>• Associations

			• Reward and threat*
**Tamura (2020)**	• User-centered design<br>• Community-based participatory research principles	• Adapted socio-ecological model	• Goals and planning<br>• Feedback and monitoring<br>• Social support<br>• Shaping knowledge<br>• Natural consequences<br>• Associations<br>• Reward and threat
**vanderWeegen (2013, 2015)**<br>**Verwey (2014a, 2014b)**	• User-centered design<br>• Shah's methodological framework for medical device development	• The Five A's model<br>• Physician-based Assessment and Counselling for Exercise intervention (PACE)	• Goals and planning<br>• Feedback and monitoring<br>• Social support<br>• Shaping knowledge*<br>• Natural consequences<br>• Comparison of outcomes*<br>• Reward and threat*
**vanGenugten (2010, 2012, 2014)**	• Intervention mapping	• Theory of Planned Behavior (TPB)<br>• Self-Determination Theory (SDT)<br>• Relapse prevention theory<br>• Precaution Adoption Process Model (PAPM)<br>• Self-regulation theory	• Goals and planning<br>• Feedback and monitoring<br>• Social support<br>• Shaping knowledge<br>• Natural consequences<br>• Associations<br>• Repetition and substitution<br>• Comparison of outcomes<br>• Reward and threat<br>• Regulation<br>• Antecedents*<br>• Self-belief<br>• Covert learning
**Watson (2012)**	• Not reported	• Combination of behavioral and social cognitive strategies	• Goals and planning<br>• Feedback and monitoring<br>• Social support<br>• Shaping knowledge<br>• Natural consequences*<br>• Reward and threat*

**Yom-Tov (2017)**	• Not reported	• Not reported	• Goals and planning<br>• Feedback and monitoring<br>• Natural consequences<br>• Comparison of behavior*<br>• Associations*
**Zahedani (2023)**	• Not reported	• Not reported	• Goals and planning<br>• Feedback and monitoring<br>• Shaping knowledge<br>• Natural consequences*<br>• Repetition and substitution<br>• Reward and threat<br>• Covert learning*

The * next to a BCT group indicates that it is likely that this behavior change technique is included in the intervention based on the intervention description, but this cannot be confirmed with certainty.
