# Supplementary material for: Systematic review of dynamically tailored eHealth interventions targeting physical activity and healthy diet in chronic disease
Source: NPJ Digit Med. 2025 Nov 19;8:696. doi: 10.1038/s41746-025-02054-7 (PMC12630729; doi:10.1038/s41746-025-02054-7)
Supplement: Supplementary file 6 — Supplementary data5 [file 41746_2025_2054_MOESM6_ESM.pdf]

Supplementary Data 5. Study Evaluation and Participant Characteristics

Author (year)	Inclusion criteria	Exclusion criteria	Recruitment/Setting
**Almeida (2015)**<br>**Estabrooks (2011)**	• No chest pains and anormal electrocardiogram during the treadmill stress test<br>• Currently physically inactive or insufficiently active (<150 moderate intensity physical activity/week)	• Younger than 18 years of age<br>• Had no access to a telephone<br>• Not able to read or understand English<br>• A contraindication to physical activity identified during stress testing<br>• Already had a membership to a recreation center	• During an outpatient health care visit and referred by the primary care physician
**Ambeba (2015)**<br>**Bizhanova (2023)**<br>**Burke (2017, 2020, 2022a, 2022b)**<br>**Cheng (2023)**<br>**Kariuki (2023)**<br>**Wang (2012)**	• Age ≥ 18 years<br>• Currently own and regularly use a smartphone with a data plan (iPhone or Android)<br>• BMI ≥ 27 and ≤ 43<br>• Complete a 5-day electronic food diary during the run-in period	• Having an unstable condition requiring physician-supervised diet and exercise (e.g. recent myocardial infarction)<br>• Physical limitations that preclude moderate-intensity PA<br>• Pregnant or intending to become pregnant during the study period<br>• Serious mental illness (e.g. schizophrenia)<br>• Alcohol intake > 4 drinks/day<br>• Currently in a formal weight loss program or use of weight loss medication<br>• History of bariatric surgery<br>• Score > 32 on the Eating Habits Checklist<br>• Unwilling to use the Fitbit device provided (or their own Fitbit device)	• Recruitment via Pitt+Me (a research registry hosted by the Clinical Translational Science Institute)<br>• University email announcements<br>• Electronic and postal mailings<br>• Study announcements on various social media sites<br>• Fliers posted in the community, and primary care practices with whom the Co-PI has collaborated for previous lifestyle studies

**Baert (2018)**<br>**Bohanec (2021)**<br>**Clays (2021)**<br>**Voorend (2019)**	• Willing and able to make use of a smartphone and to give informed consent for participation in the study<br>• Adults ≥18 years old<br>• Ischemic or non-ischemic HF disease<br>• Functional New York Heart Association (NYHA) class 2–3<br>• Reduced left ventricular ejection fraction ≤40%<br>• Ambulatory HF patients in stable condition<br>• Good cognitive function<br>• Sufficient knowledge of the native language (Dutch in Belgium, Italian in Italy)	• Suffering from a concomitant end-stage chronic kidney disease necessitating hemodialysis<br>• Already participating in a disease management program influencing the HeartMan intervention	• Recruitment via 3 hospitals in Belgium and one hospital and a local health authority in Italy
**Beckie (2024)**<br>**Sengupta (2020a, 2020b)**	• English-speaking women ≥35 years of age<br>• Documented coronary heart disease with or without coronary revascularization within the prior 5 year<br>• Able to participate in physical activity unaided	• A psychiatric diagnosis<br>• A debilitating neurological disorder<br>• A markedly shortened life expectancy	• Three outpatient cardiology clinics

**Bennett (2013, 2018)**<br>**Foley (2012, 2016)**<br>**Steinberg (2013)**	Steinberg2013<br>• Women aged 25-50 years<br>• A body mass index (BMI) greater than or equal to 25 kg/m2<br>• Come to all study assessments over 6 months<br>• Use a personal cell phone to send and receive up to 5 texts per day for 6 months without compensation for the text messages<br>• Be randomized into either treatment arm.<br>Bennett2018:<br>• Aged 21 to 65 years<br>• A BMI of 30.0-44.9 kg/m2<br>• A weight ≤ 330 pounds<br>• A current diagnosis of hypertension, type 2 diabetes, and/or hyperlipidemia<br>• At least two visits to a participating Piedmont center in the prior 12 months, North Carolina residency<br>• The ability to read and write in English<br>• Must have a mobile phone and be willing and able to send/receive 3-9 text messages per week	Steinberg2013:<br>• Pregnancy or planned pregnancy within the next 6 months<br>• A history of myocardial infarction or stroke within the past 2 years<br>Bennett2018:<br>• Current pregnancy<br>• Being ≤ 12 months postpartum<br>• Cohabitation with another study participant<br>• Current employment by Piedmont<br>• Current participation in another obesity treatment study or a study involving physical activity, high blood pressure, diabetes, or high cholesterol<br>• Plans to move outside of the study region within the next two years<br>• Must not have had a cardiovascular event in the prior 6 months or a diagnosis of coronary obstructive pulmonary disease<br>• Congestive heart failure or tachycardia<br>• No history of a condition that would affect body weight, for which weight loss is contraindicated, or that might impact treatment<br>• Having profound cognitive, developmental or psychiatric disorders or who have been hospitalized in a psychiatric facility in the prior 12 months	Steinberg2013:<br>• Via a nonprofit church-based community wellness organization located in Raleigh (North Carolina, USA)<br>• In the surrounding community via flyers posted in neighborhood businesses<br>• Outreach to adults in the area who had expressed interest in weight loss research trials<br>Bennett2018:<br>• Via four federally-qualified community health centers (CHCs) of Piedmont Health Services, Inc. (Piedmont), central North Carolina, USA. Participating CHCs are located in Carrboro, Burlington, and Prospect Hill, North Carolina
**Bond (2014)**<br>**Thomas (2015)**	• Participants were 21 to 70 years of age<br>• Overweight or obese (BMI≥25 kg/m2)	• Not reported	• Recruitment via study advertisements placed in local newspapers in Providence, Rhode Island, USA<br>• Research hospital network-affiliated intranet/internet sites<br>• Social media outlets (i.e. Facebook and Twitter)

**Boudreau (2016)**<br>**Moreau (2015)**	• Canadian men and women with self-reported T2D residing in the province of Quebec<br>• Not meeting the Canadian Diabetes Association guidelines on moderate-intensity aerobic physical activity<br>• Being able to understand French<br>• Having access to the Internet<br>• Being between 18 and 65 years of age<br>• Not having medical indications limiting the practice of PA	• Not reported	• Cananda, partnership with Diabète Québec for recruitment
**Buchan (2020)**	• Female<br>• Overweight or obese (BMI=25–30; waist circumference>80cm)<br>• Aged between 25 and 54 years<br>• Access to an iOS Apple device, internet, USB power outlet and weighing scale	• Significant weight loss in the past 6 months (>5% weight change)<br>• Those already following a weight loss program<br>• Serious medical conditions or contraindications to the practice of physical exercise<br>• Any medication that may affect body weight or blood pressure	• Recruitment among the general population
**Chokshi (2017)**	• Aged ≥18 years<br>• A history of acute coronary syndrome<br>• Had coronary catheterization for suspected ischemic heart disease that resulted in a definitive diagnosis	• Already enrolled in a formal cardiac rehabilitation program within the past 1 year<br>• Did not have access to a smartphone or tablet compatible with the wearable device<br>• Admitted to the hospital and were not being discharged to home<br>• Any other reason that participation was unsafe	• Four hospitals in southeastern Pennsylvania: Hospital of the University of Pennsylvania, Penn Presbyterian Medical Center, Chester County Hospital, and Lancaster General Hospital

**Collins (2010, 2012, 2013)**	• Aged between 18 and 60 years<br>• BMI between 25 and 40 kg/m2<br>• Agree to not participate in other weight loss programs during the study<br>• Pass a health-screening questionnaire and be available for assessment sessions<br>• Have access to a computer with e-mail and Internet facilities	• Currently pregnant or trying to become pregnant<br>• History of major medical problems such as heart disease or diabetes requiring insulin treatment<br>• Orthopedic or joint problems that are a barrier to physical activity<br>• Recent weight loss of ≥ 4.5 kg in previous 6 months<br>• Taking medications that might be affected by weight loss or affect weight loss	• Participants were recruited in the Hunter region of NSW (Australia) through advertising
**Daryabeygi-Khotbehsara (2022, 2023)**	• Diagnosed with type 2 diabetes<br>• Aged 35-65 years<br>• Own an Android smartphone & currently interact or engage with apps<br>• Have no limitations to engage in low- and moderate-intensity physical activity<br>• Be able to communicate in English<br>• Be able to provide consent	• Not reported	• Online advertisement on social media(Facebook and Instagram) among Australians
**Dorsch (2018, 2020)**	• Older than 18 years<br>• Diagnosed with hypertension<br>• On antihypertensive therapy for at least 3 months<br>• Using an iPhone were included	• Had chronic kidney disease (CKD), heart failure, systolic blood pressure >180 mmHg, diastolic blood pressure >110 mmHg, insulin-requiring diabetes mellitus<br>• Taking loop diuretics, corticosteroids, or nonsteroidal anti-inflammatory medications	• Michigan Medicine, formerly the University of Michigan Health System
**Finkelstein (2015)**	• Adult women with BMI > 30 kg/m2<br>• Inactive for > 3 hours on an average day	• Pregnant per participant's information<br>• Inability to walk<br>• Medical reasons to limit activity<br>• Poorly controlled hypertension, SBP >160 mm Hg, DBP >100 mg Hg	• Flyers and from previous focus groups

**Forman (2019a, 2019b) Goldstein (2017, 2020, 2021a, 2021b)**	Forman2019a:<br>• Adults (18–65 years old)<br>• Overweight or obesity (body mass index 25–50 kg/m2)<br>• Owned an iPhone<br>Forman 2019b:<br>• Owning an iPhone with a data plan<br>• Willing to purchase or be lent a wireless body scale<br>• BMI of 25–50 kg/m2<br>• Aged 18–70 years<br>• Living in the USA	Forman2019a:<br>• Being enrolled in another structured weight loss program<br>• Being pregnant or planning to become pregnant<br>• Reported disordered eating symptoms<br>• Had a medical condition that contraindicated weight loss<br>• Had begun or changed dosage of a weight or appetite-affecting medication in the past 3 months<br>• Had a history of bariatric surgery<br>Forman 2019b:<br>• Recent weight loss of 5% or more<br>• Current enrollment in another structured weight control program<br>• History of bariatric surgery<br>• Pregnant or planning to become pregnant within the study period<br>• Eating disorder symptomatology during the screening call<br>• Recent (i.e., within the last 3 months) change in weight-influencing medication<br>• Serious medical condition that may influence weight, appetite, or eating behavior	Forman2019a:<br>• Recruitment through print and online advertisements<br>Forman 2019b:<br>• Recruitment through press releases<br>• Facebook advertisements<br>• Radio ads
**Gatwood (2020)**	• Being a member of the Diabetes, Wellness, and Prevention Coalition Patient Advisory Council	• Not reported	• Members were recruited from urban areas within the study's operating region

**Golbus (2024)**<br>**Hellem (2023)**	Hellem2023:<br>• 18 years of age or older<br>• English-speaking<br>Golbus2024:<br>• Participants must be 18 years or older<br>• Have a self-reported history of hypertension with no medication changes in the past 4 weeks<br>• Own a smartphone with a compatible operating system (Apple or Android) for downloading the myBPmyLife app<br>• Have an email address<br>• Be fluent in spoken and written English<br>• Sign written informed consent	Hellem2023:<br>• Cognitive or language deficits<br>• Hearing or vision loss<br>• Could not read and write in English<br>Golbus2024:<br>• Contraindications to physical activity or a sodium-restricted diet<br>• Unstable symptoms or very high BP (SBP >180mmHg, DBP >120mmHg)<br>• End-stage renal disease<br>Difficulty using a BP cuff or smartwatch<br>• Daily sodium intake <1500mg<br>• Were pregnant or planning pregnancy<br>• Had heart failure<br>• Another household member in the study<br>• Involved in another wearable device or investigational study	Hellem2023:<br>• Waiting rooms at the FQHC clinic and the University of Michigan Cardiovascular Center<br>Golbus2024:<br>• Recruitment from University of Michigan Health, an academic medical center, and the Hamilton Community Health Network (HCHN), a network of federally qualified health center clinics in Flint, Michigan
**Gupta (2015)**	• Had obesity and stress	• Not reported	• Not reported
**Hamborg (2024)**<br>**Martens Anderson (2022)**	• Age ≥18 years<br>• Participation in a supervised exercise-based cardiac rehabilitation program in either hospital or municipality setting<br>• Access to a personal mobile phone with a Danish number<br>• Ability to walk 3 meters without assistance	• Insufficient Danish language skills to read and understand text messages and questionnaires<br>• Being cognitively or mentally unable to participate<br>• Terminal with a life expectancy of fewer than three months	• Slagelse and Holbæk Hospitals, Region Zealand, and in the City of Slagelse (municipality)

**Hemnes (2021)**<br>**Martin (2015)**	Martin 2015:<br>• Aged 18 to 69 years<br>• Using a Fitbug-compatible smartphone (ie, iPhone≥4S, Galaxy≥S3)<br>• Reporting <3 days/week of moderate or vigorous leisure-time activity lasting ≥30 min/day by the long form of the International Physical Activity Questionnaire (IPAQ)<br>Hemnes2021:<br>• Adults (aged 18 years or older)<br>• A diagnosis of idiopathic, heritable, drug/toxin-associated, or connective tissue disease-associated PAH who were in World Health Organization (WHO) functional class I, II, or III<br>• Participants were ambulatory and receiving a stable PAH medical regimen for at least 3 months	Martin2015:<br>• Not reported<br>Hemnes2021:<br>• Currently enrolled in another interventional study<br>• Unable to perform normal activity due to reliance on a cane/walker<br>• Activity-limiting angina or activity-limiting osteoarthritis<br>• PAH etiology other than idiopathic, heritable, or associated with connective tissue disease or toxins<br>• Pregnancy<br>• Forced vital capacity <65%<br>• WHO class IV heart failure<br>• Preferred form of activity is not measured by an activity tracker (e.g. swimming, yoga, or activities on wheels)	Martin2015:<br>• Recruited at an academic CVD prevention center in Baltimore, Maryland<br>Hemnes2024:<br>• Recruitment via Vanderbilt University Medical Center
**Hietbrink (2023a, 2023b)**	Hietbrink2023a:<br>• ≥18 years and were familiar with an Android smartphone (version 5.0 or higher)<br>Hietbrink2023b:<br>• Diagnosed with T2D<br>• In possession of a smartphone<br>• Dutch-speaking<br>• Signed an informed consent form	Hietbrink2023a:<br>• Underwent renal replacement therapy<br>• Were engaged in drug abuse<br>• Insufficient proficiency in the Dutch language<br>Hietbrink2023b:<br>• Unable to open or read text messages or emails	Hietbrink2023a+b:<br>• Recruitment via Ziekenhuisgroep Twente (ZGT) hospital, the Netherlands
**Khunti (2021)**<br>**Morton (2015)**<br>**Yates (2015)**	• Aged 40–74 years old for white European, or aged 25–74 years old for South Asian<br>• Have a previously recorded plasma glucose or HbA1c value in the prediabetes range within the last five years<br>• Have access to a mobile phone and willing to use it as part of the study	• Unable to take part in ambulatory-based activity<br>• Pregnant<br>• Involved in other related intervention studies<br>• Diagnosed with diabetes, or diabetes detected at baseline visit<br>• Unable to understand basic written and verbal English<br>• Unable to give informed consent	• Via primary care and existing databases from other studies

**Kim (2024)**<br>**Park (2024)**	Park2024:<br>• A T2DM diagnosis<br>• Age 40-69<br>• HbA1c of 7.0% in the last three months<br>• Android smartphone ownership<br>• The ability to walk without assistive devices	Park2024:<br>• Having difficulty using smartphones<br>• Serious pain<br>• Cognitive problems<br>• Participating in other self-care research<br>• Clinically unstable patients who received acute treatment within the past year	Park2024:<br>• Pusan National University Yangsan Hospital's diabetes outpatient clinic
**Korinek (2018)**	• Were generally healthy<br>• Insufficiently active<br>• 40–65 years old<br>• A body mass index (BMI) of 25–45 kg/m2<br>• Owned and regularly used an Android phone capable of connecting to a Fitbit Zip via Bluetooth 4.0<br>• Were willing to engage with the mHealth intervention for 14 weeks<br>• Considered insufficiently active (engaged in less than 1000 metabolic equivalent of task (MET)-minutes/week as measured by the International Physical Activity Questionnaire)	• Did not speak English<br>Were pregnant<br>• Had a BMI > 45 kg/m2<br>• Indicated medical problems that preclude unsupervised PA based on the Physical Activity Readiness Questionnaire (PAR-Q)<br>• Were currently participating in a commercial or research-related diet or exercise program	• Participants were recruited nationally through community advertising techniques
**Leitner (2022)**	• Be pre-hypertensive or have Stage I hypertension (SBP between 120-140/ DBP under 90 per ACC/AHA 2017 guidelines)<br>• Not be taking any antihypertensive medications	• Not reported	• Remote recruitment from anywhere in the USA
**Lim (2016)**	• Diagnosed with type 2 diabetes<br>• Aged 60 years or older<br>• A glycated hemoglobin (HbA1c) level of 7.0–10.5 % (53.0–91.3 mmol/mol)	• Being unable to use text messages<br>• Not having access to the internet for any reason	• Recruitment via the outpatient clinic of the Seoul National University Bundang Hospital (SNUBH)
**Lin (2015)**	• African American adults aged 21+ years<br>• A body mass index > 27<br>• Having text-messaging capability	• Self-reported substance abuse<br>• Uncontrolled hypertension<br>• A score > 38 on the Inventory of Depressive Symptomatology<br>• Other health conditions that made it unsafe to participate	• Baltimore-area churches

**Nezami (2022)**	• Be 21 to 55 years old<br>• Have a BMI of 25 to 50 kg/m2<br>• Have a child aged 2 to 12 years old<br>• Speak and read English<br>• Own an iPhone with an active data plan	• Current participation in another weight loss or nutrition program<br>• Being pregnant, breastfeeding, or planning pregnancy in the next 6 months<br>• Having lost more than 10 lb in the last 6 months<br>• 150 weekly minutes or more of moderate-to-vigorous physical activity<br>• Planning to relocate or be out of town for 2 weeks or more in the next 8 months<br>• Being unable to complete two study visits at the clinic<br>• Having preexisting medical condition(s) that preclude adherence to an unsupervised exercise program<br>• Undergoing treatment of diabetes with insulin or oral medications that may cause hypoglycemia<br>• Having a history of a diagnosed eating disorder<br>• Having a diagnosis of schizophrenia or bipolar disorder<br>• Being hospitalized for a psychiatric diagnosis within the last year<br>• Reporting a past diagnosis or current symptoms of alcohol or substance dependence	• Email lists and advertisements on Facebook and Instagram in the Raleigh-Durham area of North Carolina
**Novak (2024)**<br>**Vetrovsky (2023)**	• Diagnosis of prediabetes or type 2 diabetes according to the Czech guidelines for GPs<br>• Age 18 years or older<br>• Followed for prediabetes/diabetes by a participating GP<br>• Regular mobile phone users (not necessarily a smartphone), able and willing to answer calls and read text messages as part of the study<br>• Able and willing to wear and use a wrist-worn Fitbit activity tracker for the study duration<br>• Written informed consent provided before any assessment related to the study	• Unable to walk for any reason.<br>• Pregnant<br>• Having a household member already recruited for this study to avoid contamination<br>• Living in a residential or nursing care home where the imposed regime could interfere with the intervention<br>• Having any co-morbid conditions that would seriously affect their adherence to the trial procedures	• Recruitment was conducted by collaborating GPs in the Czech Republic

**Pellegrini (2015)**	• Adults between 21 and 70 years old<br>• Physician-diagnosed type 2 diabetes<br>• Needed to own an Android smartphone<br>• Be willing to wear an intervention accelerometer and use the NEAT! application on their smartphone<br>• Have a sedentary occupation or spend ≥75 % of the day sitting	• Unable to ambulate without assistance<br>• Did not wear the assessment accelerometer >7 days at baseline	• Via flyers in the Chicago land community and online postings (e.g., Craigslist)
**Plaete (2015)**<br>**Poppe (2017, 2018, 2019a, 2019b)**	Poppe 2018:<br>• Having type 2 diabetes<br>• Being ≥18 years old<br>• Dutch-speaking<br>• Not having participated in earlier studies with 'MyPlan'<br>Poppe 2019:<br>• Being literate in the Dutch language to engage in the intervention<br>• Being computer literate<br>• Having internet access<br>• Not having participated in the qualitative study about MyPlan 2.0<br>• Diagnosed with type 2 diabetes since at least 1 month<br>• Being 18 years or older	Poppe 2018:<br>• Not reported<br>Poppe 2019:<br>• Not reported	Poppe 2018:<br>• Diabetes Association Flanders<br>• The Ghent University Hospital<br>• Snowball sampling<br>Poppe 2019:<br>• The Ghent University Hospital and the Damian General Hospital (Ostend)<br>• Advertisements via the Flemish Diabetes Association<br>• In adults with type 2 diabetes who participated in the previous research of the involved research groups
**Radhakrishnan (2020, 2021)**	• Adults who were aged 55 years or older<br>• English-speaking<br>• Diagnosed with heart failure classified according to the New York Heart Association's HF classification as class II or III during their inpatient stay or outpatient visit to the cardiac center<br>• Own a smartphone<br>• Can independently walk without a walker or human assistance<br>• Have a score of 4 or higher on the Mini-Cog cognitive screen	• Severe visual or tactile impairments, which would prevent the use of a smartphone<br>• End-stage renal failure or terminal illness, both of which adversely affect HF prognosis	• Cardiac rehabilitation centres in Texas<br>• Via recruitment company Trialfacts to recruit participants from Texas and Oklahoma

**Reinwand (2013)**<br>**Storm (2016)**	• Age between 20 and 85 years<br>• No contraindications for physical activity and fruit and vegetable consumption<br>• Having an interest in improving physical activity and fruit and vegetable consumption<br>• Sufficient reading and writing skills in the relevant language (German or Dutch)<br>• Computer literacy and Internet access	• Not reported	• Cardiac rehabilitation centers and heart training groups in Germany and the Netherlands<br>• Online platforms focused on diabetes and cardiovascular diseases<br>• Research companies with online panels in both countries
**Richardson (2007, 2010)**	• Over 18 years of age<br>• Had at least one of the following: BMI ≥ 25, type 2 diabetes, or coronary artery disease<br>• Had access to an Internet-connected computer with Windows XP or Vista operating system<br>• Had a valid email address<br>• Used email at least once per week<br>• Had to be sedentary<br>• Had access to a treating physician who could provide medical clearance	• Were pregnant<br>• Could not walk a block on their own<br>• Could not make their own medicolegal decisions	• Recruitment from a University of Michigan Health System provider
**Schoenthaler (2020)**	<u>Patients</u><br>• Have had a diagnosis of T2D for ≥6 months<br>• Have had uncontrolled T2D, defined as HbA1c >7%, documented in the EHR at least twice in the past year<br>• Be fluent in English or Spanish<br>• Be willing to send and receive text messages<br>• Be aged ≥18 years<br><u>Providers</u><br>• Were a primary care provider practicing at the participating practices<br>• Provided care to at least five patients with T2D	<u>Patients</u><br>• Refused or were unable to provide informed consent<br>• Had acute renal failure, end-stage renal disease (ESRD) or evidence of dialysis, renal transplantation, or other ESRD-related services documented in the HER<br>• Were participating in another T2D study<br>• Had significant psychiatric comorbidity or reports of substance abuse<br>• Were pregnant or planning to become pregnant within 12 months<br>• Planned to discontinue care at the practice within the next 12 months	• Recruitment from a network of primary care practices of New York University Langone Health (NYULH)

**Shibuta (2023)**	• Employees of 4 private enterprises<br>• Systolic BP had been ≥140 mm Hg at a workplace health checkup in the fiscal year 2017<br>• Working in the Tokyo metropolitan area	• Systolic BP of ≥180 mm Hg<br>• Recent hemoglobin level <10 g/dL<br>• Diabetes other than type 2<br>Experience of any hypoglycemic events within the past 3 months<br>• Pregnancy, lactation, or pregnancy plans in the near future	• Recruitment via 4 enterprises in the service industry (e.g. department store) and belong to Tokyu Department Store Health Insurance Society
**Steinberg (2020)**	• Women aged 21–70 years<br>• BMI >18.5 kg/m^2<br>• Hypertension (self-reported or confirmed by medication use or having a recent systolic measurement of 120 to 159 mmHg or a diastolic blood pressure measurement of 80 to 99 mmHg)<br>• Owned a smartphone with an updated operating system<br>• A data plan<br>• An email address<br>• Fluency in English<br>• Willing to receive daily text messages	• A cardiovascular event in the last 6 months<br>• A current cancer diagnosis<br>• Had been institutionalized for a psychiatric disorder within the past year<br>• Were pregnant or lactating<br>• Enrolled in another dietary change study	• Flyers distributed to gyms, community centers, grocery stores, and health and wellness clinics throughout Raleigh, Durham, and Chapel Hill, North Carolina<br>• Study details posted on Research Match, a national clinical trials registry that matches participants to studies<br>• Social media posts on Twitter, Facebook, and Nextdoor<br>• Contact with participants who were ineligible for other studies but showed interest in similar behavioral studies
**Sun (2020)**	• Not reported	• Not reported	• Not reported

**Sze (2023)**<br>**Waki (2024)**	• Aged 20 years and above<br>• BMI 22 kg/m2 and above<br>• HbA1c 7.5 % or more as recorded in the latest blood investigation within three months from the time of consent acquisition<br>• The latest average number of steps per week was less than 10,000 steps / day<br>• Being in the contemplation, preparation, or action stage of the transtheoretical model (TTM) to achieve the target step count of 10,000 steps / day<br>• Willing to go to the designated community pharmacy for monthly meetings with the pharmacist during the research period	• Wearing a pacemaker<br>• History of hypoglycemic attack within three months from the time of consent acquisition,<br>• History of moderate or severe heart disease<br>• Blood pressure of 180/110 mmHg or higher<br>• History of severe heat stroke<br>• Moderate or severe mental illness<br>• Diagnosis of hyperthyroidism and a history of treatment other than thyroid hormone supplementation within the last 12 months<br>• Autoimmune disease, liver disease, gastrointestinal disease, or neurological disease, in which the chief of diabetes internal medicine judges that restriction of exercise therapy is necessary<br>• Respiratory illness<br>• Most recent blood investigations in the last three months from the time of study consent acquisition with hemoglobin (Hb) < 10 g / dL, or albumin (Alb) 3.0 g / dL or less, or eGFR 30 mL/min/1.73 m2 or less (diabetic nephropathy stage 4 and 5)<br>• Retinopathy diagnosed within the past year from the time of consent acquisition,<br>• Restrictions on medium-intensity aerobic exercise and physical activity (150 min / week of fast walking and 10,000 steps / day of walking as a guide)<br>• Pregnant or lactating women and women with a possibility of pregnancy<br>• Participating in other clinical studies at the time of study registration<br>• At time of enrollment, receiving diabetes dialysis prophylaxis or planning to receive diabetes dialysis prophylaxis during the intervention period	• Recruitment via Mitsui Memorial Hospital Japan

**Tabak (2013, 2014a, 2014b, 2014c)**<br>**Wieringa (2011)**	• A clinical diagnosis of COPD<br>• No infection or exacerbation in the 4 weeks prior to start of the study<br>• A current smoker	• Disorders or progressive disease seriously influencing daily activities or causing inability to use the smartphone application<br>• Other diseases influencing bronchial symptoms and/or lung function<br>• Need for regular oxygen therapy (>16 h per day or pO2 < 7.2 kPa)<br>• History of asthma<br>• Recently (<6 weeks) started training with a physiotherapist	• Recruitment via the department of pulmonary medicine of the Medisch Spectrum Twente hospital in Enschede, the Netherlands
**vanderWeegen (2013, 2015)**<br>**Verwey (2014a, 2014b)**	• Between 40 and 70 years old with DM2 or COPD<br>• Did not comply with the Dutch Norm for Healthy Exercise<br>• Access to a computer with an internet connection, and mastery of the Dutch language<br>• For the DM2 patients: BMI >25<br>• For the COPD patients: a clinical diagnosis of COPD according to the GOLD-criteria stage 1-3, known to be stable in their respiratory function for at least 6 weeks, and on a stable drug regimen	• The presence of coexisting medical conditions with a low survival rate<br>• Severe psychiatric illness<br>• Chronic disorders or diseases that seriously influence the ability to be physically active<br>• Being treated primarily by a medical specialist<br>• Participating in another PA intervention	• Recruitment via 24 family practices in the South of Netherlands
**vanGenugten (2010, 2012, 2014)**	• Being overweight (BMI = 25-30 kg/m2)<br>• Aged between 25 and 65 years<br>• Sufficient command of the Dutch language<br>• Access to the Internet is required	• Not having a sufficient command of the Dutch language<br>• Not having Internet access<br>• Being pregnant<br>• Following a diet prescribed diet<br>• Having a history of depression or eating disorders	• Through advertisements in local newspapers<br>• Flyers that were delivered door-to-door<br>• In waiting rooms of GP's<br>• Among the employees of 4 large companies<br>• Rotterdam region, the Netherlands

**Watson (2012)**	• Between 20 and 55 years old<br>• Had a BMI between 25 and 35 kg/m2<br>• Were fluent in spoken and written English<br>• Had a primary care physician<br>• Had access to a personal computer with an available USB port, speakers, and Internet access<br>• Either answered no to all 7 questions on the Physical Activity Readiness Questionnaire (PAR-Q) or obtained written permission from their primary care physician to take part in the study	• Not reported	• Recruitment took place in Boston, Massachusetts, USA<br>• Through advertisements in local newspapers<br>• On a local website (Craigslist)<br>• At healthcare facilities<br>• Through broadcast emails within the hospital email network
**Yom-Tov (2017)**	• Nonoptimal glycemic control (HbA1c over 6.5%)<br>• A sedentary lifestyle with no dedicated physical activity up to the recruitment to the study<br>• Ownership of an Android-based smartphone with a data connection	• Other types of diabetes<br>• Any disability that precludes walking for 20 min	• The endocrinology and diabetes outpatient clinic at a tertiary hospital in Israel
**Zahedani (2023)**	• Age of 18 years<br>• A prior diagnosis of diabetes who were not taking insulin<br>• A diagnosis of prediabetes<br>• No history of glucose abnormalities<br>• A signed disclaimer to use deidentified data	• Not reported	• Not reported

Author (year)	Intervention group(s) (IG)	Control group(s) (CG)	No. participants	Gender female (%)	Age Mean (SD)
**Almeida (2015)<br>Estabrooks (2011)**	• ENV: Environmental intervention<br>• SC: Social cognitive intervention<br>• COMBO: Combined environmental (ENV) and social cognitive (SC) intervention	• CON: Control condition (healthy eating personal action plan)	452	59.3	58.6 (9.7)
**Ambeba (2015)<br>Bizhanova (2023)<br>Burke (2017, 2020, 2022a, 2022b)<br>Cheng (2023)<br>Kariuki (2023)<br>Wang (2012)**	• SMARTER intervention (SM+FB group)	• Self-monitoring only (SM-group)	Burke2022b: 502<br>Cheng2023: 356	79.5	45.0 (14.4)
**Baert (2018)<br>Bohanec (2021)<br>Clays (2021)<br>Voorend (2019)**	• Heartman DSS intervention	• Usual care	Voorend2019: 10<br>Clays2021: 56	Voorend2019: 30.0<br>Clays2021: 23.0	Voorend2019: 56.3 (11.3)<br>Clays2021: 63.0 (10.5)
**Beckie (2024)<br>Sengupta (2020a, 2020b)**	• HerBeat intervention	• Educational usual care (usual care + workbook and 90-min DVD)	47	100.0	IG: 62.7 (7.3)<br>CG: 59.7 (10.5)
**Bennett (2013, 2018)<br>Foley (2012, 2016)<br>Steinberg (2013)**	• Shape/Track intervention	Steinberg2013:<br>• Education control arm (2 in-person group education sessions + a set of videos at 3 months that covered topics + pedometers + a “prescription” to walk 10,000 steps per day)<br>Bennett2018:<br>• Usual care (standard of care + trainings on obesity treatment + self-help materials + quarterly newsletters)	Steinberg2013: 50<br>Bennett2018: 351	Steinberg2013: 100.0<br>Bennett2018: 68.0	Steinberg2013: 38.3 (8.2)<br>Bennet2018: 50.7 (8.9)
**Bond (2014)<br>Thomas (2015)**	• Condition 1: 3-min break after 30 continuous sedentary minutes<br>• Condition 2: 6-min break after 60 continuous sedentary minutes	• Not applicable	30	83.0	47.5 (13.5)

	• Condition 3: 12-min break after 120 continuous sedentary minutes				
**Boudreau (2016)**<br>**Moreau (2015)**	• DEF computer tailored intervention (computer tailoring + PA cognitions messages)	• Not applicable	11	54.5	53.5 (Not reported)
**Buchan (2020)**	• Onitor Track + exercise intervention + dietary intervention + Facebook group	• Not applicable	15	100.0	Median (IQR):<br>42.0 (30.0-52.0)
**Chokshi (2017)**	• Financial incentive-based program (personalized step goal, self-monitoring with wearable, daily feedback and loss-framed financial incentives)	• Step goal 10.000 steps per day that could be adjusted, wearable, not informed about baseline step count	105	IG: 28.0<br>CG: 32.7	IG: 60.0 (9.5)<br>CG: 59.1 (11.5)
**Collins (2010, 2012, 2013)**	• IG1: Standard (Basic) commercial online weight loss program<br>• IG2: Extra support (Enhanced) version of the online weight loss program and received system-generated personalized feedback, based on diary entries and website use	• Wait-list control	301	58.5	41.9 (10.2)
**Daryabeygi-Khotbehsara (2022, 2023)**	• iMove intervention	• Not applicable	10	70.0	49.0 (8.6)
**Dorsch (2018, 2020)**	• LowSalt4Life intervention (app group)	• Usual care (no app group)	50	IG: 58.0<br>CG: 61.0	IG: 56.6 (10.0)<br>CG: 58.2 (11.0)
**Finkelstein (2015)**	• Group A: Inactivity reminder active (period 1), inactivity reminder inactive (period 2)<br>• Group B: Inactivity reminder inactive (period 1), inactivity reminder active (period 2)	• Not applicable	30	100.0	52.0 (12.0)
**Forman (2019a, 2019b)**<br>**Goldstein (2017, 2020, 2021a, 2021b)**	• Weight Watchers + OnTrack	• Weight Watchers	181	IG: 84.0<br>CG: 87.1	IG: 47.2 (13.3)<br>CG: 44.5 (14.1)

**Gatwood (2020)**	• Messages from MODEL intervention	• Not applicable	16	81.3	60.8 (range 45-70)
**Golbus (2024)**<br>**Hellem (2023)**	• Researcher-generated mHealth notifications<br>• Community-generated mHealth notifications	• Not applicable	86	FQHC: 58.0<br>UC: 70.0	FQHC: 48.6 (11.4)<br>UC: 49.1 (19.2)
**Gupta (2015)**	• Let's Exercise intervention	• Not applicable	33	Not reported	Range<br>24.0-30.0
**Hamborg (2024)**<br>**Martens Anderson (2022)**	• The FAIR intervention	• Not applicable	8	37.5	Median (range)<br>57.0 (37.0-74.0)
**Hemnes (2021)**<br>**Martin (2015)**	Martin2015:<br>• Unblinded self-monitoring without text messages<br>• Unblinded self-monitoring with text messages<br>Hemnes2024:<br>Text-messaging arm	Martin2015:<br>• Blinded self-monitoring<br>Hemnes2024:<br>• Usual care	Martin2015: 48<br>Hemnes2024: 42	Martin2015: 46.0<br>Hemnes2024:<br>IG: 75.0<br>CG: 95.0	Martin2015:<br>58.0 (8.0)<br>Hemnes2024:<br>Median (IQR)<br>IG: 47.0 (41.0-54.0)<br>CG 47.0 (36.0-58.0)
**Hietbrink (2023a, 2023b)**	Hietbrink2023a:<br>• Self-monitoring of physical activity, diet and glucose values + E-Supporter coaching content)<br>Hietbrink2023b:<br>• Self-monitoring with Fitbit and food diary + E-Supporter coaching content	• Not applicable	Hietbrink2023a: 9<br>Hietbrink2023b: 20 (15 for physical activity and 5 for nutrition)	Hietbrink2023a:<br>22.0<br>Hietbrink2023b:<br>30.0	Hietbrink2023a:<br>65.2 (8.7)<br>Hietbrink2023b:<br>68.0 (8.0)
**Khunti (2021)**<br>**Morton (2015)**<br>**Yates (2015)**	• Walking Away (WA): 3h group-based, theory-driven, behavioral intervention + annual refresher + pedometer<br>• Walking Away Plus (WAP): WA + mHealth follow-on support with goal-setting and automated feedback	• Advice leaflet about nondiabetic hyperglycemia and physical activity	1366	WA: 49.6<br>WAP: 49.1<br>CG: 49.1	WA: 59.4 (9.4)<br>WAP: 59.3 (9.1)<br>CG: 59.4 (8.8)
**Kim (2024)**<br>**Park (2024)**	• Automated Personalized Self-care Program (APSC program)	• Standard care	32	IG: 26.3<br>CG: 53.8	IG: 58.2 (7.0)<br>CG: 58.2 (8.9)
**Korinek (2018)**	• JustWalk intervention	• Not applicable	20	90.0	47.3 (6.1)

**Leitner (2022)**	• Experimental group 1 (self-monitoring + one-time recommendation<br>• Experimental group 2 (self-monitoring + weekly recommendations)	• Self-monitoring without recommendations	38	IG1: 33.0<br>IG2: 46.0<br>CG: 37.0	IG1: 50.1 (15.0)<br>IG2: 48.4 (12.2)<br>CG: 52.9 (13.1)
**Lim (2016)**	• U-healthcare intervention	• Self-monitored blood glucose (SMBG) group (glucose meter and activity monitor)	100	IG: 20.0<br>CG: 30.0	IG: 64.3 (5.2)<br>CG: 65.8 (4.7)
**Lin (2015)**	• TRIMM text message intervention (standard care + TRIMM)	• Standard care control (20-minute one-on-one sessions with a dietitian + a visit with a study physician to review their health status + educational materials on diet and physical activity + a digital pedometer)	124	IG: 90.5<br>CG: 78.7	IG: 49.2 (12.7)<br>CG: 52.3 (12.0)
**Nezami (2022)**	• Simplified group (75-minute group kickoff session + activity tracker (Fitbit Inspire) + smart scale (Withing's Body) + PATH study app + daily goals + simplified self-monitoring using the Traffic Light Diet)	• Standard group (75-minute group kickoff session + activity tracker (Fitbit Inspire) + smart scale (Withing's Body) + PATH study app + daily goals + daily self-monitoring of dietary caloric intake)	72	IG: 94.3<br>CG: 94.6	IG: 40.2 (4.7)<br>CG: 39.8 (4.7)
**Novak (2024)**<br>**Vetrovsky (2023)**	• ENERGISED intervention	• Not applicable	10	0.0	Range<br>40.0-76.0
**Pellegrini (2015)**	• NEAT! Intervention	• Not applicable	9 (8/9 completed the intervention)	78.0	53.1 (10.7)
**Plaete (2015)**<br>**Poppe (2017, 2018, 2019a, 2019b)**	<u>Poppe2018:</u><br>• MyPlan2.0 intervention<br><u>Poppe2019b:</u><br>• IG1: MyPlan2.0 Physical activity<br>• IG2: MyPlan2.0 Sedentary behavior	<u>Poppe2018:</u><br>• Not applicable<br><u>Poppe2019b:</u><br>• Waiting-list control group	<u>Poppe 2018:</u> 21<br><u>Poppe 2019b:</u> 54 (32 for PA and 22 for SB)	<u>Poppe2018:</u> 38.1<br><u>Poppe2019b:</u> 37.0	<u>Poppe2018:</u><br>65.9 (5.6)<br><u>Poppe2019b:</u><br>62.7 (8.4)
**Radhakrishnan (2020, 2021)**	• Heart Mountain intervention	• Sensors-only control for weight and physical activity self-monitoring	38	47.0	Age range (N)<br>55-64y: 53<br>65-74y: 32<br>>75y: 15
**Reinwand (2013)**<br>**Storm (2016)**	• RENATA intervention	• Waiting control group	790	62.9	50.8 (12.2)

**Richardson (2007, 2010)**	• Online community arm (study website to view graphs of their walking progress + individually-tailored motivational messages + weekly calculated goals + online community (post and read messages with other participants))	• No online community arm (study website to view graphs of their walking progress + individually-tailored motivational messages + weekly calculated goals)	324	65.0	52.0 (11.4)
**Schoenthaler (2020)**	• i-Matter intervention	• Not applicable	Patients: 10 (7 patients completed the first round of testing (1 Spanish-speaking) and 3 patients completed the second round)<br>Providers: 6	Patients: Not reported<br>Providers: 50.0	Patients and providers: Not reported
**Shibuta (2023)**	• DialBetes Step intervention	• Not applicable	30	37.0	52.9 (5.3)
**Steinberg (2020)**	• Dash Cloud intervention (daily diet tracking using the Nutritionix app + daily or weekly text messages + animated skills videos + the DASH booklet available from the NHLBI)	• Active Comparator Arm (daily diet tracking using the Nutritionix app + a video that introduced the DASH dietary pattern + the DASH booklet available from the NHLBI)	59	100.0	49.9 (11.9)
**Sun (2020)**	• BeActive intervention	• Not applicable	5	20.0	Range 23.0-34.0
**Sze (2023)**<br>**Waki (2024)**	• StepAdd intervention	• Not applicable	33	24.2	61.5 (9.4)
**Tabak (2013, 2014a, 2014b, 2014c)**<br>**Wieringa (2011)**	Tabak2013&2014a:<br>• Activity Coach intervention<br>Tabak2014b:<br>• Condition Coach (Activity Coach + teleconsultation + web-based exercising + self-management support)	Tabak2013&2014a:<br>• Not applicable<br>Tabak2014b:<br>• Usual care	Tabak2013: 21<br>Tabak2014a: 15<br>Tabak2014b: 29	Tabak2013: 66.7<br>Tabak2014a: 40.0<br>Tabak2014b:<br>IG: 50.0<br>CG: 50.0	Tabak2013:<br>63.5 (9.6)<br>Tabak2014a:<br>66.0 (9.2)<br>Tabak2014b:<br>IG: 64.1 (9.0)<br>CG: 62.8 (7.4)
**vanderWeegen (2013, 2015)**<br>**Verwey (2014a, 2014b)**	• Group 1: Tool (It's LiFe monitoring and feedback tool + Self-Management Support Program (SSP)<br>• Group 2: Self-Management Support Program (SSP)	• Group 3: Care as usual	199	G1: 52.3<br>G2: 47.0<br>G3: 54.4	G1: 57.5 (7.0)<br>G2: 56.9 (8.3)<br>G3: 59.2 (7.5)

**vanGenugten (2010, 2012, 2014)**	• Tailored information (TI) website GRIPP	• Generic Information (GI) website (main components were 3 modules with general information on weight gain prevention with similar content and similar reminders as TI group)	539	69.1	47.8 (9.4)
**Watson (2012)**	• Pedometer + ActiHealth website + Virtual Coach Program	• Pedometer + ActiHealth website	70	IG: 89.0<br>CG: 80.0	IG: 44.1 (Not reported)<br>CG: 40.6 (Not reported)
**Yom-Tov (2017)**	• Text messaging intervention with reinforcement learning algorithm	• Unchanging one-weekly reminders to exercise	27	IG: 40.0<br>CG: 14.3	IG: 58.7 (2.1)<br>CG: 55.1 (3.6)
**Zahedani (2023)**	• Season of Me program (4-weeks with and 8-weeks without CGM)	• Not applicable	1066, consisting of normoglycemic individuals (n = 746), individuals with prediabetes (n = 206), and individuals with T2D (n = 94)	51.0	49.0 (11.5)
