# Supplementary material for: Systematic review of dynamically tailored eHealth interventions targeting physical activity and healthy diet in chronic disease
Source: NPJ Digit Med. 2025 Nov 19;8:696. doi: 10.1038/s41746-025-02054-7 (PMC12630729; doi:10.1038/s41746-025-02054-7)
Supplement: Supplementary file 7 — Supplementary data6 [file 41746_2025_2054_MOESM7_ESM.pdf]

Supplementary Data 6. Study Findings

Author (year)	Outcome type	Results[1]
**Almeida (2015)**<br>**Estabrooks (2011)**	Effects on health behavior<br>(Almeida 2015)	**Weekly minutes of moderate and vigorous physical activity (PA)**<br>Main effect of time: A significant increase in PA was observed across all participants between baseline and the 1-month follow-up (F1,441 = 30.03, p < 0.001). A time-by-condition interaction was observed (F3,441 = 8.33, p < 0.001).<br>Environmental (ENV): Baseline M 65.76 (SE 7.04); 1 month M 74.04 (SE 8.37). Increase in PA: M +8.3 (SD 8.1).<br>No significant difference compared to Control (no p-value reported).<br>Social Cognitive (SC): Baseline M 63.38 (SE 7.30); 1 month M 108.46 (SE 8.69). Increase in PA: M +45.1 (SD 10.2).<br>Significant improvement compared to Control (p = 0.004) and Environmental (p < 0.05).<br>Combination (COMBO): Baseline M 56.25 (SE 6.72); 1 month M 109.71 (SE 7.99). Increase in PA: M +53.4 (SD 8.9).<br>Significant improvement compared to Control (p < 0.001) and Environmental (p = 0.003). There was no difference between COMBO and SC.<br>Control (CON): Baseline M 80.49 (SE 7.37); 1 month M 77.97 (SE 8.77). Decrease in PA: M -2.5 (SD 10.8).
	Effects on health behavior<br>(Almeida 2015)	**The percentage (%) of participants meeting public health recommendations**<br>At 1-month follow-up:<br>Environmental (ENV): 15.9%<br>Social Cognitive (SC): 27.6%<br>Combination (COMBO): 29.0%<br>Control (CON): 15.5%<br>Significant differences: Both SC and COMBO groups had significantly higher percentages of participants meeting recommendations compared to the control (CON) group (χ^2 = 10.343, p = 0.016), while the ENV group did not significantly differ from the CON group.
	Use/adherence/engagement<br>(Almeida 2015)	**The proportion of participants (%) who completed the automated 1-month follow-up phone calls (IVR)**<br>91.8% (415/452) of participants completed the 1-month IVR calls. 8.2% (37/452) of participants did not complete the 1-month IVR call, but none actively withdrew from the study.<br>Environmental (ENV): 96.5% completion rate.<br>Social Cognitive (SC): 83% completion rate. Significantly lower than the other groups ($F_{1,3}$ = 5.254, p < 0.001).<br>Combination (COMBO): 92.8% completion rate.<br>Control (CON): 94.3% completion rate. No significant differences observed between groups other than SC.
**Ambeba (2015)**<br>**Bizhanova (2023)**<br>**Burke (2017, 2020, 2022a, 2022b)**<br>**Cheng (2023)**<br>**Kariuki (2023)**<br>**Wang (2012)**	Effects on health behavior<br>(Cheng 2023)	**Changes in Healthy Eating Index (HEI)-2015 scores**<br>HEI-scores increased in both groups at 6 months (SM+FB: +2.3, SM: +2.2), with scores stabilizing or slightly declining by 12 months.<br>Participants with ≥5% weight loss had higher HEI-2015 scores at 6 months (67.46 [65.27, 70.12] vs. 62.41 [60.26, 63.94]) despite similar HEI-2015 scores at baseline (62.18 (59.47, 65.31) vs. 62.06 (59.77, 63.67).<br>Differences diminished by 12 months: ≥ 5 % weight loss: 65.38 (62.87, 67.97) vs. < 5 % weight loss: 62.72 (60.28, 64.43).
	Effects on weight<br>(Burke 2022a+b)	**Change in weight (in % and kg) from baseline to 6 months and 12 months (SM+FB versus SM)**<br>On average, both groups had statistically significant weight loss over 12 months (b6 months=–2.94, 95% CI –3.70 to –2.19; b12 months=–2.34, 95% CI –3.10 to –1.59; F= 61.46; p < 0.001).<br>6 months: No significant difference between groups for percentage weight loss (mean difference = −0.04%, 95% CI: −0.99% to 0.91%, p = 0.940) or absolute weight loss (mean difference = 0.04 kg, 95% CI: −0.99 to 0.91 kg, p = 0.869) at 6 months.<br>12 months No significant difference between groups in percent (mean difference = –0.27%; 95% CI –1.57% to 1.03%; t=–0.41; p = 0.68) or absolute weight change (2-sample t test=0.67; p = 0.50) at 12 months.

	Effects on weight<br>(Burke 2022a)	**Change in BMI from baseline to 6 months in kg/m^2**<br>Both groups showed significant reductions in BMI from baseline to 6 months: the SM+FB group decreased by 1.21 kg/m^2 (95% CI: 0.43 to 1.99, p = 0.002), and the SM group by 1.80 kg/m^2 (95% CI: 1.02 to 2.57, p < 0.0001).<br>No significant difference between groups: mean difference = −0.59 kg/m^2, 95% CI: −1.44 to 0.26, p = 0.174.
	Effects on weight<br>(Burke 2022a+b)	**Proportion of participants achieving at least 3% or 5% weight loss compared to their baseline weight**<br><u>6 months:</u> In total, 30.1% participants lost ≥5%. No significant difference between SM+FB (31.9%) and SM (28.3%) groups (χ^2 test p = 0.381).<br>Both groups had 44.6% achieving ≥3% weight loss; no significant difference (p = 0.999).<br><u>12 months:</u> SM+FB: 26.3% vs. SM: 29.1% participants who lost ≥5% weight (X^2 = 0.49; p = 0.49).
	Effects on weight<br>(Burke 2022a)	**Reduction in waist circumference from baseline in centimeters (cm)**<br><u>Female participants:</u> SM+FB: M –3.18 cm (95% CI: –5.64 to –0.71, p = 0.012). SM: M –2.73 cm (95% CI: –5.21 to –0.25, p = 0.031).<br>No significant difference between groups (mean difference = –0.11, 95% CI: −2.87 to 2.65, p = 0.937).<br><u>Male participants:</u> SM+FB: M –3.94 cm (95% CI: –9.72 to 1.84, p = 0.179). SM: M –7.29 cm (95% CI: –11.83 to –2.75, p = 0.002).<br>No significant difference between groups (mean difference = 0.05, 95% CI: −5.53 to 5.64, p = 0.985).
	Effects on clinical health outcomes<br>(Burke 2022a)	**Group differences in blood pressure (BP) change from baseline in mmHg**<br><u>Systolic BP: SM+FB:</u> M −0.97 mmHg (95% CI: −3.77 to 1.83, p = 0.497) SM: M 1.32 mmHg (95% CI: −1.72 to 4.36, p = 0.393)<br>No significant group differences for systolic BP (mean difference: −1.21, 95% CI: −4.27 to 1.85, p = 0.438).<br><u>Diastolic BP:</u> SM+FB: M 0.23 mmHg (95% CI: −1.78 to 2.24, p = 0.823) SM: M 1.85 mmHg (95% CI: −0.24 to 3.94, p = 0.082)<br>No significant group differences for diastolic BP (mean difference: −1.46, 95% CI: −3.59 to 0.66, p = 0.177).
	Use/adherence/engagement<br>(Burke 2022a+b)	**Monthly percentage (%) of feedback messages opened by participants**<br><u>6 months:</u> Mean percentage of FB messages opened: 54.8% (SD 24.9%), range 2.2%-97.4%.<br><u>12 months:</u> Median percentage of FB messages opened: 42.19% (Q1: 39.20%, Q3: 66.48%, range: 1.28%-93.70%).<br>A 1% increase in FB messages opened was associated with 0.10 greater percent weight loss (b = –0.10; 95% CI –0.13 to –0.07; p < 0.001).
**Baert (2018)**<br>**Bohanec (2021)**<br>**Clays (2021)**<br>**Voorend (2019)**	User experiences<br>(Voorend 2019)	**User feedback (qualitative assessment)**<br>Overall, participants enjoyed using the HeartMan system as it encouraged them to do exercises and be more physically active. They found the information about diet quite useful and for those participants who interpreted HeartMan as a system for self monitoring, keeping track of their health parameters made them feel more aware of their health. The analysis showed that participants had different needs and preferences regarding self-management.
	Effects on quality of life<br>(Clays 2021)	**Health-related quality of life (HRQoL) measured using the Minnesota Living with Heart Failure Questionnaire (0-105)**<br>Intervention: M −1.0 (SD 14.4), p = 0.70.<br>Control: M +1.7 (SD 13.8), p = 0.58.<br>Between-group difference: p = 0.50 (not significant)
	Effects on clinical health outcomes<br>(Clays 2021)	**Physical capacity to perform exercise, measured by 6-min walking test (6MWT) in meters**<br>Intervention: +0.9 (SD 88.8), p = 0.96.<br>Control: +4.8 (SD 39.4), p = 0.62.<br>Between-group difference: p = 0.84 (not significant).
	Effects on clinical health outcomes<br>(Clays 2021)	**Changes in Left ventricular ejection fraction (LVEF) in percentage (%)**<br>Intervention: +3.4% (SD 7.0), p < 0.05.<br>Control: −0.4% (SD 5.8), p = 0.78.<br>Between-group difference: p = 0.08 (not significant).
**Beckie (2024)**	Usability	**Usability measured with the System Usability Scale score (max. 100)**

**Sengupta (2020a, 2020b)**		The mean score for the Her Beat group was 82.0 ± 20.6.
	Effects on health behavior<br>(Beckie 2024)	**Changes in the International Physical Activity Questionnaire (IPAQ) short form (SF) in MET·min·wk**<br>HerBeat group no significant change (M 2942.0 SD 3500.9 to M 3429.9 SD 2621.7; p = 0.548, d = −0.130).<br>E-UC group no significant change (M 3017 SD 3070.3 to M 2711.4 SD 1834.3; p = 0.664, d = 0.099).<br>No significant between-group difference at 3 months (p = 0.372, d = −0.315).
	Effects on weight<br>(Beckie 2024)	**Changes in Body Mass Index (BMI) in kg/m^2**<br>HerBeat group: No significant change (M 29.1 SD 5.5 to M 29.1 SD 5.6, p = 0.74).<br>E-UC group: No significant change (M 29.4 SD 5.9 to M 29.4 SD 6.0, p = 0.92).<br>Between groups at 3 months not significant: p = 0.84.
	Effects on weight<br>(Beckie 2024)	**Changes in waist circumference in centimeters (cm)**<br>HerBeat group: Significant improvement (M 95.1 SD 14.1 cm to M 92.1 SD 14.9 cm, p = 0.008).<br>E-UC group: No significant change (M 98.3 SD 12.9 cm to M 97.9 SD 12.6 cm, p = 0.74).<br>Between groups at 3 months: p = 0.18.
	Effects on clinical health outcomes<br>(Beckie 2024)	**Physical capacity measured by 6-min walking test (6MWT) in meters**<br>HerBeat group improved significantly (M 441.6 SD 57.7 m at baseline to M 463.7 SD 71.9 m at 3 months; p = 0.016, d = 0.558).<br>E-UC group showed no significant improvement (M 423.8 SD 92.6 m at baseline to M 425.1 SD 92.6 m at 3 months; p = 0.894, d = −0.030).<br>The between-group difference at 3 months was not statistically significant (p = 0.137, d = 0.468).
	Effects on clinical health outcomes<br>(Beckie 2024)	**Changes in systolic and diastolic blood pressure (BP) in mmHg**<br>**Systolic BP:**<br>HerBeat group: M 128.4 SD 16.9 to M 122.6 SD 16.1; not significant (p = 0.11).<br>E-UC group: M 131.1 SD 16.0 to M 129.1 SD 26.6; not significant (p = 0.35).<br>Between groups at 3 months not significant: p = 0.34.<br>**Diastolic BP:**<br>HerBeat group: M 78.5 SD 8.1 to M 74.9 SD 9.5; significant (p = 0.03).<br>E-UC group: M 78.8 SD 11.3 to M 76.9 SD 12.9; not significant (p = 0.38).<br>Between groups at 3 months not significant: p = 0.56.
**Bennett (2013, 2018)**<br>**Foley (2012, 2016)**<br>**Steinberg (2013)**	User experiences<br>(Steinberg 2013)	**Intervention Satisfaction (4-point Likert scale from strongly agree (=4) to strongly disagree (=1))**<br>Most participants strongly agreed that texting was easy (70%, 16/23) and helpful (68%, 15/22), and 76% (16/21) either somewhat or strongly agreed that the text messages helped them increase the number of daily steps walked. 71% (15/19) reported that it took less than 3 minutes to reply to texts. More than half (57%, 12/21) felt that receiving daily texts was very important and approximately three-quarters of participants (76%, 16/21) felt the frequency of texting was appropriate. 82% (18/22) were satisfied with the feedback, and 76% (16/21) agreed the frequency was appropriate.
	Effects on weight<br>(Bennett 2018)	**Changes in weight and significant weight loss (>3% or >5% of their initial weight) in kg and percentages (%)**<br>At 6 months: Mean difference= –4.4 kg, 95% CI: –5.5, –3.3 kg, p < 0.001<br>At 12 months: Adjusted mean difference= –3.8 kg, 95% CI: –5.1, –2.5 kg, p < 0.001<br>Significantly more participants in the intervention group lost >5% of initial weight at 6 and 12 months compared to usual care.<br>At 6 (43% vs 6%, estimated RR: 6.8, 95% CI: 3.6, 12.7, p < 0.001) and 12 months (40.4% vs 16.7%, estimated RR: 2.4, 95% CI: 1.6, 3.5, p < 0.001).<br>A significantly larger proportion of intervention participants lost >3% of their initial weight, relative to usual care (6 months: 56% vs 15%, estimated RR: 3.8, 95% CI: 2.5, 5.6, p < 0.001, 12 months: 55% vs 30%, estimated RR: 1.8, 95% CI: 1.4, 2.4, p < 0.001).
	Effects on weight	**Changes in waist circumference in centimeters (cm)**

	(Bennett 2018)	<u>6 months:</u><br>Within-group difference: Intervention group: –3.4 cm, 95% CI: –4.3, –2.4; usual care: 0.1 cm, 95% CI: –0.8, 1.1<br>Between-groups difference: –3.5 cm (95% CI: –4.8, –2.2, p = < 0.0001)<br><u>12 months:</u><br>Within-group difference: Intervention group: –2.9 cm, 95% CI: –4.0, –1.9; usual care: 0.6 cm, 95% CI: –0.4, 1.6<br>Between-groups difference: –3.6 cm (95% CI: –5.0, –2.1, p = < 0.0001)
	Effects on clinical health outcomes<br>(Bennett 2018)	**Changes in blood pressure in mmHg**<br>There were significant reductions in blood pressure within both study arms at all timepoints; however, levels did not differ between treatment arms.<br>**Systolic Blood Pressure (SBP)**<br><u>6 months:</u><br>Within-group difference: Intervention group: –4.6 mmHg, 95% CI: –7.5, –1.7; usual care: –3.4 mmHg, 95% CI: –6.3, –0.6<br>Between-groups difference: –1.2 mmHg (95% CI: –5.0 to 2.6, p = 0.54).<br>Between-groups difference: –1.2 mmHg (95% CI: –5.0, 2.6, p = 0.54).<br><u>12 months:</u><br>Within-group difference: Intervention group: –8.4 mmHg, 95% CI: –11.4, –5.3; usual care: –7.5 mmHg, 95% CI: –10.4, –4.5<br>Between-groups difference: –0.9 mmHg (95% CI: –4.9, 3.1, p = 0.65).<br>**Diastolic Blood Pressure (DBP)**<br><u>6 months:</u><br>Within-group difference: Intervention group: –4.1 mmHg, 95% CI: –5.9, –2.4; usual care: –2.5 mmHg, 95% CI: –4.2, –0.8<br>Between-groups difference: –1.6 mmHg (95% CI: –3.9, 0.7, p = 0.16).<br><u>12 months:</u><br>Within-group difference: Intervention group: –5.2 mmHg, 95% CI: –7.1, –3.3; usual care: –4.2 mmHg, 95% CI: –6.1, –2.4<br>Between-groups difference: –1.0 mmHg (95% CI: –3.5, 1.5, p = 0.43).
	Effects on clinical health outcomes<br>(Bennett 2018)	**Changes in HbA1c levels in percentages (%)**<br>Within group differences: Intervention group: -0.3%, 95% CI: -0.5, -0.2; usual care: -0.2% 95% CI: -0.04, -0.001<br>Between-groups difference: –0.2% (95% CI: –0.4, 0.04, p = 0.11). There were no between-group differences in HbA1c.
	Effects on clinical health outcomes<br>(Bennett 2018)	**Changes in cholesterol levels (total cholesterol, HDL, LDL and triglycerides) in mg/dL**<br>The intervention appears to have had a limited effect on total cholesterol, LDL, and triglycerides. A significant improvement was seen in HDL cholesterol after 12 months.<br><u>Total Cholesterol:</u> Between-groups difference: 3.1 mg/dL (95% CI: –4.7, 10.9, p = 0.44).<br><u>LDL Cholesterol:</u> Between-groups difference: –3.2 mg/dL (95% CI: –10.5, 4.1, p = 0.39).<br><u>HDL Cholesterol:</u> Between-groups difference: 3.5 mg/dL (95% CI: 1.1, 5.9, p = 0.005).<br><u>Triglycerides:</u> Between-groups difference: 6.8 mg/dL (95% CI: –14.0, 27.6, p = 0.52).
	Use/adherence/engagement<br>(Bennett 2018)	**Intervention use in percentages (%)**<br>Median weekly self-monitoring completion: 93.2% (IQR 54%-100%).<br>Median weekly coaching call completion: 89% (IQR 50%-100%).<br>Participants weighed themselves a median of 2.8 days/week (IQR 1.2-4.5) (42.9% of expected days, SD 28.4%).
	Use/adherence/engagement<br>(Steinberg 2013)	**Completion rates of self-monitoring via text messaging and behavioral adherence to goals during the intervention in percentages (%)**<br>Daily adherence rate as a percentage (M 49%, SD 28, IQR 27%-78%).

		Daily goal attainment score (M 6.3, SD 2.8, IQR 4.0-8.2).<br>Steps reported per day (M 4994, SD 2741, IQR 3016-6489).<br>No significant correlation between average goal attainment score and text messaging adherence (r= 0.24; p = 0.25) or mean number of steps reported and text messaging adherence (r= 0.33; p = 0.11).
**Bond (2014)**<br>**Thomas (2015)**	User experiences<br>(Bond 2014)	**Acceptability of the intervention (ratings on a 5-point Likert scale, reported in % of participants)**<br>90% (27/30) of participants rated the intervention as significantly increasing their motivation and reducing sedentary behavior (Likert scores of 4 or 5). Preference for break conditions: Most preferred: 6-min (56.7%), followed by 3-min (33.3%), and 12-min (10.0%). Least preferred: 12-min (53.3%) and 3-min (46.7%).
	Effects on health behavior<br>(Bond 2014)	**Change in daily time spent in sedentary behavior in percentage of waking hours (%)**<br>Percent time spent in sedentary behavior was significantly decreased in all 3 physical activity break conditions relative to baseline (p < 0.005).<br>Pairwise comparison: 3-min breaks reduced sedentary time more than 12-min breaks (p = 0.04).<br>Baseline: 72.2% (95% CI: 68.5; 76.0).<br>3-min: 66.3% (95% CI: 61.7; 71.0).<br>6-min: 66.6% (95% CI: 61.5; 71.7).<br>12-min: 69.0% (95% CI: 64.7; 73.2).
	Effects on health behavior<br>(Bond 2014)	**Change in daily time spent in MVPA in percentage of waking hours (%)**<br>Percent time in MVPA increased significantly in all conditions (p < 0.01).<br>Baseline: 5.0% (95% CI: 3.6; 6.3).<br>3-min: 7.0% (95% CI: 5.4; 8.7).<br>6-min: 6.7% (95% CI: 5.0, 8.4).<br>12-min: 6.4% (95% CI: 4.7; 8.1).
	Use/adherence/engagement<br>(Thomas 2015)	**The extent to which participants carried the smartphone during the intervention period in days and hours per day**<br>Participants carried the smartphone for an average of 6.90 days (SE 0.04, 95% CI [6.81, 6.99]) per 7-day condition and 14.94 hours per day (SE 0.48, 95% CI [13.96, 15.91]). No significant differences were observed between conditions (e.g., p = 0.534 for days and p = 0.260 for hours/day).<br>No trend in smartphone use over time was detected (p = 0.225).
**Boudreau (2016)**<br>**Moreau (2015)**	Usability<br>(Moreau 2015)	**Usability testing in two phases**<br>Over 40 improvements were made, ranging from interface redesigns to reducing complexity and streamlining the user experience.
**Buchan (2020)**	User experiences	**Semi-structured interviews on the acceptability and usability of the Onitor Track and app**<br>Participants reported using wearables and health apps primarily for weight loss and fitness, with motivation often driven by specific events. Step counting, calorie tracking, and exercise monitoring were commonly highlighted as useful features. The Onitor Track was less favored compared to other devices due to its lack of instant feedback. Technical issues affected some users' activity levels. Diet changes were widely reported, with mixed views on the low-carb approach. Facebook group interactions provided social support for some, but others found it less engaging due to its artificial nature.
	Effects on weight	**Change in body weight in kg**<br>Median weight reduction: −2.7 kg (IQR −1.6 to −3.3).<br>Significant difference between baseline and week 4 (p = 0.018).
	Effects on weight	**Change in Body Mass Index (BMI) in kg/m^2**<br>Median BMI reduction: −1.1 (IQR −0.6 to −1.2).<br>Significant difference between baseline and week 4 (p = 0.018).
	Effects on weight	**Change in waist circumference (WC) in centimeters (cm)**

		Median WC reduction: −4 cm (IQR −2.5 to −5.5).<br>Significant difference between baseline and week 4 (p = 0.027).
	Use/adherence/engagement	**App and device usage in number of times, hours, and days**<br>Onitor Track usage and app engagement decreased each week, with participants wearing the device for a median of 4 days (2–14.5 days) and opening the app 25 times (18–81 times) over 3 weeks. Self-monitoring and app use dropped by week 4.<br><u>App usage (total hours over 3 weeks):</u> Median 126 h (IQR 42.5–169.5)<br><u>App sessions (continuous use periods over 3 weeks):</u> Median 22 (IQR 11–47)<br><u>App opened (total times over 3 weeks):</u> Median 25 (IQR 18–81) Days test unit worn (over 3 weeks): Median 4 days (IQR 2–14.5)<br><u>Test unit time worn (hours/day):</u> Median 13.9 h (IQR 8.1–14.9 h)
**Chokshi (2017)**	User experiences	**Self-reported healthcare utilization and patient experience**<br>Participants in both arms agreed that the study helped them to increase their PA levels (incentive arm: 80.0% vs. control arm: 81.0%) and to improve their cardiac health (incentive arm: 84.4% vs. control arm: 88.2%). A slightly higher percentage in the incentive arm were satisfied with their experiences with the intervention compared to the control arm (incentive arm: 80.0% vs. control arm: 72.9%). More participants in the incentive group reported they would continue using the wearable device (incentive arm: 83.8% vs. control arm: 5.8%).
	Effects on health behavior	**Change in mean daily steps**<br>The adjusted differences in mean daily steps between the incentive and control arms were (between-group differences):<br>Ramp-up phase: +1061 steps (95% CI: 386–1736, p < 0.01).<br>Maintenance phase: +1368 steps (95% CI: 571–2164, p < 0.001).<br>Follow-up phase: +1154 steps (95% CI: 282–2027, p < 0.01).<br>In the fully adjusted model, the effects remained significant in the maintenance (p < 0.01) and follow-up phase (p = 0.03), but not in the ramp-up phase (p = 0.06) (between-group differences).
**Collins (2010, 2012, 2013)**	Effects on health behavior (Collins 2013)	**Change in total physical activity measured in MET minutes per week (enhanced versus basic)**<br><u>12 weeks:</u><br>Absolute difference between groups: 96.19 (-424.3, 616.66), p = 0.72<br><u>24 weeks:</u><br>Absolute difference between groups: 358.58 (-285.1, 1002.3), p = 0.27<br>Within-group significance: Not significant in either group.<br>Between-group significance: No significant difference between groups.
	Effects on health behavior (Collins 2013)	**Change in daily energy intake in kcal**<br><u>12 weeks:</u><br>Difference: 267.74 (-186.4, 721.90), p = 0.25<br><u>24 weeks:</u><br>Difference: 18.23 (-439.2, 475.66), p = 0.94<br>Within-group differences: Not reported.<br>Between-group differences: No significant difference between groups.
	Effects on weight (Collins 2013)	**Change in body weight in kg**<br><u>12 weeks:</u><br>Difference: 0.6 kg (-0.3, 1.6), p = 0.21<br>Within-group significance: Both groups showed a significant reduction from baseline.<br>Between-group significance: No significant difference between groups.

		24 weeks:<br>Difference: 0.7 kg (-0.6, 2.0), p = 0.27<br>Within-group significance: Both groups showed a significant reduction from baseline.<br>Between-group significance: No significant difference between groups.
	Effects on weight<br>(Collins 2013)	**Change in Body Mass Index (BMI) in kg/m^2**<br>12 weeks:<br>Difference: 0.2 (-0.1, 0.5), p = 0.28<br>Within-group significance: Both groups showed a significant reduction from baseline.<br>Between-group significance: No significant difference between groups.<br>24 weeks:<br>Difference: 0.2 (-0.2, 0.6), p = 0.29<br>Within-group significance: Both groups showed a significant reduction from baseline.<br>Between-group significance: No significant difference between groups.
	Effects on weight<br>(Collins 2013)	**Change in waist circumference at the umbilical level in cm**<br>12 weeks:<br>Difference: 0.2 (-0.9, 1.3), p = 0.73<br>Within-group significance: Both groups showed a significant reduction from baseline.<br>Between-group significance: No significant difference between groups.<br>24 weeks:<br>Difference: 0.9 (-0.5, 2.3), p = 0.22<br>Within-group significance: Both groups showed a significant reduction from baseline.<br>Between-group significance: No significant difference between groups.
	Effects on clinical health outcomes<br>(Collins 2013)	**Secondary outcomes blood pressure, LDL, HDL, triglycerides, total cholesterol, glucose, insulin in mmHg, mmol/L, mIU/L**<br>No significant changes in clinical outcomes between enhances and basic group.
	Use/adherence/engagement<br>(Collins 2013)	**Total number of days participants accessed the intervention website**<br>12 weeks:<br>Difference: 9.45 (3.34, 15.56), p = 0.002<br>24 weeks:<br>Difference: 12.47 (4.73, 20.20), p = 0.002<br>Within-group differences: Not reported<br>Between-group differences: Significant difference in favor of the enhanced group at both time points (p = 0.002).
**Daryabeygi-Khotbehsara (2022, 2023)**	User experiences<br>(Daryabeygi-Khotbehsara 2022)	**Qualitative experiences with iMove among type 2 diabetes patients**<br>The focus group results highlighted that participants appreciated multifunctional health apps with reminders but disliked complicated sign-in processes and were concerned about data privacy. For iMOVE, they liked the home tab's donut chart but had concerns about privacy on the profile tab. While the app was generally easy to navigate, the activity tab and Bluetooth functionality were unclear. Based on this feedback, modifications were made, including adding a goal-setting tab, adjusting colors, and improving clarity in the activity tab and Bluetooth connectivity.
**Dorsch (2018, 2020)**	User experiences<br>(Dorsch 2020)	**App experiences measured in a survey**

		In a survey of 24 app users, 79% found the app useful and applied its information in their daily lives, while 71% considered the information important. Additionally, 83% agreed the app was easy to use. However, 13% found it confusing, and only 4% reported difficulty in understanding it.
	Effects on health behavior<br>(Dorsch 2020)	**Dietary sodium intake (ASA24) (an electronic 24-hour recall)**<br>App group showed a greater reduction in sodium intake than the No App group (-1537 mg vs. -233 mg, p = 0.07)
	Effects on clinical health outcomes<br>(Dorsch 2020)	**Sodium excretion measured through urine collection over 24 hours (mg/day)**<br>App group had a greater reduction in sodium excretion (-637 mg) than the No App group (-322 mg, p = 0.47)
	Effects on clinical health outcomes<br>(Dorsch 2020)	**Estimate of 24-hour sodium excretion based on a fasting morning urine sample (Kawasaki formula, mg/day)**<br>App group had a reduction of -462 mg vs. an increase of +381 mg in the No App group (p = 0.03).
	Effects on clinical health outcomes<br>(Dorsch 2020)	**Change in systolic blood pressure (BP) in mmHg**<br>Systolic BP decreased by 7.5 mmHg in the App group compared to 0.7 mmHg in the No App group (p = 0.12).
	Use/adherence/engagement<br>(Dorsch 2020)	**App usage in number of interactions**<br>App users received a median of 126 notifications; scanning activity was sustained throughout the study.
**Finkelstein (2015)**	User experiences	**Acceptance of the mobile app and willingness to use it in the future**<br>Majority expressed high acceptance and indicated future use of the mobile app
	Effects on health behavior	**Prolonged inactivity episodes (percentage of inactivity over 2-hour slots)**<br>Inactivity was lower during “message-on” periods (24.6%) compared to “message-off” (30.4%) (p < 0.02).<br>Group A showed a significant reduction (p < 0.004), while Group B showed no change (p = 0.98).
	Effects on health behavior	**Average daily number of steps taken by participants**<br>Non-significant changes in step counts (within-group).<br>Group A had more steps during "message-on" (M 6199.1 [SD 2062.1]) vs "message-off" (M 5614.8 [SD 1855.1]), while Group B showed no relevant changes during "message-on" (M 5699.4 [SD 2224.4]) vs "message-off" (M 5771.4 [SD 2552.4]).
**Forman (2019a, 2019b) Goldstein (2017, 2020, 2021a, 2021b)**	User experiences<br>(Forman 2019b)	**Satisfaction and acceptability (Scores from 1 (Strongly Disagree) to 7 (Strongly Agree); Weight Watchers (WW) versus WW + OnTrack (OT))**<br><u>WW:</u> M 4.70, SD 1.52.<br><u>WW + OT:</u> M 4.46, SD 1.38, t(133) = −0.96, p = 0.34.<br>OT interventions were rated as helpful and accurate 72.8% of the time.<br>Higher satisfaction in BTS group for WW + OT (M 4.99, SD 1.12) than FS (M 4.19, SD 1.43), t(71.79) = 2.91, p = 0.005, d = 0.62.<br>This difference was not observed among WW participants only: BTS M 5.11 (SD 1.30), FS M 4.54 (SD 1.60); t(43) = 1.14, p = 0.26.
	Effects on health behavior<br>(Forman 2019b)	**Frequency of lapses during intervention**<br><u>WW + OT:</u> M 29.72 lapses (SD 29.11), decreased significantly over the study period, F(5.49,621.14) = 4.48, p < 0.001, ηp^2 = 0.04.<br>No interaction with diet type, F(5.46,611.70) = 0.98, p = 0.43, ηp^2 = 0.01.
	Effects on weight<br>(Forman 2019b)	**Percentage (%) weight loss moderated by diet type**<br>No main effect of treatment condition, F(1,173) = 0.15, p = 0.70, but statistically significant moderated by diet type, F(1,173) = 9.68, p = 0.002, ηp^2 = 0.05.<br><u>WW Beyond the scale (BTS) group:</u> WW + OT (M 4.7%, SE 0.55) vs. WW (M 2.6%, SE 0.80).<br><u>WW Freestyle (FS) group:</u> WW + OT (M 2.9%, SE 0.38) vs. WW (M 4.5%, SE 0.52).

	Use/adherence/engagement<br>(Forman 2019b)	**OnTrack compliance (percentage of completed lapse trigger entries) and intervention access (percentage of alerts and full interventions accessed)**<br>Compliance: 62.9% (SD 28.11) of lapse trigger entries completed.<br>Risk alerts: M 118.5 (SD 52.77). 46.9% (SD 26.18) opened. This declined over time, $F(7,581) = 3.50$, $p = 0.001$, $\eta p^2 = 0.04$.<br>Full interventions: 7.4% accessed (SD 10.10), stable over time ($F[7, 175] = 0.56$, $p = 0.67$, $\eta p2 = 0.02$).<br>Full interventions most frequently accessed: “Goal setting” targeting low confidence (opened on 46.8% of the occasions delivered), “Accepting urges/cravings” targeting boredom (44.4% of occasions), “Get support” targeting low confidence (35.6% of occasions), and “Motivation Mantra” targeting low motivation (33.9% of occasions).
**Gatwood (2020)**	User experiences	**Evaluation of the content, tone, and clarity of messages designed for diabetes patients (qualitative feedback from 16 participants)**<br>Participants favored messages with a positive tone, simple language, clear actions, and relatable examples. Messages that were overly complex or had a blaming tone were received negatively.
**Golbus (2024)**<br>**Hellem (2023)**	User experiences<br>(Hellem 2023)	**Comparison of researcher versus community-generated notifications (qualitative feedback)**<br>Community-generated notifications enhanced authenticity and relatability. FQHC participants emphasized community, unmet social needs, and religiosity, while university participants highlighted work and resource access.
**Gupta (2015)**	User experiences	**User satisfaction and feedback on "Let’s Exercise" app (5-point Likert scale (from "strongly agree" to "strongly disagree")**<br>84.8% of users were satisfied, 60.6% would continue using the app, and suggestions included meditation and offline functionality.
**Hamborg (2024)**<br>**Martens Anderson (2022)**	User experiences<br>(Hamborg 2024)	**Participants’ perceptions of integrating physical activity post-cardiovascular event with the FAIR intervention (qualitative feedback)**<br>Participants valued the FAIR intervention as a bridge to independent physical activity. Text messages and action plans provided motivation and support. However, some participants desired more personal interaction and group-based activities for sustained support.
**Hemnes (2021)**<br>**Martin (2015)**	User experiences<br>(Martin 2015)	**Participant satisfaction with the mActive intervention (mean scores)**<br>Participants assigned the activity tracker a mean score of 4.0 of 5.0, and text messages 3.8 of 5.0 (4=good; 5=great).
	Effects on quality of life<br>(Hemnes 2021)	**Change in QoL scores measured with the emPHasis-10 score (0-50) and SF-36 score (0-100)**<br><u>emPHasis:</u> Significant improvement in QoL in the intervention group according to emPHasis-10 (–4.2 points; p = 0.046).<br><u>SF-36 MCS:</u> Intervention: Adjusted difference at week 12: +3.53 (p = 0.14)<br><u>SF-36 PCS:</u> Intervention: Adjusted difference at week 12: –1.6 kg (p = 0.36)
	Effects on health behavior<br>(Hemnes 2021)	**Difference in average daily steps**<br>Significant change in average steps in intervention versus control (week 12 vs. baseline): Adjusted difference at week 12: +1,250 steps (p = 0.03).<br>Final adjusted difference at day 84: +1,103 steps (95% CI: +522 to +1,683; p < 0.001).
	Effects on health behavior<br>(Hemnes 2021)	**Moderate-to-vigorous physical activity (MVPA) in minutes per day**<br>Adjusted difference at week 12 for intervention versus control: +5.9 min (p = 0.05).
	Effects on weight<br>(Hemnes 2021)	**Change in total body weight in kg**<br>No significant change: Adjusted difference at week 12 for intervention versus control: –0.3 kg (p = 0.70)
	Effects on clinical health outcomes<br>(Hemnes 2021)	**Change in 6-Minute Walk Distance (6MWD) in meters**<br>No significant change between groups (model estimated difference at week 12: +14 m; p = 0.52).
	Use/adherence/engagement<br>(Hemnes2021)	**The proportion of days participants met predefined wear-time thresholds (>10 hours/day or >100 steps/day) in percentages and hours**<br><u>Compliance:</u><br>Participants met the stringent compliance threshold (>10 h/day) on 87.7% of days, with no significant differences between groups. A broader compliance definition (>100 steps/day) was met on 93.4% of days, also with no group differences.<br><u>Wear time:</u>

		No baseline difference between the intervention and control groups (104.5 SD 30.7 h vs. 113.3 SD 14.8 h; p = 0.79). At week 12, wear time was significantly higher in the intervention group (112.2 SD 29.3 h vs. 103.8 SD 21.5 h; p = 0.04).
**Hietbrink (2023a, 2023b)**	User experiences (Hietbrink 2023b)	**Acceptability of the E-supporter (qualitative feedback)**<br>Many participants preferred E-supporter as part of blended care. Some found it less effective than in-person support, while others appreciated the daily guidance and reminders. Participants generally found the messages helpful but felt they were sometimes repetitive or not personalized enough. Most found the messaging frequency appropriate, with mixed views on duration. E-supporter increased motivation and lifestyle awareness, with examples of reduced salt intake and weight loss for some participants.
	Effects on health behavior (Hietbrink 2023b)	**Physical activity measured in steps per day**<br>Daily step count significantly improved postintervention (median increase from 6426 [IQR 2908.5-6811.5] to 8131 steps [IQR 4368.25-9855.5]; p = 0.04).
	Effects on health behavior (Hietbrink 2023b)	**Changes in DHD15 index score (0-120)**<br>Improvements in dietary scores were observed among participants in the healthy diet module.<br>Percent improvements of 24.4% and 22.3% in DHD-15 index.
	Use/adherence/engagement (Hietbrink 2023a)	**Intervention usage in percentages (%)**<br>78% of the participants experienced problems receiving the 2 daily motivational messages; 11% participant received no messages at all, which could be explained by a human error made by the researcher. 97.1% of the messages were read. Participants liked 73.8% and disliked 12.1% of the content of the motivational messages. 43% (range 0%-100%) of the psychological exercises were completed.
**Khunti (2021)**<br>**Morton (2015)**<br>**Yates (2015)**	User experiences (Morton 2015)	**User feedback on the content and structure of Walking Away Plus (WAP) (qualitative feedback)**<br>The piloting phase indicated that (1) the structure of the follow-on support (including the brief telephone call) was acceptable, (2) the frequency of text messages over the 8-week pilot phase was acceptable but should be reduced over time, (3) the content and language used in the text messages were acceptable, (4) minor technical issues needed to be resolved, and (5) participant instructions in both the Walking Away session and the follow-on support booklet required refinement.
	Effects on quality of life (Khunti 2021)	**Quality of life measured with SF-8, EQ-5D-5L, VAS**<br>No statistically significant changes over time between WA vs. control or WAP vs. control in all QoL measures.
	Effects on health behavior (Khunti 2021)	**Total ambulatory activity (steps/day)**<br><u>12 months:</u><br>WA increased by +264 (97.5% CI: -70, 597) steps/day relative to control (not significantly)<br>WAP increased by +547 (97.5% CI: 211, 882) steps/day relative to control (significantly)<br><u>48 months:</u><br>No significant difference between groups (WA vs. control: +91 [97.5% CI: -282, 463], WAP vs. control: 121 [97.5% CI: -290, 532])
	Effects on health behavior (Khunti 2021)	**Time spent in moderate-to-vigorous physical activity (MVPA) in minutes per day**<br><u>12 months:</u><br>WA increased by +1.3 min/day (97.5% CI: -1.7, 4.3) (not significantly)<br>WAP increased by +3.5 min/day (97.5% CI: 0.6, 6.5) compared to control (significantly)<br><u>48 months:</u><br>No significant differences between groups (WA vs. control: 0.5 [-2.8, 3.7], WAP vs. control: 1.6 [-1.9, 5.0])
	Effects on weight (Khunti 2021)	**Changes in body weight in kg**<br><u>12 months:</u> Significant improvement in WA group vs. control<br>WA vs. control: -0.60 kg (CI: -1.18, -0.03)<br>WAP vs. control: -0.05 kg (CI: -0.62, 0.52)

		<u>48 months:</u> Significant improvement in WA group vs. control<br>WA vs. control: -1.00 kg (CI: -1.92, -0.07)<br>WAP vs. control: -0.23 kg (CI: -1.16, 0.70)
	Effects on weight<br>(Khunti 2021)	**Changes in abdominal circumference in cm**<br><u>12 months:</u> Significant improvement for WA vs. control<br>WA vs. control: -1.28 cm (CI: -2.18, -0.38)<br>WAP vs. control: -0.47 cm (CI: -1.39, 0.45)<br><u>48 months:</u> Significant improvement for WA vs. control<br>WA vs. control: -1.57 cm (CI: -2.70, -0.45)<br>WAP vs. control: -1.09 cm (CI: -2.33, 0.15)
	Effects on clinical health outcomes<br>(Khunti 2021)	**Changes in cholesterol over time measured in mmol/L**<br>**Total cholesterol (mmol/l)**<br>12 months: No significant differences<br>WA vs. control: –0.04 mmol/l (97.5% CI: –0.15, 0.06)<br>WAP vs. control: –0.08 mmol/l (97.5% CI: –0.18, 0.03)<br>48 months: No significant differences<br>WA vs. control: 0.02 mmol/l (97.5% CI: –0.11, 0.15)<br>WAP vs. control: –0.02 mmol/l (97.5% CI: –0.16, 0.11)<br>**HDL cholesterol (mmol/l)**<br>12 months: No significant differences<br>WA vs. control: 0.00 mmol/l (97.5% CI: –0.03, 0.04)<br>WAP vs. control: 0.01 mmol/l (97.5% CI: –0.03, 0.05)<br>48 months: No significant differences<br>WA vs. control: 0.00 mmol/l (97.5% CI: –0.03, 0.04)<br>WAP vs. control: 0.04 mmol/l (97.5% CI: –0.01, 0.08)<br>**LDL cholesterol (mmol/l)**<br>12 months: No significant differences<br>WA vs. control: –0.02 mmol/l (97.5% CI: –0.12, 0.07)<br>WAP vs. control: –0.04 mmol/l (97.5% CI: –0.13, 0.05)<br>48 months: No significant differences<br>WA vs. control: 0.03 mmol/l (97.5% CI: –0.08, 0.15)<br>WAP vs. control: 0.00 mmol/l (97.5% CI: –0.11, 0.12)<br>**Triglycerides (mmol/l)**<br>12 months: Significant differences for WAP vs. control<br>WA vs. control: –0.09 mmol/l (97.5% CI: –0.25, 0.06)<br>WAP vs. control: –0.15 mmol/l (97.5% CI: –0.29, –0.01)<br>48 months: No significant differences<br>WA vs. control: –0.07 mmol/l (97.5% CI: –0.18, 0.03)<br>WAP vs. control: –0.11 mmol/l (97.5% CI: –0.21, 0.00)
	Effects on clinical health outcomes	**Changes in HbA1c over time measured in mmol/mol**<br><u>12 months:</u> No significant differences

	(Khunti 2021)	WA vs. control: -0.14 mmol/mol (97.5% CI: -0.47, 0.20)<br>WAP vs. control: -0.10 mmol/mol (97.5% CI: -0.43, 0.23)<br>48 months: No significant differences<br>WA vs. control: -0.13 mmol/mol (97.5% CI: -0.68, 0.42)<br>WAP vs. control: -0.01 mmol/mol (97.5% CI: -0.63, 0.61)
	Use/adherence/engagement<br>(Khunti 2021)	**Intervention engagement and adherence (percentage of participants)**<br>High initial engagement with 80% attendance at the first session. Participants reported ease of use and interest in the intervention.<br>48 months goal setting (most or some time)<br>WAP: 78.8%<br>WA: 73.0%<br>Control: 60.7%<br>48 months pedometer use (most or some time)<br>WAP: 64.2%<br>WA: 49.7%<br>Control: 19.6%<br>48 months physical activity log use (most or some time)<br>WAP: 40.9%<br>WA: 30.6%<br>Control: 17.5%
**Kim (2024)**<br>**Park (2024)**	Usability<br>(Park 2024)	**Intervention usability measured with the System Usability Scale (SUS, range 0-100)**<br>SUS score 66.25<br>75% would continue using the app, with varied ease and confidence levels.<br>Satisfaction rated 8.06/10.
	User experiences<br>(Park 2024)	**Participant reflections on self-care, motivation, and family support (qualitative feedback)**<br>Participants reported increased self-reflection, a sense of duty for self-care, support from family, and motivation from feedback, though some frustration over self-management challenges. Messages and facilitator contact helped to improve self-care.
	Effects on health behavior<br>(Park 2024)	**Physical activity (MET-min/week)**<br>IG: 2229.26 (SD 1244.87) to 3384.74 (SD 1491.93)<br>CG: 2429.92 (SD 1791.73) to 2964.38 (SD 1164.43) p = 0.247 (Not significant)<br>No significant between-group differences in physical activity levels.
	Effects on health behavior<br>(Park 2024)	**Percentage of recommended food intake (% RFI) for different food groups**<br>Significant between-group difference for grains (p = 0.024). No significant differences for other dietary intake variables.<br>Grains<br>IG: 144.7% to 112.3%<br>CG: 136.3% to 157.3% p = 0.024 (Significant)<br>Protein Foods<br>IG: 127.7% to 107.2%<br>CG: 113.4% to 133.4% p = 0.239 (Not significant)<br>Vegetables IG: 70.5% to 63.8%<br>CG: 69.5% to 61.3% p = 0.932 (Not significant)

		<u>Fruits</u><br>IG: 32.1% to 35.2%<br>CG: 36.2% to 52.6% p = 0.591 (Not significant)<br><u>Dairy</u><br>IG: 8.5% to 28.2%<br>CG: 6.2% to 8.6% p = 0.071 (Not significant)
	Effects on clinical health outcomes<br>(Park 2024)	**Changes in HbA1c in percentages (%)**<br>IG: 8.3% (SD 0.9) to 7.4% (SD 0.8)<br>CG: 8.2% (SD 1.1) to 8.0% (SD 1.2), p = 0.029 (Significant)<br>Significant between-group difference for HbA1c (p = 0.029).
	Use/adherence/engagement<br>(Park 2024)	**Participant engagement rates (number of activities per week)**<br>Weekly averages: exercise (4.61), diet (5.77), BG monitoring (4.90), medication (5.62), overall self-care (5.22 days per week).
**Korinek (2018)**	User experiences	**Participant perceptions of app goals, usability, and continuation interest (interviews and surveys)**<br>Participants liked receiving different daily goals (100%), perceived the app as easy-to-use (85%) and expressed interest in continued app use (88%). The most common problem was sync lag with Fitbit.
	Effects on health behavior	**Step count increase in steps per day (Model 1: Linear Mixed-Effects Model)**<br>Average increase of 2650.9 steps/day (SE 405.03, p < 0.001) from baseline to intervention; minor, non-significant decrease across cycles.
	Effects on health behavior	**Step count trajectory in steps per day (Model 2: Quadratic Mixed-Effects Model)**<br>Baseline of 5301 steps. Increase of 1505.8 (SE 272.62, p<0.001) steps/day up from baseline to cycle 1. Average participant hits a ceiling for increasing steps near cycle 3, followed by a decline. Quadratic model better fits data trajectory, indicating a peak in activity at cycle 3.
	Use/adherence/engagement	**Evaluation of adherence to step goals and survey completion (number of non-wear days, completion percentage of daily surveys)**<br>High adherence, with only 10 participants missing step data; 90% of surveys completed, with some non-wear days during days 40–70.
**Leitner (2022)**	Effects on clinical health outcomes	**Comparison of BP changes in mmHg**<br><u>Mean BP Change (mmHg):</u><br>Control group: -0.3 (SBP), -0.9 (DBP)<br>Experimental group 1: -3.8 (SBP), -2.3 (DBP)<br>Experimental group 2: -4.0 (SBP), -4.7 (DBP)<br>Between-group significance: Experimental group 2 had a significantly greater DBP reduction than experimental group 1 (no p-value/CI reported).<br>Experimental groups 1 and 2 achieved a significant BP reduction compared to the control group.<br><u>Maximum BP Change (mmHg):</u><br>Control group: -3.3 (SBP), -2.5 (DBP)<br>Experimental group 1: -10.5 (SBP), -8.8 (DBP)<br>Experimental group 2: -9.9 (SBP), -8.3 (DBP)<br>Both experimental groups showed significantly greater reductions in maximum BP compared to the control group.
	Use/adherence/engagement	**Recommendation Compliance (Experimental group 2) in percentage of compliance**<br>Compliance was highest in the Stress & Diet category (90%) and lowest in Activity (42%). Overall, 60% of the 192 recommendations had sufficient data to assess compliance, indicating moderate to good adherence to personalized recommendations.
**Lim (2016)**	Effects on health behavior	**Change in exercise frequency per week**<br>Significant decrease in both groups, but the decrease was greater in the u-healthcare group than the SMBG group (-8.5% vs. -2.4%, p < 0.05).
	Effects on health behavior	**Change in average caloric intake (% caloric change)**

		Greater reduction in caloric intake in the u-healthcare group than the SMBG group (-8.5% vs. -2.4%, $p < 0.05$).<br>No significant between group-difference (no exact p-value/CI reported).
	Effects on weight	**Change in waist circumference in cm**<br>Reduced significantly in the u-healthcare group (95.1 [7.9] to 92.9 [8.6] cm, p = 0.002). No significant changes in the SMBG group (96.5 [9.5] to 94.9 [9.9], p = 0.065).<br>Significant between group-difference (no p-value/CI reported).
	Effects on weight	**Change in Body Mass Index (BMI) in kg/m^2**<br>Reduced significantly in the u-healthcare group (26.3 [3.5] to 25.7 [3.6], p = 0.002). No significant changes in the SMBG group (26.8 [3.4] to 26.5 [3.7], p = 0.110).<br>Significant between group-difference (no p-value/CI reported).
	Effects on clinical health outcomes	**Changes in HbA1c (%)**<br>Significantly decreased in the u-healthcare group (8.0 [0.7] to 7.3 [0.9] %, $p < 0.001$). No significant change in the SMBG group (8.1 [0.8] to 7.9 [1.2] %, p = 0.936).<br>Significant between group-difference (no p-value/CI reported).
	Effects on clinical health outcomes	**Target HbA1c (HbA1c levels <7.0% (53.0 mmol/mol) without hypoglycemia) in percentage of patients**<br>Patients achieving HbA1c <7.0% were significantly higher in the u-healthcare group (26%) than in the SMBG group (12%; $p < 0.05$).<br>Patients with >1% HbA1c reduction were higher in the u-healthcare group (58%) than the SMBG group (18%; $p < 0.05$).<br>In per-protocol analysis: 67.4% vs. 21.4% ($p < 0.05$).
	Effects on clinical health outcomes	**Glucose lowering medication adjustment (% of participants)**<br>11.6% of the u-healthcare group reduced their medication dosage, compared to none in the SMBG group.
**Lin (2015)**	User experiences	**Participant satisfaction (feedback survey with 5-point Likert scale)**<br>Both groups rated >4 on most metrics, except weight loss and physical activity (slightly lower). Most participants (95%) would recommend the TRIMM program. Intervention-group participants reported finding the messages pleasant, mean response of 4.3 on the 5-point Likert scale. 93% of TRIMM participants reported that they would find it helpful to continue receiving text messages.
	Effects on weight	**Change in weight in kg**<br><u>Intervention group:</u> Weight decreased significantly from a mean of 101.8 kg at baseline to 99.1 kg at 3 months (mean difference −2.6 kg, 95% CI: −3.8 to −1.5) and to 98.1 kg at 6 months (mean difference −3.7 kg, 95% CI: −5.3 to −2.1).<br><u>Control group:</u> Weight remained virtually stable, with a mean weight of 101.2 kg at baseline, 101.1 kg at 3 months (mean difference −0.2 kg, 95% CI: −1.0 to 0.7) and 101.0 kg at 6 months (mean difference −0.2 kg, 95% CI: −1.4 to 1.0).<br>TRIMM participants lost more weight than those in standard care. The mean between-group difference in weight change from baseline was −2.5 kg (95%CI: −4.3 to −0.6; $p < 0.001$) at 3 months and −3.4 kg (95% CI: −5.2 to −1.7; p = 0.001) at 6 months.
	Use/adherence/engagement	**Percentage of days participants responded to daily interactive messages**<br>Engagement decreased from 66% in month 1 to 37% in month 6, with a 6-month mean of 47.6%. Each additional response day correlated with greater weight loss (0.1 kg at 3 months, significant; 0.03 kg at 6 months, non-significant).
**Nezami (2022)**	User experiences	**Program satisfaction (N=68) in percentage**<br>Satisfaction was high in both groups (Standard: 82%; Simplified: 81%). Standard group more likely to continue tracking (mean score 5.7 vs. 4.3, p = 0.025).
	Effects on health behavior	**Daily caloric intake reduction in kcal**<br>Standard group reduced daily intake by -247.3 (95% CI: -500.1 to 5.5) kcal, and Simplified group by -295.9 kcal (-581.3 to -10.6; p = 0.87).
	Effects on weight	**Change in weight in percentage and kg**

		The Standard group had an average PWL of −5.7% (95% CI: −8.3% to −3.2%) and weight loss of 5.9 kg (SD 8.3), while the Simplified group had a PWL of −4.0% (95% CI: −5.7% to −2.3%) and a weight loss of 3.5 kg (SD 4.5). No significant between-group differences were observed.
	Effects on weight	**Achievement of 5% and 10% weight loss in percentage of participants**<br>No significant difference between groups: ~43% in both groups achieved 5% weight loss, while 18.9% (Standard) and 14.3% (Simplified) achieved 10% weight loss.
	Use/adherence/engagement	**Frequency of dietary tracking days**<br>No between-group difference in dietary tracking days (Standard: 89.2 days, Simplified: 86.2 days).
**Novak (2024)**<br>**Vetrovsky (2023)**	User experiences	**Patient experience with mHealth intervention during pilot phase (average number of text messages per week and qualitative feedback)**<br>Patients received 9.2 ± 10.6 messages per week. Five felt the frequency was excessive; three indicated poor timing of prompts, especially during inappropriate times (e.g., while at work). To improve user experience, the frequency of messages was reduced, just-in-time prompts were refined, and message content was varied to prevent repetitiveness.
**Pellegrini (2015)**	User experiences	**NEAT! Acceptability (agreement in percentage and feedback responses)**<br>All participants found the technology motivating, with 87.5% expressing willingness to continue using it. Positive feedback included acceptability of intervention design (prompts, intervention options), perceived efficacy and ease of use, though accelerometer battery life and connectivity issues were noted.
	Effects on health behavior	**Changes in sedentary behavior and physical activity**<br>Among 7/8 participants, daily sedentary time decreased by 8.1% (SD 4.5; p = 0.003) and daily time spent in light-intensity physical activity increased by 7.9% (SD 5.5; p = 0.009). Including data from the eighth participant shifted the reduction in sedentary time to the level of a trend (p = 0.08) while still showing a significant increase in light activity (p =0.047). Over the month, the number of sedentary breaks decreased by an average of 15.8 breaks (SD 8.8; p = 0.003), and the duration of each break extended by an average of 1.0 minutes (SD 0.5; p = 0.002).
	Use/adherence/engagement	**NEAT! Usage**<br>Participants used NEAT! for an average of 21.9 days, 7.6 hours per day. Participants were prompted to stand approximately 5.8 times/day, selecting the "stand" option 62.6% of the time.
**Plaete (2015)**<br>**Poppe (2017, 2018, 2019a, 2019b)**	User experiences (Poppe 2018)	**User experiences with MyPlan 2.0 (qualitative feedback)**<br>While many participants didn't find the information new, they noted increased awareness of their SB or lack of PA. Action planning and evaluation of action plans were particularly appreciated for promoting behavior change. There was mixed feedback on the usefulness of the barrier identification. Participants appreciated the user-friendliness, simplicity, conciseness, and time efficiency of the website. Most participants were already informed about possible benefits but noted benefits to mental health. Some questioned the relevance to their physical health because they did not experience any physical changes themselves. Several participants experienced encouragement from family members, which facilitated adherence to the action plans.
	Effects on health behavior (Poppe 2019)	**Total sitting time (minutes per day)**<br>CG: Pre = M 553.06 (SD 174.05), Post = M 567.94 (SD 211.84).<br>IG-PA: Pre = M 592.83 (SD 232.52), Post = M 470.38 (SD 185.23).<br>IG-SB: Pre = M 599.17 (SD 133.58), Post = M 579.44 (SD 188.84).<br>Borderline significant intervention effect favoring the PA intervention group (p = 0.09).
	Effects on health behavior (Poppe 2019)	**Daily breaks from sedentary time (breaks per day)**<br>CG: Pre = M 16.63 (SD 2.25), Post = M 15.65 (SD 2.87).<br>IG-PA: Pre = M 15.51 (SD 3.08), Post = M 14.64 (SD 2.39).<br>IG-SB: Pre = M 16.88 (SD 1.69), Post = M 17.50 (SD 1.45).<br>Significant intervention effect favoring the SB intervention group (p = 0.005).

	Effects on health behavior (Poppe 2019)	**Moderate-to-Vigorous Physical Activity (MVPA) with accelerometer**<br><u>CG:</u> Pre = M 23.20 (SD 12.92), Post = M 19.36 (SD 14.77).<br><u>IG-PA:</u> Pre = M 17.07 (SD 15.68), Post = M 25.50 (SD 15.77).<br><u>IG-SB:</u> Pre = M 20.23 (SD 13.87), Post = M 19.38 (SD 13.81).<br>Borderline significant intervention effect favoring the PA intervention group (p = 0.049).
	Use/adherence/engagement (Poppe 2018)	**Website usage (minutes and number of participants)**<br>Average total time on website: 48.8 minutes (SD = 23.1; range = 17–111)<br>Session 1: M 22.2 min (SD 10.8; range = 9–46), 15 participants visited optional pages (71.4%)<br>Session 2: M 7.1 min (SD 4.4; range = 2–19), 13 participants (61.9%)<br>Session 3: M 6.8 min (SD 4.3; range = 2–21), 18 participants (85.7%)<br>Session 4: M 6.0 min (SD 3.8; range = 1–15), 13 participants (61.9%)<br>Session 5: M 6.5 min (SD 6.3; range = 1–30), 17 participants (81.0%)
**Radhakrishnan (2020, 2021)**	User experiences (Radhakrishnan2021)	**Participant feedback on game features, barriers, and motivation for self-management (survey)**<br>87% (13/15) of participants completed the survey. Positive themes included competitive nature, motivation, ease of the interface, and opportunity to learn. Barriers included syncing issues, unrealistic step goals, and simplistic/not appealing features.
	Effects on quality of life (Radhakrishnan2021)	**Changes in quality of life as measured by KCCQ**<br>Both groups demonstrated a clinically and statistically significant within-group increase in quality of life at 6, 12, and 24 weeks compared with baseline on the KCCQ.
	Effects on health behavior (Radhakrishnan2021)	**Change in steps per day**<br>The IG showed modest increases (2742 [SD 2499] to 3365 [SD 2821]), while the CG had decreases in physical activity (2638 [SD 1573] to 2444 [SD 1757]).
	Effects on clinical health outcomes (Radhakrishnan2021)	**Improvement in functional status as measured by KCCQ scores**<br>Clinically significant 7-point increase at 6 weeks in the IG. Both groups showed sustained improvements at 24 weeks.
	Use/adherence/engagement (Radhakrishnan2021)	**Frequency of weighing behaviors (mean days per week)**<br>The IG showed a 40% increase in weighing 5+ days per week and a decrease of 6% was observed in the CG (effect size = 0.53).
	Use/adherence/engagement (Radhakrishnan2021)	**Device and app usage, including game engagement among IG participants (percentage of participants)**<br>71% (11/15) of IG participants used the game app more than 50% of the days.<br>Game engagement was positively correlated with weighing and physical activity data (r = 0.72 and r = 0.9, respectively).
**Reinwand (2013)**<br>**Storm (2016)**	Use/adherence/engagement (Storm 2016)	**Intervention session attendance (number of sessions attended, percentage of participants completing session)**<br>Participation declined from 90.8% (314/790) in the first session to 19.9% (69/790) participation in the last session of the 8-week intervention. On average, participants completed 2.0 sessions (SD 2.4), with most completing only one session (41.9%).
**Richardson (2007, 2010)**	Effects on health behavior (Richardson2010)	**Average daily step counts in steps per day**<br>Daily steps increased significantly in both arms (intention-to-treat: +1888 steps/day; p < 0.001 and completers: +2477 steps/day; p < 0.001).<br>No significant differences between arms in step-count increases (p = 0.82).
	Use/adherence/engagement (Richardson2010)	**Intervention website exposure (number of homepage hits and views of tailored messages)**<br>The online community arm had more homepage hits (p = 0.02), but no differences in views of tailored messages were observed.
	Use/adherence/engagement (Richardson2010)	**Online community use (percentage of participants using the community; number of posts and views)**<br>65% of participants used the online community (posters or lurkers). The community provided empathy, encouragement, and social support. Both participants and staff referenced noncommunity components frequently.

	Use/adherence/engagement (Richardson2010)	**Program engagement (percentage of days with uploads)**<br>Online community participants uploaded data more frequently (87% vs. 75%, p = 0.001) and had higher completion rates (79% vs. 66%, p = 0.02).
**Schoenthaler (2020)**	User experiences	**User perceptions about messages (patients, qualitative feedback)**<br>Patients found messages helpful, non-intrusive, and easy to use. The number of messages sent was generally adequate. Suggestions included more motivational content and adjustments in message timing and wording. Qualitative feedback confirmed alignment with high scores on TAM3 survey results for the PRO (e.g. intention to use over time, perceived usefulness, use behavior, social influences)
	User experiences	**User perceptions about Personalized Reports Feedback (patients, qualitative feedback)**<br>The majority of patients (8/9, 89%) felt the report was easy to read, eye-catching, and comprehensive. Patients preferred the 1-month report for readability. Several patients had difficulty reading the bar graphs of PROs that were collected biweekly. Motivational messages and insights were added based on feedback. Benefits of using the personalized report for diabetes self-management included being able to analyze how well one is adhering to recommended diabetes behaviors, providing visual cues to take responsibility for one's health, and providing support to stay on track to be successful with diabetes. Qualitative feedback confirmed alignment with the scores on TAM3 survey results for the report (e.g. intention to use over time, perceived usefulness, use behavior, social influences).
	User experiences	**Providers' evaluation of integrating reports into clinical practice and patient education needs (qualitative feedback)**<br>Providers appreciated the personalized reports as valuable tools for engaging patients in diabetes self-management, particularly for visualizing trends and discussing key behaviors during consultations. They recommended pre-program education on diabetes guidelines and medication. To integrate the reports into clinical workflows, they suggested adding a dedicated tab in the EHR (Epic) with summary views. Based on this feedback, the study team enhanced the reports with insights to motivate behavioral changes and worked on seamless Epic integration.
	Use/adherence/engagement	**Use behavior (patients, quantitative counts and minutes)**<br>Initial response rate 78.7% (256/325), improved to 84.6% (188/222) after protocol adjustments; issues with message format and timing were addressed.
**Shibuta (2023)**	User experiences	**Perceived usefulness of the intervention system (ratings of system usability)**<br>55% (16/29) of participants found the system useful, and 41% (12/29) somewhat useful. Participants spent 16.5 minutes/day on average, with 90% (26/29) agreeing it was worth the time.
	Effects on health behavior	**Change in median steps per day**<br>Significant increase from P0b to P1b (+1493 steps/day, p < 0.001).<br>No significant increase from P0b to P2b (+1056 steps/day, p = 0.04).<br>Subgroup analysis showed less-active participants had a significant increase at P1b (median +1451; 95% CI +111 to +3220 steps per day; p = 0.005 vs median +1958; 95% CI −544 to +3055 steps per day; p = 0.05 in the more active group).<br>Both groups did not significantly change steps per day at P2b.
	Effects on weight	**Change in body weight in percentage and Body Mass Index (BMI) in kg/m^2**<br>Significant decreases in body weight and BMI at both P1a (p < 0.001) and P2a (p = 0.001).<br>Median body weight reductions: 1.8% (P1a) and 2.1% (P2a).
	Effects on clinical health outcomes	**Change on blood pressure (BP) in mmHg**<br>BP did not change significantly at P1b but decreased significantly at P2b (all p < 0.025), except for morning systolic BP (p = 0.07).
	Effects on clinical health outcomes	**Change in HDL cholesterol in mg/dL**<br>Significant increase at P1a compared to P0b (p < 0.001), but not at P2a (p = 0.23).
	Use/adherence/engagement	**The evaluation of participant engagement (percentage of data recorded)**

		Median step count measurements were 96.4% (IQR 85.7%-100%) during P0b and 97% (IQR 88.7%-100%) during the intervention. Recording of other parameters exceeded 85%. 23% (7/30) of participants recorded any exercise. System issues were reported by 79% (27/34) of participants, mostly related to step count recording (50%, 31/62)).
**Steinberg (2020)**	User experiences	**Satisfaction with intervention activities (percentage agreement or disagreement with statements)**<br>82% found the Nutritionix app easy to use; 50% would use it frequently. 79% of the intervention arm would recommend the intervention to others. 79% of the participants felt the DASH dietary pattern tips were easy to understand. 55% found the DASH score helpful, and 28% felt the text messages were personalized. 34% reported that the text messages helped them reach their diet goals. 76% agreed the DASH score of 10 was hard to achieve.
	Effects on health behavior	**Change in DASH adherence (DASH score, scale; mean/median change in nutrient intake)**<br>Baseline DASH score: Intervention (2.2, SD 1.3) vs active comparator (2.4, SD 1.3); p = 0.85.<br>DASH score change (3 months): Intervention (+0.8, 95% CI: 0.2-1.5; p = 0.02) and active comparator (+0.8, 95% CI: 0.4-1.2; p < 0.001).<br>Participants in the intervention arm had DASH scores that were 0.01 (95% CI: –0.7 to 0.7) points lower than the active comparator arm at 3 months (p = 0.97). No between-group differences; nutrient changes included increased fiber and reduced saturated fat in both arms.
	Effects on clinical health outcomes	**Change in blood pressure in mmHg**<br>Systolic BP: Intervention vs comparator: –2.8 mmHg (95% CI: –1.8 to 7.4; p = 0.23)<br>Diastolic BP: Intervention vs comparator: –3.6 mmHg (95% CI: –0.2 to 7.3; p = 0.07)
	Use/adherence/engagement	**Engagement with diet tracking (mean days per week tracked; proportion of participants tracking ≥5 days/week; change in days/week over time)**<br>Mean days tracked: Active comparator (4.6, SD 2.7) vs intervention (4.2, SD 2.1); p = 0.54. ≥5 days/week: Active comparator (63%) vs intervention (47%); p = 0.24. Intervention group experienced a steeper reduction in diet-tracking engagement over time, with rates decreasing by 0.23 (95% CI: 0.16-0.29) days per week (p < 0.001), which is about a day per month faster than the active comparator group.
**Sun (2020)**	User experiences	**Satisfaction with the incentive mechanism**<br>Participants earned an average of 5,620 points (SD 2,720) and expressed overall satisfaction. However, motivation was context-dependent, with barriers such as being busy, tired, or the effort required in certain settings (e.g., workplaces). Participants suggested that larger rewards for challenging contexts might improve motivation.
	Use/adherence/engagement	**Adherence rate of the JIT intervention (Percentage adherence (%) and the number of intervention messages per day)**<br>Participants received an average of 3.1 messages per day (SD 0.9). The adherence rate was low, averaging 9.4% (SD 5.0). Participants attributed this to poor receptivity due to frequent messages causing "intervention fatigue." Contextual barriers, such as timing and availability, were mentioned as limitations.
**Sze (2023)**<br>**Waki (2024)**	User experiences	**Participant satisfaction with app features (% satisfaction rates)**<br>78.1% were "satisfied" or "very satisfied" with the app. The app features that most people found useful for self-management of diabetes included the body weight, blood pressure, and step count measurement and recording function. Features like personalized goal setting and progress monitoring were found "just right" by over 80% in terms of frequency. 84.4% would continue using it if recommended by their doctor.
	Effects on health behavior	**Change in average steps per day**<br>Step count increased from 5436 (SD 2231) to 10,150 (SD 3908; p < 0.0001), representing an 86.7% increase in the mean.<br>The step goal achievement rate remained steady and high throughout the intervention, with most patients routinely recording more steps than their daily goal.
	Effects on weight	**Change in Body Mass Index (BMI) in kg/m^2**<br>BMI reduced significantly (28.7 SD 3.7 vs 28.4 SD 3.6, p = 0.0038).

	Effects on clinical health outcomes	**Change in HbA1c (%)**<br>HbA1c reduced from 8.58 SD 1.02 to 7.79 SD 1.11 (p = 0.0001).<br>Subgroup analysis (no medication change) showed a significant reduction of -0.73% (95% CI: -1.04, −0.43).
**Tabak (2013, 2014a, 2014b, 2014c)<br>Wieringa (2011)**	User experiences<br>(Tabak 2013)	**Acceptance and Usability of the Activity Coach (5-point or 7-point Likert scale)**<br>Performance Expectancy: 60% of patients responded positively, indicating they believe the activity coach is useful but may not significantly improve their health complaints (mean score: 3.2/5).<br>Effort Expectancy: 76% responded positively, perceiving the intervention as requiring minimal effort to use.<br>Intention to Use: 65% expressed a willingness to use the activity coach in the future. Preferred durations of use were as follows: 45% "always," 15% "1 year," 5% "3 months," 15% "1 month," 15% "1-2 weeks," and 5% "never."<br>Activity Graph: Mean score of 5.4 (7-point scale). Patients appreciated the color scheme (score: 6.3) but found the graph less clear (score: 4.7).<br>Motivational Cues: Mean score of 5.5. Cues were regarded as readable and motivating.
	Effects on health behavior<br>(Tabak 2014a)	**Response to motivational cues in percentage change in activity level**<br>809 motivational cues were used of which 250 (31%) encouraging, 421 (52%) neutral, and 138 (17%) discouraging cues.<br>Discouraging cues: Significant reduction in activity levels at all intervals, with the largest decrease at 30 minutes (-29%, p < 0.001).<br>Encouraging cues: Significant increase in activity levels within the first 5–10 minutes (+23% at 5 min, p = 0.005; +15% at 10 min, p = 0.017).<br>Neutral cues: No significant response at any interval (-3% to -6%, p > 0.05).
	Effects on health behavior<br>(Tabak 2014a)	**Change in activity levels and balance (activity (CPM), balance (%), and percentage change in activity levels)**<br>On a group level, no significant changes in activity or balance were observed. Corrected activity levels (adjusted for reactivity) showed significant improvement (+13%, p = 0.008). Response to encouraging cues correlated with increased activity levels (rs = 0.66, p = 0.026) but not to balance.
	Effects on clinical health outcomes<br>(Tabak 2014b)	**Exacerbations and hospitalizations for the Condition Coach (number of events; median duration in days)**<br>Exacerbations (telehealth group only): Total: 33 exacerbations (median 2.0, IQR 1.0–3.0). One patient experienced 11 exacerbations. Some exacerbations occurred within 28 days of the previous one. Data for exacerbations in the control group were not available.<br>Hospitalizations for COPD:<br>Telehealth group: 4 hospitalizations. Median length of stay 5.5 days (IQR 4.8-6.3).<br>Control group: 5 hospitalizations. Median length of stay 7.0 days (IQR 6.0-7.0). 5 Emergency Room (ER) visits in both groups.
	Use/adherence/engagement<br>(Tabak 2014b)	**Use of the Activity Coach (number of days the activity coach module was used in feedback or monitoring mode)**<br>The activity coach was used for 299 days (132 days in monitoring mode, 167 days in feedback mode). The activity coach was rarely used outside of the measurement weeks (T0-T4).
**vanderWeegen (2013, 2015)<br>Verwey (2014a, 2014b)**	Effects on health behavior<br>(vanderWeegen 2015)	**Change in minutes of moderate and vigorous physical activity**<br>Group 1 (Tool +SSP) improved +9.53 minutes from baseline to T2.<br>Group 2 (SSP) showed -2.13 minutes from baseline to T2.<br>Group 3 (usual care) decreased -1.73 minutes from baseline to T2.<br>At 4-6 months (T1):<br>Tool & SSP vs. Care as usual: Significant difference with an adjusted mean difference of +11.73 (95% CI: 6.21, 17.25; p < 0.001).<br>Tool & SSP vs. SSP: Significant difference with an adjusted mean difference of +7.86 (95% CI: 2.18, 13.54; p = 0.003).<br>At 9 months (T2):<br>Tool & SSP vs. Care as usual: Significant difference with an adjusted mean difference of +10.59 (95% CI: 4.94, 16.25; p < 0.001).<br>Tool & SSP vs. SSP: Significant difference with an adjusted mean difference of +9.41 (95% CI: 3.70, 15.11; p < 0.001).<br>All time points: There were no significant differences between SSP and Care as usual at either time point (p > 0.05).
	Effects on quality of life	**Quality of life RAND Physical Component score**

	(vanderWeegen 2015)	Group 1 (Tool +SSP) increased +1.6 points from baseline to T2.<br>Group 2 (SSP) improved +2.1 point from baseline to T2.<br>Group 3 (usual care) changed 0 points from baseline to T2.<br>At 9 months, SSP scored significantly higher than care as usual (adjusted mean difference: +2.99, p = 0.005), and significantly higher than Tool + SSP (-2.65, p = 0.020). No statistically significant improvement for Tool+SSP versus care as usual (+0.34, p = 1.000)
	Effects on quality of life (vanderWeegen 2015)	**Quality of life RAND Mental Component score**<br>Group 1 (Tool +SSP) increased +0.1 points from baseline to T2.<br>Group 2 (SSP) improved +1.5 point from baseline to T2.<br>Group 3 (usual care) increased 0.2 points from baseline to T2.<br>At 4-6 months, both intervention groups scored higher than care as usual (+3.23, p = 0.04; +4.39, p = 0.002).
**vanGenugten (2010, 2012, 2014)**	User experiences (vanGenugten 2012)	**Perceived Information Quality (mean scores on 1-5)**<br>TI group reported lower reading rates than the GI group (TI 4.07 vs. GI 4.5, p < 0.001) and were and were slightly less positive about the lengths of the texts (TI 3.20 vs GI 3.07, p = 0.01). TI content perceived as more individualized than the GI group (3.20 vs. 2.83, p = 0.001). No significant differences in usefulness or attractiveness.
	Effects on health behavior (vanGenugten 2012)	**Change in minutes of physical activity per day, self-reported by participants**<br>PA decreased significantly in the total population over time (p = 0.002), but the group*time interaction was not significant (p = 0.44).<br>Mean differences at 1 month were -1.2 minutes (TI) and -0.7 minutes (GI).<br>Mean differences at 6 months were +0.2 minutes (TI) and +9.1 minutes (GI).
	Effects on health behavior (vanGenugten 2012)	**Change in fat intake per week**<br>Mean fat intake decreased significantly over time (p < 0.001) with no significant group*time interaction (p = 0.74).<br>The mean difference at 1 months was -1.7 (TI) and -1.6 (GI).<br>The mean difference at 6 months was -1.6 (TI) and -1.4 (GI).
	Effects on weight (vanGenugten 2012)	**Change in Body Mass Index (BMI) in kg/m^2**<br>BMI did not change significantly over time (p = 0.09), and no significant group*time interaction effect was observed (p = 0.09).<br>The mean difference between baseline and 6-month was -0.08 (TI) and -0.30 (GI).
	Effects on weight (vanGenugten 2012)	**Change in waist circumference in cm**<br>Waist circumference decreased significantly over time (p < 0.001), but there was no significant group*time interaction effect (p = 0.12).<br>The mean difference was -1.48 cm (TI) and -2.46 cm (GI).
	Use/adherence/engagement (vanGenugten 2014)	**Module Visitation Rates (only tailored intervention [TI]) in percentage of participants**<br>Module visitation rates:<br>First module (93.3%, 251/269)<br>Second module (74.1%, 199/269)<br>Third module (26.7%, 71/269)<br>Fourth module (15.2% 40/269).<br>Mean visits: 1.8 (median: 1).
**Watson (2012)**	User experiences	**Participant benefits and satisfaction (% of participants)**<br>Benefits: 93% (28/30) intervention vs. 90% (28/31) control reported benefits (p = 0.67). Behavioral changes: More frequent exercise (86% (25/29) intervention vs. 72% (21/29) control, p = 0.19); improved diet (45% (13/29) intervention vs. 21% (6/29) control, p = 0.05). Intervention-specific: 58% (18/31) found the virtual coach motivating, 87% (27/31) felt guilty skipping appointments.
	Effects on health behavior	**Percentage change in step count**

		Intervention group step count remained stable (6943 to 7024, p = 0.85), while the control group showed a significant decline (7174 to 6149, p = 0.01). Significant percentage change difference observed between P1 and P3 (p = 0.02); changes from P1 to P2 and P1 to P4 were not significant (p = 0.12 and 0.07, respectively). Repeated measures ANOVA: significant difference across all periods in the intervention versus control arms (p = 0.02).
	Effects on weight	**Change in Body Mass Index (BMI) in kg/m²**<br>No significant changes in weight, BMI (mean reduction: 0.04 control vs. 0.25 intervention, p = 0.44).
	Use/adherence/engagement	**Engagement and protocol adherence**<br>Recorded step data days: 87% (73/84) control vs. 85% (71/84) intervention (p = 0.64).<br>Mean intervention sessions: 28.9 per participant (range 3–63, recommended 36).<br>Weekly visits decreased from 2.8 in week 1 to 1.9 in week 12 (p = 0.08).<br>No significant correlation between session frequency and step count performance.
**Yom-Tov (2017)**	User experiences	**Satisfaction with feedback messages (percentage of positive responses)**<br>Both control and learned policy group participants reported increasing their physical activity. Personalized messages significantly increased self-reported activity frequency compared to the control group (p < 0.01). Control group found messages less helpful.
	Effects on health behavior	**Change in activity (fraction of target) and steps per minute**<br>Personalized feedback (learned policy) increased activity and walking rate significantly compared to the control group (linear slopes positive for learned policy).
	Effects on health behavior	**Change in activity based on different types and sequences of messages**<br>Positive-social messages led to the highest activity increase, whereas negative messages and positive-self messages led to a decrease in the amount of activity. The differences in the change of activity between the initial policy and the learned policy were statistically significant (ANOVA p = 0.004). Time-dependent feedback correlated with greater activity change (p = 0.02).
	Effects on clinical health outcomes	**Change in HbA1c levels (%)**<br>Average HbA1c decreased by 0.28% (SD 0.84).<br>Personalized feedback, higher initial HbA1c, and lower activity targets led to greater reductions (R^2=0.405, p < 0.01).
	Use/adherence/engagement	**App usage duration**<br>App data was available for 20 weeks (SEM 1.6).<br>No significant difference between treatment and control groups (p = 0.30).
**Zahedani (2023)**	Effects on health behavior	**Change in daily physical activity in minutes**<br>Adjusted minutes/day increased from 49 to 97 min/day.<br>Significant subgroup increases: 50–109 min/day (healthy nondiabetics), 45–73 min/day (prediabetics).<br>No significant change in T2D participants (45 to 46 min/day).
	Effects on health behavior	**Change in macronutrient composition and caloric intake (grams and percent of total calories)**<br>Decreases: caloric intake (-21.8%), carbohydrates (-2.6%), sugar (-12.3%), saturated fat (-0.5%).<br>Increases: protein (+3.45%), total fat (+1.1%), fiber (+7.9%).<br>Dietary changes indicate improvement in macronutrient composition.
	Effects on weight	**Change in body weight in pounds (lbs) and percentage of body weight**<br>28 days (N=567): All groups of individuals significantly decreased their body weight over 28 days (p < 0.0001).<br>75.5% of the 567 participants lost weight over the first 28 days.<br>Average weight loss of 2.5 lbs (nondiabetic/prediabetic) and 4.4 lbs (T2D).<br>12 weeks (N=137): Weight loss of 2.6 lbs (nondiabetic, p < 0.0001), 6.8 lbs (prediabetic, p = 0.003), 9.4 lbs (T2D, p = 0.0007).

		Greater weight loss observed in individuals with higher starting weights.<br>Percent loss at 12 weeks: 1.5% (nondiabetic), 2.3% (prediabetic), 5.1% (T2D).
	Effects on clinical health outcomes	**Change Time in Range (%, in range: 70–180 mg/dL for T2D, 70–140 mg/dL without T2D)**<br>Baseline TIR: 82% (T2D), 91% (prediabetes and healthy nondiabetics).<br>TIR did not significantly improve for the group as a whole.<br><u>Suboptimal baseline (<90% TIR):</u> TIR increased by 9.8% (T2D), 6.2% (prediabetes), 9.6% (healthy nondiabetics).<br><u>Baseline TIR <70%:</u> TIR increased by 13.2% (T2D), 9.6% (prediabetes), and 22% (healthy nondiabetics, $p < 0.0001$).<br>Only the healthy nondiabetic group reached statistical significance.
	Effects on clinical health outcomes	**Change in Glucose Management Indicator (GMI = Estimated HbA1c derived from CGM data) in percentages**<br><u>GMI baseline >7%:</u> decreased by 0.43% ($p < 0.001$).<br><u>GMI baseline >6%:</u> decreased by 0.22% ($p < 0.00001$).<br>Reductions were significant across all glycemic subgroups.

[1] We only reported relevant outcomes for the review aims in this table. A more detailed overview of results can be requested from the authors.
